# A modular synthetic route to size-defined immunogenic *Haemophilus influenzae* b antigens is key to the identification of an octasaccharide lead vaccine candidate†
†Electronic supplementary information (ESI) available: Experimental methods, compound characterization including spectra, supplementary figures and table, conjugation and immunization details. See DOI: 10.1039/c7sc04521b


**DOI:** 10.1039/c7sc04521b

**Published:** 2017-12-11

**Authors:** J. Y. Baek, A. Geissner, D. C. K. Rathwell, D. Meierhofer, C. L. Pereira, P. H. Seeberger

**Affiliations:** a Max Planck Institute of Colloids and Interfaces , 14476 Potsdam , Germany . Email: Peter.Seeberger@mpikg.mpg.de ; Email: claney.pereira@vaxxilon.com; b Freie Universität Berlin , Department of Chemistry and Biochemistry , 14195 Berlin , Germany; c Max-Planck Institute for Molecular Genetics (MPIMG) , 14195 Berlin , Germany

## Abstract

The first glycoconjugate vaccine using isolated glycans was licensed to protect children from *Haemophilus influenzae* serotype b (Hib) infections. Subsequently, the first semisynthetic glycoconjugate vaccine using a mixture of antigens derived by polymerization targeted the same pathogen. Still, a detailed understanding concerning the correlation between oligosaccharide chain length and the immune response towards the polyribosyl-ribitol-phosphate (PRP) capsular polysaccharide that surrounds Hib remains elusive. The design of semisynthetic and synthetic Hib vaccines critically depends on the identification of the minimally protective epitope. Here, we demonstrate that an octasaccharide antigen containing four repeating disaccharide units resembles PRP polysaccharide in terms of immunogenicity and recognition by anti-Hib antibodies. Key to this discovery was the development of a modular synthesis that enabled access to oligosaccharides up to decamers. Glycan arrays containing the synthetic oligosaccharides were used to analyze anti-PRP sera for antibodies. Conjugates of the synthetic antigens and the carrier protein CRM197, which is used in licensed vaccines, were employed in immunization studies in rabbits.

## Abstract 2

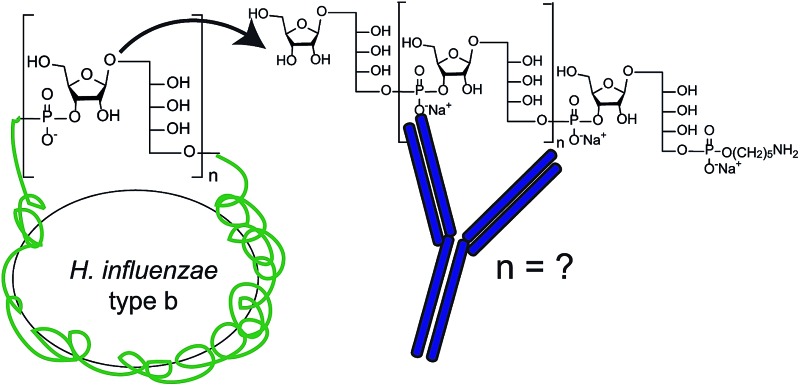
A *Haemophilus influenzae* b vaccine lead antigen was identified by the immunological evaluation of chemically precisely defined capsular polysaccharide repeating unit oligosaccharides.

## Introduction

**Fig. 1:**
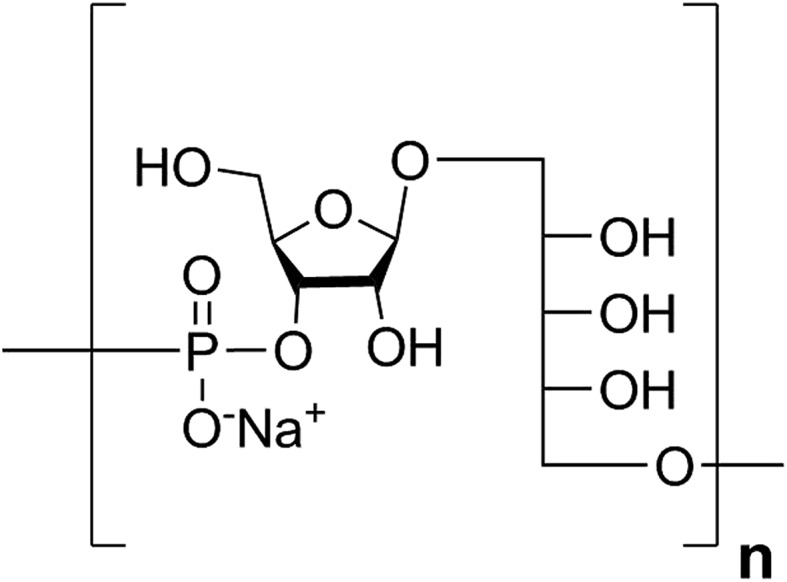
Structure of the Hib CPS RU.

*Haemophilus influenzae* is a major cause of bacterial respiratory tract infections that can lead to severe diseases such as pneumonia, sepsis, and meningitis.^1^ While unencapsulated clones (nontypable *H. influenzae*) often cause local infections like otitis media or sinusitis, invasive disease is usually caused by *H. influenzae* expressing an antiopsonic polysaccharide capsule.^2^ Serotype b (Hib) is coated with a capsular polysaccharide (CPS) made up of polyribosyl-ribitol-phosphate (PRP) repeating units (RU) (Fig. 1) and possesses the highest invasive potential among encapsulated *H. influenzae*; thus, it is a major health concern, especially for children.^3^

This bacterium became the first pathogen for which a glycoconjugate vaccine was licensed.^4^ These glycoconjugate vaccines are produced from PRP that is isolated from bacterial fermentation, often size reduced, and subsequently chemically coupled to a carrier protein that induces a T cell-dependent immune response.^5^ Routine vaccination in many countries has led to significant declines in bacterial burdens.^6^

PRP is an interesting target for carbohydrate synthesis because of both its biological importance and the synthetic challenge inherent in generating longer oligosaccharides to serve as Hib vaccine antigens.^7^–^10^ Chemical synthesis provides a means to access chemically well-defined carbohydrate antigens without the danger of contamination from a pathogen culture.^11^ In the case of Hib, several methods have been used to obtain synthetic PRP (sPRP) oligosaccharides. For example, Just's group synthesized short fragments of Hib PRP as trimers and pentamers by means of solution and solid phase approaches.^12^–^15^ Immunogenicity studies on semisynthetic glycoconjugates^10^,^16^ resulted in the first approved glycoconjugate vaccine in which the oligosaccharide hapten had been accessed by chemical synthesis.^17^ This vaccine, an sPRP-tetanus toxoid (TT) conjugate referred to as Quimi-Hib was developed in Cuba and is now in routine use there and in several other countries.^18^

Though clinically effective, the oligosaccharide component of Quimi-Hib represents a mixture of oligosaccharides, six to eight RUs on average, obtained by polycondensation.^17^ Size is considered to be an important factor in the efficacy of Hib glycoconjugate vaccines, but studies using oligo- and polysaccharides of varying length have yielded conflicting results.^19^ This finding can at least partially be attributed to the use of oligosaccharides obtained by chemically degraded PRP using different methods which do not yield defined oligosaccharides but rather mixtures of different lengths; thus, the lengths of the oligosaccharides that were tested varied considerably. Studies on length-defined sPRP have thus far only focused on a few shorter PRPs.^10^,^20^ Not only for Hib but for all other glycoconjugate vaccines it remains to be determined whether immunogenicity correlates with antigen length. This issue is of central importance for the development of semisynthetic or fully synthetic vaccines.^21^

To provide synthetic oligosaccharide antigens of defined length as tools to address the correlation of antigen length and immunogenicity, a solution-phase strategy using a disaccharide building block and elongation by means of H-phosphonate chemistry was chosen. The resulting glycans were immobilized on glycan arrays for antibody analysis and coupled to CRM197, an approved carrier protein for glycoconjugates,^21^,^22^ for subsequent immunizations. Immunogenicity studies in rabbits with semisynthetic sPRP-CRM197 glycoconjugates reveal that an octasaccharide containing four repeating disaccharide units induces antibody levels similar to those induced by longer oligosaccharides.

## Results and discussion

### Synthetic targets and strategy

**Fig. 2:**
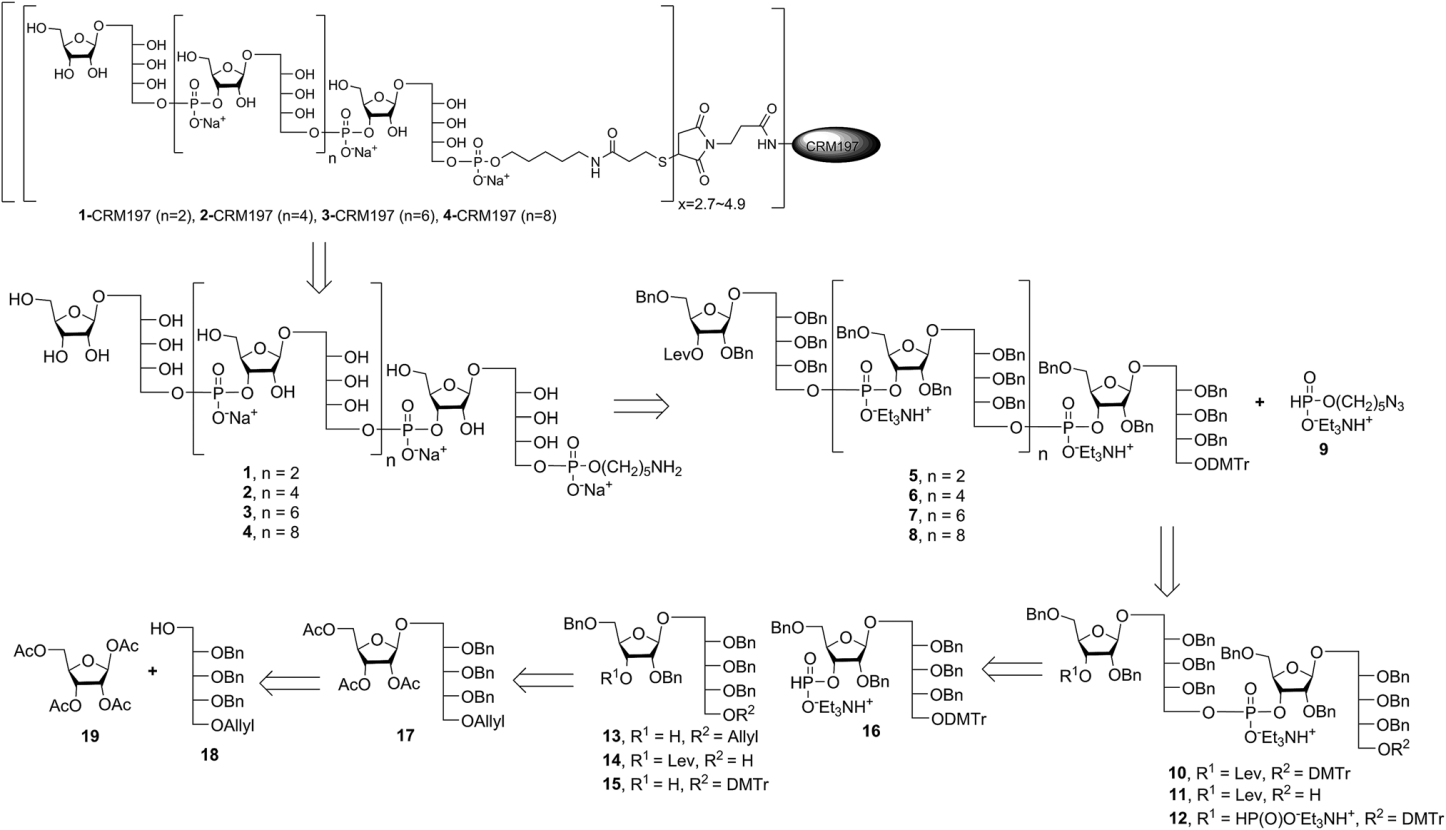
Retrosynthetic analysis of sPRP oligosaccharides **1–4** and respective sPRP-CRM197 glycoconjugates.

Retrosynthetic analysis of sPRP oligosaccharides **1–4** revealed a flexible strategy to access tetramer to decamer corresponding to structures that are present in the mixture of the Quimi-Hib™ vaccine (Fig. 2). Dimer **10**, with its orthogonal protecting groups, is the key intermediate for obtaining compounds **5–8**, and it is in turn produced by coupling disaccharide **14** (ref. 17) with H-phosphonate disaccharide **16**. Disaccharides **13** and **16** can be obtained from **17**, which is the product of β-stereoselective glycosylation between the suitably protected ribitol **18** (ref. 12, 13 and 17) and the commercially available peracetylated β-d-ribofuranose **19**.

### Synthesis of ribitol and ribose–ribitol building blocks

**Scheme 1:**
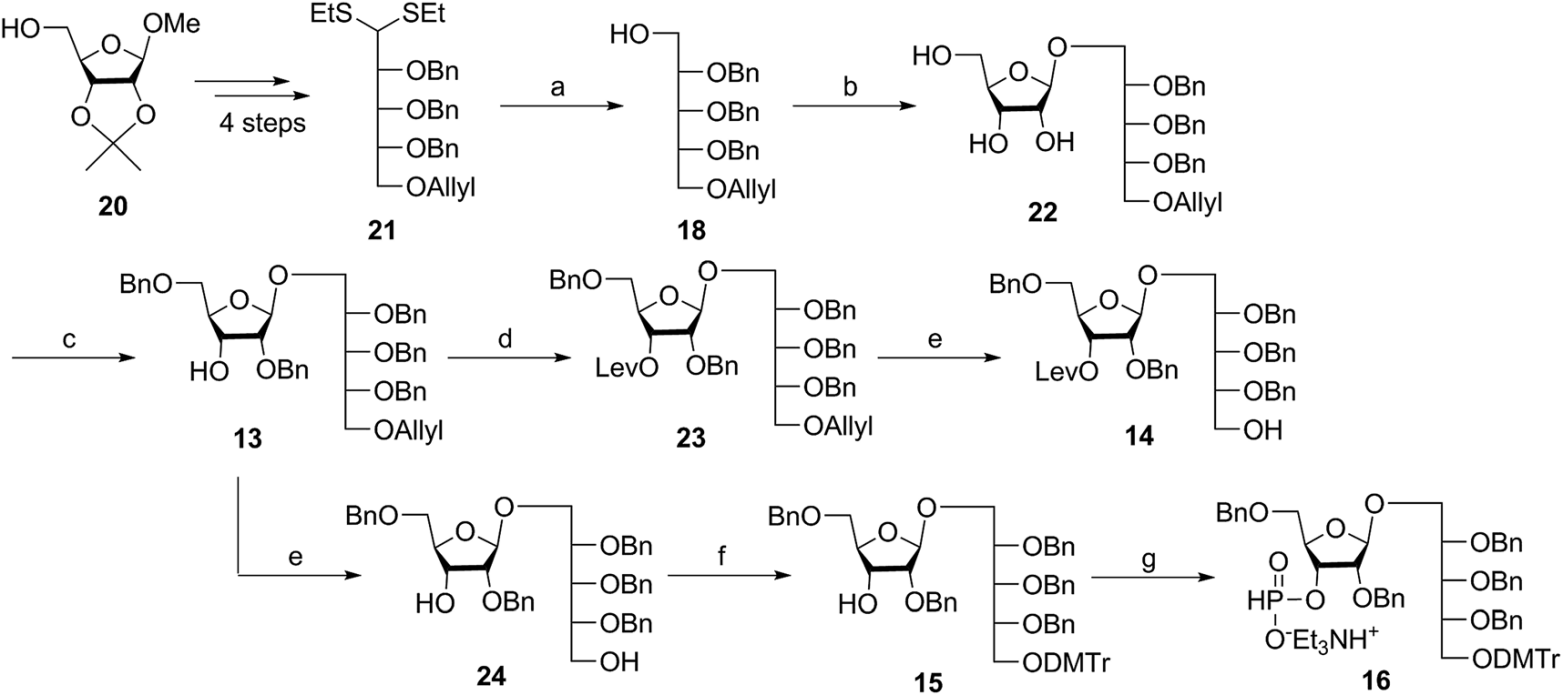
Synthesis of ribitol unit **18** and disaccharides **13**, **14**, and **16**. Reagents and conditions: (a) (i) NIS, acetone, –78 °C; (ii) NaBH_4_, EtOH, 0 °C, 43% (six steps from **20**); (b) (i) **19**, BF_3_·OEt_2_, DCM, –78 °C, 80%; (ii) NaOMe, MeOH, RT, 90%; (c) Bu_2_SnO, toluene, reflux, 2 h, then BnCl, TBAI, NaH, DMF, 50 °C, 2 h, 50%; (d) LevOH, DMAP, DIC, DCM, RT, 93%; (e) Pd(PPh_3_)_4_, DMBA, MeOH, RT, 75% for **14**, 75% for **24**; (f) DMTrCl, DMAP, DCM, RT, 93%; (g) PCl_3_, Et_3_N, imidazole, DCM, 0 °C, 85%. NIS = *N*-iodosuccinimide, TBAI = tetrabutylammonium iodide, DIC = 1,3-diisopropylcarbodiimide, DMAP = 4-dimethylaminopyridine, DMBA = 1,3-dimethylbarbituric acid.

Several syntheses of ribitol unit **18** have been reported,^7^–^10^,^12^–^15^,^17^ but none of these methods is scalable. Ribitol derivative **18**, required for the synthesis of sPRP, was prepared from the known dithioacetal building block **21** (ref. 12 and 13) that was obtained in turn from methyl 2,3-*O*-isopropylidene-β-d-ribofuranoside **20***via* a four step synthesis involving allylation at the 5-*O*-position, cleavage of isopropylidene, furanose ring opening and benzylation.^12^,^13^ Compound **21** was prepared at a larger scale with fewer purifications and improved yields (ESI†). Although hydrolysis of the dithioacetal moiety of **21** had been previously carried out using a mercury(ii) reagent followed by reduction to **18**,^7^–^10^,^12^,^13^ (Scheme 1) the removal of excess mercury(ii) reagent was cumbersome at a large scale and only afforded the intermediate aldehyde in low yield and with poor reproducibility. Thus, to generate ribitol derivative **18**, dithioacetalribitol **21** was instead treated with NIS in aqueous acetone at –78 °C to yield the aldehyde, which was reduced using NaBH_4_, thereby circumventing toxic mercury based reagents. Protected ribitol **18** was readily synthesized at a 30 g scale from methyl 2,3-*O*-isopropylidene-β-d-ribofuranoside with an overall yield of 43% over six steps.

β-Stereoselective glycosylation of **19** with **18** using BF_3_·OEt_2_ in dichloromethane (DCM) provided the disaccharide ribosylribitol **17** in 80% yield,^17^ which was deacetylated under Zemplén conditions to give disaccharide **22** (90% yield). To selectively protect the 2- and 5-*O*-positions of the ribose backbone, the permanent benzyl ether (Bn) protecting group was introduced *via* tin-mediated alkylation,^17^ providing **13** in 50% yield in a single step (Scheme 1). Regioselective benzylation as well as the stereochemical configuration of key disaccharide **13** was confirmed by NMR and these data agree with previous reports.^17^,^23^ Disaccharide **14** was then obtained by protection of the 3-hydroxyl of **13** as the levulinate ester **23** followed by deallylation using Pd(PPh_3_)_4_ and 1,3-dimethylbarbituric acid (DMBA).^24^ This deallylation method offers milder reaction conditions and shorter reaction times than other Pd-catalyzed methods. The synthesis of disaccharide **16** started with allyl deprotection of **13** to give diol **24**, which was selectively protected at the 5-*O*-position of ribitol as a dimethoxytrityl (DMTr) ether leading to intermediate **15**, which was in turn converted into H-phosphonate **16** by phosphitylation using PCl_3_–imidazole–Et_3_N.^17^

### Synthesis of key disaccharides **10**, **11**, and **12**

**Scheme 2:**
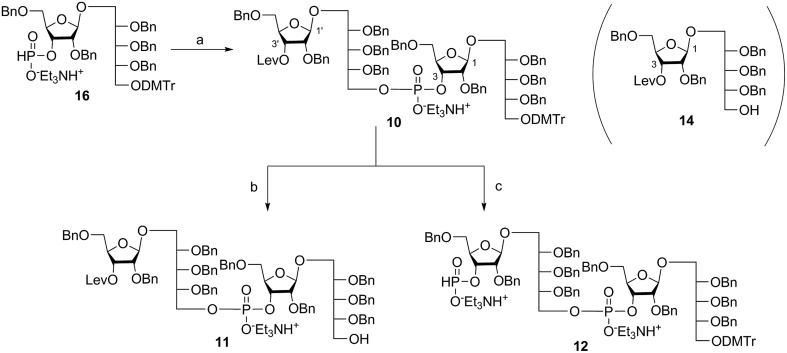
Synthesis of dimeric PRP fragments **10**, **11**, and **12**. Reagents and conditions: (a) **14**, PivCl, pyridine, 0 °C, then I_2_, pyridine/H_2_O, RT, 85%; (b) TCA, DCM, RT, 90%; (c) (i) hydrazine acetate, DCM, RT, 90%; (ii) PCl_3_, Et_3_N, imidazole, DCM, 0 °C. PivCl = pivaloyl chloride, TCA = trichloroacetic acid.

Coupling disaccharides **14** and **16** (Scheme 2) using pivaloyl chloride (PivCl) as activator, followed by *in situ* oxidation with iodine in pyridine/water, generated dimeric PRP fragment **10** in 85% yield. The newly formed phosphodiester bond was confirmed by ^31^P NMR (*δ* – 1.52 ppm). Fragment **10** served as central intermediate as it was converted into **11** by selective removal of the DMT group using trichloroacetic acid (TCA) in DCM, and, into **12** by removal of the levulinate ester using hydrazine-acetate followed by H-phosphonate formation. These dimeric PRP units became the crucial intermediates for the synthesis of tetrameric, hexameric, octameric, and decameric sPRP oligosaccharides.

### Synthesis of the tetrameric PRP oligosaccharide **1**

**Scheme 3:**
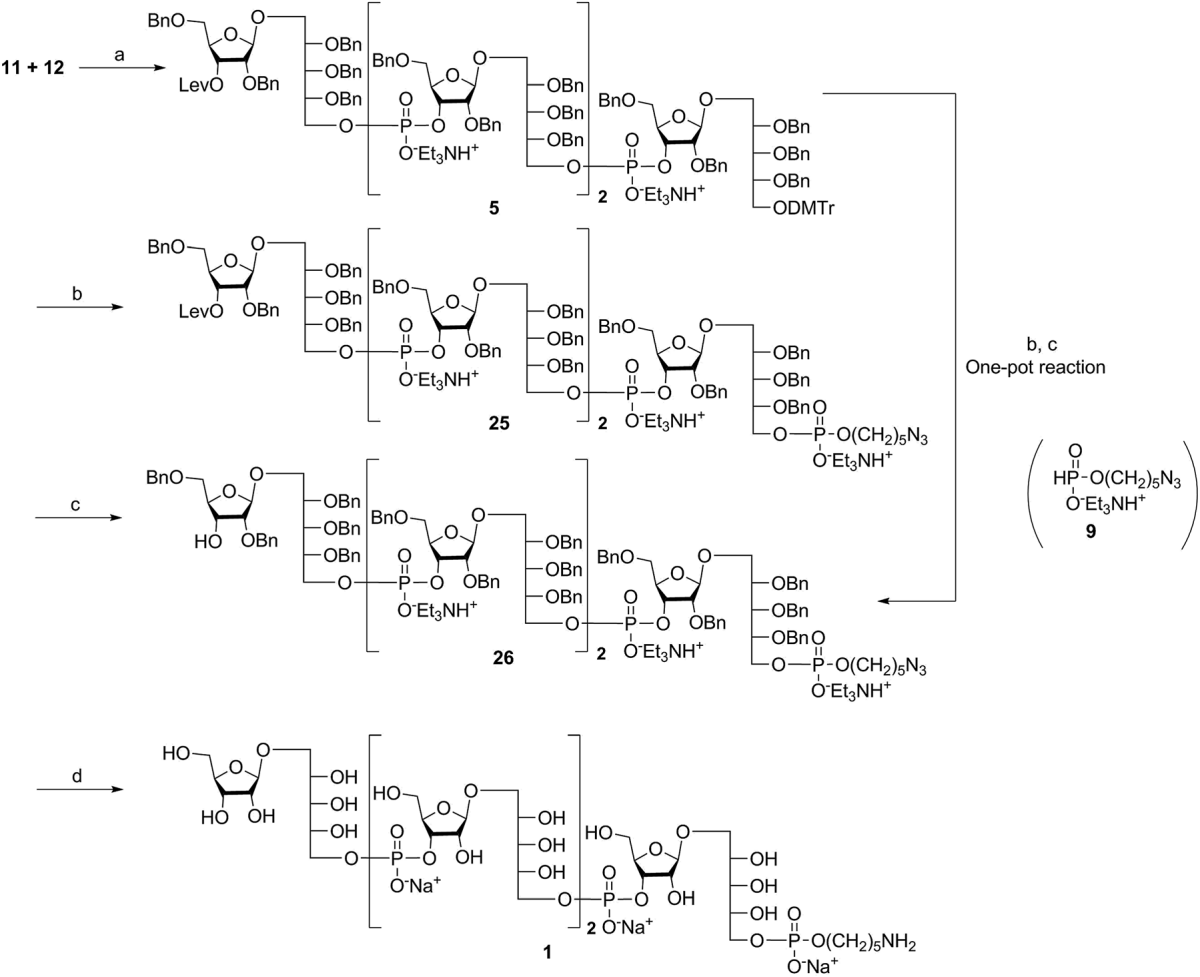
Synthesis of the tetrameric PRP fragment **1** containing the aminopentyl linker. Reagents and conditions: (a) PivCl, pyridine, 0 °C, then I_2_, pyridine/H_2_O, RT, 85%; (b) (i) TCA, DCM, RT; (ii) 9, PivCl, pyridine, 0 °C, then I_2_, pyridine/H_2_O, RT, 80% (over two steps); (c) hydrazine acetate, DCM, RT, 90% or 70% (one pot; three steps); (d) Pd/C, H_2_, EtOAc/MeOH/50% AcOH(aq.), 89%. PivCl = pivaloyl chloride, TCA = trichloroacetic acid.

The synthesis of tetrameric PRP fragment **1** was accomplished using a (2+2) block approach involving coupling of dimer **11** and H-phosphonate **12** using PivCl as activator.^7^–^10^,^12^,^13^ To suppress several competing undesired side reactions such as *O*-acylation^17^ and *P*-acylation,^25^ a diluted solution of PivCl was added slowly to the reaction mixture of **11** and **12**. Oxidation of the H-phosphonate intermediate of the tetrameric PRP fragment was accomplished using reported conditions^17^ and provided compound **5** in 85% yield as the triethylammonium salt (Scheme 3). The presence of the phosphodiester bridge in **5** was confirmed by ^31^P NMR, which revealed three resonances at *δ* 0.73, –0.05, and –0.47 ppm. Trityl cleavage from **5** followed by coupling with H-phosphonate linker **9** and subsequent oxidation gave the PRP intermediate **25**. Delevulination led to **26** that was subjected to hydrogenolysis using a mixture of EtOAc/MeOH/50% AcOH(aq.) resulting in tetrameric PRP **1** as the sodium salt after purification (Scheme 3).

**Fig. 3:**
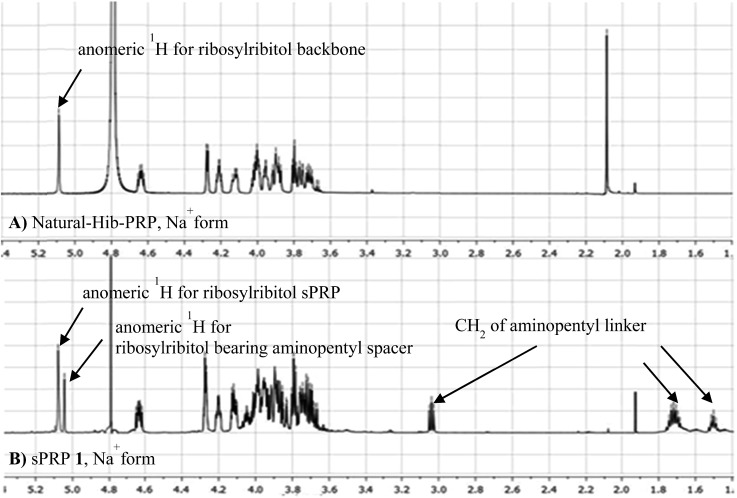
Comparison between (A) ^1^H NMR of natural Hib-PRP, Na^+^ form^26^ and (B) sPRP **1**, Na^+^ form.

The ^1^H NMR spectrum of the sodium form of sPRP **1** and that of the isolated Hib-PRP WHO standard are in good agreement with one another (Fig. 3).^26^ The spectra are similar for the backbone structure of ribose and ribitol and only display additional resonances in the case of sPRP because of the presence of the C5 alkyl linker. Furthermore, the spectrum for sPRP **1** we recorded (Fig. 3b) and the spectrum previously reported in the literature were found to be similar.^9^

**Scheme 4:**
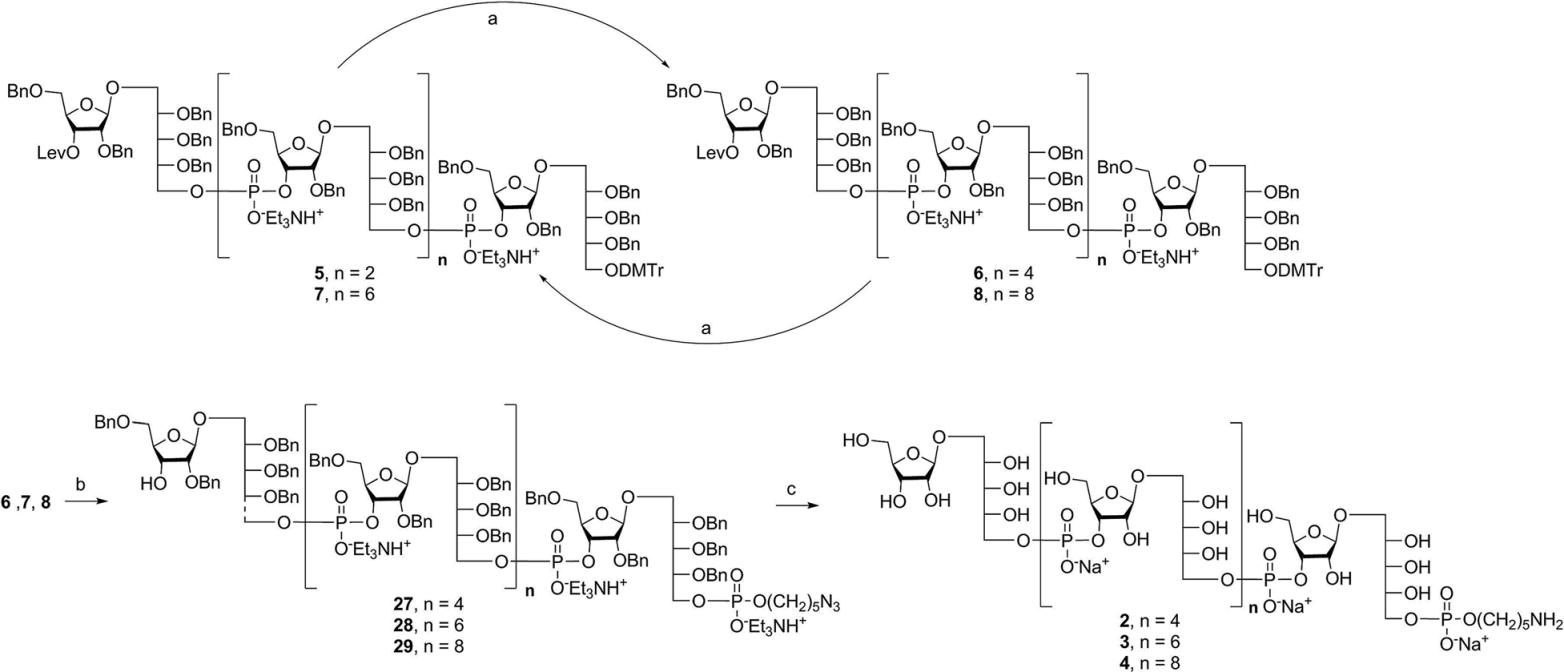
Synthesis of elongated sPRP oligosaccharides **2**, **3**, and **4**. Reagents and conditions: (a) (i) TCA, DCM, RT; (ii) **12**, PivCl, pyridine, 0 °C, then I_2_, pyridine/H_2_O, RT, 85% for **6**, 83% for **7**, 80% for **8** (over two steps); (b) (i) TCA, DCM, RT; (ii) **9**, PivCl, pyridine, 0 °C, then I_2_, pyridine/H_2_O, RT; (iii) hydrazine acetate, DCM, RT, 74% for **27**, 72% for **28**, 72% for **29** (over three steps); (c) Pd/C, H_2_, EtOAc/MeOH/50% AcOH(aq.), 87% for **2**, 80% for **3**, 79% for **4**. PivCl = pivaloyl chloride, TCA = trichloroacetic acid.

Alternatively, in order to improve PRP fragment elongation and reduce the number of purification steps, we carried out the three sequential reactions as a “one-pot” process of detritylation, coupling/oxidation, and delevulinilation to afford compound **26** in 70% yield over three steps (Scheme 3). This modified process was applied to the synthesis of Hib fragments **6–8** (Scheme 4).

### Synthesis of the hexameric, octameric, and decameric sPRP oligosaccharides **2**, **3**, and **4**

Starting from tetrameric sPRP **5** and the iterative one-pot approach using dimeric intermediate **12** and H-phosphonate aminopentyl linker **9**, the hexameric, octameric and decameric sPRP intermediates **27**, **28** and **29**, respectively, were obtained using the methods described above (Scheme 4). Finally, global deprotection of sPRP fragments **27**, **28**, and **29** under hydrogenolysis conditions using palladium and H_2_ (50 psi) in a mixture of EtOAc/MeOH/50% AcOH(aq.), followed by gel filtration over Sephadex LH-20 and cation-exchange (Dowex 50WX4, Na^+^ form), gave the sPRP oligosaccharides **2**, **3**, and **4** in 87%, 80%, and 79% yields, respectively (Scheme 4). The structural details were confirmed by NMR (ESI†).

### Microarray analysis of PRP-directed polyclonal antibodies

**Fig. 4:**
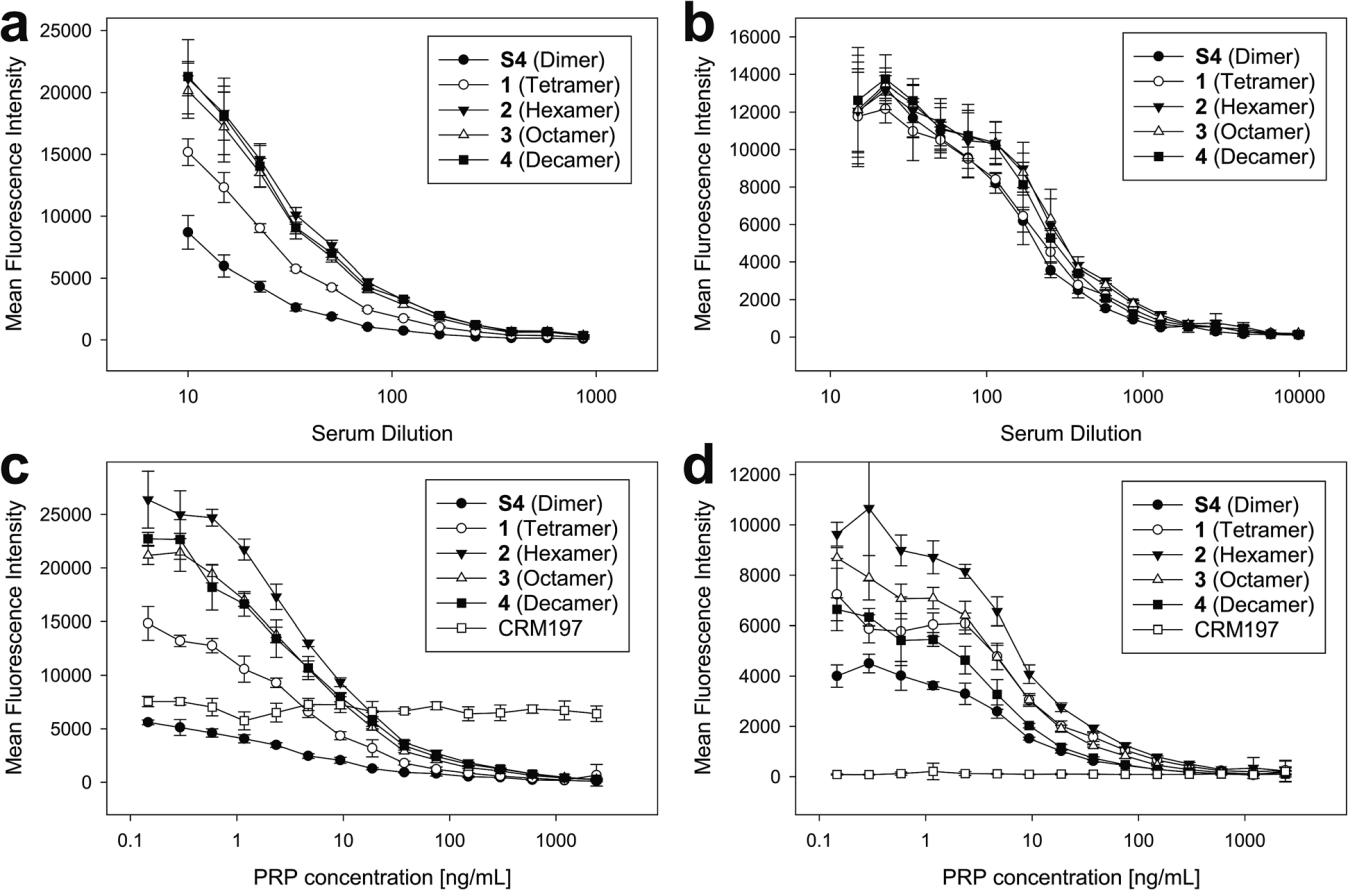
Glycan array binding analysis of anti-PRP antibodies to sPRP oligosaccharides **1–4**. (a and b) Microarray slides printed with sPRP were incubated with a dilution series of a human reference serum (a) and rabbit anti-Hib antiserum (b) followed by a fluorescent secondary antibody, and fluorescence intensities for the synthetic length-defined glycans were determined. (c and d) To verify specificity of the analyzed antibodies, antibody binding was inhibited competitively by adding different concentrations of native PRP to human reference serum diluted 1 : 200 (c) or rabbit typing serum diluted 1 : 1000 (d). All values are the mean of eight spots from two replicate wells with error bars denoting the standard deviation (SD).

To ensure that the size-defined sPRP oligosaccharides are recognized by antibodies raised against natural Hib PRP, glycan array analyses were performed.^27^ The sPRP oligosaccharides containing the primary aminopentyl linker were immobilized on *N*-hydroxysuccinimide (NHS)-hydrogel glass slides (Fig. S1†) and the surface was subsequently incubated with one of two sera that contain Hib PRP-specific antibodies (Fig. 4a and b). Concentration-dependent binding to all sPRP oligosaccharides was observed for pooled human sera used to calibrate Hib titer analyses as well as for a rabbit antiserum used for bacterial serotyping assays. Antibodies from the human serum also recognized the carrier protein CRM197 that was printed alongside the oligosaccharides, probably due the fact that diphtheria toxoid and CRM197 are routinely used as a vaccine antigen against diphtheria and as a carrier protein in approved glycoconjugate vaccines, respectively.^21^,^28^

As the glycan arrays were probed with sera and not purified antibodies, an inhibition assay with the native Hib PRP was used to validate that antibodies binding to the sPRP oligosaccharides were indeed directed towards native Hib PRP. Although this specificity confirmation might not be strictly necessary for the rabbit hyperimmune serum as it is obtained under well-defined experimental conditions, it is known that humans develop antibodies against many glycan antigens over their lifetimes^29^ that might cross-react with the sPRP oligosaccharides by chance. Therefore, different concentrations of WHO PRP standard were added to dilutions of both sera to capture Hib PRP-specific antibodies, thereby preventing them from binding to the sPRP oligosaccharides on the microarray surface and leading to signal suppression as a result of cross-reactivity. For both sera, concentration dependent signal suppression was seen that was complete at the highest employed PRP concentrations (Fig. 4c and d). All antibodies that recognized the sPRP oligosaccharides were directed against Hib PRP. Failure of the human serum to suppress binding to the CRM197 spots confirmed specificity of the inhibition assay.

While the cross-reactivity between the sPRP oligosaccharides and anti-PRP antibodies was readily established, determining the size of epitopes bound by the anti-PRP antibodies in the two sera, was not possible. For the human serum, it was observed that the binding signals for dimer **S4** were reduced compared to the larger oligosaccharides (Fig. 4a, see ESI† for chemical structure), in agreement with an earlier immunization study that described comparatively poor reactivity of the dimer in rabbits: a dimer–TT conjugate induced significantly lower anti-PRP antibody levels than a trimer–TT conjugate.^20^ Binding signals to tetramer **1** were lower as well, albeit less pronounced. However, comparison of fluorescence intensities on glycan arrays is an imperfect measure of antibody binding strength since identical, repetitive epitopes result potentially in differing epitope densities. For example, if the same number of decamers and dimers were immobilized, the number of repeating units (potential binding sites) would be fivefold higher for the decamer. However, it must also be considered that sterics or a high formal negative charge may impede immobilization of similar numbers of the larger sPRP oligosaccharides. Similar binding levels observed for all sPRP oligosaccharides to the rabbit serum at low dilutions (Fig. 4b) are not necessarily an indication of similar epitope densities, as different antibodies react differently to changes in epitope density on glycan arrays^30^ and crowding effects at high antibody concentrations might outweigh the advantages of having high epitope densities. There are more pronounced differences in fluorescence intensity at higher dilutions for the rabbit typing serum, as can clearly be seen in the lowest PRP concentration point in Fig. 4d.

### Conjugation of CRM197 to sPRP oligosaccharides 1–4

**Scheme 5:**
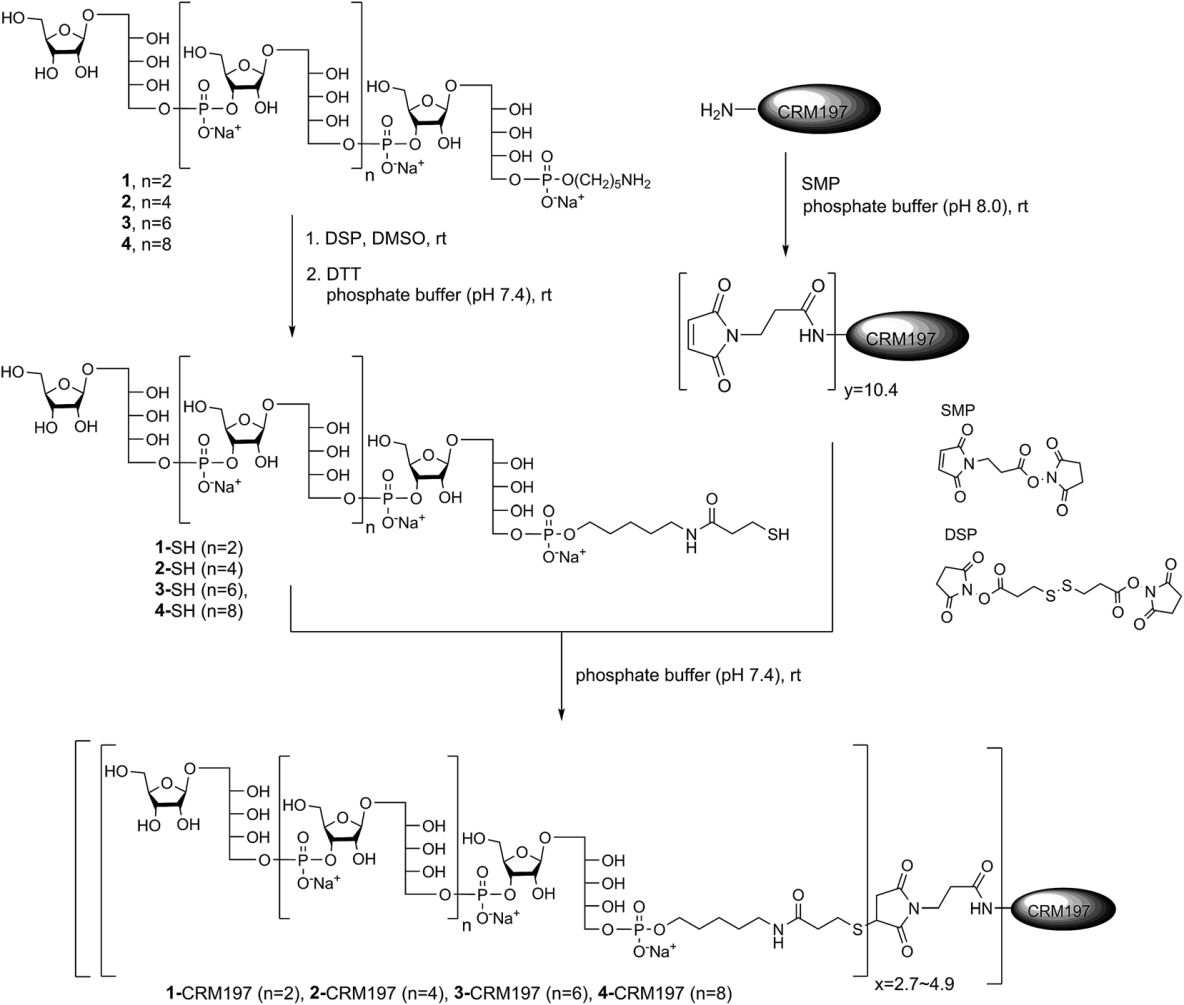
Conjugation of sPRP oligosaccharides **1–4** to CRM197 *via* the corresponding thiols. *y* represents the stoichiometry of maleimide loading, and *x* represents the stoichiometry of maleimide modification by the respective thiol: 4.9 (for **1**), 4.0 (for **2**), 3.1 (for **3**), and 2.7 (for **4**).

After establishing the immunological cross-reactivity of the sPRP constructs with PRP-directed antibodies, immunization studies with sPRP-CRM197 glycoconjugates were initiated. Mindful of the fact that proper conjugation chemistry is key to efficient glycoconjugate production,^31^ and the importance of choosing a linker that induces minimal undesired immunogenic responses,^32^ the well-established thiol–maleimide coupling method was chosen^17^,^24^,^33^,^34^ The sPRP oligosaccharides were conjugated to CRM197, a carrier protein that has been successfully used in commercial vaccines and immunological studies in conjunction with a variety of antigens.^17^,^24^,^33^,^34^ The amine group of the linker of sPRP oligosaccharide **1** was reacted with commercially available dithiobis(succinimidyl propionate) (DSP) in phosphate buffer (pH 7.4) at room temperature (Scheme 5). The disulfide bond was then cleaved using dithiothreitol (DTT) at room temperature to obtain the thiol products. Cleavage was confirmed by ^1^H NMR and MALDI-TOF MS (ESI†). The same procedure was applied to sPRP oligosaccharides **2–4**. To obtain maleimide-containing CRM197, the protein was incubated with *N*-succinimidyl 3-maleimidopropionate (SMP) in phosphate buffer, pH 7.4, at room temperature for 2 h (Scheme 5). The average stoichiometry of maleimide linkers covalently attached to the protein was determined by MALDI-TOF MS to be 10.4 (ESI†).

The well-defined thiol modified sPRP oligosaccharides were conjugated to the maleimide-containing CRM197 in phosphate buffer (pH 7.4) at room temperature (Scheme 5) to afford glycoconjugates **1**-CRM197, **2**-CRM197, **3**-CRM197 and **4**-CRM197. The number of sPRP oligosaccharides conjugated to CRM197 in each case was calculated from the mass shift measured using MALDI-TOF MS (ESI Table S1†). Further characterization of the glycoconjugates was performed using SDS–polyacrylamide gel electrophoresis (SDS–PAGE) confirming an increase in molecular weight; Western blot with polyclonal anti-diphtheria toxin reactive to CRM197 as positive control and anti-Hib antibodies showed specific attachment of Hib-reactive epitopes for all conjugates. PRP content determination based on high performance anion exchange chromatography with pulsed amperometric detection after alkaline hydrolysis (HPAEC-PAD) was used to confirm the loading from MALDI (Fig. S2†).^35^ The HPAEC-PAD data was in good agreement with MALDI-MS for conjugates **1**-CRM197 through **3**-CRM197; however, the observed saccharide concentration for **4**-CRM197 was significantly lower than expected based on the MS data (ESI Fig. S3 and Table S1†).

### Immunogenicity studies in rabbits

**Fig. 5:**
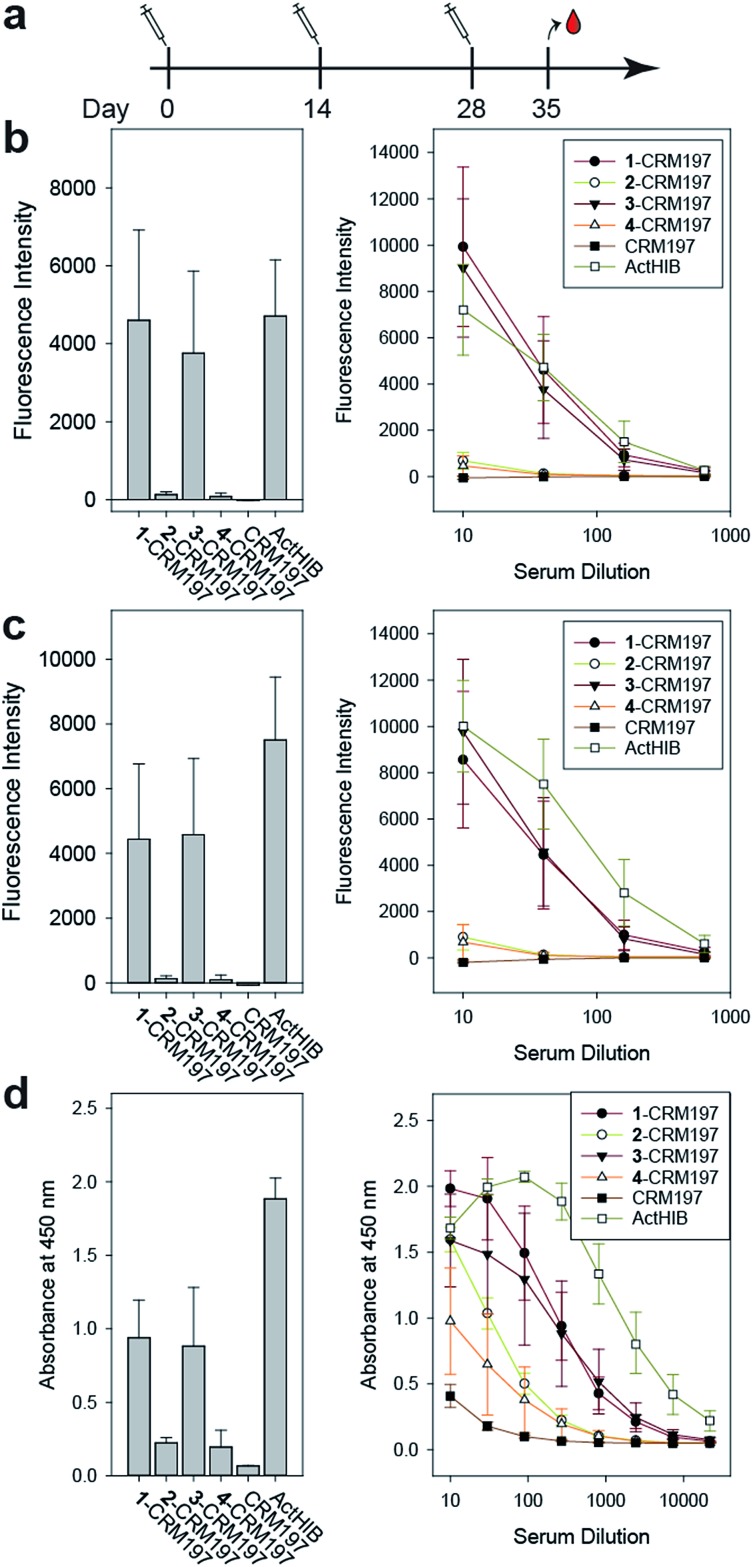
Immunization of rabbits with sPRP glycoconjugates, CRM197 negative control and ActHIB positive control. (a) Prime-boost immunization regime. Each dose contained 5 μg PRP. (b) IgG response to Hib RU dimer **S4** as determined by glycan array. Bar chart (left): response measured with serum diluted 1 : 40. Line plot (right): dilution-response series. (c) IgG response towards Hib RU decamer **4** as determined by glycan array. Charts as in (b). See ESI Fig. S4a–c† for antibody response toward **1–3** as determined by glycan array. (d) IgG response towards native Hib PRP measured by ELISA. Bar chart (left): response measured with serum diluted 1 : 270. Line plot (right): dilution–response series (b–d) each data point represents the mean of four animals with error bars representing the standard error of the mean (SEM).

The sPRP-CRM197 glycoconjugates were used to immunize Zika rabbits, the animal model of choice in earlier immunization studies involving sPRP oligosaccharides.^16^,^20^ Six groups with four rabbits per group were immunized in a prime-boost regime with unadjuvanted glycoconjugate containing 5 μg sPRP per immunization (Fig. 5a). The negative control group received CRM197, and the positive control group the approved vaccine ActHIB (5 μg PRP, corresponding to half the human dose), a conjugate of native PRP to TT.^36^

Serum IgG levels on day 35, one week after the second boost, towards the sPRP oligosaccharides were determined by glycan array analysis (Fig. 5 and S4†). As expected, no binding to the oligosaccharides was observed for the CRM197-immunized negative control group, confirming that the antibody response originates from the oligosaccharide component of the conjugate. The sPRP conjugates showed different levels of immunogenicity without a clear length-dependent trend, as tetramer conjugate **1**-CRM197 and octamer conjugate **3**-CRM197 exhibited the highest immunogenicities. This indicates that an RU tetramer is sufficient for optimal immunogenicity, in agreement with an earlier investigation of an sPRP tetramer-TT conjugate that elicited antibody levels in monkeys similar to those of a CRM197 conjugate of size-reduced PRP with an average of 20 repeating units.^10^ Comparatively lower elicited antibody levels were observed for hexamer conjugate **2**-CRM197 and decamer conjugate **4**-CRM197. In the latter case, the low immunogenicity may be caused by a too low oligosaccharide dose used for immunization (see above). However, IgM levels in rabbits immunized with **4**-CRM197 were more similar to those elicited by **1**-CRM197 and **3**-CRM197 suggesting that the amount of carbohydrate was sufficient for a comparable immune response (ESI Fig. S5†). We are confident based on the characterization data that the hexamer conjugate **2**-CRM197 preparation is of similar quality to the more immunogenic **1**-CRM197 and **3**-CRM197 samples, and that the lower immunogenicity is therefore an intrinsic feature of the RU hexamer. PRP is believed to form highly ordered, rigid secondary structures,^37^ and, depending on the available number of repeating units, oligosaccharides might fold into structures that interact with immune receptors in different ways. These immune receptors not only include B cell receptors and antibodies, but also other cell surface receptors such as lectins whose engagement by oligo- and polysaccharides can intensify or attenuate an immune response.^38^ This more complex *in vivo* setting explains the apparent discrepancy with the glycan array serum analysis (Fig. 4) that showed more uniform results.

Similar to our observations, it was previously shown that penta- and hexamers linked to synthetic T cell epitopes were of lower immunogenicity than a trimer.^20^ This conformational influence may also extend to the decamer (as mentioned above, a comparatively strong IgM response was induced here). It is also possible that the penta- and hexamers were too large for efficient processing of the T cell antigens. Rabbits, used as the animal model in our as well as the previous studies, may contribute to the lower immunogenicity seen for a single construct because they are not inbred and have more variable experimental outcomes than mice.^39^ However, mice are an unreliable animal model for sPRP conjugates.^16^,^20^

For a vaccine, the important measure is not reactivity to the synthetic oligosaccharides but serum reactivity towards the capsule which is often measured by IgG towards the natural PRP.^16^,^17^,^20^ The differences in immunogenicity seen in the response to the sPRP oligosaccharides on glycan arrays, namely high IgG levels in the cases of **1**-CRM197 and **3**-CRM197 and low in the cases of **2**-CRM197 and **4**-CRM197, were clearly mirrored when an enzyme-linked immunosorbent assay (ELISA) was used to determine the IgG binding levels towards native PRP (Fig. 5d). The responses are similar between **1**-CRM197 and **3**-CRM197, suggesting that RU tetramer **1** is not only as immunogenic as the larger oligosaccharides but also is able to induce the production of antibodies with a similar degree of cross-reactivity.

Immunogenicity of the positive control ActHIB is higher than that of the sPRP conjugates and may be attributed to the different carrier protein, as higher titers have previously been obtained in rabbits for sPRPs conjugated to TT compared to CRM197.^16^ Likely, this is a rabbit specific effect as the carrier proteins do not differ significantly in inducing immune responses in human Hib vaccines based on isolated PRP.^40^ However, possible differences between carrier proteins in humans have not yet been evaluated for sPRPs.

## Conclusions

Quimi-Hib, the only marketed sPRP glycoconjugate vaccine, contains a mixture of different length oligosaccharides.^17^ With the goal of understanding the effect of glycotope length on vaccine design, access to well-defined Hib oligosaccharides is crucial for providing the necessary tools for in-depth immunological evaluations. We developed a strategy for the [2+2], [4+2], [6+2], and [8+2] syntheses of sPRP oligosaccharides using orthogonal protecting groups. The synthetic route described here improved upon the existing methods for preparing the key ribitol and ribose-ribitol intermediates, and a one-pot coupling and cleavage process significantly simplified the purification process and improved yields. The sPRP oligosaccharides are similar to natural Hib PRP, as is evident from the NMR and biological data. Glycan array analyses revealed that sPRP oligosaccharides present cross-reactive epitopes to antibodies raised against isolated PRP. Glycoconjugates of sPRP oligosaccharides are immunogenic in a rabbit model, whereby tetrameric sPRP **1** is an excellent starting point for the design of a defined semi-synthetic glycoconjugate Hib vaccine.

## Conflicts of interest

There are no conflicts to declare.

## Supplementary Material

Supplementary information

## References

[cit1] Gilsdorf J. R. (2015). J. Infect..

[cit2] Ulanova M., Tsang R. S. W. (2014). Lancet Infect. Dis..

[cit3] Zarei A. E., Almehdar H. A., Redwan E. M. (2016). J. Immunol. Res..

[cit4] Goldblatt D. (2000). Clin. Exp. Immunol..

[cit5] Berti F., Adamo R. (2013). ACS Chem. Biol..

[cit6] Agrawal A., Murphy T. F. (2011). J. Clin. Microbiol..

[cit7] Hoogerhout P., Evenberg D., van Boeckel C., Poolman J. T., Beuvery E. C., van der Marel G. A., van Boom J. H. (1987). Tetrahedron Lett..

[cit8] Hermans J. P. G., Poot L., van der Marel G. A., Hoogerhout P., Kloosterman M., van Boom J. H., van Boeckel C. A. A., Evenberg D., Poolman J. T. (1987). Recl. Trav. Chim. Pays-Bas.

[cit9] Hoogerhout P., Funke C. W., Mellema J.-R., Wagenaars G. N., van Boeckel C. A. A., Evenberg D., Poolman J. T., Lefeber A. W. M., van der Marel G. A., Van Boom J. H. (1988). J. Carbohydr. Chem..

[cit10] Peeters C. C., Evenberg D., Hoogerhout P., Käyhty H., Saarinen L., van Boeckel C. A., van der Marel G. A., van Boom J. H., Poolman J. T. (1992). Infect. Immun..

[cit11] Seeberger P. H., Werz D. B. (2007). Nature.

[cit12] Yuan Wang Z., Just G. (1988). Tetrahedron Lett..

[cit13] Laval C., Just G. (1990). Tetrahedron.

[cit14] Elie C. J. J., Muntendam H. J., van den Elst H., van der Marel G. A., van Boom J. H., Hoogerhout P. (1989). Recl. Trav. Chim. Pays-Bas.

[cit15] Nilsson S., Bengtsson M., Norberg T. (1992). J. Carbohydr. Chem..

[cit16] Fernandez-Santana V., Cardoso F., Rodriguez A., Carmenate T., Pena L., Valdes Y., Hardy E., Mawas F., Heynngnezz L., Rodriguez M. C., Figueroa I., Chang J., Toledo M. E., Musacchio A., Hernandez I., Izquierdo M., Cosme K., Roy R., Verez-Bencomo V. (2004). Infect. Immun..

[cit17] Verez-Bencomo V., Fernández-Santana V., Hardy E., Toledo M. E., Rodríguez M. C., Heynngnezz L., Rodriguez A., Baly A., Herrera L., Izquierdo M., Villar A., Valdés Y., Cosme K., Deler M. L., Montane M., Garcia E., Ramos A., Aguilar A., Medina E., Toraño G., Sosa I., Hernandez I., Martínez R., Muzachio A., Carmenates A., Costa L., Cardoso F., Campa C., Diaz M., Roy R. (2004). Science.

[cit18] Morelli L., Poletti L., Lay L. (2011). Eur. J. Org. Chem..

[cit19] Rana R., Dalal J., Singh D., Kumar N., Hanif S., Joshi N., Chhikara M. K. (2015). Vaccine.

[cit20] Chong P., Chan N., Kandil A., Tripet B., James O., Yang Y. P., Shi S. P., Klein M. (1997). Infect. Immun..

[cit21] Astronomo R. D., Burton D. R. (2010). Nat. Rev. Drug Discovery.

[cit22] Uchida T., Pappenheimer A. M., Greany R. (1973). J. Biol. Chem..

[cit23] Chiu-Machado I., Castro-Palomino J. C., Madrazo-Alonso O., Lopetegui-Palacios C., Verez-Bencomo V. (1995). J. Carbohydr. Chem..

[cit24] Wang C.-H., Li S.-T., Lin T.-L., Cheng Y.-Y., Sun T.-H., Wang J.-T., Cheng T.-J. R., Mong K. K. T., Wong C.-H., Wu C.-Y. (2013). Angew. Chem., Int. Ed..

[cit25] Froehler B. C., Matteucci M. D. (1986). Tetrahedron Lett..

[cit26] Mawas F., Bolgiano B., Rigsby P., Crane D., Belgrave D., Corbel M. J. (2007). Biologicals.

[cit27] Geissner A., Seeberger P. H. (2016). Annu. Rev. Anal. Chem..

[cit28] Pobre K., Tashani M., Ridda I., Rashid H., Wong M., Booy R. (2014). Vaccine.

[cit29] Oyelaran O., McShane L. M., Dodd L., Gildersleeve J. C. (2009). J. Proteome Res..

[cit30] Oyelaran O., Li Q., Farnsworth D., Gildersleeve J. C. (2009). J. Proteome Res..

[cit31] Adamo R., Nilo A., Castagner B., Boutureira O., Berti F., Bernardes G. J. L. (2013). Chem. Sci..

[cit32] Ni J., Song H., Wang Y., Stamatos N. M., Wang L.-X. (2006). Bioconjugate Chem..

[cit33] van der Put R. M. F., Kim T. H., Guerreiro C., Thouron F., Hoogerhout P., Sansonetti P. J., Westdijk J., Stork M., Phalipon A., Mulard L. A. (2016). Bioconjugate Chem..

[cit34] Boeckler C., Frisch B., Muller S., Schuber F. (1996). J. Immunol. Methods.

[cit35] de Haan A., van der Put R. M. F., Beurret M. (2013). Biomed. Chromatogr..

[cit36] Kniskern P. J., Marburg S. and Ellis R. W., in Vaccine Design, ed. R. T. Borchardt, M. F. Powell and M. J. Newman, Springer US, Boston, MA, 1995, vol. 6, pp. 673–694.

[cit37] Hennessey J. P., Bednar B., Manam V. (1993). J. Liq. Chromatogr..

[cit38] Sancho D., Reis e Sousa C. (2012). Annu. Rev. Immunol..

[cit39] Festing M. F. W. (2014). ILAR J..

[cit40] Knuf M., Kowalzik F., Kieninger D. (2011). Vaccine.

