# Supplementary material for: A modular synthetic route to size-defined immunogenic Haemophilus influenzae b antigens is key to the identification of an octasaccharide lead vaccine candidate
Source: Chem Sci. 2017 Dec 11;9(5):1279–88. doi: 10.1039/c7sc04521b (PMC5887106; doi:10.1039/c7sc04521b)
Supplement: Supplementary file 1 [file SC-009-C7SC04521B-s001.pdf]

Electronic Supplementary Material (ESI) for Chemical Science.
This journal is © The Royal Society of Chemistry 2017

Supplementary Information

A modular synthetic route to size-defined immunogenic *Haemophilus influenzae* b antigens is key to the identification of an octasaccharide lead vaccine candidate

Ju Yuel Baek,[1] Andreas Geissner[1,2], Dominea Rathwell [1,2] David Meierhofer[3], Claney L. Pereira, [1,4,*] and Peter H. Seeberger[1,2,*]

[1]Max Planck Institute of Colloids and Interfaces, 14476 Potsdam, Germany

[2]Freie Universität Berlin, Department of Chemistry and Biochemistry, 14195 Berlin, Germany

[3]Max-Planck Institute for Molecular Genetics (MPIMG), 14195 Berlin, Germany

[4]Present address: Vaxxilon Deutschland GmbH, 12489 Berlin, Germany

[*]Correspondence: Peter.Seeberger@mpikg.mpg.de (P.H.S.), claney.pereira@vaxxilon.com (C.L.P)

<u>Table of Contents</u>

Supplementary Figures S1-S5 and Table S1 S3

General Synthetic Procedures S9

Experimental details and characterization data for known compounds S9

Experimental details and characterization data for new compounds S16

General procedure for elongation reaction
with the H-phosphonate intermediate **12** to produce sPRPs **6-8** S22

General procedure for coupling reaction
of **9** with sPRPs **6-8** to give sPRP intermediates **27-29** S22

General procedure for hydrogenation
of sPRP intermediates **27-29** to give elongated sPRP fragments **2-4** S23

Experimental details and characterization data for compound **6** S23

Experimental details and characterization data for compound **27** S24

Experimental details and characterization data for compound **2** S24

Experimental details and characterization data for compound **7** S25

Experimental details and characterization data for compound **28** S25

Experimental details and characterization data for compound **3** S26

Experimental details and characterization data for compound **8** S26

Experimental details and characterization data for compound **29** S27

Experimental details and characterization data for compound **4** S27

Compound S4 S28

General Procedure for the Preparation of Oligosaccharide Thiols

1-SH, **2-SH**, **3-SH**, and **4-SH** for Protein Conjugation S28

Spectra S29

Biological Experiments S86

References S90

Supplementary Figures S1-S5 and Table S1

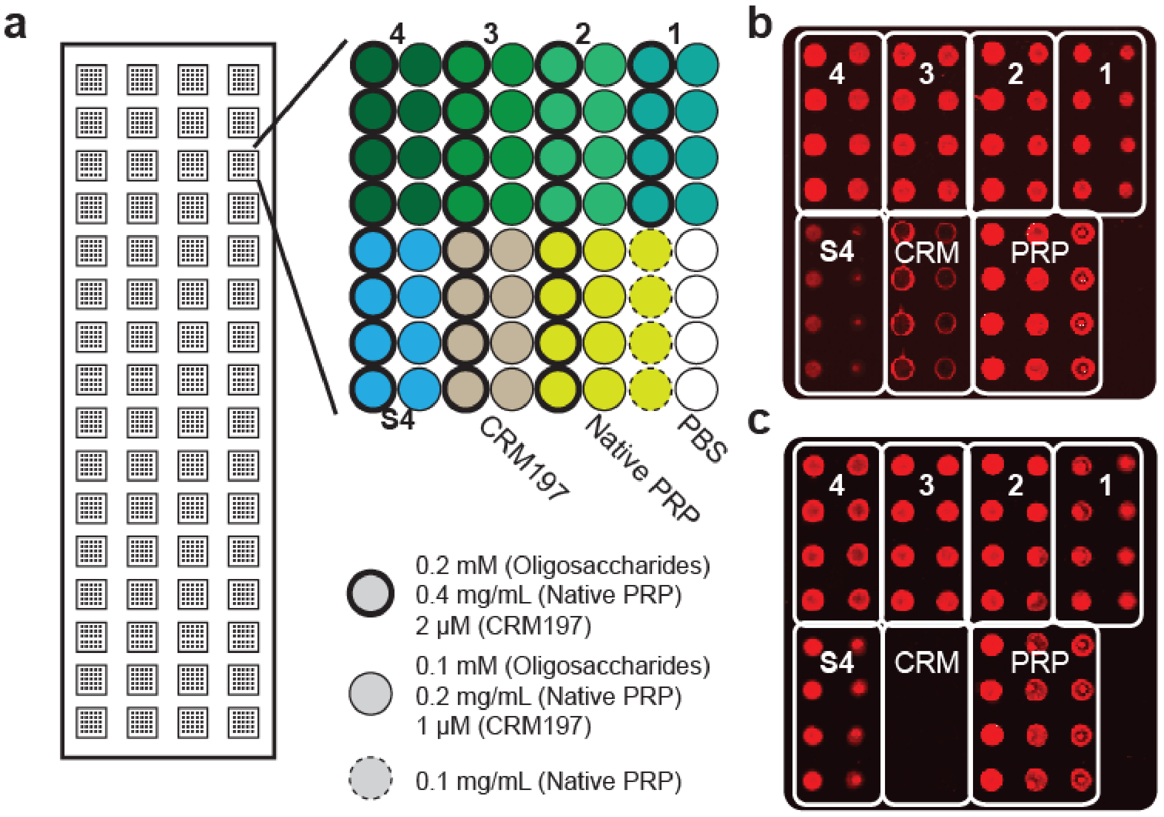

Figure S1: Glycan array printing pattern (a) and example fields of IgG from human Hib reference serum diluted 1:76 (b) and rabbit Hib typing serum diluted 1:171 (c).

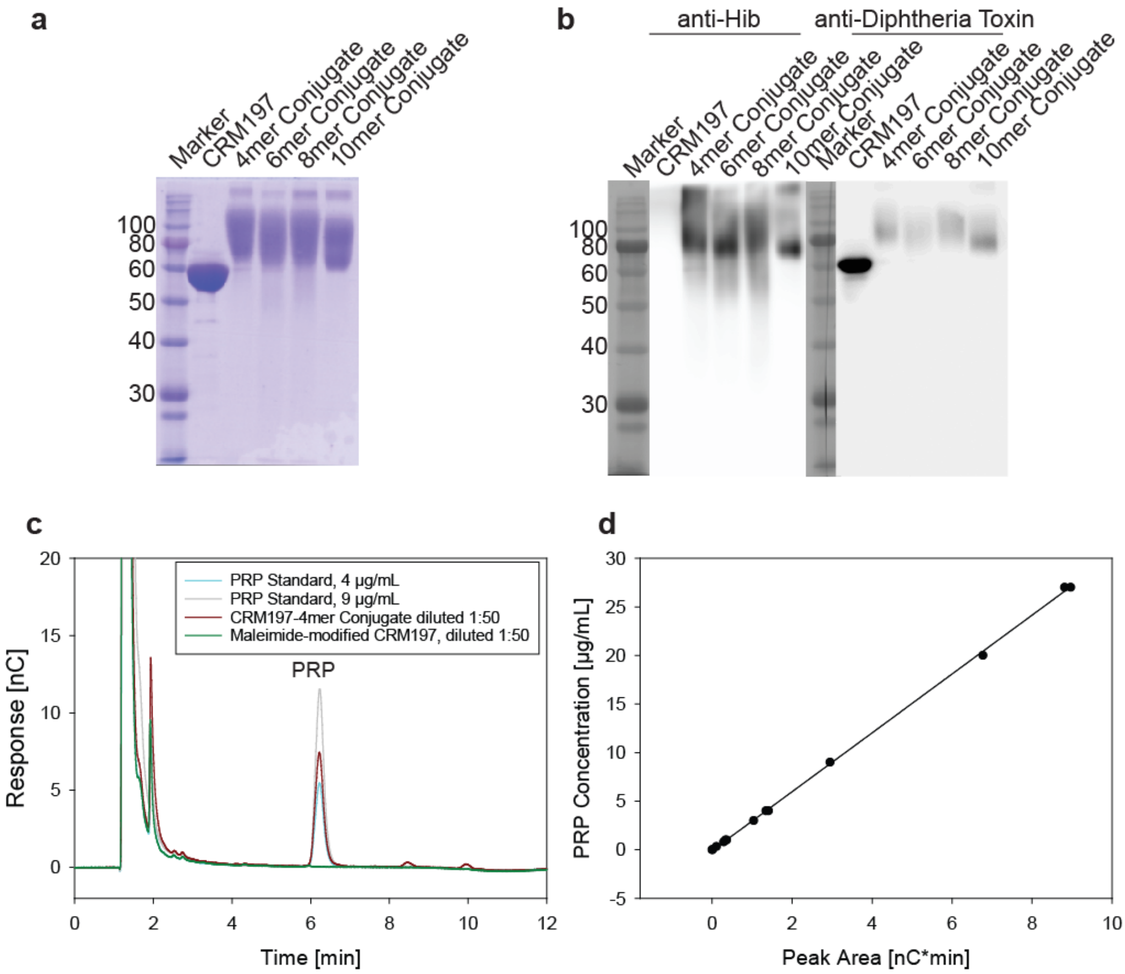

Figure S2: Characterization of glycoconjugates by SDS-PAGE, Western Blot, and HPAEC-PAD. (a) Coomassie-stained SDS gel and (b) Western Blot images. Different exposure times were used for HRP-coupled anti-rabbit and anti-goat antibodies to avoid saturation on the respective membranes. (a) Example HPAEC-PAD chromatogram: WHO PRP standard at 4 µg/mL and 9 µg/mL overlaid with the CRM197 sPRP RU tetramer conjugate **1**-CRM197 and the maleimide-functionalized CRM197 as a negative control (exported from *Chromeleon 7* and drawn with SigmaPlot). (b) Typical calibration to determine PRP concentration of solutions of CRM197 conjugates using different concentrations of PRP standard.

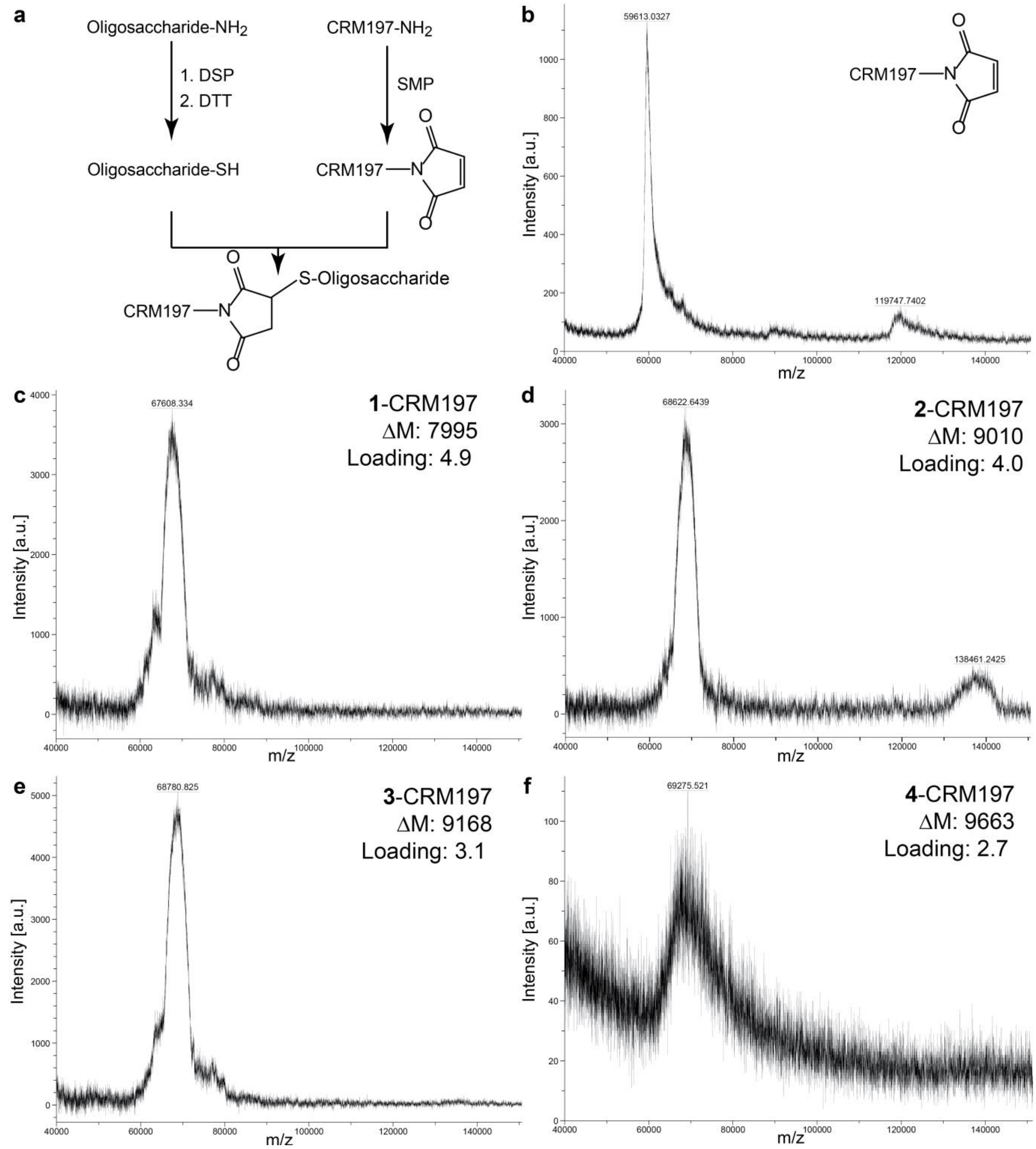

Figure S3: MALDI analysis of glycoconjugates used for immunization. a) Overview of conjugation via thiol-maleimide approach. b) Mass spectrum of maleimide-labeled CRM197; average labeling stoichiometry (59613-58026)/153=10.4. c) Mass spectrum of **1**-CRM197; average labeling stoichiometry (67608-59613)/1635=4.9. d) Mass spectrum of **2**-CRM197; average labeling stoichiometry (68622-59613)/2261=4.0. e) Mass spectrum of **3**-CRM197; average labeling stoichiometry (68780-59613)/2951=3.1. f) Mass spectrum of **4**-CRM197; average labeling stoichiometry (69275-59613)/3641=2.7.

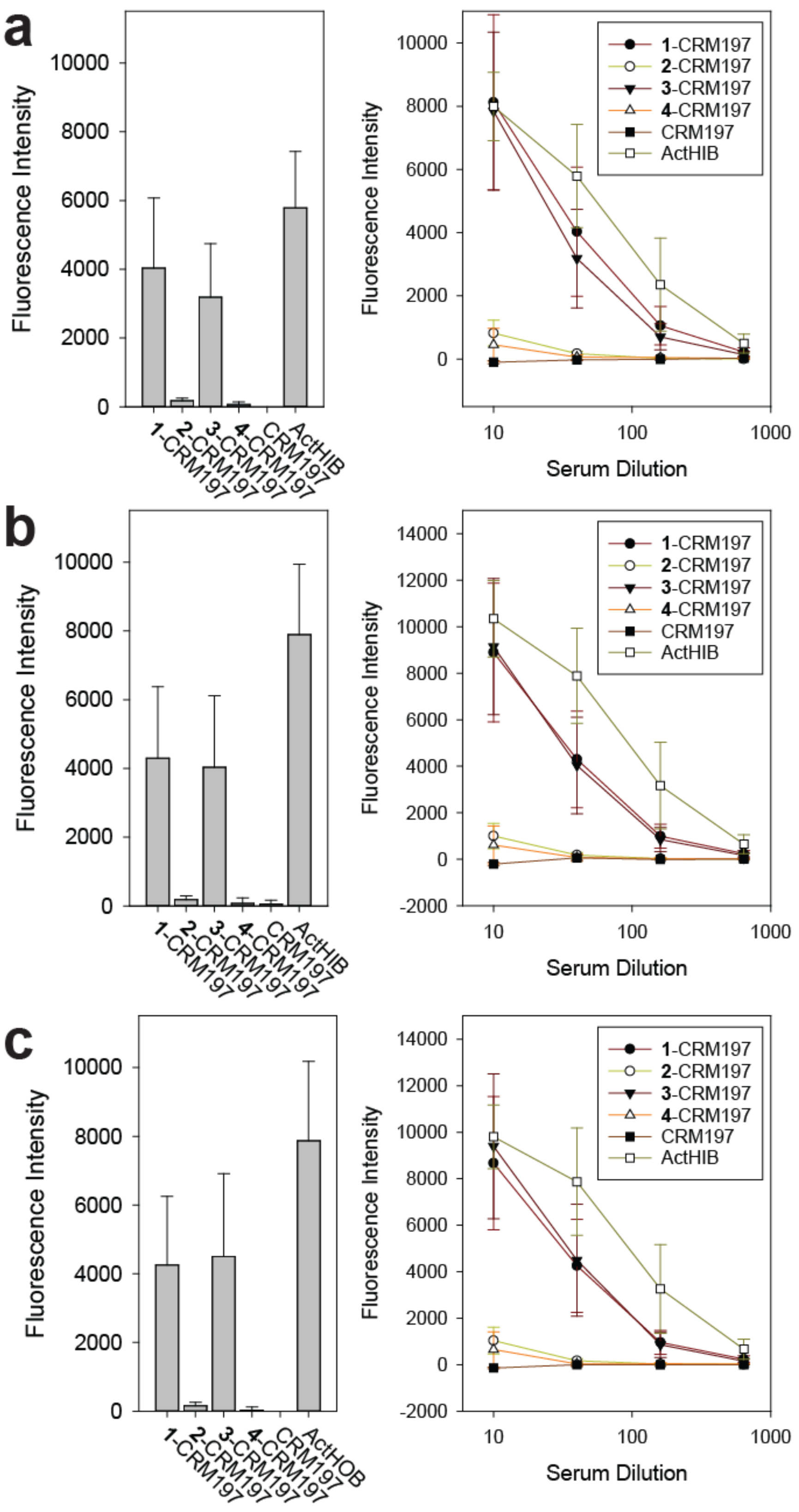

Figure S4: Addendum to main text Figure 6. Glycan array determination of IgG levels towards sPRP oligosaccharides **1** (a), **2** (b), and **3** (c). Each panel: Bar chart (left): response measured with serum diluted 1:40. Line plot (right): dilution-response series.

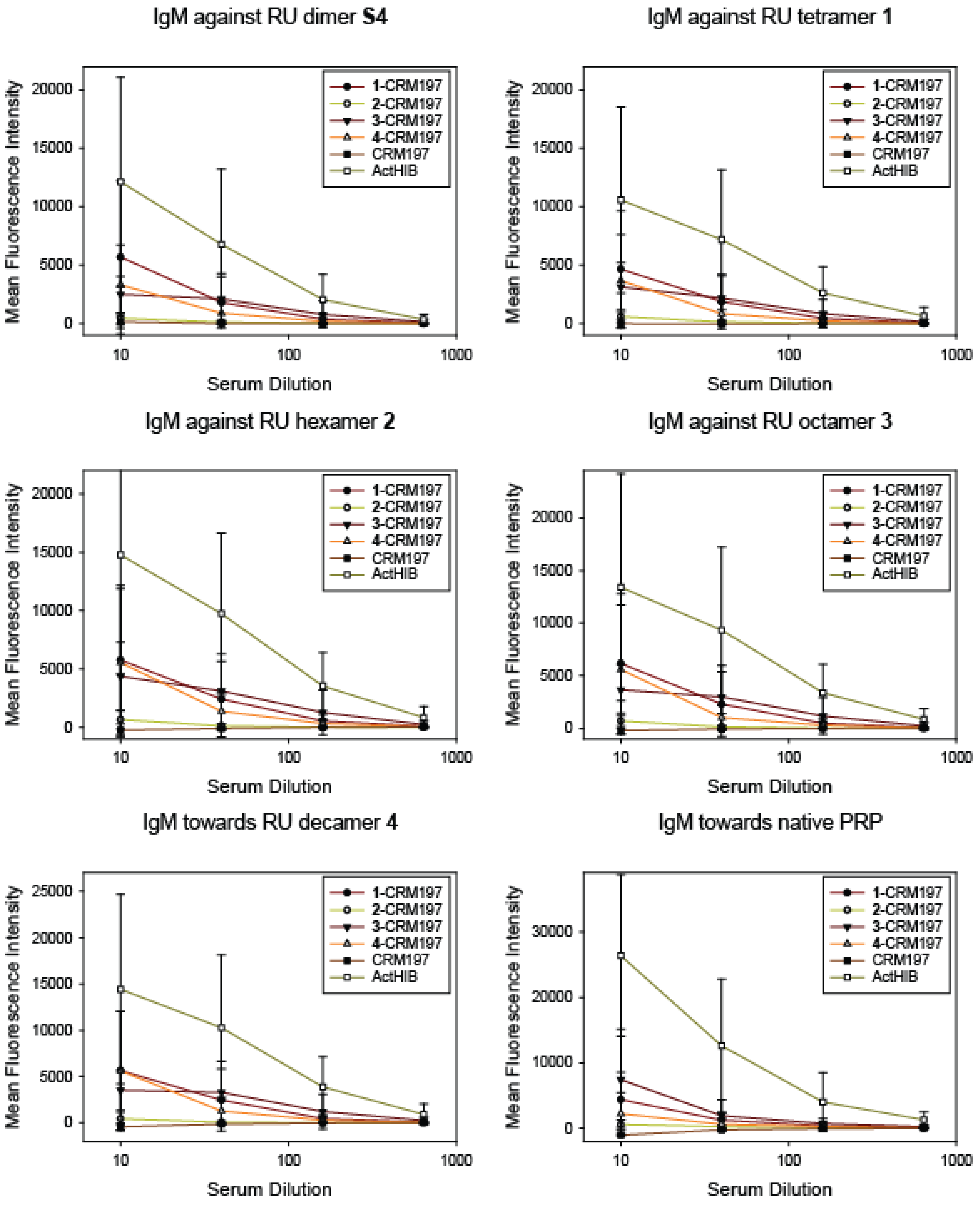

Figure S5: IgM levels towards sPRP oligosaccharides and native PRP in rabbit sera on day 35 (one week post second boost) determined by glcan arrays analysis.

Table S1: Concentrations of oligosaccharides in conjugates used for rabbit immunizations determined by BCA and MALDI-TOF MS or HPAEC-PAD.

Conjugate	c(Protein) [mg/mL] (BCA)	Loading [a]	c(sPRP) [b] (BCA/MS) [mg/mL]	c(sPRP) (HPAEC-PAD) [mg/mL]	
				Run 1	Run 2
**1**-CRM197	3.6	4.9	0.42	0.39	0.41
**2**-CRM197	3.6	4.0	0.51	0.60	0.59
**3**-CRM197	4.2	3.1	0.62	0.52	0.52
**4**-CRM197	2.1	2.7	0.33	0.04	0.05

[a] in mol oligosaccharide per mol protein as determined by MALDI-TOF MS

[b] calculated from loading and protein concentration, value used for immunization dose calculation

General Synthetic Procedures

All reagents were obtained from commercial suppliers and were used as supplied without further purification. Reactions were performed in heat dried glassware under an argon atmosphere using standard Schlenk techniques. Dry solvents were obtained from a Waters Dry Solvent System. Solvents for chromatography were of technical grade and distilled under reduced pressure prior to use. Deionized water was obtained from an in-house purification system. Analytical thin-layer chromatography (TLC) was performed on Kieselgel 60 F254 glass plates precoated with a 0.25 mm thickness of silica gel. Compounds were visualized by UV light at 254 nm or by dipping the plates in a cerium sulfate ammonium molybdate (CAM) solution or sulfuric acid-ethanol solution followed by heating. Flash chromatography was performed on Kieselgel 60 (230-400 mesh). Freeze-drying of aqueous solutions was performed using a Christ Alpha 2-4 LD Lyophilizer. ^{1}H and ^{13}C NMR spectra were obtained on a *Varian* MR-400 (400 MHz) or on a *Varian* Premium COMPACT 600 (600 MHz) and are reported in parts per million (δ) relative to the resonance of the solvent. Coupling constants (*J*) are reported in Hertz (Hz). Optical rotations (OR) were measured with a Schmidt & Haensch UniPol L 1000 at concentrations (c) expressed in g/100 mL. MALDI-TOF spectra were recorded on a Bruker Daltonics Autoflex Speed or Ultraflex II, using 2,4,6-trihydroxyacetophenone (THAP) as the matrix. High-resolution mass spectra (HRMS) were recorded with an Agilent 6210 ESI-TOF mass spectrometer at the Freie Universität Berlin (Mass Spectrometry Core Facility) and are given in *m/z*.

Experimental details and characterization data for known compounds

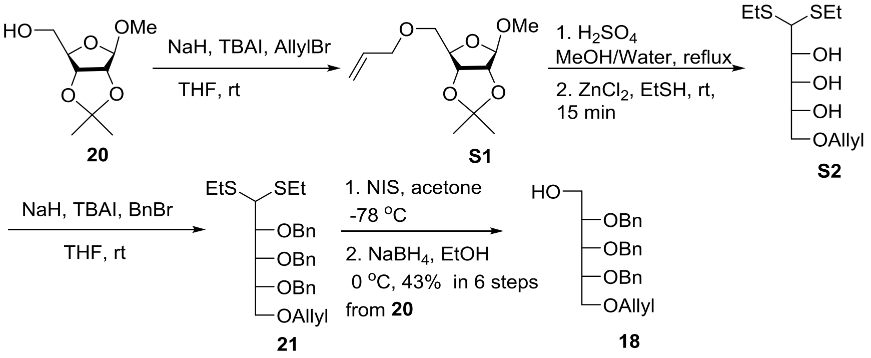

Scheme S1. Synthesis of ribitol building block **18**

Methyl 5-*O*-allyl-2,3-*O*-isopropylidene-β-D-ribofuranoside (S1)[1,2]

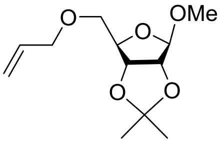

To a solution of commercially available Methyl 2,3-*O*-isopropylidene-β-D-ribofuranoside **20** (30 g, 147 mmol) in THF (300 mL) was added tetrabutylammonium iodide (TBAI) (5.43 g, 14.69 mmol) and the reaction mixture was cooled to 0 °C. Sodium hydride (11.75 g, 294 mmol, 60% in oil dispersion) was added and the reaction stirred for 10 min, after which allylbromide (25.4 mL, 294 mmol) was added at 0 °C and the reaction mixture was allowed to warm to room temperature over 30 min. The resulting

solution was stirred at room temperature for 20 h. The reaction mixture was diluted with water (100 mL) and extracted with EtOAc (100 mL). The combined organic phase was washed with brine, dried over $MgSO_4$, filtered and concentrated under vacuum. The obtained residue was diluted with hexanes (400 mL) and concentrated to provide the desired compound as an oil, which was carried forward into the next step without purification.

R_f = 0.30 (Hexanes/EtOAc, 5:1, v/v); ^{1}H NMR (400 MHz, $CDCl_3$) δ 1.29 (s, 3H), 1.45 (s, 3H), 3.29 (s, 3H), 3.35-3.47 (m, 2H), 3.99 (dd, *J* = 6.4, 2.8 Hz, 2H), 4.30 (t, *J* = 7.6 Hz, 1H), 4.54 (d, *J* = 6.0 Hz, 1H), 4.65 (d, *J* = 6.0 Hz, 1H), 4.93(s, 1H), 5.15 (dd, *J* = 10.4, 1.2 Hz, 1H), 5.25 (dd, *J* = 17.2, 1.6 Hz, 1H), 5.92–5.80 (m, 1H); ^{13}C NMR (101 MHz, $CDCl_3$) δ 25.2, 26.7, 54.9, 71.1, 72.3, 82.3, 85.2, 85.3, 109.3, 112.4, 117.2, 134.7.

5-*O*-Allyl-D-ribose diethyl dithioacetal (S2) [1,2]

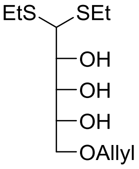

To a solution of **S1** (33.9 g, 139 mmol) in a mixture of methanol (1300 mL) and water (170 mL) was added concentrated sulfuric acid (7.40 mL) and the reaction mixture was refluxed for 3 h. The mixture was cooled to room temperature and the solvents removed under vacuum. The residue was quenched with aqueous (aq) sat. $NaHCO_3$ (200 mL), extracted with EtOAc (400 mL), washed with brine (500 mL), dried over $MgSO_4$, filtered and concentrated to obtain the crude methyl 5-*O*-allyl-β-D-ribofuranoside as an oil, which was carried forward into the next step without purification.

Anhydrous zinc chloride (53.4 mL, 392 mmol) was added to a solution of methyl 5-*O*-allyl-β-D-ribofuranoside (20 g, 98 mmol) in EtSH (87 mL, 1175mmol) at room temperature. After stirring for 1 h, the reaction was quenched with 0.1 N HCl (150 mL). EtSH was removed from the residue by extraction with EtOAc (400 mL) and treatment with aq. sat. $NaHCO_3$ (200 mL). The combined organic phase was washed with brine (500 mL). After drying with $MgSO_4$, filtration and concentration yielded the crude product as a yellowish oil (24.8 g), which was carried forward into the next step without purification.

R_f = 0.36 (Hexanes/EtOAc, 1:1, v/v); ^{1}H NMR (400 MHz, $CDCl_3$) δ 1.24–1.28 (m, 6H), 2.55–2.71 (m, 4H), 3.64–3.70 (dd, *J* = 5.0, 1.2 Hz, 2H), 3.84–3.90 (m, 1H), 3.93–4.05 (m, 4H), 4.24 (s, 1H), 5.15–5.30 (m, 2H), 5.80–5.92 (m, 1H); ^{13}C NMR (101 MHz, $CDCl_3$) δ 14.6, 14.7, 25.8 (2C), 54.7, 71.3, 71.7, 72.6, 73.1, 75.2, 117.8, 134.1.

5-*O*-allyl-2,3,4-Tri-*O*-benzyl-D-ribose diethyl dithioacetal (21) [1,2]

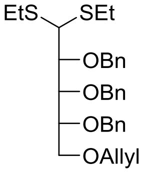

To a solution of **S2** (24.8 g, 84 mmol) in a mixture of THF (320 mL) and DMF (80 mL) at 0 °C was added NaH (12.05 g, 502 mmol, 60% in oil dispersion), followed by slow addition of benzyl bromide (49.8 mL, 418 mmol), after which the mixture was stirred at room temperature for 12 h. The reaction was quenched using ice cold water (100 mL) and extracted with EtOAc (400 mL). The combined organic phase was washed with brine (300 mL), dried over $MgSO_4$, filtered and concentrated to yield **21** as a yellowish oil, which was carried forward into the next step without purification.

R_f = 0.60 (Hexanes/EtOAc, 5:1, v/v); ^{1}H NMR (400 MHz, $CDCl_3$) δ 1.10–1.20 (m, 6H), 2.56–2.65 (m, 4H), 3.55–3.68 (m, 2H), 3.87 (dd, *J* = 5.5, 1.4 Hz, 2H), 3.93 (dd, *J* = 7.8, 3.1 Hz, 1H), 3.97–4.03 (m, 1H), 4.07 (dd, *J* = 7.7, 2.4 Hz, 1H), 4.20 (d, *J* = 3.1 Hz, 1H), 4.50–4.67 (m, 3H), 4.77 (d, *J* = 11.1 Hz, 1H), 4.90 (d, *J* = 11.0 Hz, 1H), 5.05–5.10 (m, 1H), 5.18 (dd, *J* = 17.3, 1.8 Hz, 2H), 5.77–5.87 (m, 1H), 7.17–7.33 (m, 15H); ^{13}C NMR (101 MHz, $CDCl_3$) δ 14.6 (2C), 25.1, 26.4, 54.1, 70.9, 72.3, 74.8, 79.2, 80.1, 82.5, 116.7, 127.5, 127.6, 127.7, 127.8, 128.0, 128.3 (2C), 128.4, 135.0, 138.4, 138.7, 139.0.

5-*O*-allyl-D-2,3,4-Tri-*O*-benzyl-ribitol (18) [1,2]

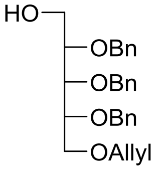

To a solution of **21** (18 g, 31.76 mmol) in acetone (130 mL) at -78 °C was added a solution of N-iodosuccinimide (NIS) (29 g, 127 mmol) in acetone (60 mL) in one portion. After 2h the reaction was complete, and the reaction mixture was quickly poured into a solution of aq. sat. $Na_2S_2O_3$ (300 mL) and vigorously shaken until the reaction mixture became colorless. After evaporating acetone under vacuum, the residue was extracted with CH_2Cl_2 (400 mL), the organic layer was washed with aq. sat. $NaHCO_3$ (300 mL), dried over $MgSO_4$, filtered and concentrated to afford the intermediate aldehyde (15 g), which was carried forward into the next step without purification.

To a solution of the aldehyde from the step above at 0 °C in EtOH (200 mL) was added $NaBH_4$ (868 mg, 22.94 mmol). The reaction mixture was allowed to warm to room temperature, stirred for 30 min, quenched at 0 °C by the dropwise addition of aq. sat. NH_4Cl and stirred for an additional 1.5 h. After removal of EtOH *in vacuo*, the resulting mixture was extracted with EtOAc (400 mL), and the organic layer was dried over $MgSO_4$, filtered and concentrated under vacuum. The residue was purified by flash

column chromatography (Hexanes/EtOAc, 1:1, v/v) to afford **18** (13 g, 43% over 6 steps) as a colorless oil.

R_f = 0.36 (Hexanes/EtOAc, 1:1, v/v); ^{1}H NMR (400 MHz, $CDCl_3$) δ 2.65 (br, 1H), 3.57–4.10 (m, 9H), 4.39 (t, *J* = 5.0 Hz, 1H), 4.53–4.75 (m, 5H), 4.84 (s, 1H), 5.15 (d, *J* = 10.4 Hz, 1H), 5.24 (dd, *J* = 17.2, 1.5 Hz, 1H), 5.80–5.94 (m, 1H), 7.20–7.40 (m, 15H); ^{13}C NMR (101 MHz, $CDCl_3$) δ 68.1, 69.8, 72.3, 72.4, 73.8, 76.0, 78.1, 78.5, 84.6, 117.0, 127.7 (2C), 128.1, 128.3, 128.4 (2C), 134.9, 138.2, 138.4.

2,3,4-tri-*O*-benzyl-1-*O*-β-D-ribofuranosyl-(1→1)- 5-*O*-allyl-2, 3, 4-tri-*O*-benzyl-D-ribitol (17)[3,4]

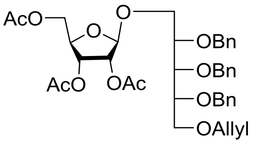

To a solution of **18** (10.90 g, 23.56 mmol) in CH_2Cl_2 (100 mL) was added BF_3•Et_2 (6.85 mL, 54.0 mmol) at 0 °C. After stirring for 5 min, a solution of **19** (5.16 g, 16.21 mmol) in CH_2Cl_2 (30 mL), was added over 10 min at 0 °C. The reaction was stirred for 15 min at 0 °C and then allowed to warm to room temperature over 1 h. The reaction mixture was quenched with triethylamine (10 mL) and combined with aq. sat. $NaHCO_3$ (100 mL), then extracted with CH_2Cl_2 (300 mL). The combined organic phase was washed with brine (100 mL), dried over $MgSO_4$, filtered and concentrated under vacuum. The residue was purified by flash column chromatography (Hexanes/EtOAc, 4:1, v/v) to obtain compound **17** as a colorless oil (9.4 g, 80%) and recover acetylated **18** (2.5g).

R_f = 0.40 (Hexanes/EtOAc, 4:1, v/v); $[\alpha]^{20}_D$ = -8.5 (*c* 1.0, $CHCl_3$); ^{1}H NMR (400 MHz, $CDCl_3$) δ 1.98 (s. 3H), 2.06 (s, 3H), 2.09 (s, 3H), 3.61-3.65 (m, 1H), 3.67 (dd, *J* = 7.5, 3.5 Hz, 1H), 3.77-3.97 (m, 7H), 4.03-4.17 (m, 2H), 4.23-4.33 (m, 2H), 4.58-4.65 (m, 3H), 4.67-4.75 (m, 4H), 4.93 (s, 1H), 5.21-5.25 (m, 1H), 5.28-5.34 (m, 1H), 5.83-5.95 (m, 1H), 7.22-7.38 (m, 15H) ;^{13}C NMR (101 MHz, $CDCl_3$) δ 20.60, 20.62, 20.9, 60.4, 65.5, 67.2, 72.0, 72.1, 72.16, 72.23, 73.6, 74.7, 77.9, 78.1, 78.5, 80.6, 107.2, 116.8, 127.4, 127.5, 127.7 (2C), 127.8, 128.1 (3C), 134.7, 138.2 (2C), 138.3, 171.0 (2C), 171.3.

2,3,4-tri-*O*-benzyl-1-*O*-β-D-ribofuranosyl-(1→1)-5-*O*-allyl-D-ribitol (22)[3]

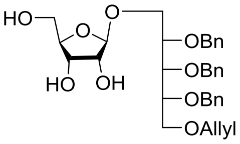

To a solution of **17** (9.4 g, 13.04 mmol) in MeOH (100 mL) was added solid NaOMe (78 mg, 1.387 mmol). The solution was stirred for 3 h at room temperature, then neutralized with Dowex amberlite-120 (H+) resin, filtered over Celite and the filtrate concentrated under vacuum to afford **22** as a colorless oil (7.0 g, 90%).

R_f = 0.46 (Hexanes/EtOAc, 5:1, v/v); $[\alpha]^{20}_D$ = -10.5 (*c* 1.0, $CHCl_3$); ^1H NMR (400 MHz, $CDCl_3$) δ 2.43 (brs, 3H), 3.55–3.63 (m, 3H), 3.68 (dd, *J* = 10.5, 3.3 Hz, 1H), 3.72–3.79 (m, 2H), 3.81–3.92 (m, 3H), 3.93–3.99 (m, 4H), 4.00–4.05 (m, 1H), 4.39 (t, *J* = 5.7 Hz, 1H), 4.57 (d, *J* = 11.7 Hz., 1H), 4.58–4.67 (m, 3H), 4.72 (d, *J* = 11.8 Hz, 1H), 4.84 (s, 1H), 5.16 (dd, *J* = 10.4, 1.6 Hz, 1H), 5.25 (dd, *J* = 17.2, 1.7 Hz, 1H), 5.83–5.93 (m, 1H), 7.25–7.36 (m, 15H); ^{13}C NMR (101 MHz, $CDCl_3$) δ 62.7, 68.1, 69.8, 70.9, 72.3, 72.4, 72.5, 73.9, 75.9, 77.5, 78.0, 78.1, 78.4, 84.7, 107.6, 117.1, 127.8, 128.1, 128.3, 128.5, 134.9, 138.2, 138.5.

2, 5-di-*O*-benzyl-β-D-ribofuranosyl-(1→1)-5-*O*-allyl-2,3,4-tri-*O*-benzyl-D-ribitol (13)[4]

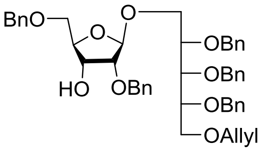

Compound **22** (2.75 g, 4.62 mmol) and dibutyltin oxide (1.73 g, 6.94 mmol) in toluene (50 mL) were refluxed for 2 h and then concentrated under vacuum. The crude acetal was dissolved in DMF (50 mL), and NaH (0.56 g, 13.87 mmol, 60% in oil dispersion) and TBAI (6.83g, 18.50 mmol) were added sequentially at room temperature and the reaction stirred at 80 °C for 1 h. Benzyl chloride (1.33 mL, 11.55 mmol) was then added and the reaction stirred at 80 °C for 5 h. The reaction mixture was cooled to room temperature and quenched with MeOH (10 mL) and aq. sat. NH_4Cl (50 mL). The aqueous layer was extracted with EtOAc (200 mL), dried over $MgSO_4$, filtered and concentrated under vacuum. The residue was purified by flash column chromatography (Hexanes/EtOAc, 7:1, v/v) to obtain **13** (3.6 g, 50%) as a colorless oil.

R_f = 0.32 (Hexanes/EtOAc, 7:1, v/v); $[\alpha]^{20}_D$ = -25.4 (*c* 1.0, $CHCl_3$); ^1H NMR (400 MHz, $CDCl_3$) δ 2.61 (d, *J* = 8.0 Hz, 1H), 3.52 (dd, *J* = 10.3, 6.4 Hz, 1H), 3.58–3.68 (m, 2H), 3.68–3.75 (m, 2H), 3.80–3.87 (m, 3H), 3.93–3.99 (m, 3H), 4.07–4.17 (m, 2H), 4.44 (d, *J* = 12.1 Hz, 1H), 4.48 (d, *J* = 12.1 Hz, 1H), 4.52–4.69 (m, 6H), 4.72 (d, *J* = 11.7 Hz, 1H), 5.01 (s, 1H), 5.10–5.17 (m, 1H), 5.20–5.28 (m, 1H), 5.82–5.95 (m, 1H), 7.18–7.45 (m, 25H); ^{13}C NMR (101 MHz, $CDCl_3$) δ 69.6, 70.2, 72.0, 72.2, 72.4, 72.6, 72.7, 73.4, 73.9, 78.2, 78.6, 78.8, 81.9, 83.2, 105.0, 116.9, 127.6 (2C), 127.7, 128.0, 128.1, 128.2, 128.4, 128.7, 135.0, 137.3, 138.3, 138.6, 138.8.

3-*O*-levulinyl-2, 5-di-*O*-benzyl-β-D-ribofuranosyl-(1→1)-5-*O*-allyl-2,3,4-tri-*O*-benzyl-D-ribitol (23)[4]

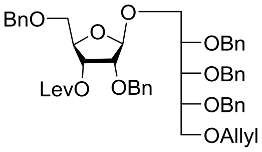

Compound **13** (2.5 g, 3.65 mmol) and *N,N*-diisopropylcarbodiimide (DIC) (0.853 mL, 5.48 mmol) were dissolved in CH_2Cl_2 (50 mL). DMAP (446 mg, 3.65 mmol) and levulinic acid (0.56 mL, 5.48 mmol) were added sequentially to the reaction mixture at room temperature. After 3 h, the aqueous layer was diluted with CH_2Cl_2 (30 mL) and aq. sat. $NaHCO_3$ (200 mL). The reaction was extracted with CH_2Cl_2 and the organics dried over $MgSO_4$, filtered and concentrated under vacuum. The residue was purified by flash column chromatography (Hexanes/EtOAc, 4:1, v/v) to obtain **23** as a colorless oil (2.7 g, 85%). R_f = 0.32 (Hexanes/EtOAc, 4:1, v/v); $[\alpha]^{20}_D$ = -30.8 (*c* 1.0, $CHCl_3$); ^{1}H NMR (400 MHz, $CDCl_3$) δ 2.13 (s, 3H), 2.55–2.62 (m, 2H), 2.65–2.70 (m, 2H), 3.50–3.57 (m, 2H), 3.60–3.66 (m, 1H), 3.68–3.75 (m, 2H), 3.80–3.89 (m, 3H), 3.91–3.99 (m, 3H), 4.09 (dd, *J* = 5.1, 2.0 Hz, 1H), 4.32 (q, *J* = 5.4 Hz, 1H), 4.42 (d, *J* = 12.0 Hz, 1H), 4.46 (d, *J* = 12.1 Hz, 1H), 4.50–4.59 (m, 3H), 4.61–4.69 (m, 4H), 4.72 (d, *J* = 11.8 Hz, 1H), 5.02 (d, *J* = 2.0 Hz, 1H), 5.12–5.18 (m, 2H), 5.25 (dd, *J* = 17.2, 1.7 Hz, 1H), 5.83–5.94 (m, 1H), 7.17–7.38 (m, 25H); ^{13}C NMR (101 MHz, $CDCl_3$) δ 28.4, 30.1, 38.1, 66.6, 68.0, 70.2, 71.6, 72.3, 72.4, 72.5, 73.0, 73.4, 73.6, 73.9, 74.0, 78.3, 78.5, 78.8, 80.3, 80.9, 106.2, 116.9, 127.7, 127.8, 127.9 (2C), 128.1, 128.3, 128.4 (3C), 128.5, 128.7, 135.1, 136.0, 137.8, 138.3, 138.6,138.8, 172.8, 206.7.

3-*O*-levulinyl-2, 5-di-*O*-benzyl-β-D-ribofuranosyl-(1→1)-2,3,4-tri-*O*-benzyl-D-ribitol (14) [1,2]

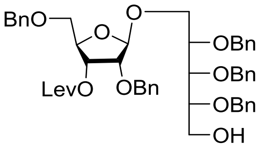

To a mixture of **23** (389 mg, 0.446 mmol) and 1,3-dimethylbarbituric acid (209 mg, 1.337 mmol) in MeOH (5 mL) was added $Pd(PPh_3)_4$ (103 mg, 0.089 mmol) at room temperature. The resulting mixture was stirred at 40 °C for 5 h. The mixture was cooled to room temperature and diluted with EtOAc (20 mL) and aq. sat. $NaHCO_3$ (50 mL). The aqueous layer was extracted with EtOAc (100mL) and the combined organic layers were washed with brine (100 mL), dried over $MgSO_4$ filtered, and concentrated under vacuum. The crude product was purified by flash column chromatography (Hexanes/EtOAc, 4:1, v/v) to yield the deallylated product **14** (278 mg, 75%) as a colorless oil.

R_f = 0.30 (Hexanes/EtOAc, 4:1, v/v); $[\alpha]^{20}_D$ = -9.8 (*c* 0.6, $CHCl_3$); ^{1}H NMR (400 MHz, $CDCl_3$) δ 2.14 (s, 3H), 2.55–2.62 (m, 2H), 2.65–2.70 (m, 2H), 3.50–3.58 (m, 2H), 3.66–3.76 (m, 4H), 3.80 (dd, *J* = 9.4, 4.6 Hz, 1H), 3.87 (t, *J* = 4.9 Hz, 1H), 3.94 (dd, *J* = 10.6, 5.5 Hz, 1H), 4.09 (dd, *J* = 5.0, 2.1 Hz, 1H), 4.32 (q, *J* = 5.1 Hz, 1H), 4.42 (d, *J* = 12.1 Hz, 1H), 4.46 (d, *J* = 12.1 Hz, 1H), 4.50–4.59 (m, 4H), 4.62–4.69 (m, 3H), 5.01 (d, *J* = 2.1 Hz, 1H), 5.16 (t, *J* = 5.3 Hz, 1H), 7.17–7.38 (m, 25H); ^{13}C NMR (101 MHz, $CDCl_3$) δ 28.1, 30.0, 38.0, 61.5, 67.5, 71.4, 72.0, 72.5, 73.0, 73.4, 73.8, 74.1, 77.9, 78.9, 79.1, 80.3, 80.8, 106.1, 127.6, 127.8, 127.9, 128.0, 128.3, 128.5, 137.8, 138.2, 138.3, 172.3, 206.4.

2, 5-di-*O*-benzyl-β-D-ribofuranosyl-(1→1)-2,3,4-tri-*O*-benzyl-D-ribitol (24) [1,2]

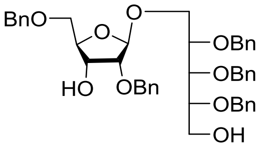

To a mixture of **13** (3.39 g, 5.07 mmol) and 1,3-dimethylbarbituric acid (1158 mg, 10.51 mmol) in MeOH (10 mL) was added $Pd(PPh_3)_4$ (1.17 g, 1.051 mmol) at room temperature. The resulting mixture was stirred at 40 °C for 5 h. The mixture was cooled to room temperature and diluted with EtOAc (100 mL) and aq. sat. $NaHCO_3$ (100 mL). The aqueous layer was extracted with EtOAc (100 mL) and the combined organic layers were washed with brine (100 mL), dried over $MgSO_4$, filtered, and concentrated under vacuum. The residue was purified by flash column chromatography (Hexanes/EtOAc, 3:1, v/v) to yield **24** (2.79 g, 75%) as a colorless oil.

R_f = 0.35 (Hexanes/EtOAc, 3:1, v/v); $[\alpha]^{20}_D$ = -20.5 (*c* 0.8, $CHCl_3$); ^{1}H NMR (400 MHz, $CDCl_3$) δ 3.49 (dd, *J* = 10.5, 5.9 Hz, 1H), 3.57 (dd, *J* = 10.5, 4.0 Hz, 1H), 3.65–3.77 (m, 4H), 3.79 (dd, *J* = 9.5, 4.6 Hz, 1H), 3.82–3.87 (m, 2H), 3.93 (dd, *J* = 10.8, 5.5 Hz, 1H), 4.00 (dd, *J* = 6.9, 4.7 Hz, 1H), 4.34 (td, *J* = 6.2, 4.1 Hz, 1H), 4.42–4.47 (m, 3H), 4.50 (d, *J* = 9.1 Hz, 1H), 4.53–4.67 (m, 8H) , 5.03 (s, 1H), 7.22–7.35 (m, 25H); ^{13}C NMR (100 MHz, $CDCl_3$) δ 61.4, 67.0, 71.5, 72.0, 72.2, 72.3, 72.4, 74.0, 77.9, 78.4, 79.1, 79.7, 80.6, 105.4, 127.5 (2C), 127.7, 127.8 (2C), 127.9 (2C), 128.1, 128.3 (2C), 128.4 (2C), 128.5, 128.6, 137.8 (2C), 138.1, 138.2, 138.3.

2, 5-di-*O*-benzyl-β-D-ribofuranosyl-(1→1)-5-*O*-dimethoxytrityl-2,3,4-tri-*O*-benzyl-D-ribitol (15) [1,2]

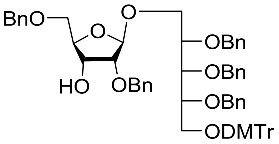

Dimethoxytrityl chloride (2.24 g, 6.61 mmol) was added to a solution of **24** (3.24 g, 4.41 mmol) in CH_2Cl_2 (5 mL), followed by pyridine (0.106 mL, 13.22 mmol) and DMAP (538 mg, 4.41 mmol) at room temperature. After stirring for 12 h, the reaction mixture was quenched using aq. sat. $NaHCO_3$ (100 mL) and extracted with CH_2Cl_2 (200 mL). The combined organic layers were washed with brine (100 mL), dried over $MgSO_4$ filtered, and concentrated under vacuum. The residue was purified by flash column chromatography (Hexanes/EtOAc, 4:1, v/v) to yield **15** (4.11 g, 90%) as a colorless oil.

R_f = 0.35 (Hexanes/EtOAc, 4:1, v/v); $[\alpha]^{20}_D$ = -10.2 (*c* 0.5, $CHCl_3$); ^{1}H NMR (400 MHz, $CDCl_3$) δ 2.49 (d, *J* = 8.8 Hz, 1H), 3.29 (dd, *J* = 10.2, 5.5 Hz, 1H), 3.36 (dd, *J* = 10.1, 2.4 Hz, 1H), 3.42 (dd, *J* = 10.3, 6.4 Hz, 1H), 3.51 (dd, *J* = 10.3, 3.9 Hz, 1H), 3.61 (dd, *J* = 10.6, 3.0 Hz, 1H), 3.66 (s, 3H), 3.65 (s, 3H), 3.72–3.89 (m, 5H), 3.96–4.08 (m, 2H), 4.36 (d, *J* = 12.2 Hz, 1H), 4.39–4.57 (m, 7H), 4.69 (d, *J* = 11.6 Hz, 1H), 4.90 (s, 1H), 6.64–6.67 (m, 4H), 6.97–7.42 (m, 34H); ^{13}C NMR (100 MHz, $CDCl_3$) δ 55.2, 63.8, 67.7, 72.0, 72.2, 72.4, 72.6, 72.7, 73.3, 73.8, 78.4, 79.0 (2C), 81.9, 83.21, 86.2, 104.8, 113.1, 126.7,

127.5, 127.6, 127.7 (2C), 127.8, 127.9, 128.0, 128.2, 128.3, 128.3 (2C), 128.6, 130.3, 136.4, 137.2, 138.3, 138.5, 138.7, 138.8, 145.3, 158.4.

3-*O*-triethylammoniumphosphonate-2, 5-di-*O*-benzyl-β-D-ribofuranosyl-(1→1)-5-*O*-dimethoxytrityl-2,3,4-tri-*O*-benzyl-D-ribitol (16)[4]

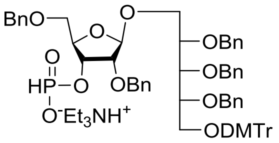

Imidazole (836 mg, 12.29 mmol) was dissolved in CH_2Cl_2 (10 mL) and the solution cooled to 0 °C. PCl_3 (0.58 mL, 6.74 mmol) and Et_3N (1.71 mL, 12.29 mmol) were added to the reaction mixture. The resulting solution was stirred for 5 min and then a solution of **15** (1.75 g, 1.686 mmol) was added over 5 min. After stirring for 1h at room temperature, the reaction mixture was quenched using aq. sat. $NaHCO_3$ (50 mL) and 1.0 M triethylammonium bicarbonate (TEAB) solution, and extracted with CH_2Cl_2 (100 mL). The combined organic layers were dried over $MgSO_4$, filtered and concentrated under vacuum. The residue was purified by flash column chromatography (DCM/MeOH, 10:1, v/v) to yield **16** (1.58 g, 85%) as a colorless oil.

R_f = 0.40 (DCM/MeOH, 10:1, v/v); ^{1}H NMR (400 MHz, $CDCl_3$) δ 1.14 (t, NCH_2CH_3), 2.86 (q, NCH_2CH_3), 3.36 (dd, *J* = 10.2, 5.5 Hz, 1H), 3.43 (dd, *J* = 10.1, 2.1 Hz, 1H), 3.54 (dd, *J* = 10.5, 6.7 Hz, 1H), 3.66–3.73 (m, 2H), 3.75 (s, 3H), 3.76 (s, 3H), 3.83–3.97 (m, 5H), 4.03 (dd, *J* = 4.6, 1.2 Hz, 1H), 4.34 (td, *J* = 6.4, 3.3 Hz, 1H), 4.42 (d, *J* = 12.1 Hz, 1H), 4.46–4.55(m, 4H), 4.58–4.66 (m, 4H), 4.67–4.77 (m, 3H), 5.01 (d, *J* = 1.2 Hz, 1H), 6.73–6.76 (m, 4H), 7.08–7.13 (m, 2H), 7.17–7.38 (m, 30H), 7.44–7.49 (m, 2H); ^{13}C NMR (101 MHz, $CDCl_3$) δ 8.5 (NCH_2CH_3), 45.4 (NCH_2CH_3), 53.6, 55.2, 63.8, 68.0, 72.0, 72.4, 72.5, 72.7, 73.3, 73.4, 73.8, 78.6, 78.9, 79.2, 81.6, 82.0, 86.2, 106.0, 113.1, 126.7, 127.4, 127.5, 127.6, 127.7, 127.8 (2C), 127.9, 128.0, 128.2, 128.3 (2C), 128.3 (2C), 130.3, 136.4, 136.5, 138.4, 138.5, 138.6, 138.8, 138.9, 145.3, 158.4; ^{31}P NMR (162 MHz, $CDCl_3$) δ 3.55.

Experimental details and characterization data of new compounds

Compound 10

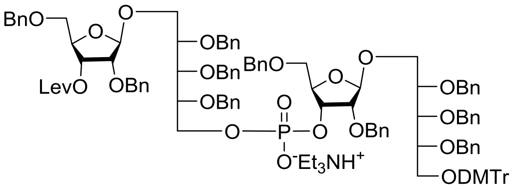

H-phosphonate **16** (835 mg, 0.759 mmol) and compound **14** (632 mg, 0.759 mmol) were dissolved in in pyridine (7 mL). Trimethylacetyl chloride (PivCl, 0.28 mL, 2.28 mmol) was then slowly added. The reaction mixture was stirred for 1 h at room temperature. A solution of iodine (193 mg, 0.759 mmol) in

pyridine-water (96:4, v/v; 5 mL) was added and the reaction was further stirred for 30 min at room temperature. The reaction mixture was diluted with CH_2Cl_2 (5 mL), washed with aq. sat. $Na_2S_2O_3$ (50 mL) and then with 1.0 M TEAB solution (20 mL). The combined organic layers were dried over $MgSO_4$, filtered, and concentrated under vacuum. The residue was purified by flash column chromatography (DCM/MeOH, 10:1, v/v) to yield **10** (1.25 g, 85%) as a colorless oil.

R_f = 0.35 (DCM/MeOH, 10:1, v/v); ^{1}H NMR (600 MHz, $CDCl_3$) δ 1.08 (t, NCH_2CH_3), 2.18 (s, 3H), 2.66 (dd, *J* = 10.4, 4.0 Hz, 2H), 2.69-2.75 (m, 2H), 2.77 (q, NCH_2CH_3), 3.43 (dd, *J* = 9.6, 5.1 Hz, 1H), 3.50 (dd, *J* = 9.5, 2.5 Hz, 1H), 3.55–3.64 (m, 4H), 3.73 (dd, *J* = 10.2, 2.2 Hz, 1H), 3.79 (s, 3H), 3.80 (s,3H), 3.82–3.84 (m, 1H), 3.91-4.05 (m, 7H), 4.14–4.19 (m, 3H), 4.35–4.53 (m, 7H), 4.54–4.78 (m, 6H), 4.81-4.90 (m, 3H), 5.06 (d, *J* = 2.0 Hz, 1H), 5.10 (d, *J* = 2.0 Hz, 1H), 5.21 (t, *J* = 5.3 Hz, 1H), 6.70–6.75 (m, 4H), 7.05–7.70 (m, 60H); ^{13}C NMR (151 MHz, $CDCl_3$) δ 8.3 (NCH_2CH_3), 27.9, 29.8, 37.8, 45.1, 55.1, 63.6, 63.8, 65.0, 67.4, 67.8, 71.6, 72.2, 72.5, 72.8, 73.1, 73.2, 73.6, 73.8, 78.6 (2C), 78.9, 79.0, 80.1, 80.2, 80.7, 81.4, 86.0, 105.8, 106.1, 113.0, 126.6, 127.2, 127.3, 127.4, 127.5, 127.6, 127.7, 127.8, 127.9, 128.0, 128.2 (2C), 128.3, 130.1, 136.3, 137.7, 138.1, 138.5, 138.6 (2C), 138.8, 145.2, 158.3, 172.1, 206.2; ^{31}P NMR (243 MHz, $CDCl_3$) δ -1.52; MALDI-TOF m/z calcd for $C_{116}H_{123}O_{24}P$ $[M+H]^+$:1930.8142, Found:1930.8310.

Compound 11

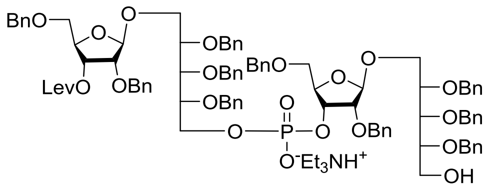

A solution of DMTr protected dimer **10** (1.28 g, 0.662 mmol) was dissolved in CH_2Cl_2 (10 mL), and trichloroacetic acid (108 mg, 0.662 mmol) was added at room temperature. After stirring for 5 h, the reaction was quenched with aq. sat. $NaHCO_3$ (100 mL) followed by 1.0 M TEAB solution (30 mL). The combined organic layers were washed with brine (100 mL), dried over $MgSO_4$, filtered, and concentrated under vacuum. The residue was purified by flash column chromatography (DCM/MeOH, 10:1, v/v) to yield **11** (970 mg, 90%) as a colorless oil.

R_f = 0.30 (DCM/MeOH, 10:1, v/v); ^{1}H NMR (400 MHz, $CDCl_3$) δ 1.07 (t, NCH_2CH_3), 2.14 (s, 3H), 2.57 (dt, *J* = 7.9, 4.0 Hz, 2H), 2.67 (dt, *J* = 7.9, 4.0 Hz, 2H), 2.74 (q, NCH_2CH_3), 3.47–3.54 (m, 2H), 3.60–3.77 (m, 6H), 3.78–3.98 (m, 7H), 4.05–4.14 (m, 2H), 4.29–4.46 (m, 6H), 4.48–4.70 (m, 12H), 4.75–4.85 (m, 2H), 4.98 (d, *J* = 1.7 Hz, 1H), 5.04 (d, *J* = 2.2 Hz, 1H), 5.13 (t, *J* = 5.6 Hz, 1H), 6.91–7.48 (m, 55H);^{13}C NMR (100 MHz, $CDCl_3$) δ 8.3 (NCH_2CH_3), 28.0, 29.9, 38.0, 45.4 (NCH_2CH_3), 61.5, 67.4, 68.0, 71.7, 72.0, 72.2, 72.27, 72.33, 72.4, 72.86, 72.93, 73.2, 73.3, 73.8, 73.9, 74.01, 74.04,74.4, 78.0, 78.5, 78.8, 78.9, 79.1, 79.3, 80.2, 80.3, 80.9, 81.4, 82.3, 106.0, 106.4, 127.3, 127.4, 127.5, 127.6,127.8, 128.0, 128.2, 128.3, 128.4, 137.8, 138.2, 138.3, 138.4, 138.6, 138.7, 138.9, 172.2, 206.3;

^{31}P NMR (162 MHz, $CDCl_3$) δ 0.01; MALDI-TOF m/z calcd for $C_{95}H_{105}O_{22}P$ $[M+H]^+$: 1628.6835, Found: 1628.4865.

Compound 12

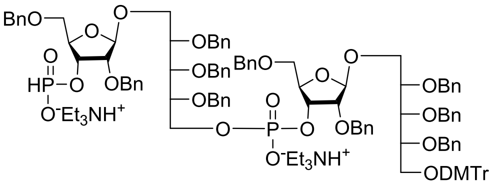

Hydrazine acetate (72.8 mg, 0.791 mmol) and pyridine (89 μL, 0.949 mmol) were added to a solution of **10** (1.50 g, 0.791 mmol) in CH_2Cl_2 (20 mL) at room temperature. After stirring for 5 h, the reaction mixture was quenched using aq. sat. $NaHCO_3$(50 mL) and extracted with CH_2Cl_2 (100 mL). The combined organic layers were washed with brine (50 mL), dried over $MgSO_4$, filtered, and concentrated under vacuum to get crude delevulinated compound (1.43 g). Imidazole (434 mg, 6.38 mmol) was dissolved in CH_2Cl_2 (10 mL) and the solution cooled to 0 °C. PCl_3 (0.28 mL, 3.19 mmol) and Et_3N (0.89 mL, 6.38 mmol) were added to the reaction mixture. The resulting solution was stirred for 5 min and then a solution of crude delevulinated compound (1.43 g, 0.791mmol) was added. After stirring for 1h at room temperature, the reaction mixture was quenched using aq. sat. $NaHCO_3$(50 mL) and 1.0 M TEAB solution (20 mL), and extracted with CH_2Cl_2 (100 mL). The combined organic layers were dried over $MgSO_4$, filtered, and concentrated under vacuum.The crude product **12**, a colorless oil, was carried forward into the next coupling reaction without purification.

R_f=0.35(DCM/MeOH, 10:1, v/v); ^1H NMR (400 MHz, $CDCl_3$)δ1.03 (t, NCH_2CH_3), 2.70 (q, NCH_2CH_3), 3.36–3.50 (m, 5H), 3.50–3.72 (m, 3H), 3.74 (s, 3H), 3.75 (s, 3H), 3.76–3.82 (m, 2H), 3.88–4.06 (m, 8H), 4.14–4.27 (m, 4H), 4.39–4.85 (m, 21H), 4.83–4.91 (m, 3H), 5.02 (brs, 1H), 5.09 (brs, 1H), 6.73 – 6.83 (m, 4H), 7.10–7.57 (m, 59H); ^{13}C NMR (100 MHz, $CDCl_3$)δ8.4 (NCH_2CH_3), 27.4, 29.8, 45.3 (NCH_2CH_3), 55.3, 63.8, 65.3, 67.7, 68.1, 72.18, 72.23, 72.3, 72.4, 72.5, 72.6, 73.2 (2C), 73.7, 78.4, 78.9, 79.1, 81.4, 81.5, 81.9, 83.1, 83.2, 86.1,105.0, 106.2, 113.1, 126.7, 127.3 (2C), 127.5, 127.6, 127.7, 127.7, 127.7 (2C), 127.8, 127.9, 128.0, 128.09, 128.12, 128.2, 128.3 (3C), 128.4, 128.5, 128.6, 130.3, 136.4 (2C), 137.2, 138.2, 138.5, 138.6, 138.7, 138.8 (2C), 145.3, 158.4; ^{31}P NMR (162 MHz, $CDCl_3$) δ3.49, -0.10.

Compound 5

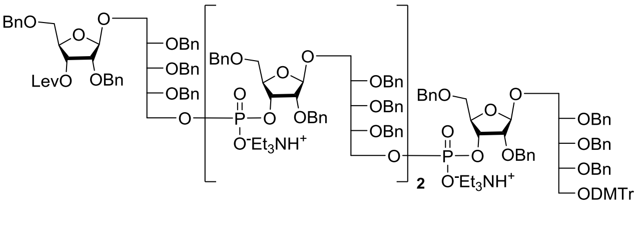

The crude H-phosphonate **12** (625 mg, 0.330 mmol) and compound **11** (483 mg, 0.297 mmol) were dissolved in pyridine (7 mL) and trimethylacetyl chloride (0.20 mL, 1.65 mmol) was added in one portion and stirred for 1 h at room temperature. A solution of iodine (84 mg, 0.330 mmol) in pyridine-water (96:4, v/v; 5 mL) was added and the reaction was further stirred for 30 min at room temperature. The reaction mixture was diluted with CH_2Cl_2 (5 mL), and the aqueous layer washed with aq. sat. $Na_2S_2O_3$ (100 mL) and then with 1.0 M TEAB solution (30 mL), dried over $MgSO_4$ filtered, and concentrated under vacuum. The residue was purified by flash column chromatography (DCM/MeOH, 10:1, v/v) to yield **5** (987 mg, 85%) as a colorless oil.

R_f = 0.30 (DCM/MeOH, 10:1, v/v); ^{1}H NMR (600 MHz, $CDCl_3$) δ 1.01 (t, NCH_2CH_3), 2.12 (s, 3H), 2.57 (t, *J* = 6.2 Hz, 2H), 2.63 (t, *J* = 6.2 Hz, 2H), 2.67 (q, NCH_2CH_3), 3.32 (dd, *J* = 10.0, 5.4 Hz, 2H), 3.39 (d, *J* = 8.2 Hz, 2H), 3.42–3.52 (m, 5H), 3.62–3.72 (m, 6H), 3.73 (s, 3H), 3.74 (s, 3H), 3.77–3.95 (m, 15H), 4.03–4.16 (m, 6H), 4.25–4.44 (m, 16H), 4.45–4.66 (m, 27H), 4.67–4.85 (m, 10H), 4.92–5.05 (m, 4H), 5.11 (t, *J* = 5.4 Hz, 1H), 6.68–6.74 (m, 4H), 6.99–7.49 (m, 109H); ^{13}C NMR (151 MHz, $CDCl_3$) δ 8.6 (NCH_2CH_3), 29.7, 29.8, 38.0, 45.2 (NCH_2CH_3), 55.2, 63.6, 65.1, 67.9, 71.6, 72.1, 72.2, 72.3, 72.5, 72.8, 73.1, 73.2, 73.7, 73.8, 74.0, 74.4, 78.4, 78.5, 78.8 (2C), 78.9, 79.1, 80.1, 80.8, 81.5, 82.3, 86.0, 105.9, 106.2, 113.0, 126.6, 127.2 (2C), 127.3, 127.4 (2C), 127.5 (2C), 127.6 (2C), 127.7 (2C), 127.8 (3C), 127.91, 128.0, 128.1, 128.15, 128.2 (2C), 128.3 (2C), 128.4, 130.2, 136.3, 136.4, 137.7, 138.1, 138.6 (2C), 138.7 (3C), 138.8 (2C), 138.8 (2C), 145.3, 158.3, 172.1, 206.3; ^{31}P NMR (243 MHz, $CDCl_3$) δ 0.73, -0.05, -0.47; MALDI-TOF m/z calcd for $C_{206}H_{220}O_{46}P_3^-$[M+2H]$^-$: 3522.409, Found: 3522.548.

5-azidopentyl H-phosphonate (9)

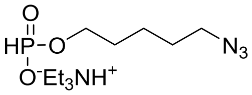

Imidazole (2.30 g, 33.76 mmol) was dissolved in CH_2Cl_2 (10 mL) and the solution cooled to 0 °C. PCl_3 (1.47 mL, 16.88 mmol) and Et_3N (4.70 mL, 33.76 mmol) were added to the reaction mixture. The resulting solution was stirred for 5 min and then a solution of 5-azido-1-pentanol[5] (545 mg, 4.22mmol) was added. After stirring for 1h at room temperature, the reaction mixture was quenched using aq. sat. $NaHCO_3$ (100 mL) and 1.0 M TEAB solution (30 mL), and extracted with CH_2Cl_2 (100 mL). The combined organic layers were dried over $MgSO_4$, filtered and concentrated under vacuum. The crude compound **13** was directly used in the downstream coupling reaction without purification.

Colorless oil, R_f = 0.50 (DCM/MeOH, 10:1, v/v); ^{1}H NMR (400 MHz, $CDCl_3$) δ 1.41–1.53 (m, 2H), 1.58–1.67 (m, 2H), 1.69–1.77 (m, 2H), 3.25–3.35 (m, 2H), 4.03–4.15 (m, 2H);^{13}C NMR (100 MHz, $CDCl_3$) δ 22.8, 28.3, 29.9, 51.1, 65.3; ^{31}P NMR (162 MHz, $CDCl_3$) δ 7.78.

Compound 25

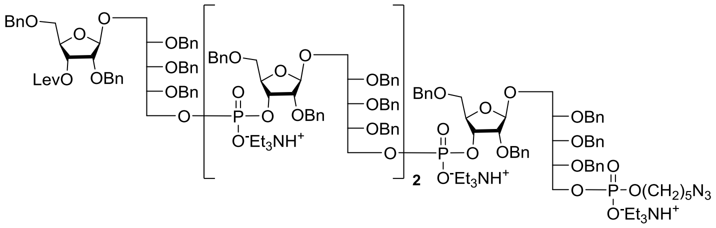

A solution of DMTr protected dimer **5** (175 mg, 0.050 mmol) was dissolved in CH_2Cl_2 (10 mL) and trichloroacetic acid (13 mg, 0.075 mmol) was added at room temperature. After stirring for 5 h, the reaction was quenched using aq. sat. $NaHCO_3$ (50 mL) and then with 1.0 M TEAB solution (20 mL). The organic layer was washed with brine (100 mL), dried over $MgSO_4$, filtered, and concentrated under vacuum. The residue was passed through silica gel (DCM/MeOH, 10:1, v/v) to yield the intermediate 5-OH tetramer; this compound (126 mg, 0.039 mmol) and 5-azidopentyl H-phosphonate (**9**, 15 mg, 0.078 mmol) were dissolved in pyridine (5 mL). Trimethylacetyl chloride (14 μL, 0.117 mmol) was added and the reaction mixture was stirred for 1 h at room temperature. A solution of iodine (20 mg, 0.078 mmol) in pyridine-water (96:4, v/v; 5 mL) was added and the reaction was stirred for 30 min at room temperature. The reaction mixture was diluted with CH_2Cl_2 (5 mL), and the organic layer was washed with aq. sat. $Na_2S_2O_3$ (50 mL) and then with 1.0 M TEAB solution (20 mL), dried over $MgSO_4$, filtered, and concentrated under vacuum. The residue obtained was purified by flash column chromatography (DCM/MeOH, 10:1, v/v) to yield **25** (110 mg, 80%) as a colorless oil.

R_f = 0.28 (DCM/MeOH, 10:1, v/v); ^{1}H NMR (600 MHz, $CDCl_3$) δ 1.05 (t, NCH_2CH_3), 1.29–1.39 (m, 2H), 1.43–1.59 (m, 4H), 2.11 (s, 3H), 2.52–2.61 (m, 2H), 2.62–2.66 (m, 2H), 2.72 (q, NCH_2CH_3), 3.12 (t, *J* = 7.2 Hz, 2H), 3.43–3.55 (m, 6H), 3.61–3.78 (m, 9H), 3.79–3.97 (m, 20H), 4.02–4.12 (m, 8H), 4.21–4.45 (m, 16H), 4.46–4.70 (m, 21H), 4.72–4.86 (m, 8H), 4.92–5.05 (m, 4H), 5.11 (t, *J* = 5.2 Hz, 1H), 7.10–7.10 (m, 100H); ^{13}C NMR (151 MHz, $CDCl_3$) δ 8.7 (NCH_2CH_3), 27.4, 28.1, 28.7, 29.9, 38.0, 45.4 (NCH_2CH_3), 52.7, 64.9, 65.1, 68.1, 71.7, 72.4, 72.9, 73.2, 73.3, 73.9, 74.1, 74.4, 78.1, 78.6 (2C), 79.0, 79.1, 80.2, 80.9, 81.6, 82.3, 106.0, 106.3, 127.3, 127.4, 127.6 (2C), 127.8, 128.0 (2C), 128.3, 128.4 (2C), 137.9, 138.3, 138.7, 138.9, 139.0, 172.2, 206.3; ^{31}P NMR (243 MHz, $CDCl_3$) δ -2.33, -3.00, -3.08; MALDI-TOF m/z calcd for $C_{190}H_{214}N_4NaO_{47}P_4^-$[M+Na+$NH_4$+H]$^-$: 3450.333, Found: 3450.689.

Compound 26

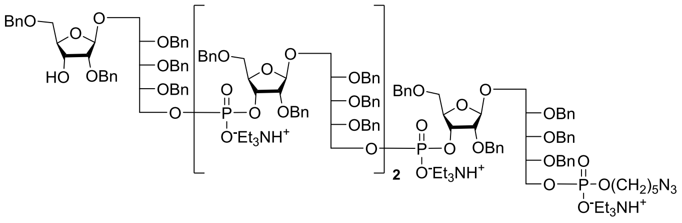

Hydrazine acetate (3.2 mg, 0.017 mmol) and pyridine (8 μL, 0.085 mmol) were added to a solution of

25 (58 mg, 0.017 mmol) in CH_2Cl_2 (5 mL) at room temperature. After stirring for 5 h, the reaction mixture was quenched using aq. sat. $NaHCO_3$ (50 mL) and extracted with CH_2Cl_2 (100 mL). The combined organic layers were washed with brine (50 mL), dried over $MgSO_4$, filtered, and concentrated under vacuum. The residue was purified by flash column chromatography (DCM/MeOH, 10:1, v/v) to yield **26** (50.7 mg, 90%) as a colorless oil.

R_f = 0.30 (DCM/MeOH, 10:1, v/v); ^{1}H NMR (600 MHz, $CDCl_3$) δ 1.02 (t, NCH_2CH_3), 1.30–1.35 (m, 2H), 1.46–1.57 (m, 4H), 2.67 (q, NCH_2CH_3), 3.12 (t, *J* = 7.0 Hz, 2H), 3.44–3.52 (m, 4H), 3.57 (dd, *J* = 10.3, 3.8 Hz, 2H), 3.62–3.76 (m, 8H), 3.88–3.97 (m, 18H), 4.03–4.12 (m, 8H), 4.25–4.47 (m, 18H), 4.48–4.70 (m, 22H), 4.70–4.83 (m, 11H), 4.94 (s, 1H), 4.96–5.03 (m, 3H), 6.97–7.46 (m, 100H); ^{13}C NMR (151 MHz, $CDCl_3$) δ 8.9 (NCH_2CH_3), 23.1, 28.6, 30.0, 30.0, 30.4, 32.0, 45.3 (NCH_2CH_3), 51.4, 64.9, 65.1, 65.2, 67.6, 68.0, 70.7, 72.2, 72.3, 72.6, 73.2, 73.3, 73.8, 74.5, 78.4, 78.6, 78.8, 79.0, 79.1 (2C), 81.6, 81.8, 82.3, 83.0, 104.9, 106.3 (2C), 106.4, 127.2, 127.3, 127.4, 127.5, 127.7 (2C), 127.8, 127.9 (2C), 128.0, 128.1, 128.2, 128.3, 128.6, 137.4, 138.3, 138.7, 138.8 (2C), 138.9 (2C), 139.0 (2C); ^{31}P NMR (243 MHz, $CDCl_3$) δ -2.33, -3.00, -3.08; MALDI-TOF m/z calcd for $C_{185}H_{207}N_3O_{45}P_4^-$[M+3H]$^-$: 3314.291, Found: 3314.328.

Compound 1

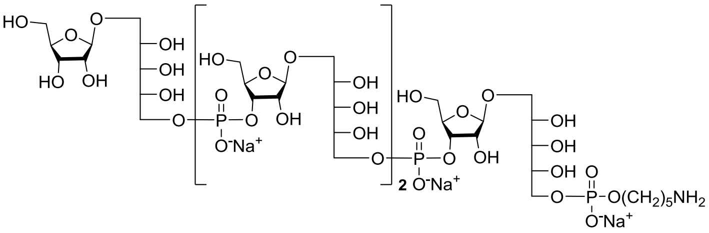

The compound **26** (58 mg, 0.017 mmol) was dissolved in a mixture of EtOAc/MeOH/50 % AcOH(aq) (1:1:0.5:0.2, v/v; 5 mL). Pd/C (10%)(10 mg) was added and the reaction mixture stirred under H_2 (40 psi) for 36 h. The reaction mixture was filtered through a pad of Celite and the filtrate concentrated. The crude product was purified by Sephadex LH-20 (H_2O) and the fractions lyophilized. Lyophilized compound was passed through a column packed with Dowex X-8 resin, Na^+ form, and eluted with Milli-Q water (10 mL). Lyophilization yielded compound **1** (13 mg, 85%) as a white foam.

^{1}H NMR (600 MHz, D_2O) δ 1.47–1.53 (m, 2H), 1.66–1.75 (m, 4H), 3.04 (t, *J* = 7.0 Hz, 2H), 3.61–3.81 (m, 13H), 3.82–3.86 (m, 3H), 3.87–4.08 (m, 20H), 4.09–4.13 (m, 4H), 4.17–4.23 (m, 3H), 4.26–4.29 (m, 4H), 4.59–4.70 (m, 3H), 5.04 (s, 1H), 5.08 (s, 3H); ^{13}C NMR (151 MHz, D_2O) δ 24.6, 28.9, 42.0, 65.0, 65.2, 68.4, 69.0, 69.3, 71.1, 71.2, 72.7, 73.2, 73.5, 74.0, 76.4, 76.9, 84.6, 85.3, 109.3, 109.5; ^{31}P NMR (243 MHz, D_2O) δ -1.96, -2.71, -2.81; MALDI-TOF m/z calcd for $C_{45}H_{85}NNa_3O_{45}P_4^-$[M+3Na]$^-$: 1552.304, Found: 1552.699.

General procedure for elongation reaction with the H-phosphonate intermediate 12 to produce sPRPs 6-8

To solutions of DMTr protected sPRPs **5**, **6**, or **7** (1 equiv.) in CH_2Cl_2 was added trichloroacetic acid (1.5 equiv.) at rt. After stirring for 5 h, the reaction was quenched with sat. $NaHCO_3$ and then with 1.0 M TEAB solution.The combined organic layers were washed with brine, dried over $MgSO_4$, filtered, and concentrated in vacuo. The obtained 5-OH intermediate compound was used without purification in the following step. H-phosphonate diester intermediate **12** (1.1 equiv.) and the 5-OH intermediate compound (1 equiv.) were dissolved in pyridine.Trimethylacetyl chloride (3 equiv.) was added and the reaction mixture stirred for 1 h at room temperature. A solution of I_2 (1 equiv.) in pyridine-water (96:4, v/v; 5 mL) was added and the reaction was further stirred for 30 min at room temperature. The reaction mixture was diluted with CH_2Cl_2, washed with sat. $Na_2S_2O_3$ and sat. $NaHCO_3$ followed by 1.0 M TEAB solution and extracted with CH_2Cl_2. The combined organic layers were washed with brine, dried over $MgSO_4$, filtered, and concentrated in vacuo. The residue was purified by silica gel chromatography (DCM/MeOH, 10:1, v/v).

General procedure for the coupling reaction of 9 with sPRPs 6-8 to give sPRP intermediates 27-29

To a solution of DMTr protected sPRP fragment (1 equiv.) in CH_2Cl_2 was added trichloroacetic acid (1.5 equiv.) at rt. After stirring for 5 h, the reaction was quenched with sat. $NaHCO_3$ and then with 1.0 M TEAB solution.The combined organic layers were washed with brine, dried over $MgSO_4$, filtered, and concentrated in vacuo. The obtained 5-OH intermediate PRP fragment compound was used without purification in the following step. H-phosphonate diester intermediate **9** (1.2 equiv.) and 5-OH intermediate PRP fragment compound (1 equiv.) were dissolved in pyridine.Trimethylacetyl chloride (3 equiv.) was added and the reaction mixture stirred for 1 h at rt. A solution of I_2 (1 equiv.) in pyridine-water (96:4, v/v; 5 mL) was added and the reaction was further stirred for 30 min at room temperature. The reaction mixture was diluted with CH_2Cl_2, washed with sat. $Na_2S_2O_3$ and sat. $NaHCO_3$ followed by 1.0 M TEAB solution and extracted with CH_2Cl_2. The combined organic layers were washed with brine, dried over $MgSO_4$, filtered, and concentrated in vacuo. The residue was purified by silica gel chromatography (DCM/MeOH, 10:1, v/v).

General procedure for hydrogenation of sPRP- intermediates 27-29 to give elongated sPRP fragments 2-4

The sPRP-linker (1 equiv.) was added to a mixture of EtOAc/MeOH/50 % AcOH (1:1:0.5:0.2, v/v; 5 mL). Pd/C (10%) was added and the reaction mixture was stirred under H_2 (50 psi) for 36 h. The reaction mixture was filtered through a Celite pad and concentrated. The crude product was purified by Sephadex LH-20 (H_2O). Lyophilized compound was poured through a column packed with Dowex 50W X8 Na^+ form resin and eluted with 10 mL of Milli-Q water. Lyophilization then gave the fully deprotected Hib-PRP bears phosphodiester aminopentyl spacer as a white solid.

Compound 6

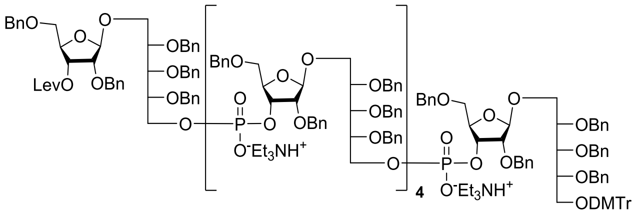

Compound **6** (34 mg, 85%) was prepared as described above. Colorless oil, R_f = 0.20 (DCM/MeOH, 10:1, v/v); ^{1}H NMR (600 MHz, $CDCl_3$) δ 1.02 (t, NCH_2CH_3), 2.11 (s, 3H), 2.52–2.57 (m, 2H), 2.62–2.67 (m, 2H), 2.67 (q, NCH_2CH_3), 3.31 (dd, *J* =10.2, 5.4 Hz, 1H), 3.43 (d, *J* = 8.6 Hz, 1H), 3.42–3.55 (m, 7H), 3.61–3.70 (m, 8H), 3.73 (s, 3H), 3.74 (s, 3H), 3.75-3.78 (m, 4H), 3.79–3.95 (m, 22H), 4.03–4.13 (m, 11H), 4.23–4.44 (m, 23H), 4.45–4.87 (m, 52H), 4.92–5.05 (m, 6H), 5.11 (t, *J* = 5.4 Hz, 1H), 6.67–6.75 (m, 4H), 6.87–7.52 (m, 159H); ^{13}C NMR (151 MHz, $CDCl_3$) δ 8.6 (NCH_2CH_3), 29.8, 29.9, 38.0, 45.3 (NCH_2CH_3), 55.3, 63.4, 63.8, 65.3, 68.1, 68.2, 71.7, 72.3, 72.6, 72.9, 73.2, 73.3, 73.8, 73.9, 74.1, 74.5, 78.5, 78.6, 78.9, 79.1, 80.2, 80.9, 81.6, 82.3, 82.5, 86.1, 106.0, 106.3, 113.1, 127.3, 127.4, 127.6 (2C), 127.8 (2C), 127.9, 128.0, 128.1, 128.3 (2C), 128.4 (2C), 128.5, 130.3, 136.4, 137.9, 138.3, 138.7, 138.9, 139.0, 145.3, 158.4, 172.2, 206.4; ^{31}P NMR (162 MHz, $CDCl_3$) δ 0.01, -0.06, -0.09; MALDI-TOF m/z calcd for $C_{296}H_{316}O_{68}P_5^{3-}$[M+2H]$^{3-}$: 5112.997, Found: 5112.571.

Compound 27

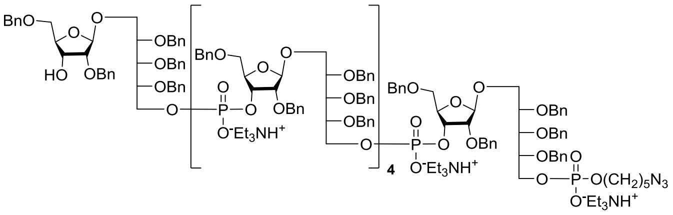

Compound **27** (29 mg, 74% over three steps) was prepared as described above. Colorless oil, R_f = 0.20 (DCM/MeOH, 10:1, v/v); ^{1}H NMR (400 MHz, $CDCl_3$) δ 1.02 (t, NCH_2CH_3), 1.32–1.35 (m, 2H), 1.44–1.56 (m, 4H), 2.68 (q, NCH_2CH_3), 3.12 (t, *J* = 7.0 Hz, 2H), 3.42–3.50 (m, 6H), 3.52–3.58 (m, 2H), 3.63–3.76 (m, 14H), 3.77–3.97 (m, 27H), 4.02 – 4.14 (m, 12H), 4.23–4.45 (m, 22H), 4.46–4.66 (m, 35H), 4.67–4.83 (m, 18H), 4.93–5.03 (m, 6H), 7.05–7.50 (m, 150H); ^{13}C NMR (100 MHz, $CDCl_3$) δ 8.3 (NCH_2CH_3), 23.0, 28.5, 29.7, 32.0, 45.1 (NCH_2CH_3), 51.4, 64.9, 67.5, 67.9, 72.1, 72.2, 72.5, 73.2, 73.8, 74.4, 77.9, 78.3, 78.5, 78.7, 78.9, 81.5, 81.7, 82.2, 82.9, 104.8, 106.2, 127.18, 127.23, 127.29, 127.34, 127.4, 127.6, 127.7, 127.75, 127.79, 137.84, 127.9, 128.0, 128.2 (2C), 128.3, 128.5, 137.3, 138.3, 138.6, 138.7 (2C), 138.8 (2C), 138.9; ^{31}P NMR (162 MHz, $CDCl_3$) δ 0.07, 0.01, -0.08; MALDI-TOF m/z calcd for $C_{275}H_{303}N_3O_{67}P_6^{2-}$[M+4H]$^{2-}$: 4904.883, Found: 4904.113.

Compound 2

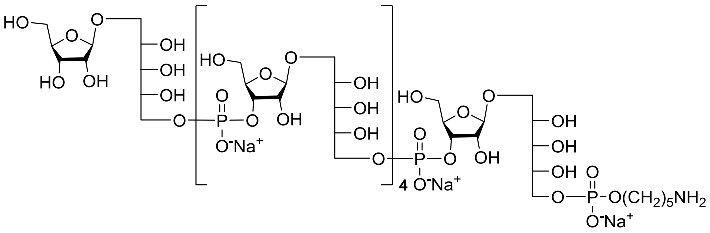

Compound **2** (8 mg, 87%) was prepared as described above. White form, ^{1}H NMR (600 MHz, D_2O) δ 1.46–1.53 (m, 2H), 1.67–1.75 (m, 4H), 3.04 (t, *J* = 7.5 Hz, 2H), 3.65–4.05 (m, 52H), 4.10–4.14 (m, 6H), 4.17–4.23 (m, 5H), 4.24–4.30 (m, 6H), 4.60–4.65 (m, 5H), 5.04 (s, 1H), 5.08 (s, 5H); ^{13}C NMR (151 MHz, D_2O) δ24.6, 28.9, 31.7, 42.0, 65.0, 65.1, 68.4, 69.0, 69.3, 71.1, 71.2, 72.7, 73.2, 73.5, 74.0, 74.5, 76.4, 76.9, 84.5, 85.3, 109.3, 109.5; ^{31}P NMR (243 MHz, D_2O) δ -1.97, -2.80, -2.82; MALDI-TOF m/z calcd for $C_{65}H_{125}N_2Na_3O_{67}P_6^{2-}$:[M+$NH_4$+3Na]$^{2-}$: 2260.4565, Found: 2260.8466, and $C_{65}H_{129}N_3Na_3O_{67}P_6^-$:[M+2$NH_4$+3Na]$^-$: 2278.490, Found: 2278.906.

Compound 7

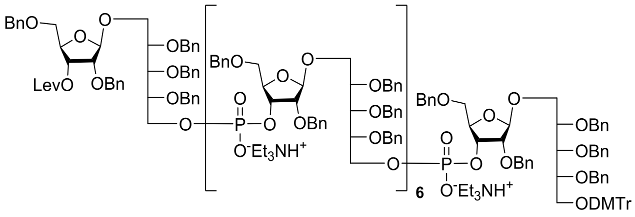

Compound **7** (35 mg, 83%) was prepared as described above. Colorless oil, R_f = 0.20 (DCM/MeOH, 10:1, v/v); ^{1}H NMR (600 MHz, $CDCl_3$) δ 1.02 (t, NCH_2CH_3), 2.12 (s, 3H), 2.55–2.59 (m, 2H), 2.62–2.66 (m, 2H), 2.67 (q, NCH_2CH_3), 3.33 (dd, *J* = 10.2, 5.6 Hz, 2H), 3.37–3.54 (m, 11H), 3.62–3.72 (m, 14H), 3.73 (s, 3H), 3.74 (s, 3H), 3.75-3.77 (m, 4H), 3.79–3.98 (m, 32H), 4.02–4.12 (m, 13H), 4.25–4.43 (m, 30H), 4.44–4.68 (m, 49H), 4.69–4.83 (m, 20H), 4.91–5.05 (m, 7H), 5.12 (t, *J* = 5.3 Hz, 1H), 6.68–6.75 (m, 4H), 7.04–7.47 (m, 209H); ^{13}C NMR (151 MHz, $CDCl_3$) δ 9.5 (NCH_2CH_3), 29.8, 38.0, 45.6 (NCH_2CH_3), 55.2, 63.8, 64.0, 65.2, 67.5, 68.0, 70.7, 71.7, 72.3, 72.6, 72.9, 73.2, 73.3, 73.9, 74.1, 74.5, 78.0, 78.6, 78.9, 80.2, 80.9, 81.6, 82.3, 86.1, 106.0, 106.3, 106.4, 113.1, 127.2, 127.3, 127.4, 127.5, 127.6, 127.7, 127.8, 127.87, 127.91, 127.95, 127.98, 128.2, 128.35, 128.41, 130.3, 136.4, 137.8, 138.2, 138.7, 138.8, 138.9, 139.0, 145.3, 158.4, 172.2, 206.3; ^{31}P NMR (162 MHz, $CDCl_3$) δ -0.02, -0.14. MALDI-TOF m/z calcd for $C_{386}H_{413}O_{90}P_7^{4-}$[M+3H]$^{4-}$: 6704.593, Found: 6704.587.

Compound 28

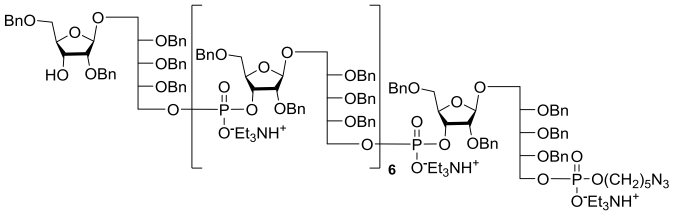

Compound **28** (27 mg, 72% over three steps) was prepared as described above. Colorless oil, R_f = 0.20 (DCM/MeOH, 10:1, v/v); ^{1}H NMR (600 MHz, $CDCl_3$) δ 1.02 (t, NCH_2CH_3), 1.31–1.35 (m, 2H), 1.46–1.56 (m, 4H), 2.70 (q, NCH_2CH_3), 3.12 (t, *J* = 7.0 Hz, 2H), 3.40–3.58 (m, 10H), 3.61–3.75 (m, 18H), 3.76–3.98 (m, 33H), 4.01–4.18 (m, 16H), 4.25–4.45 (m, 30H), 4.46–4.85 (m, 71H), 4.90–5.04 (m, 8H), 7.08–7.36 (m, 200H); ^{13}C NMR (151 MHz, $CDCl_3$) δ 8.4(NCH_2CH_3), 23.1, 28.6, 29.8, 32.0, 45.1 (NCH_2CH_3), 51.4, 65.0, 65.2, 67.8, 72.0, 72.3, 72.6, 73.1, 73.2, 73.8, 73.9, 74.5, 74.6, 78.0, 78.4, 78.5, 78.7, 79.0, 79.86, 81.5, 81.8, 82.2, 82.4, 83.0, 104.8, 106.2, 106.3, 127.2, 127.4, 127.5, 127.6, 127.7, 127.8, 127.9 (2C), 128.1, 128.2, 128.3, 128.4, 128.5, 128.6, 137.4, 138.3 (3C), 138.5, 138.6, 138.7, 138.8 (2C), 138.9, 139.0; ^{31}P NMR (243 MHz, $CDCl_3$) δ 0.71, -0.07, -0.51; MALDI-TOF m/z calcd for $C_{365}H_{407}N_5O_{89}P_8^{2-}$[M+2NH$_4$+4H]$^{2-}$: 6531.539, Found: 6531.232.

Compound 3

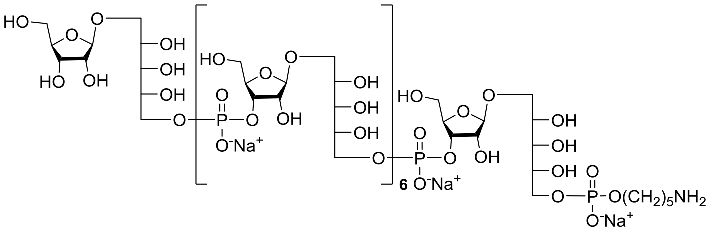

Compound **3** (7 mg, 80%) was prepared as described above. White foam, ^{1}H NMR (600 MHz, D_2O) δ 1.47–1.54 (m, 2H), 1.65–1.76 (m, 4H), 3.04 (t, *J* = 7.5 Hz, 2H), 3.62–4.09 (m, 68H), 4.10–4.15 (m, 8H), 4.16–4.23 (m, 7H), 4.24–4.29 (m, 8H), 4.61–4.67 (m, 7H), 5.04 (s, 1H), 5.08 (s, 7H); ^{13}C NMR (151 MHz, D_2O) δ24.6,28.9, 31.7, 42.0, 64.9, 65.1, 68.4, 69.0, 69.1, 69.27, 69.30, 71.1, 71.2, 72.2, 72.3, 72.7, 72.8, 73.2, 73.5, 73.6 (2C), 73.98, 74.01, 74.5, 74.8, 76.4, 76.9 (2C), 77.1, 84.6, 85.3, 109.3, 109.5; ^{31}P NMR (243 MHz, D_2O) δ 1.02, 0.18, 0.16; MALDI-TOF m/z calcd for $C_{85}H_{175}N_5O_{89}P_8{}^{2-}$[M+4NH4+2H]$^{2-}$: 2937.7233, Found: 2937.4369, and $C_{85}H_{177}N_6O_{89}P_8{}^{3-}$[M+5NH4]$^{3-}$: 2953.742, Found: 2953.471.

Compound 8

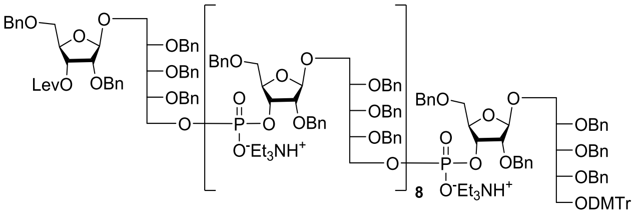

Compound **8** (34 mg, 80%) was prepared as described above. Colorless oil, R_f= 0.20 (DCM/MeOH, 10:1, v/v); ^{1}H NMR (600 MHz, $CDCl_3$) δ 1.00 (t, NCH_2CH_3), 2.12 (s, 3H), 2.53–2.58 (m, 2H), 2.62–2.64 (m, 2H), 2.66 (q, NCH_2CH_3), 3.32 (dd, *J* = 10.2, 5.6 Hz, 2H),3.40(d, *J* = 9.4 Hz, 2H), 3.37–3.54 (m, 10H), 3.61–3.70 (m, 12H), 3.73 (s, 3H), 3.74 (s, 3H), 3.75–3.77 (m, 2H), 3.79–3.97 (m, 41H), 4.03–4.14 (m, 25H), 4.15–4.19 (m, 2H), 4.25–4.45 (m, 38), 4.46–4.68 (m, 61H), 4.68–4.83 (m, 24H), 4.93–5.03 (m, 10H), 5.11 (t, *J* = 5.3 Hz, 1H), 6.68–6.75 (m, 4H), 7.05–7.45 (m, 259H); ^{13}C NMR (151 MHz, $CDCl_3$) δ 8.8 (NCH_2CH_3), 27.4, 29.9, 38.0, 45.3 (NCH_2CH_3), 55.3, 63.3, 63.4, 63.8, 65.0, 65.3, 68.0, 68.2, 72.3, 72.6, 73.2, 73.3, 73.9 (2C), 78.6, 78.7, 78.9, 79.1, 79.2, 81.6, 82.3, 86.1, 106.0, 106.3, 106.4, 113.1, 127.4, 127.6, 127.7, 127.8, 128.0, 128.3 (2C), 130.3, 136.5, 138.7, 138.9, 139.97, 139.04, 145.4, 158.4, 172.2, 206.3; ^{31}P NMR (243 MHz, D_2O) δ -3.10, -3.52; MALDI-TOF m/z calcd for $C_{476}H_{514}O_{112}P_9{}^{-}$[M+9H]$^{-}$: 8300.271, Found: 8301.862.

Compound 29

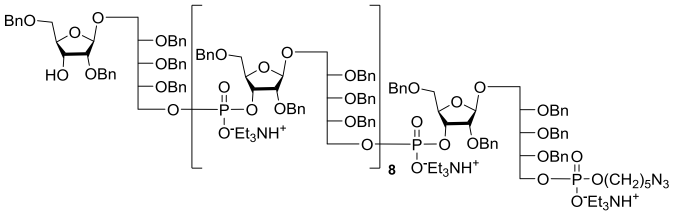

Compound **29** (27 mg, 72% over three steps) was prepared as described above. Colorless oil, R_f = 0.20 (DCM/MeOH, 10:1, v/v); ^{1}H NMR (600 MHz, $CDCl_3$) δ 1.02 (t, NCH_2CH_3), 1.33–1.36 (m, 2H), 1.42–1.58 (m, 4H), 2.72 (q, NCH_2CH_3), 3.12 (t, *J* = 7.0 Hz, 2H), 3.43–3.56 (m, 18H), 3.58–3.78 (m, 29H), 3.79–3.99 (m, 45H), 4.01–4.18 (m, 24H), 4.21–4.46 (m, 43H), 4.47–4.83 (m, 63H), 4.93–5.03 (m, 10H), 7.01–7.44 (m, 250H); ^{13}C NMR (151 MHz, $CDCl_3$) δ 8.4 (NCH_2CH_3), 22.8, 28.6, 29.8, 32.0, 45.2 (NCH_2CH_3), 51.4, 64.9 (2C), 65.1, 65.2, 67.5, 68.0, 70.6 (2C), 72.1, 72.2, 72.3, 72.4, 72.6, 73.2 (2C), 73.8, 73.9, 74.4, 74.5 (2C), 74.6, 77.4, 78.4, 78.5, 78.7, 78.8, 78.9, 81.4, 81.5, 81.8, 82.2, 82.4, 83.0, 104.8, 106.2, 106.3, 127.3 (2C), 127.4, 127.5, 127.6, 127.7 (2C), 127.8, 127.9 (2C), 128.1, 128.2, 128.3, 128.6, 138.6, 138.7, 138.8, 138.9 (3C); ^{31}P NMR (243 MHz, $CDCl_3$) δ 0.69, -0.01, -0.09; MALDI-TOF m/z calcd for $C_{455}H_{495}N_3Na_3O_{111}P_{10}^{3-}$[M+3Na+4H]$^-$: 8155.027, Found: 8155.613.

Compound 4

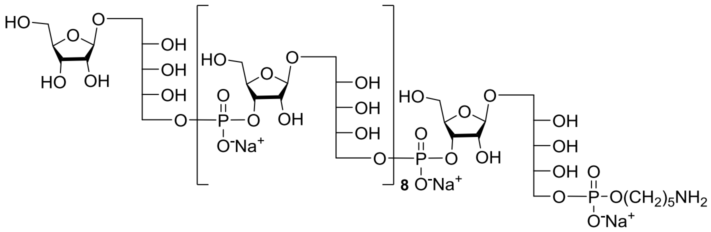

Compound **4** (6 mg,79%) was prepared as described above. White foam, ^{1}H NMR (600 MHz, D_2O) δ 1.47–1.52 (m, 2H), 1.63–1.76 (m, 4H), 3.04 (t, *J* = 7.5 Hz, 2H), 3.60–4.09 (m, 84H), 4.10–4.14 (m, 10H), 4.17–4.23 (m, 9H), 4.24–4.28 (m, 10H), 4.58–4.70 (m, 9H), 5.04 (s, 1H), 5.08 (s, 9H); ^{13}C NMR (151 MHz, D_2O) δ 24.6, 28.9, 31.7, 42.0, 65.0, 65.1, 68.4, 69.0, 69.27, 69.31, 71.1, 71.2, 72.7, 72.8, 73.2, 73.4, 73.5, 73.6 (2C), 74.0 (2C), 76.4, 76.9 (2C), 84.6 (2C), 85.3, 109.3, 109.5; ^{31}P NMR (243 MHz, D_2O) δ 0.97, 0.12, 0.13; MALDI-TOF m/z calcd for $C_{105}H_{198}N_2O_{111}P_{10}^{8-}$[M+NH$_4$+H]$^{8-}$: 3573.7330, Found: 3573.0110, and $C_{105}H_{205}N_2O_{111}P_{10}^{4-}$[M+2NH$_4$+4H]$^{4-}$: 3593.788, Found: 3593.011.

Compound S4

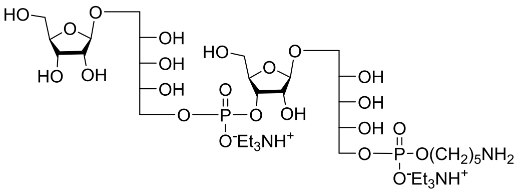

White foam, ^{1}H NMR (600 MHz, D_2O) δ 1.25 (t, NCH_2CH_3), 1.44–1.50 (m, 2H), 1.60–1.73 (m, 4H), 2.62–2.72 (m, 1H), 2.81–2.95 (m, 1H), 3.19 (q, NCH_2CH_3), 3.69–4.02 (m, 22H), 4.04–4.09 (m, 2H), 4.12–4.17 (m, 1H), 4.20–4.25 (m, 2H), 4.56–4.62 (m, 1H), 5.00 (s, 1H), 5.03 (s, 1H); ^{31}P NMR (243 MHz, D_2O) δ 0.93, 0.08.

General Procedure for the Preparation of Oligosaccharide Thiols 1-SH, 2-SH, 3-SH, and 4-SH for Protein Conjugation

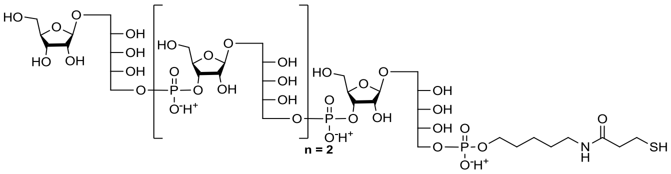

Compounds **1-4** (1 mg, 0.6μmol) were individually dissolved in pH 7.4 PBS buffer (1 mL). A dithiobis(succinimidylpropionate) (DSP) solution (0.3 mL) made from DSP (1.3 mg, 0.010 mmol) in DMSO (1 mL) was added to a solution of **1**, **2**, **3**, or **4** in pH 7.4 PBS buffer and incubated at room temperature overnight. Subsequently, dithiothreitol (DTT) (0.47 mg, 3.1μmol) was added to and the solution stirred at 40 °C for two additional hours. The corresponding compounds **1-SH**, **2-SH**, **3-SH**, and **4-SH** were purified by means of Sephadex LH-20 column chromatography (H_2O).

Physical data for compound **1-SH** as an example: White foam, ^{1}H NMR (600 MHz, D_2O) δ 1.10–1.20 (m, 2H), 1.21–1.25 (m, 1H), 1.33–1.40 (m, 1H), 1.45–1.55 (m, 2H), 1.57–1.68 (m, 2H), 2.57–2.65 (m, 1H), 2.91 (t, J = 5.95 Hz, 1H), 3.16 (t, J = 5.97 Hz, 2H), 3.40–3.77 (m, 13H), 3.79–4.03 (m, 10H), 4.01–4.07 (m, 4H), 4.10–4.15 (m, 3H), 4.17–4.25 (m, 4H), 4.52–4.60 (m, 3H), 4.98 (s, 1H), 5.01 (s, 3H), 7.40 (brs, 1H); ^{31}P NMR (243 MHz, D_2O) δ 0.99, 0.13; MALDI-TOF m/z calcd for $C_{52}H_{93}NO_{48}P_4^-$ $[M+3H]^-$: 1637.385, Found: 1637.950.

Compound S1[1,2]

^{1}H NMR (400 MHz, $CDCl_3$)

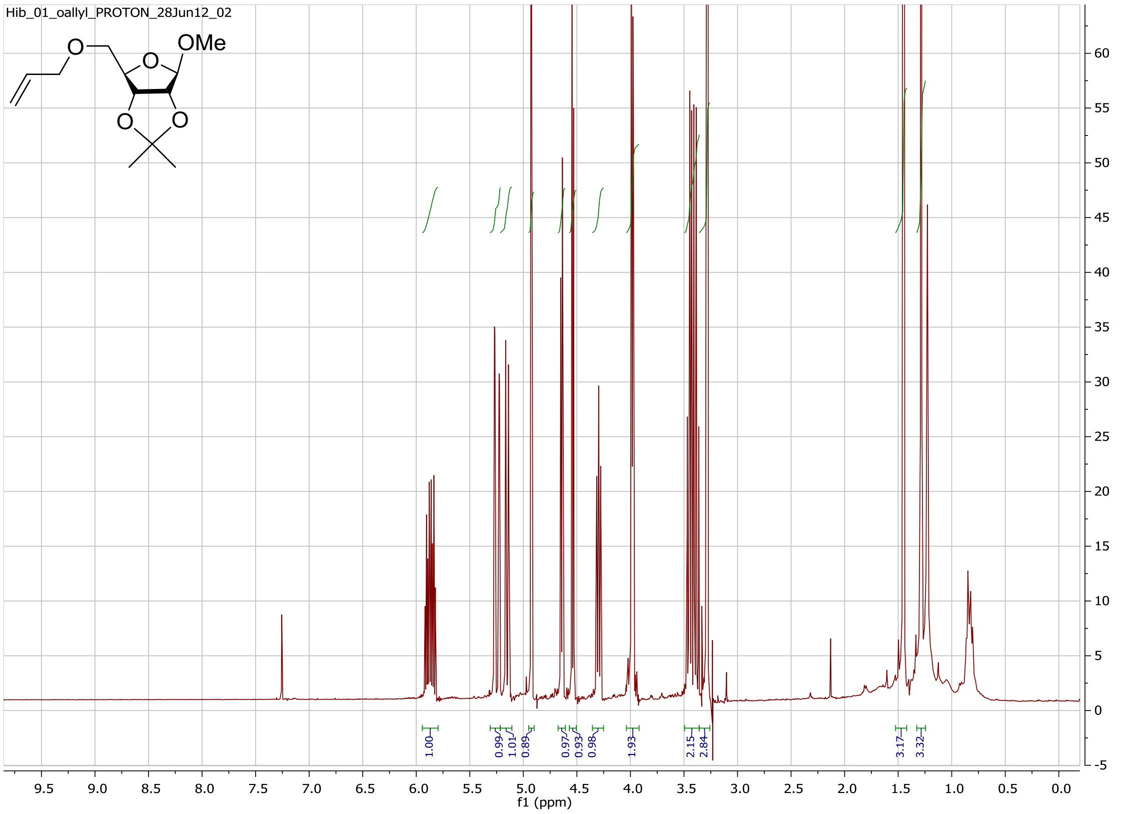

^{13}C NMR (400 MHz, $CDCl_3$)

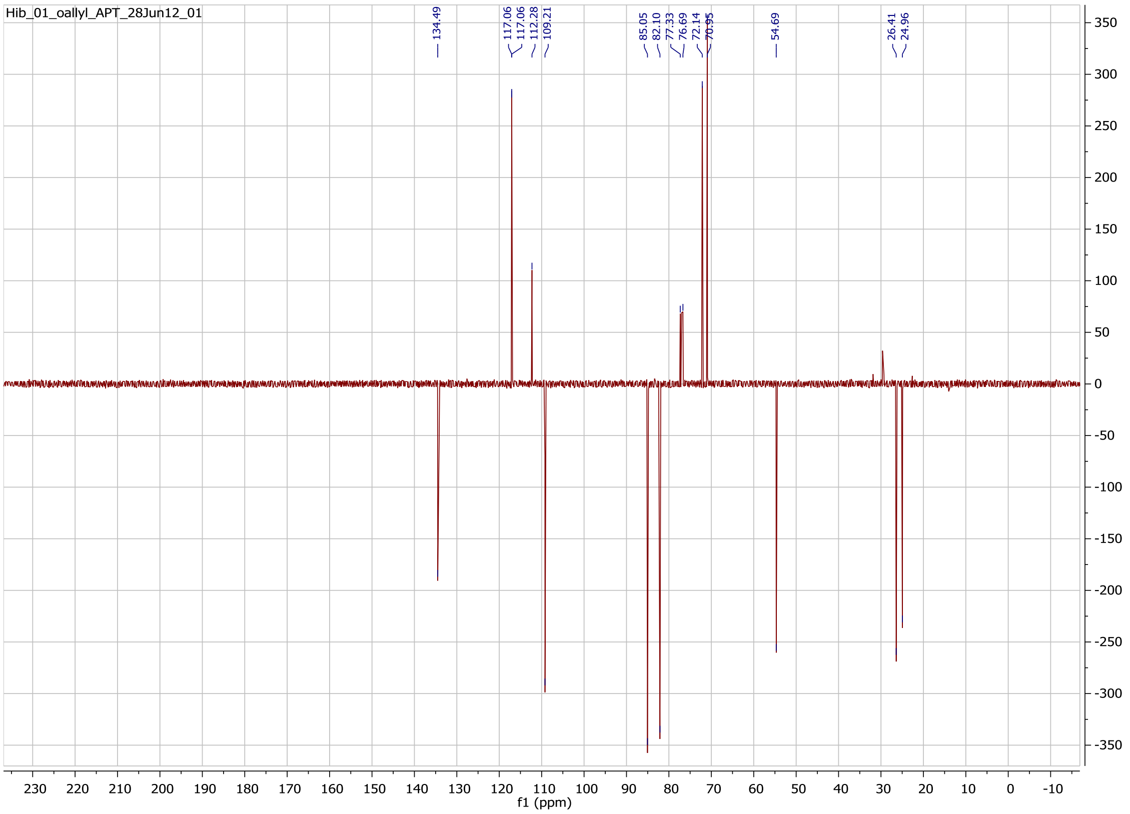

HSQC (400 MHz, $CDCl_3$)

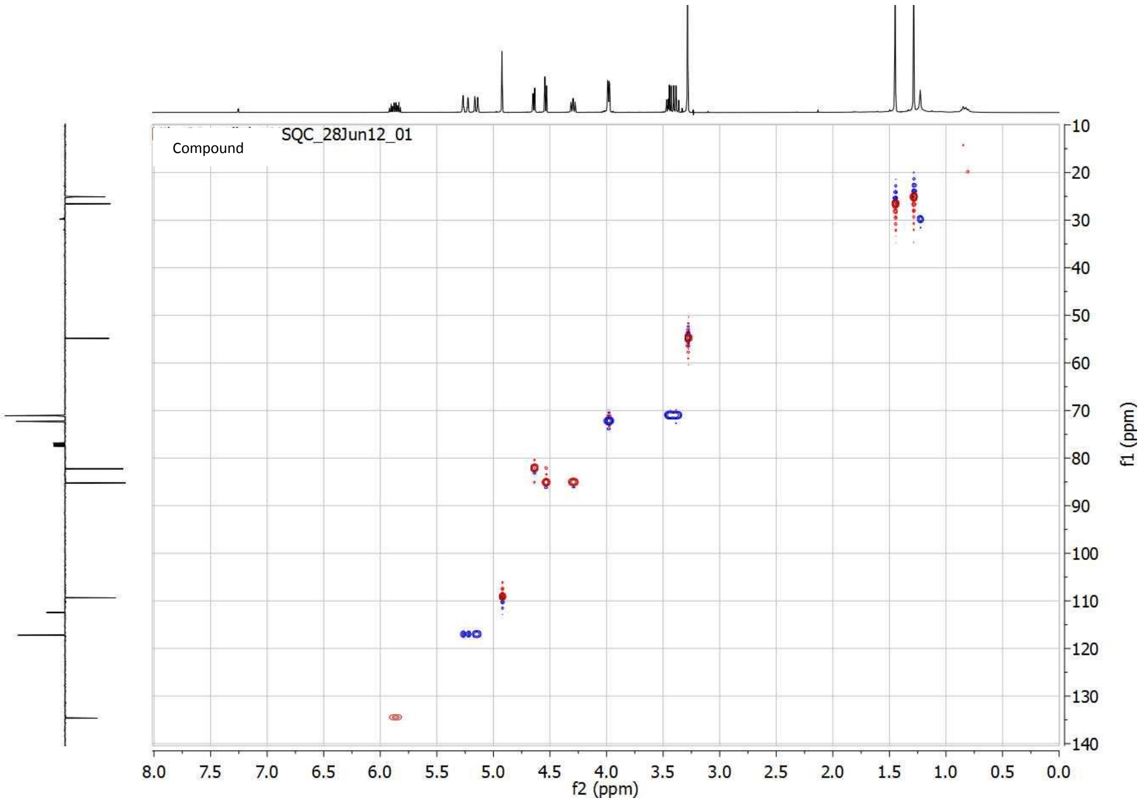

Compound S2[1,2]

^1H NMR (400 MHz, $CDCl_3$)

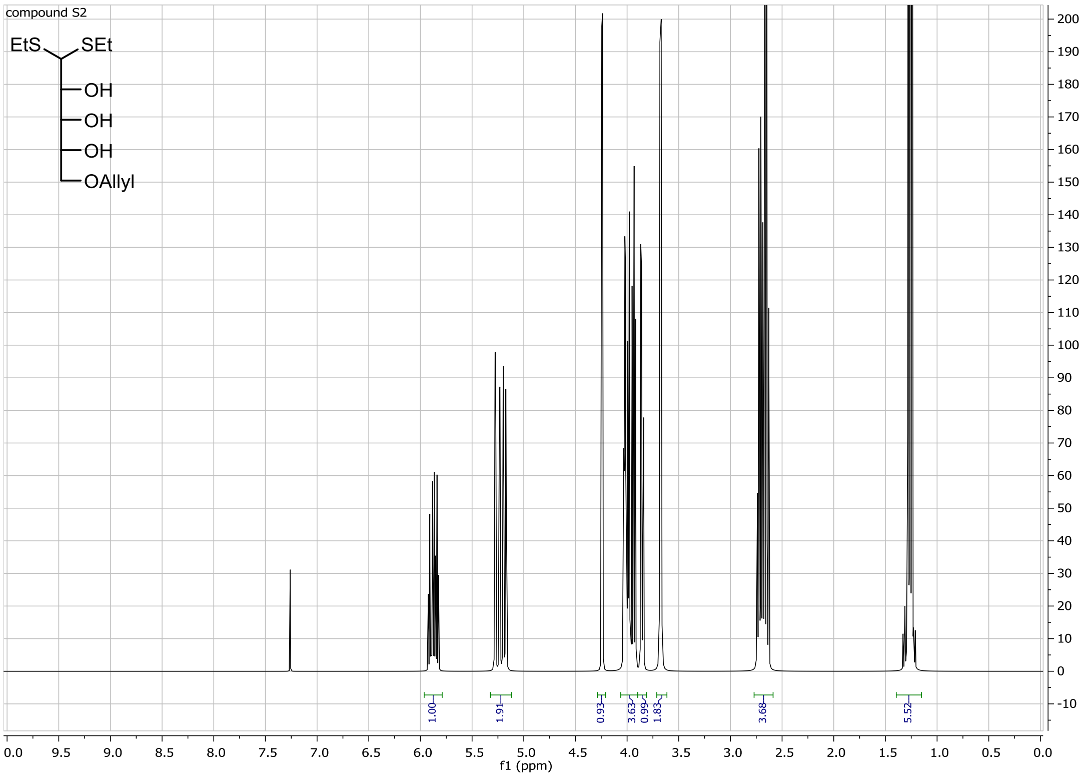

^{13}C NMR(101 MHz, $CDCl_3$)

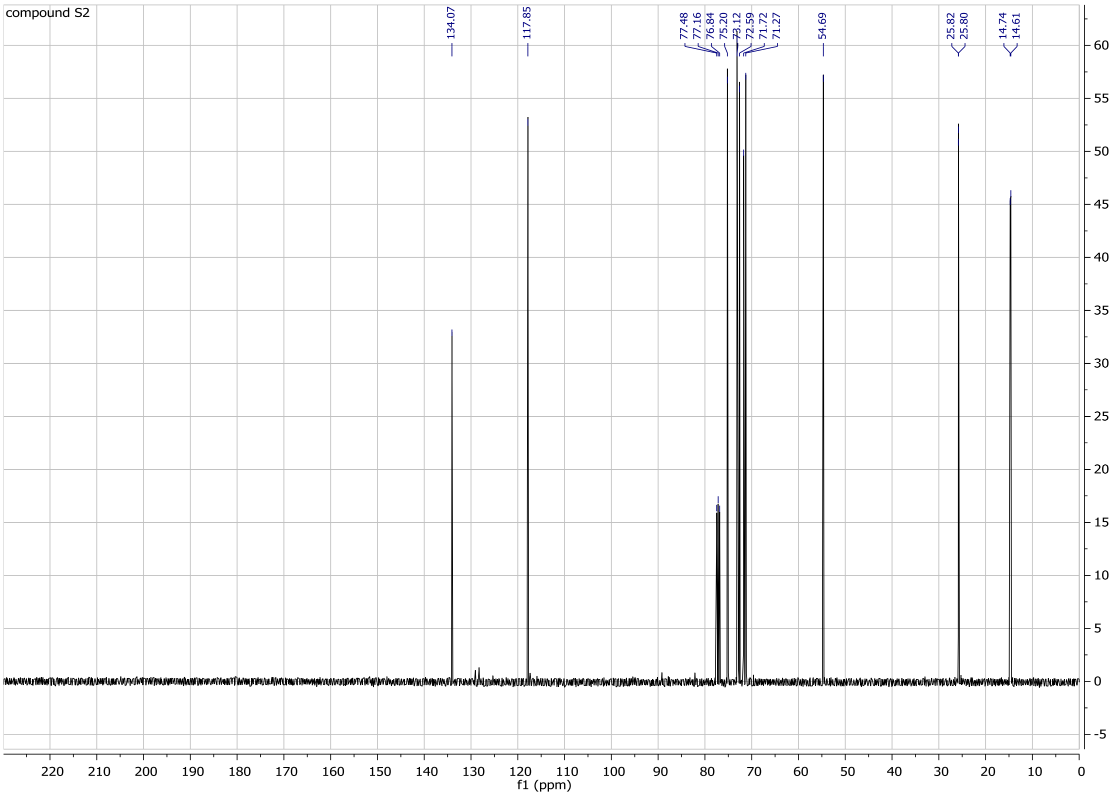

Compound 21[1,2]

^{1}H NMR (400 MHz, $CDCl_3$)

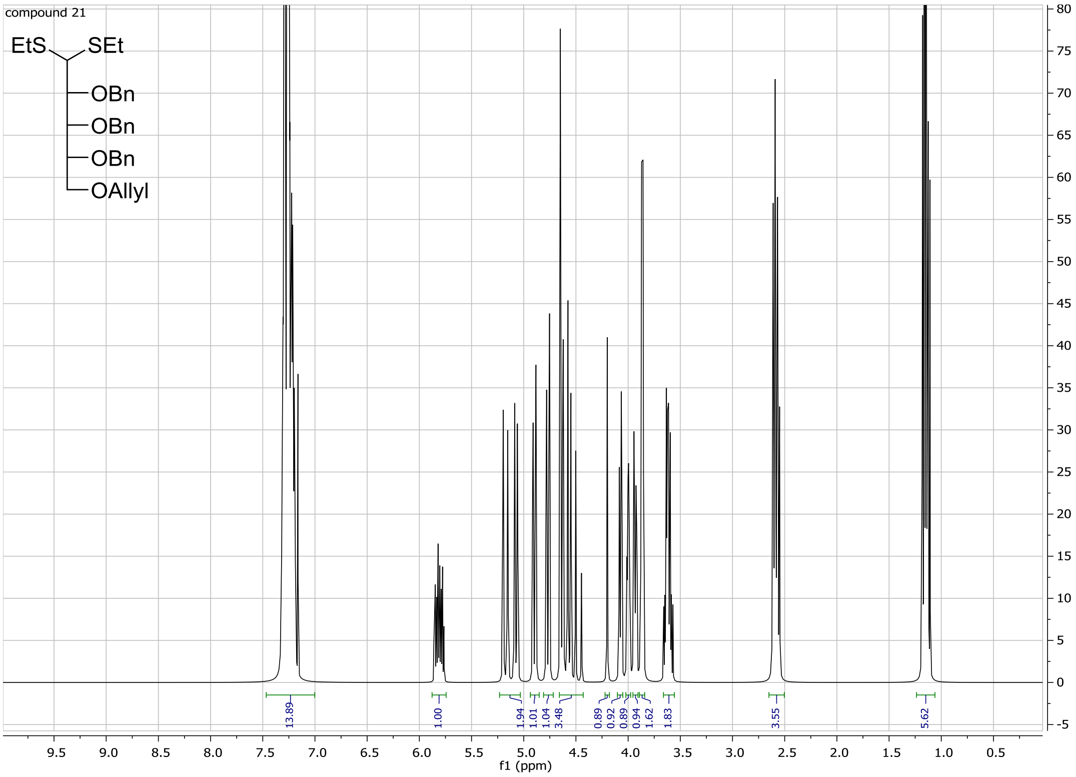

^{13}C NMR(101 MHz, $CDCl_3$)

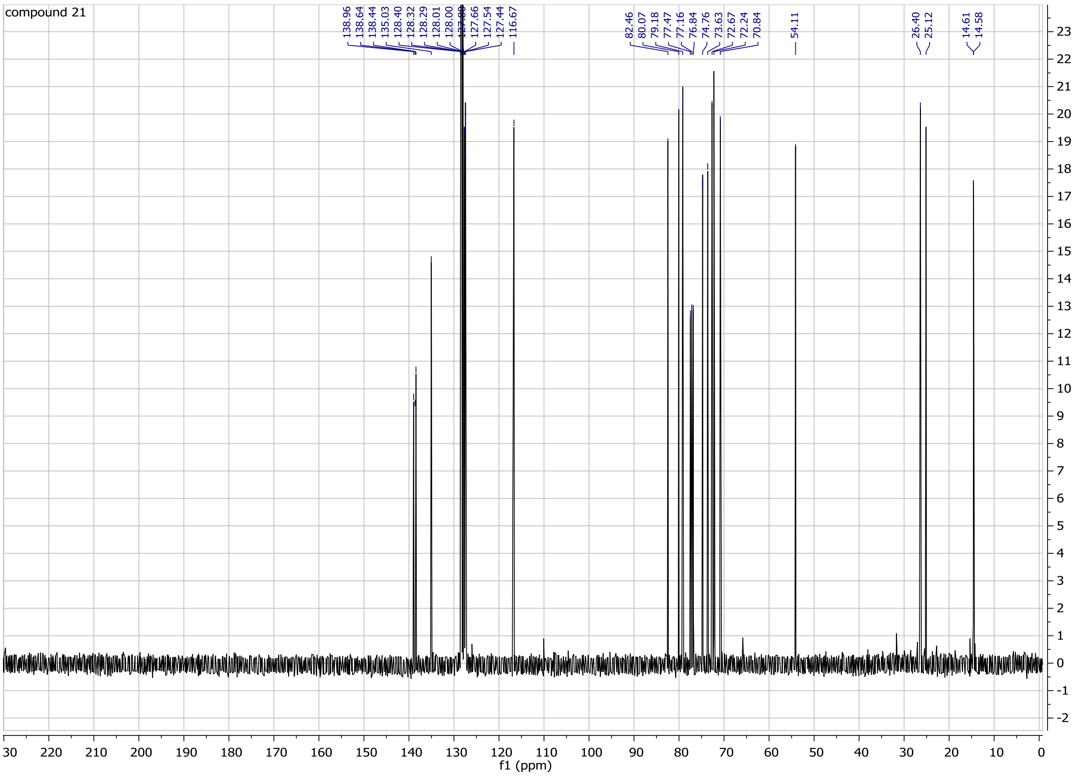

Compound 18[1,2]

^{1}H NMR (400 MHz, $CDCl_3$)

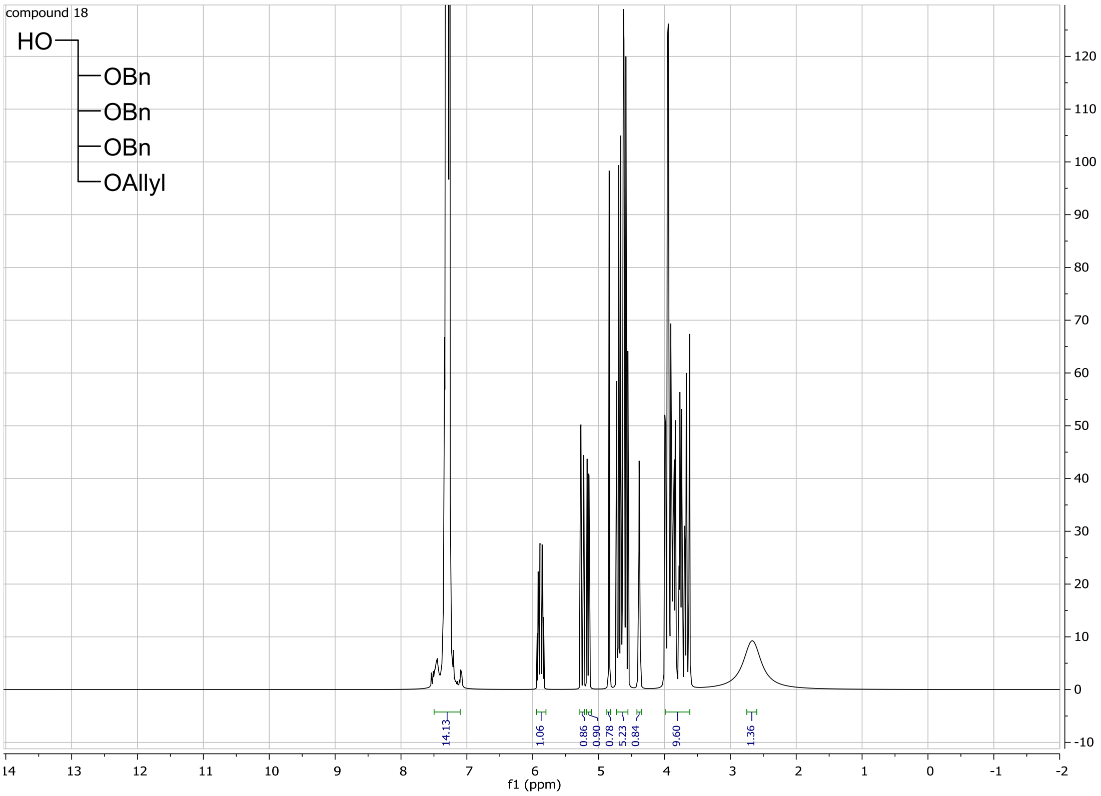

^{13}C NMR(101 MHz, $CDCl_3$)

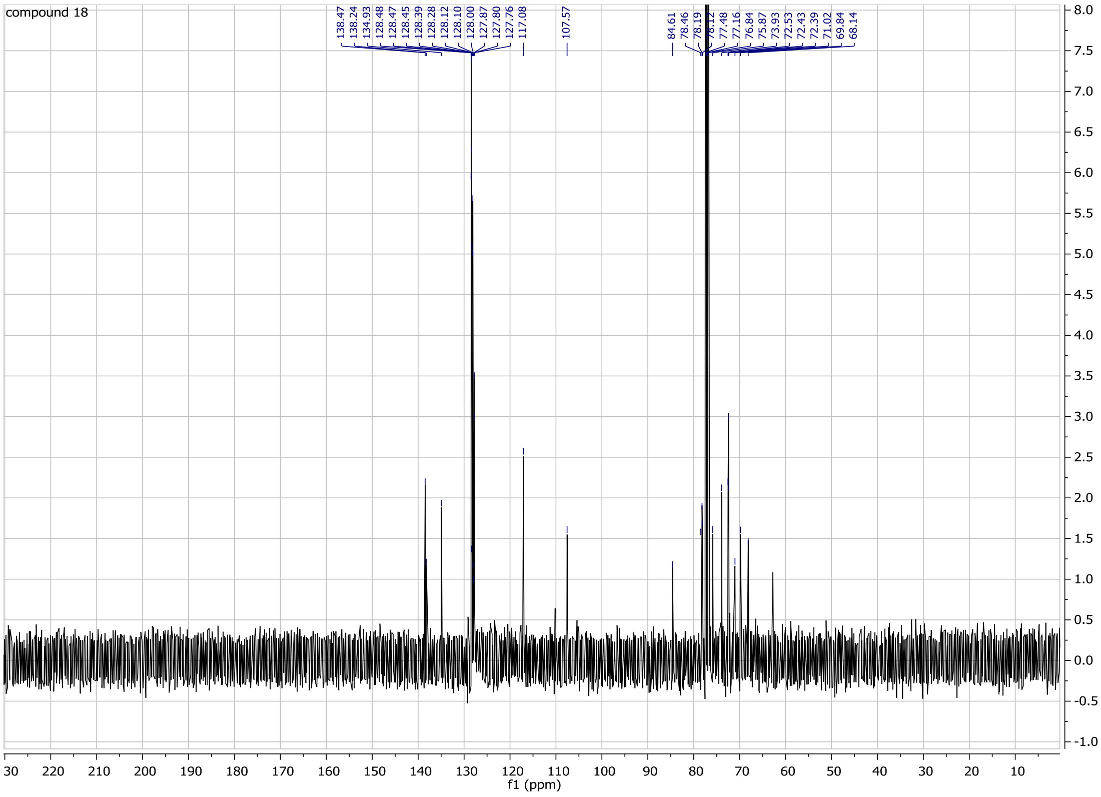

Compound 17[3,4]

^{1}H NMR (400 MHz, $CDCl_3$)

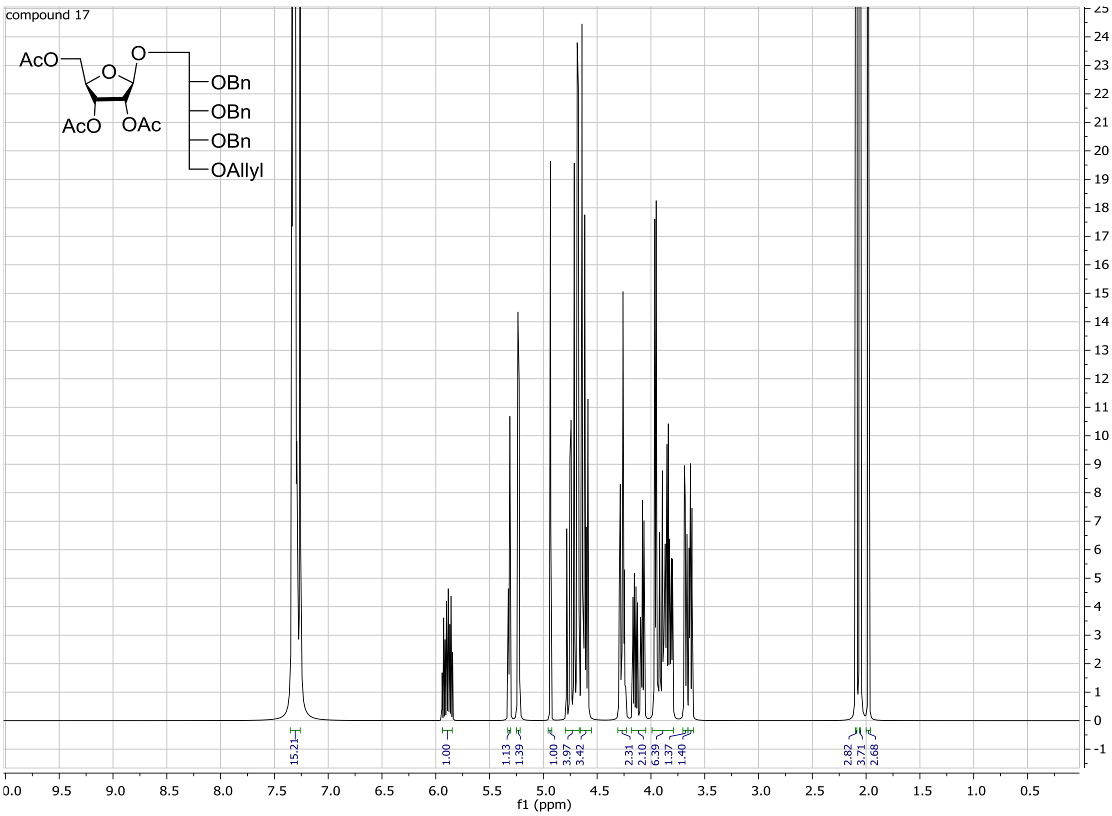

^{13}C NMR(101 MHz, $CDCl_3$)

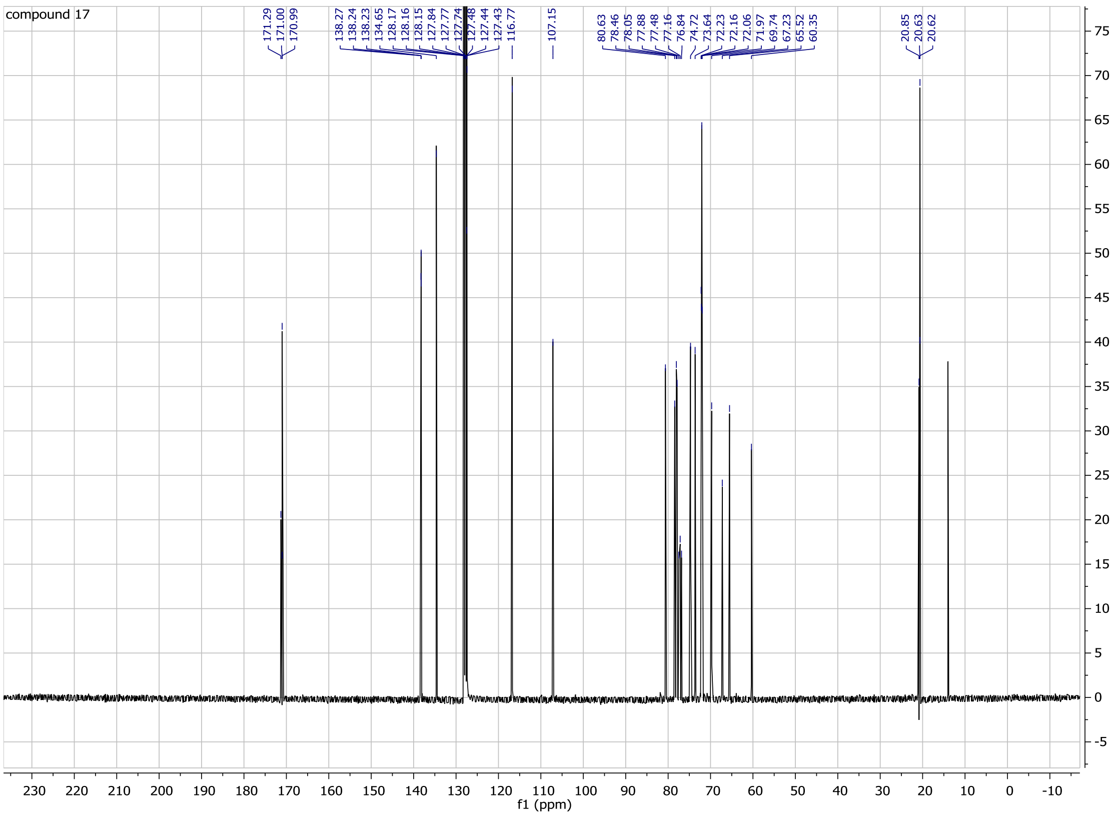

Compound 22[3]

^{1}H NMR (400 MHz, $CDCl_3$)

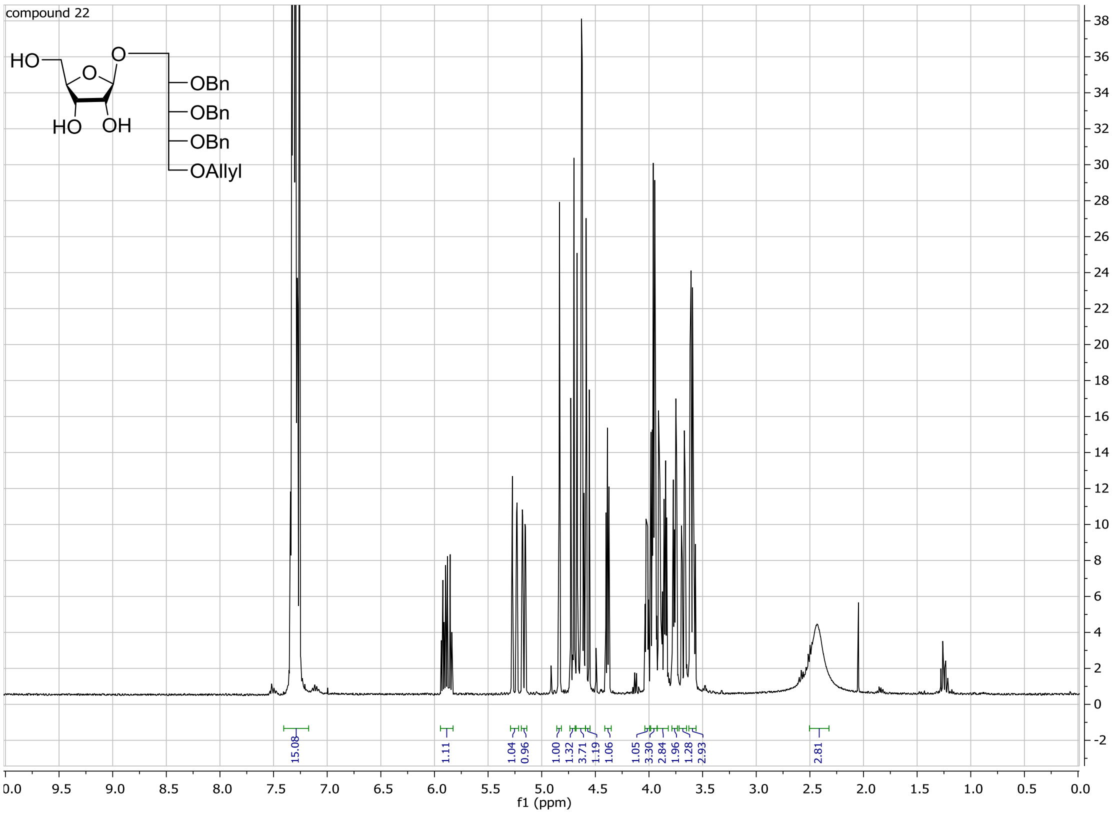

^{13}C NMR(100 MHz, $CDCl_3$)

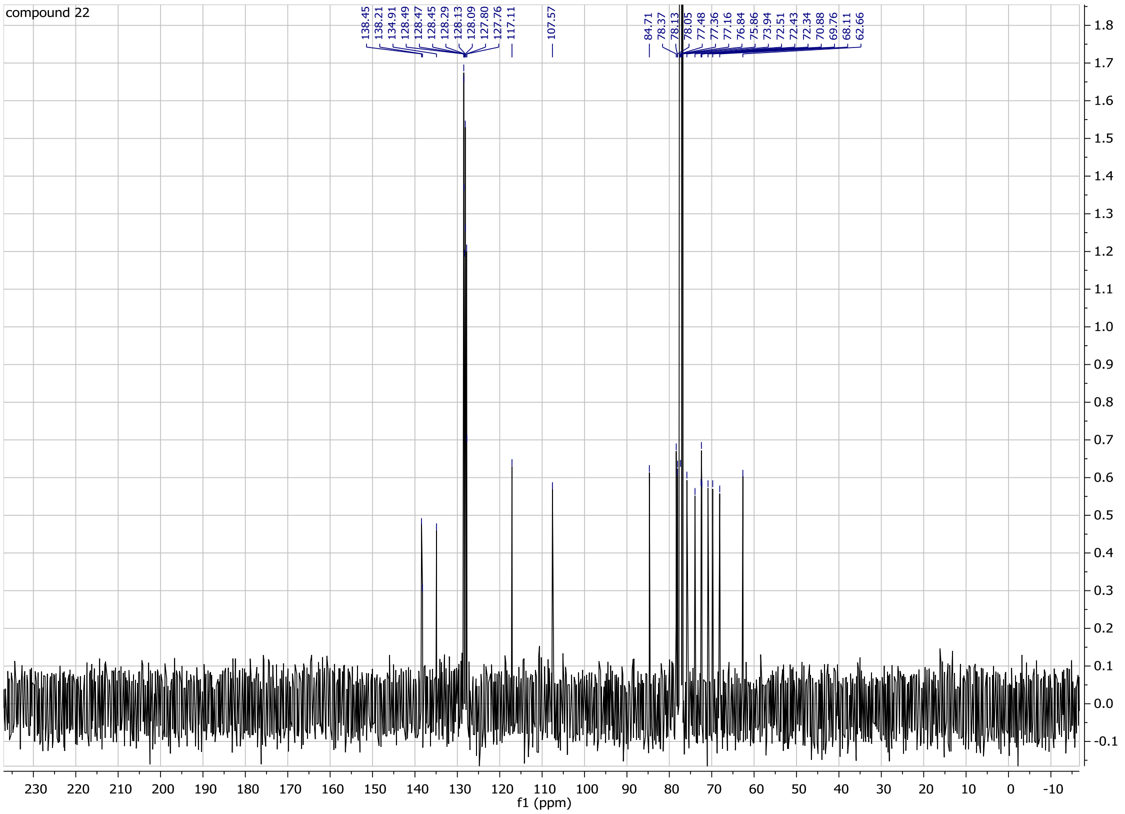

Compound 13[4]

^{1}H NMR (400 MHz, $CDCl_3$)

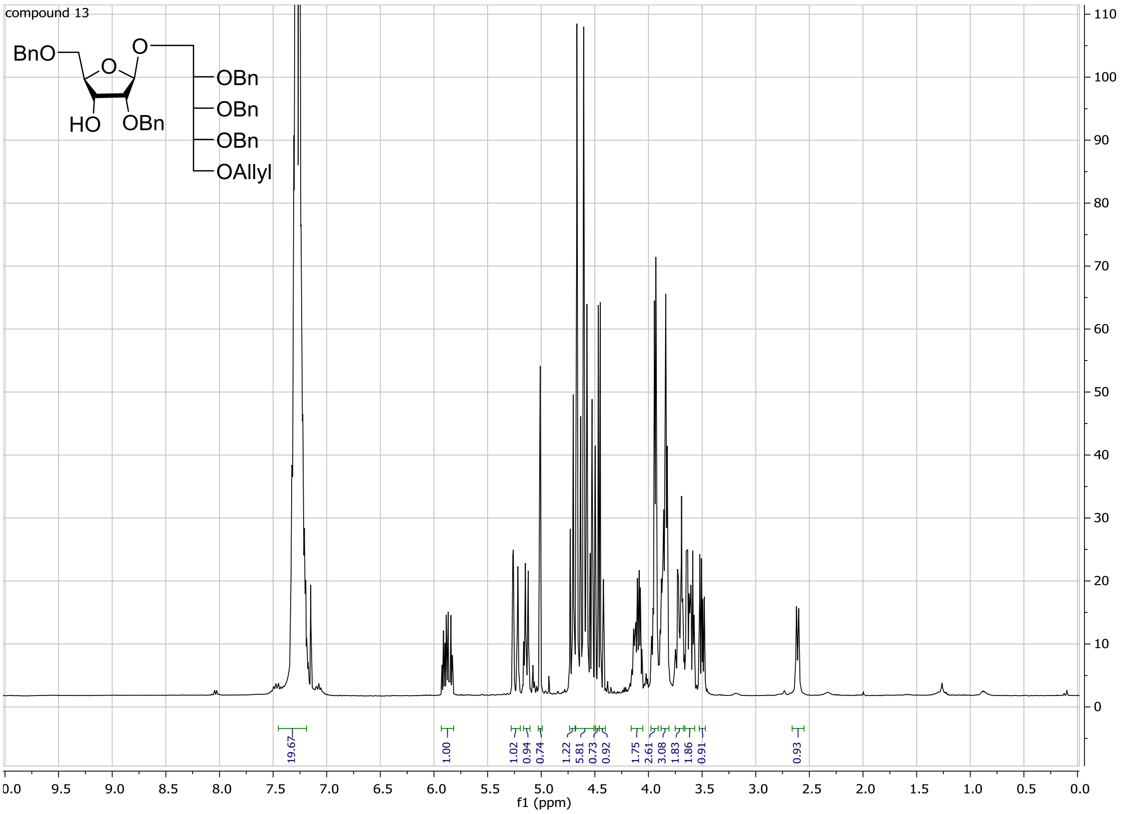

^{13}C NMR(100 MHz, $CDCl_3$)

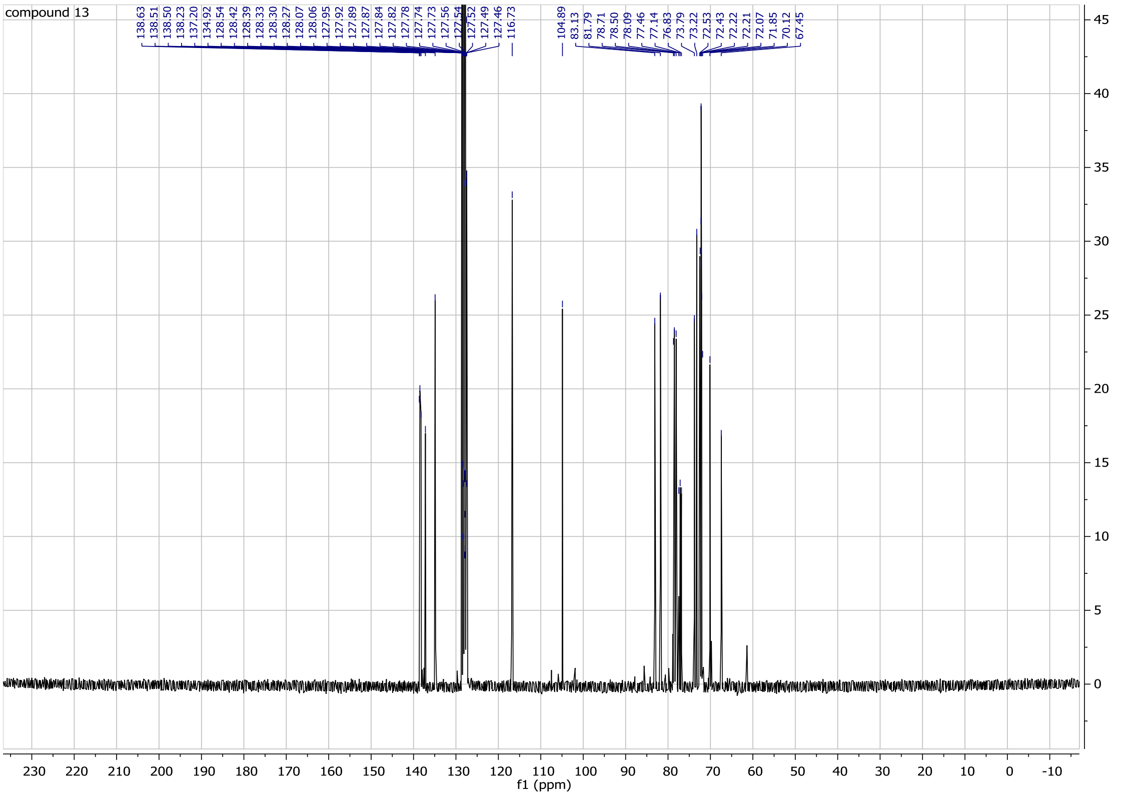

Compound 23[4]: ^{1}H NMR (400 MHz, $CDCl_3$)

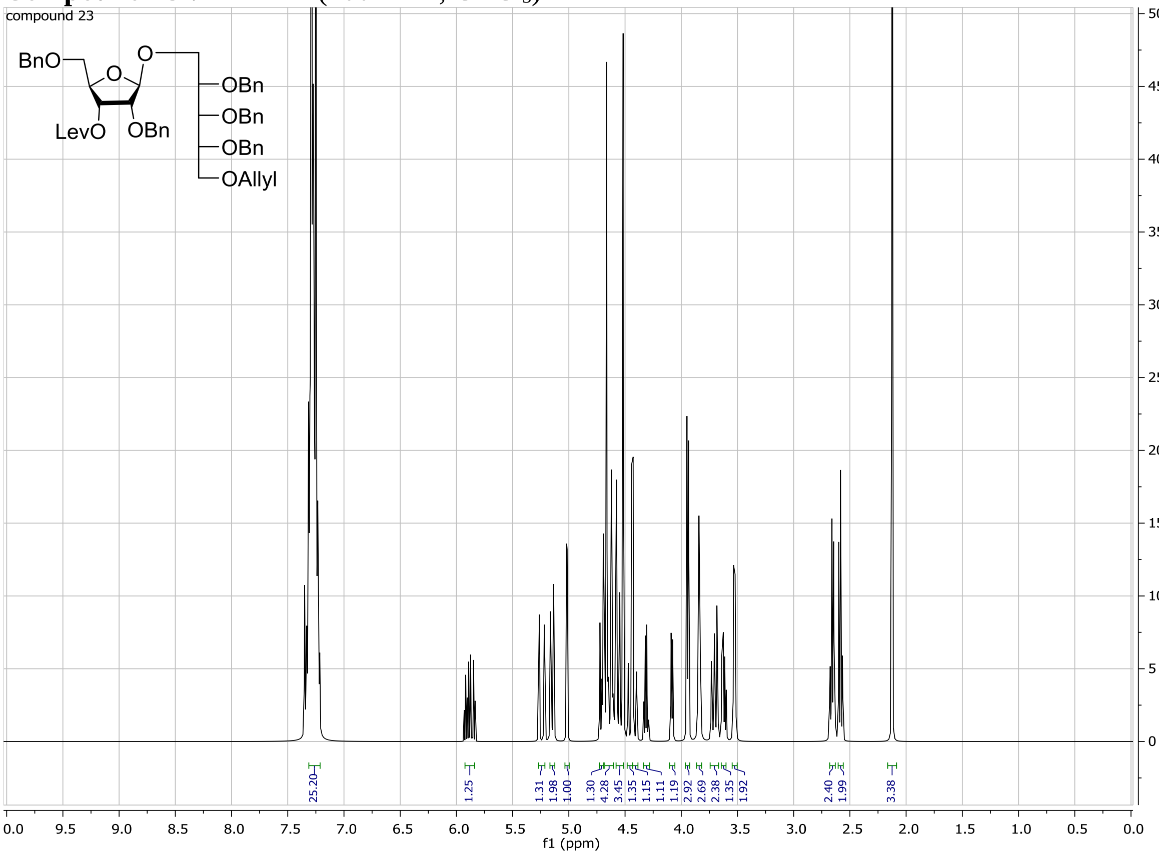

^{13}C NMR(101 MHz, $CDCl_3$)

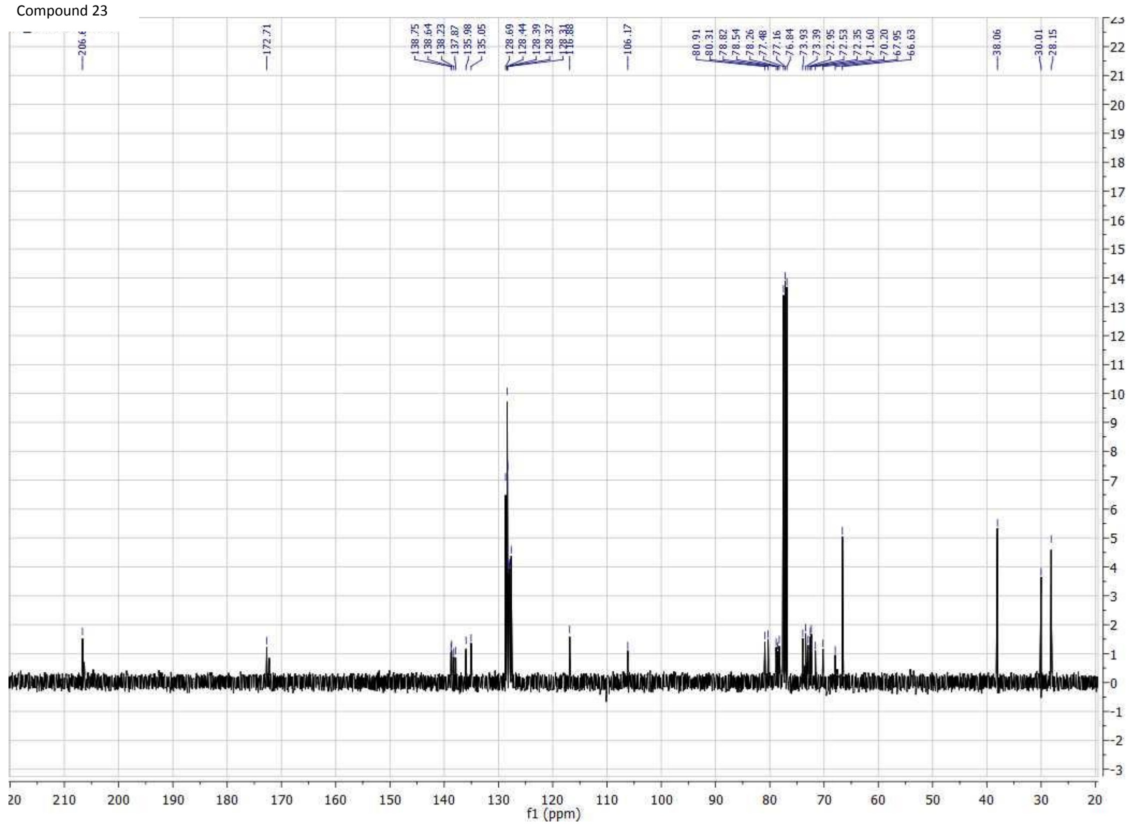

Compound 14[1,2]

^{1}H NMR (400 MHz, $CDCl_3$)

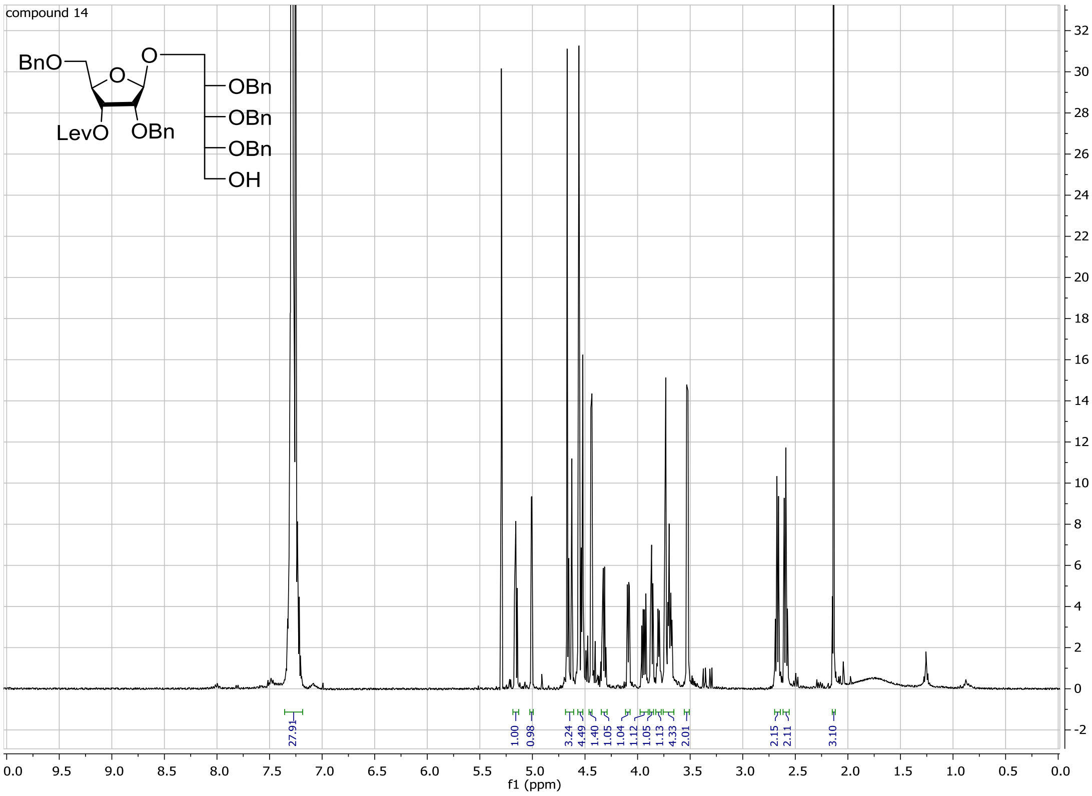

^{13}C NMR(101 MHz, $CDCl_3$)

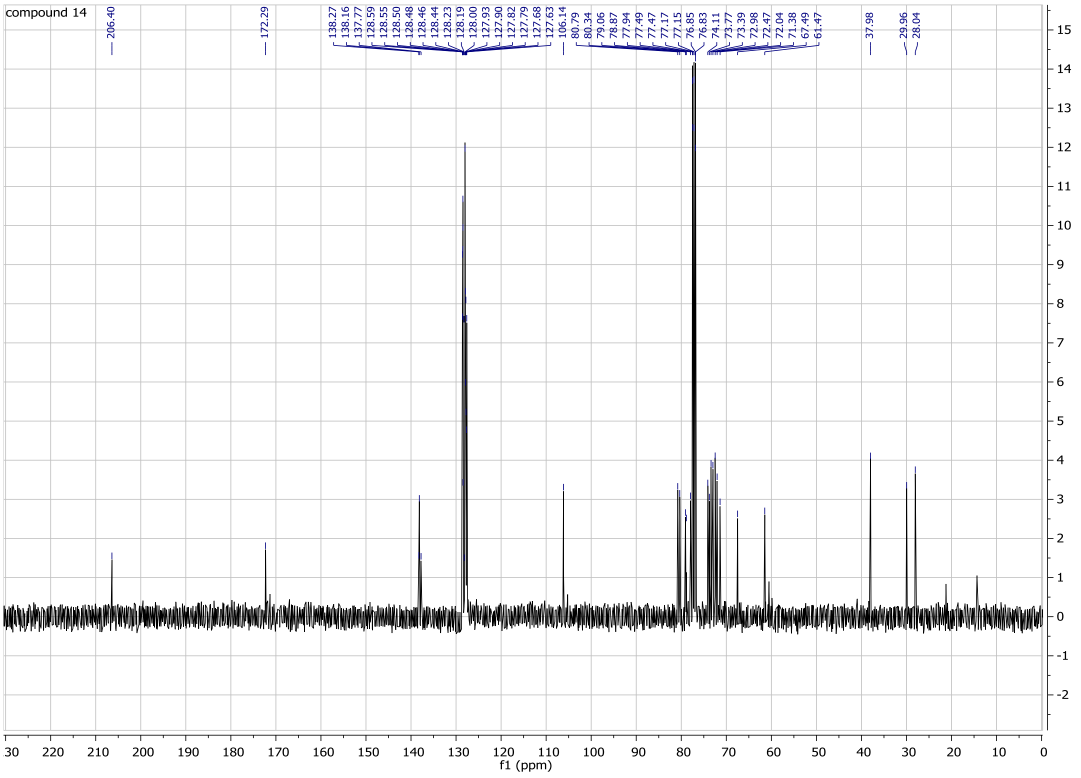

Compound 24[1,2]

^{1}H NMR (400 MHz, $CDCl_3$)

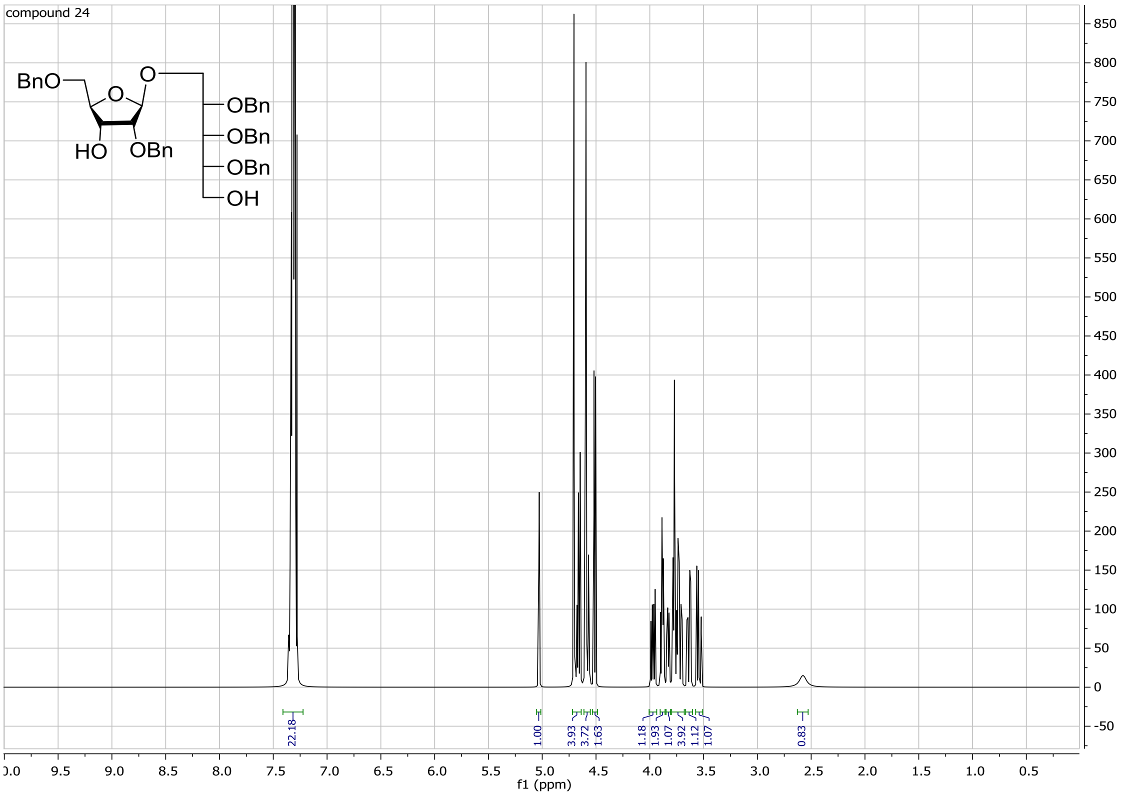

^{13}C NMR(100 MHz, $CDCl_3$)

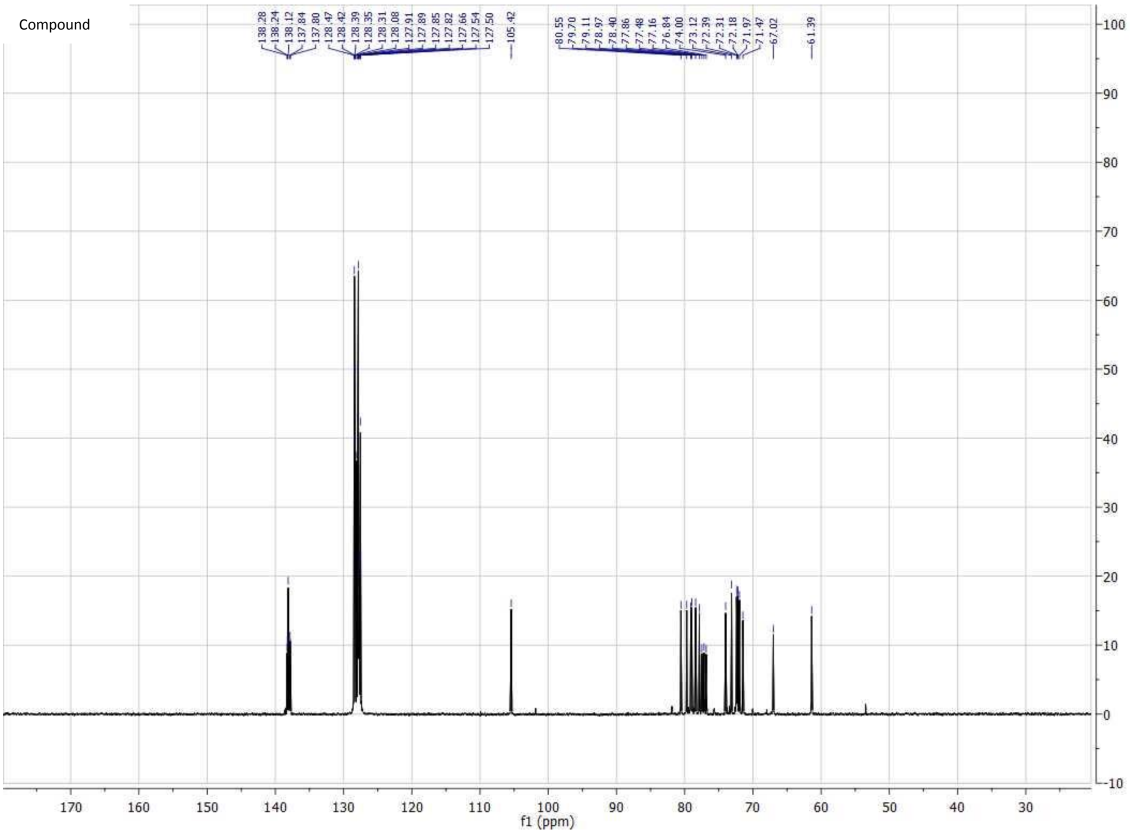

Compound 15[1,2]

^{1}H NMR (400 MHz, $CDCl_3$)

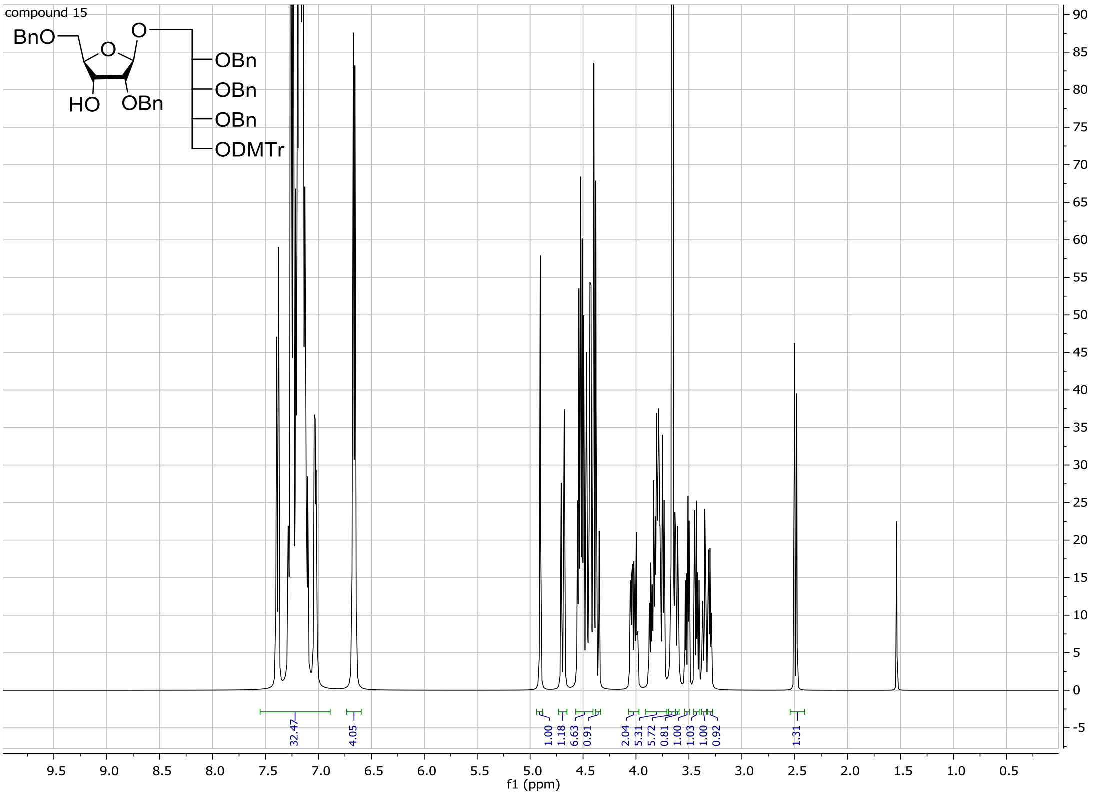

^{13}C NMR(100 MHz, $CDCl_3$)

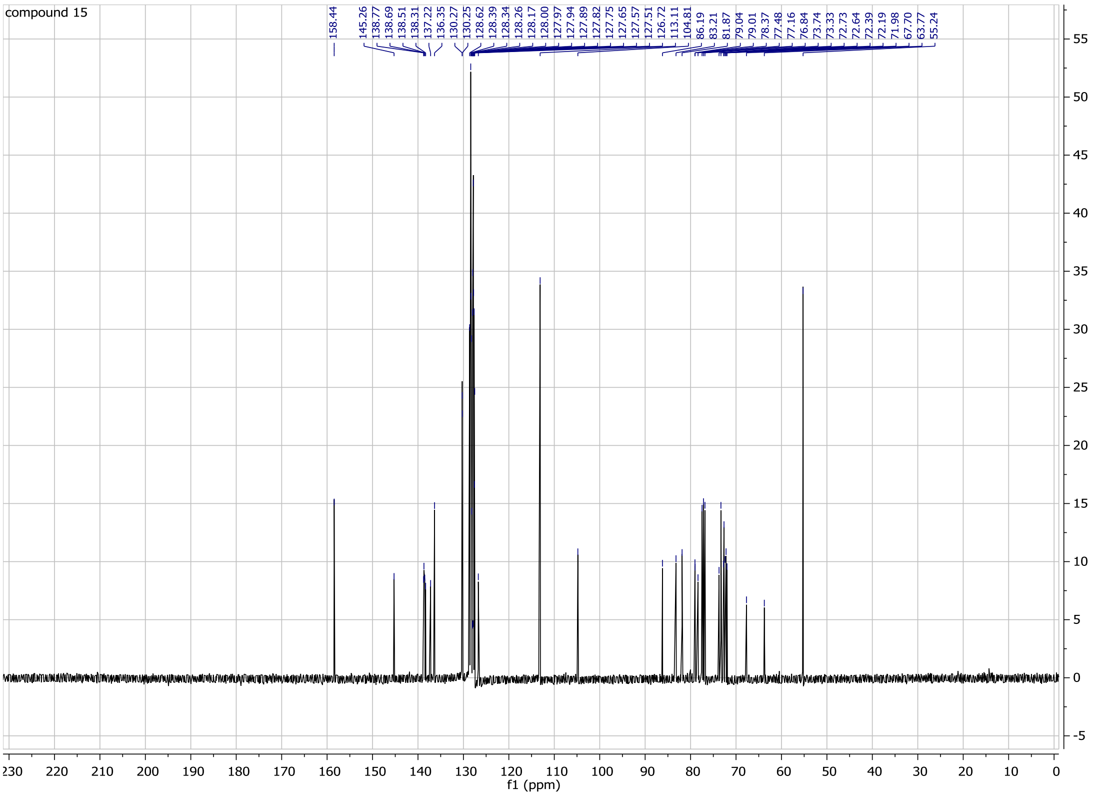

Compound 16[4]

^{1}H NMR (400 MHz, $CDCl_3$)

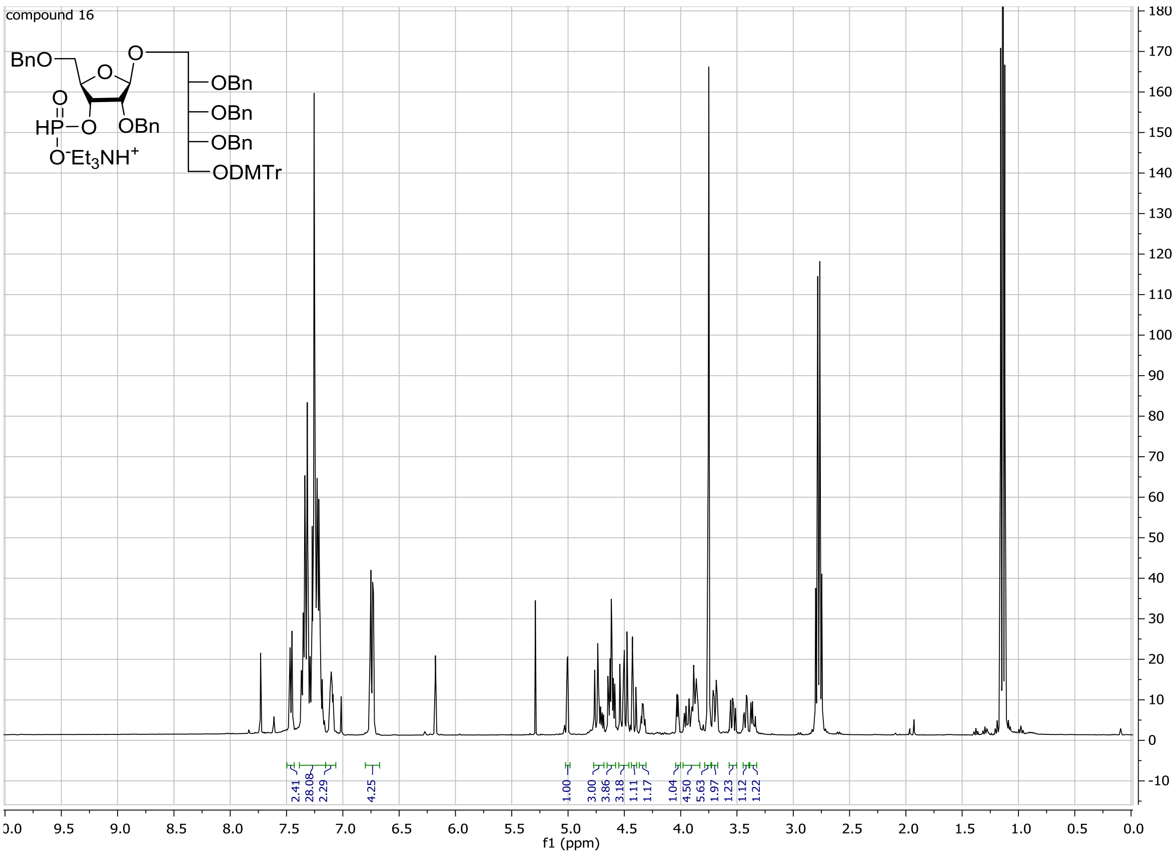

^{13}C NMR (101 MHz, $CDCl_3$)

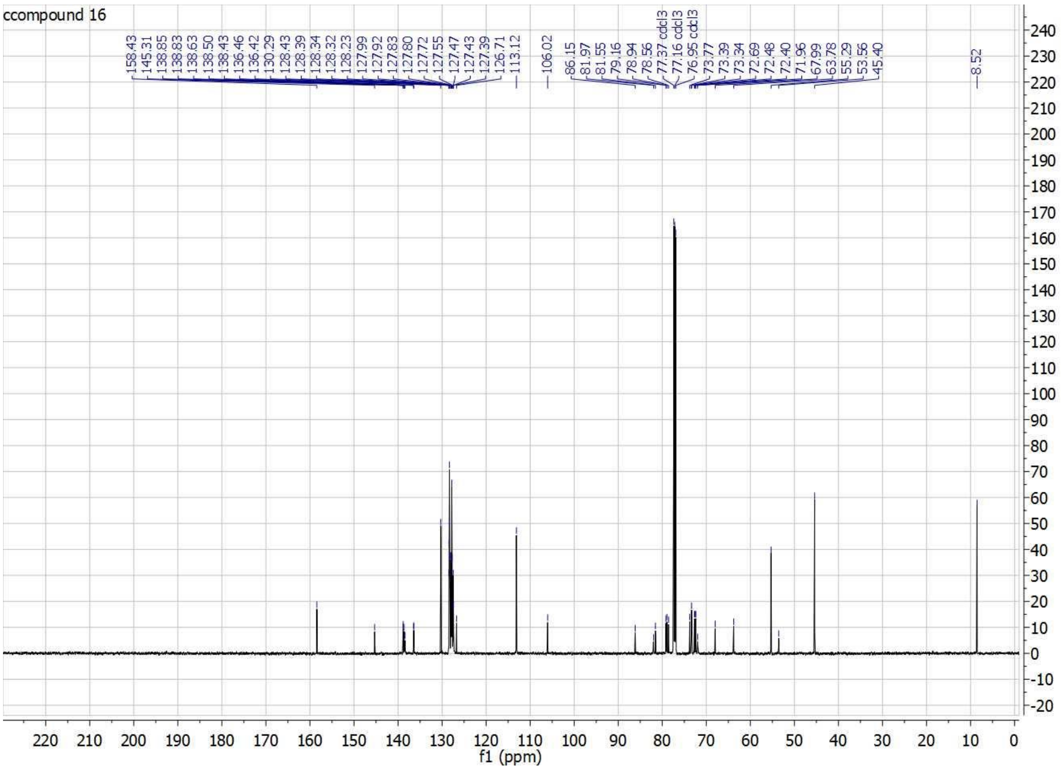

^{31}P NMR(162 MHz, $CDCl_3$)

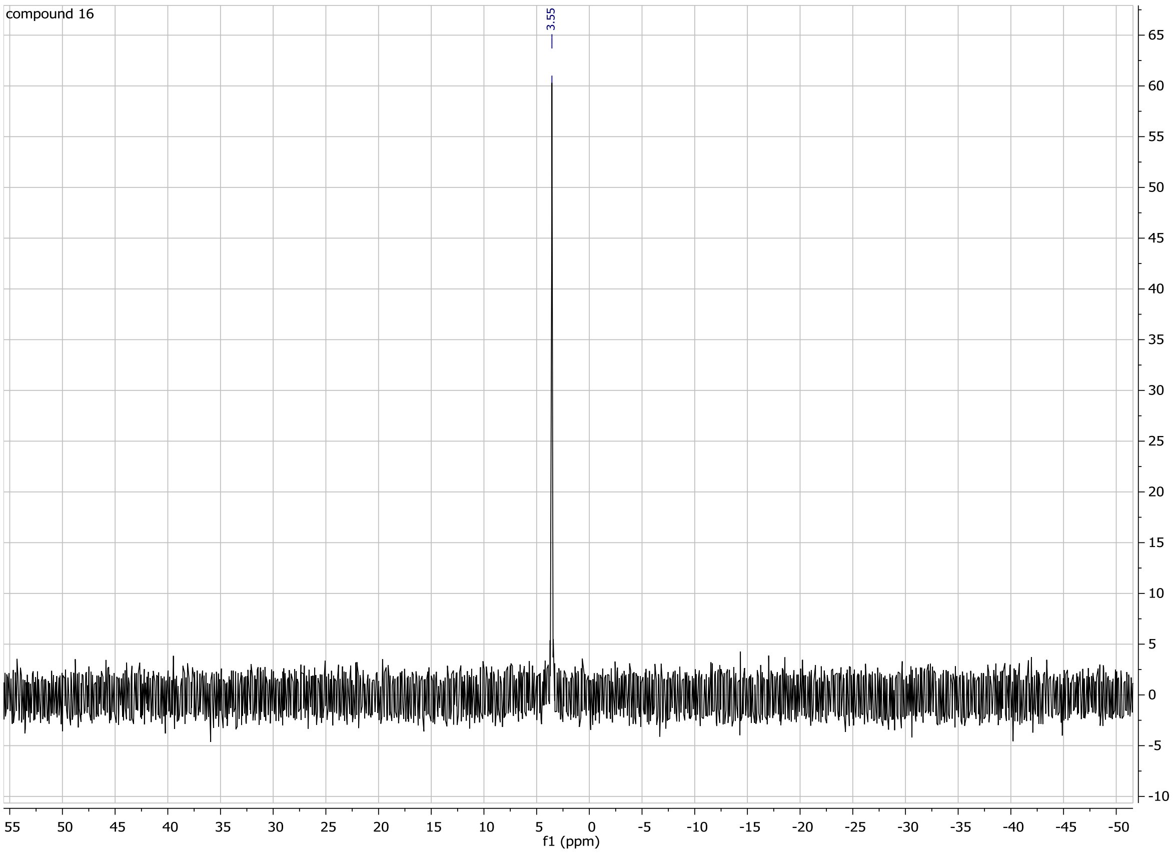

Compound 10

^{1}H NMR (600 MHz, $CDCl_3$)

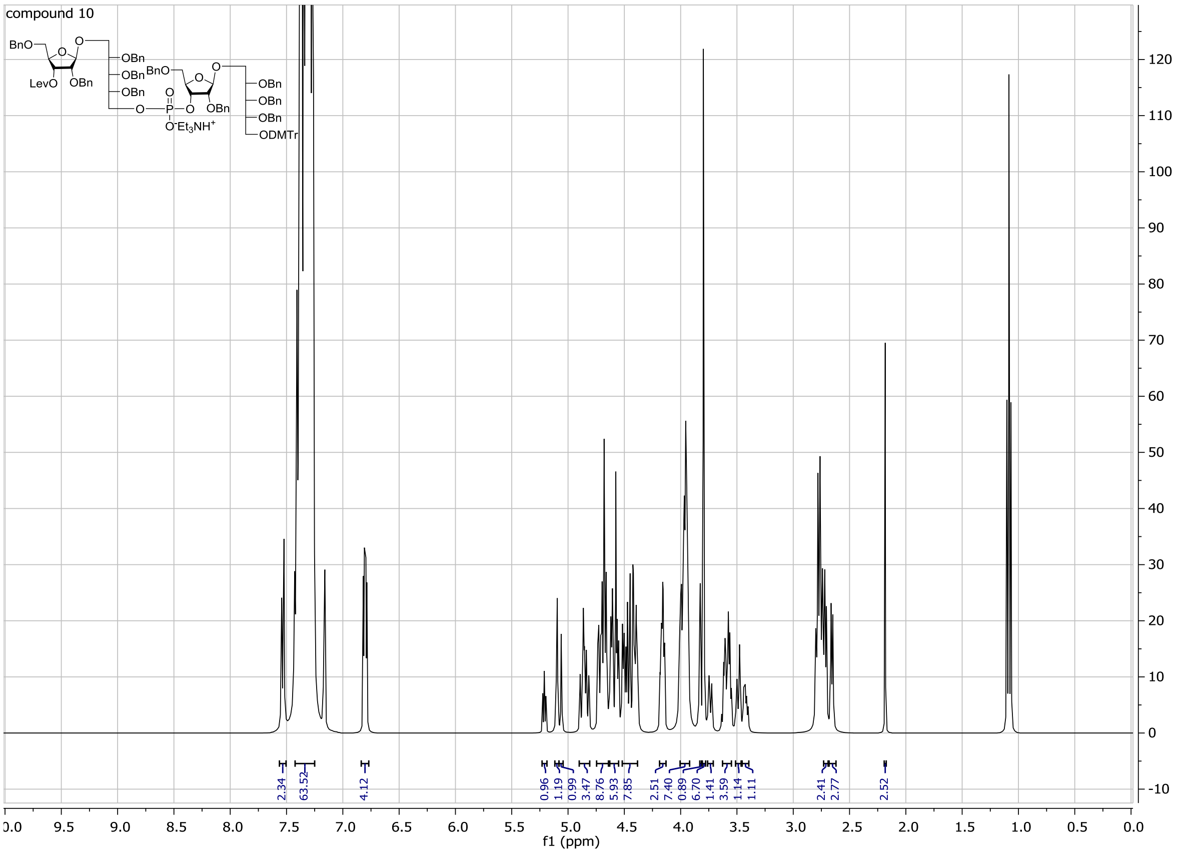

^{13}C NMR(151 MHz, $CDCl_3$)

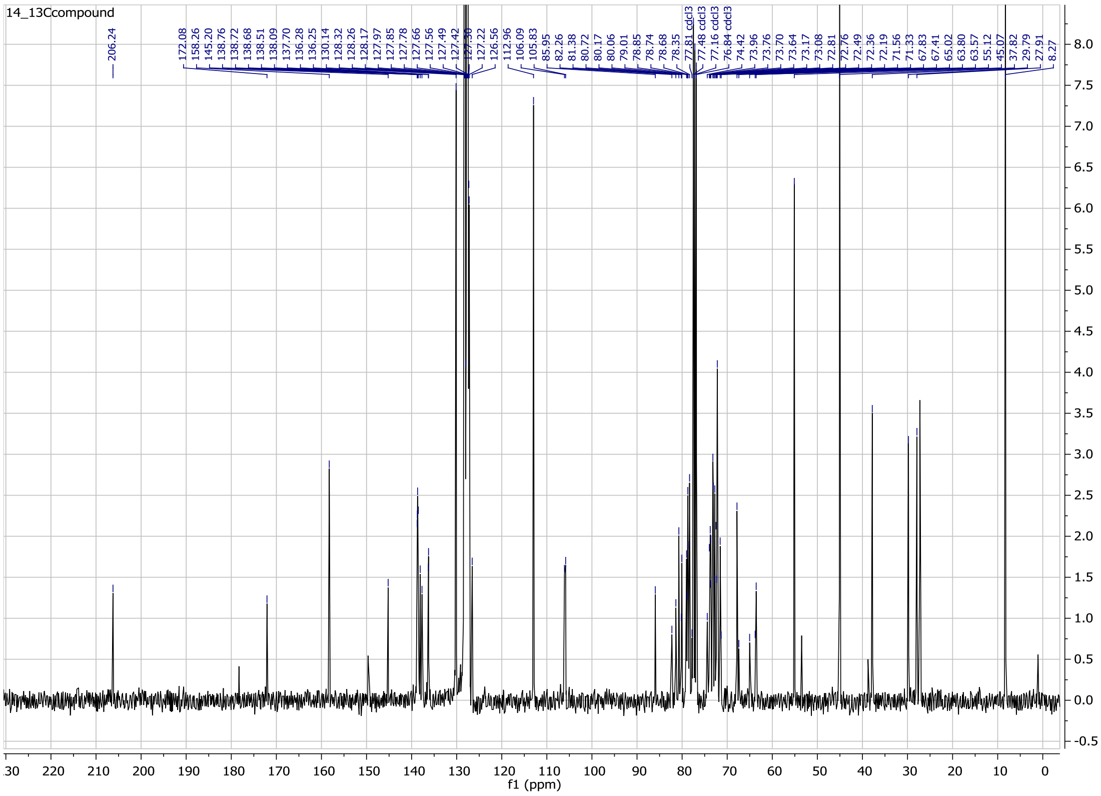

^{31}P NMR(243 MHz, $CDCl_3$)

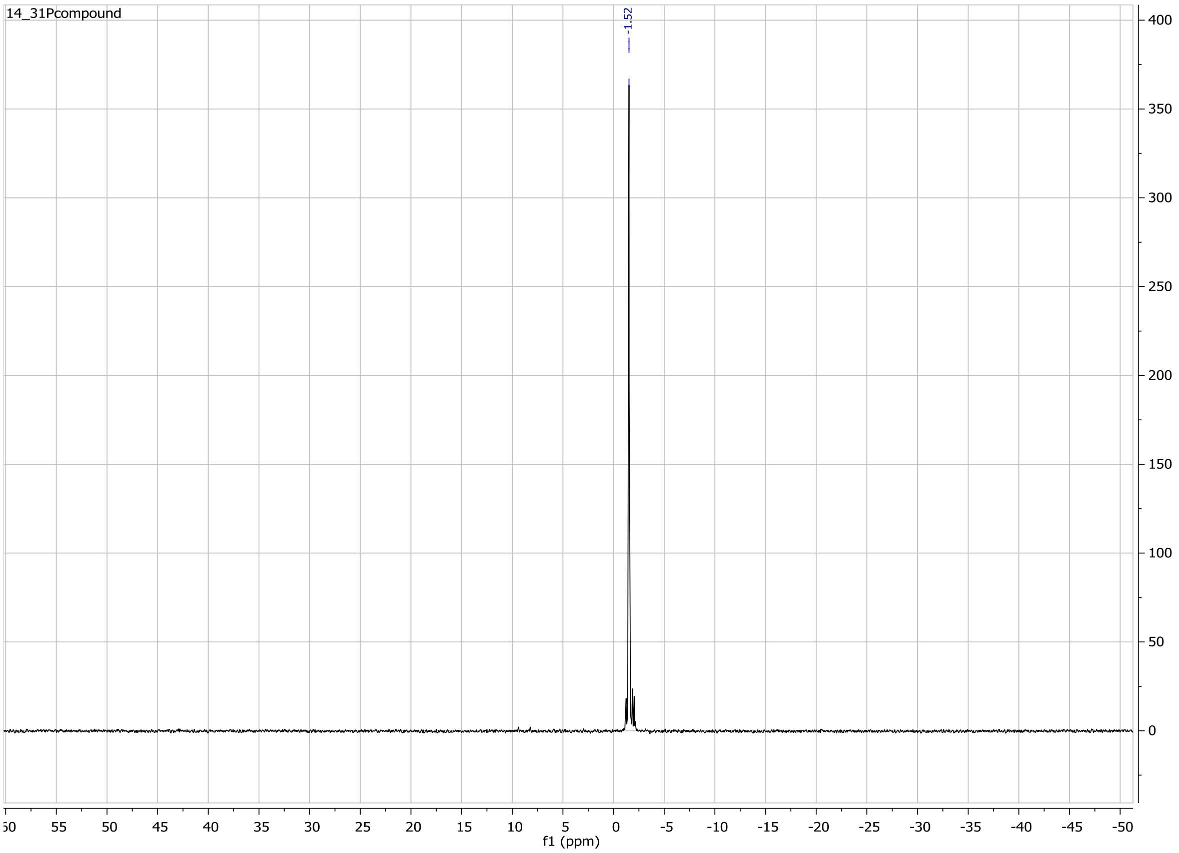

Compound 11

^{1}H NMR (600 MHz, $CDCl_3$)

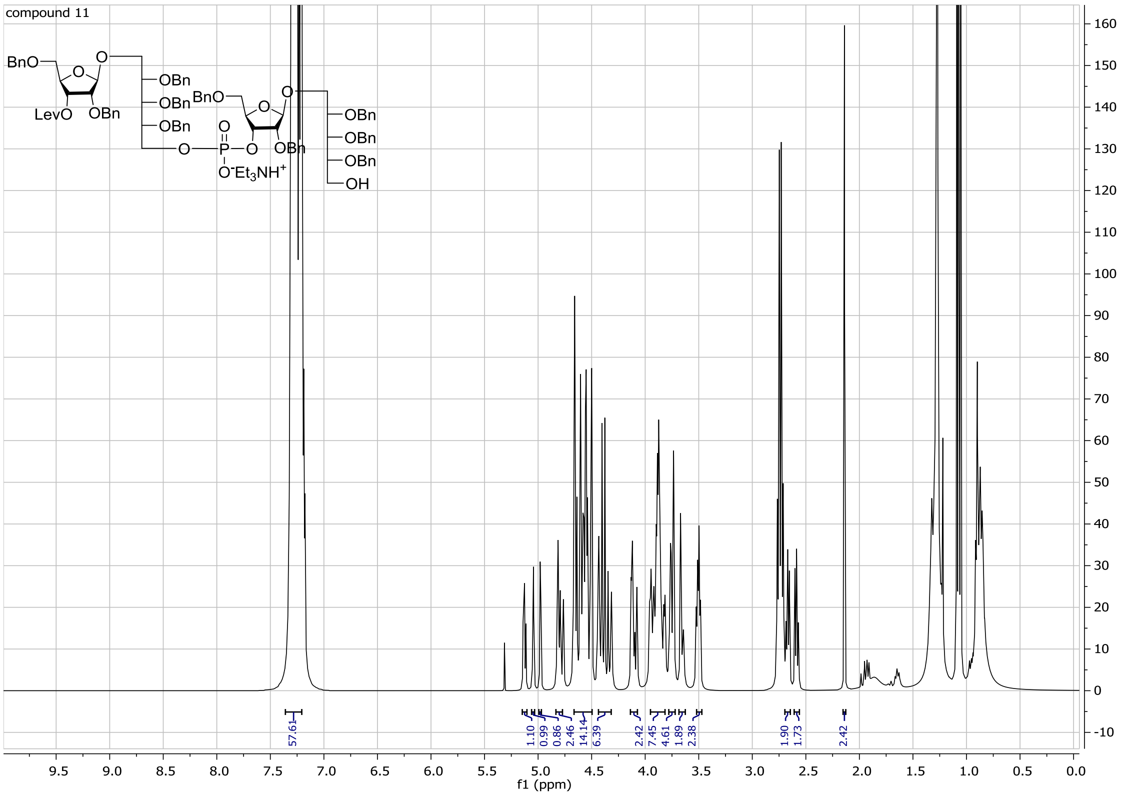

^{13}C NMR(151 MHz, $CDCl_3$)

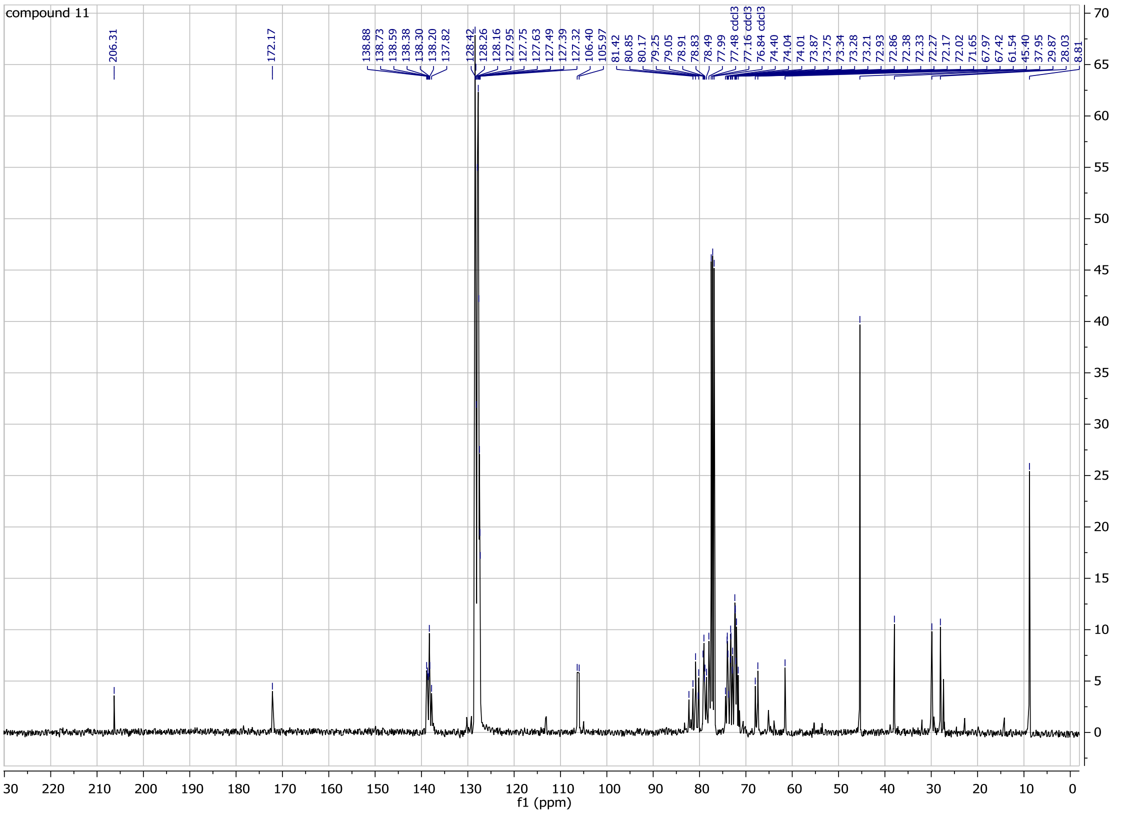

^{31}P NMR(243 MHz, $CDCl_3$)

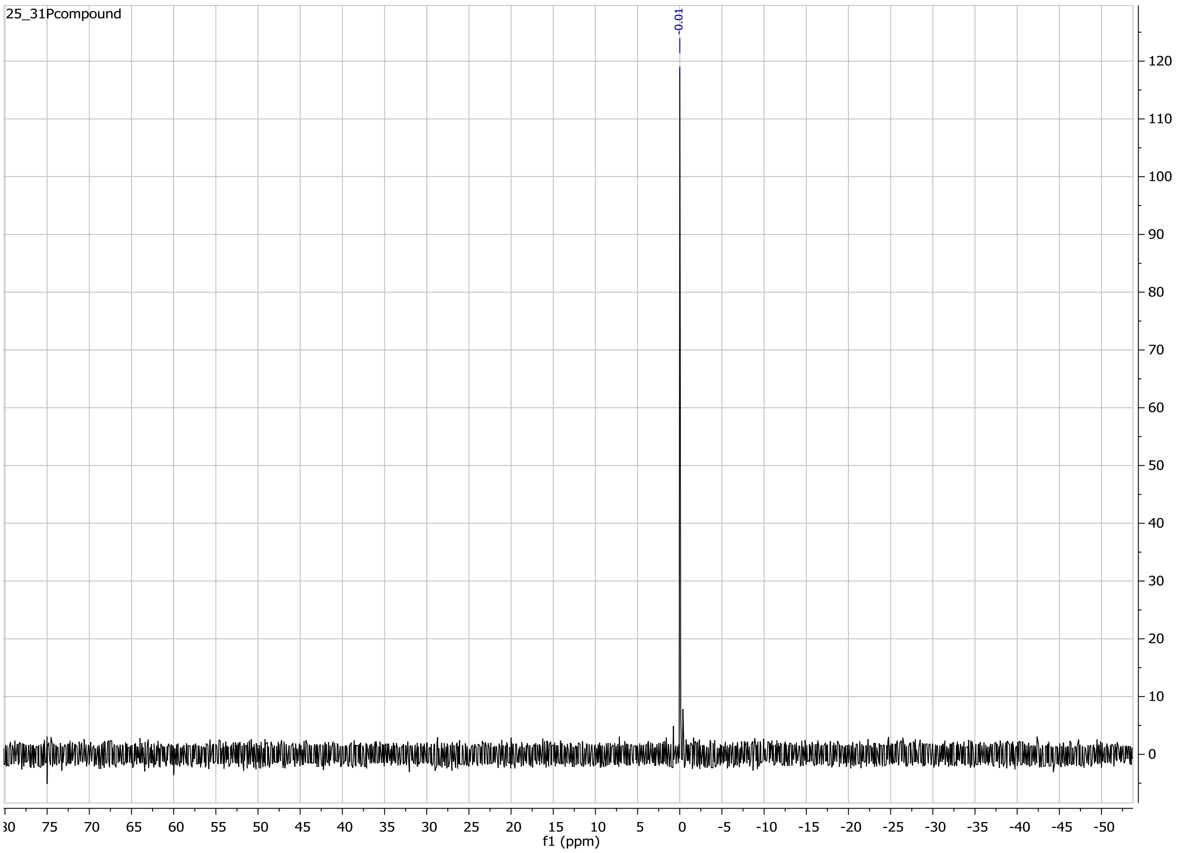

Compound 12

^{1}H NMR (600 MHz, $CDCl_3$)

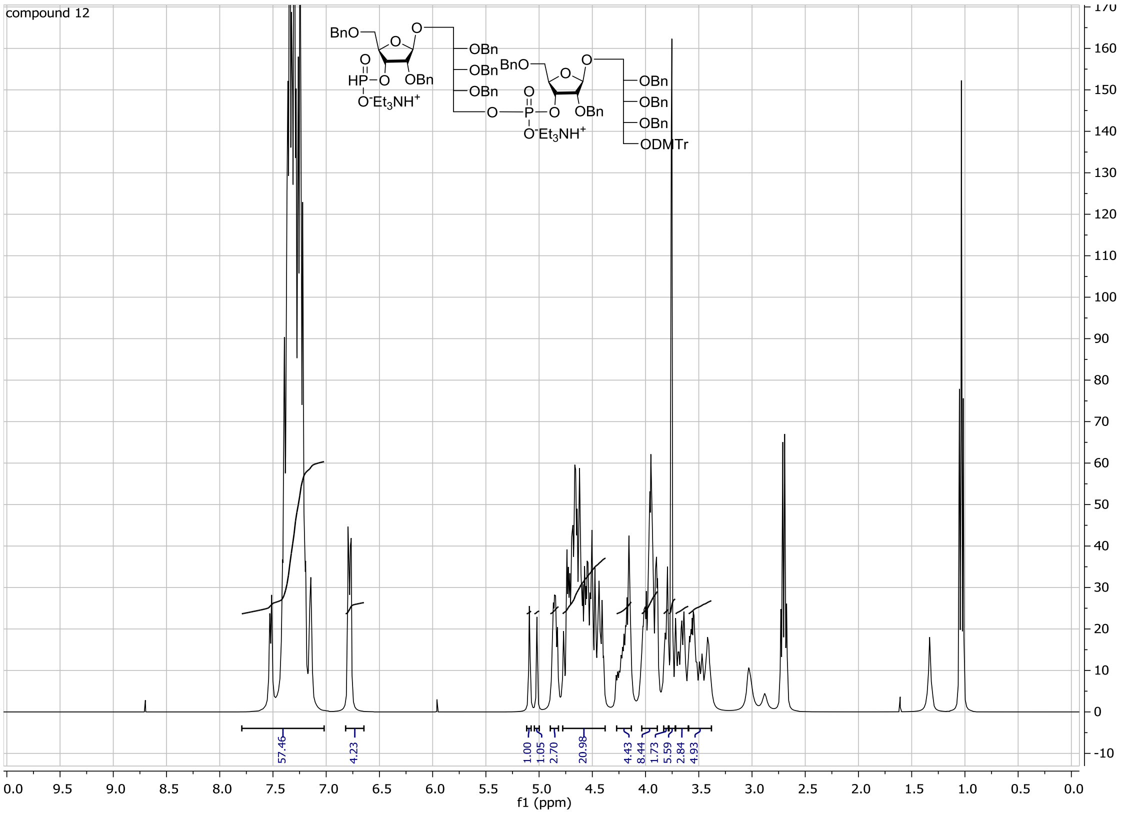

^{13}C NMR (151 MHz, $CDCl_3$)

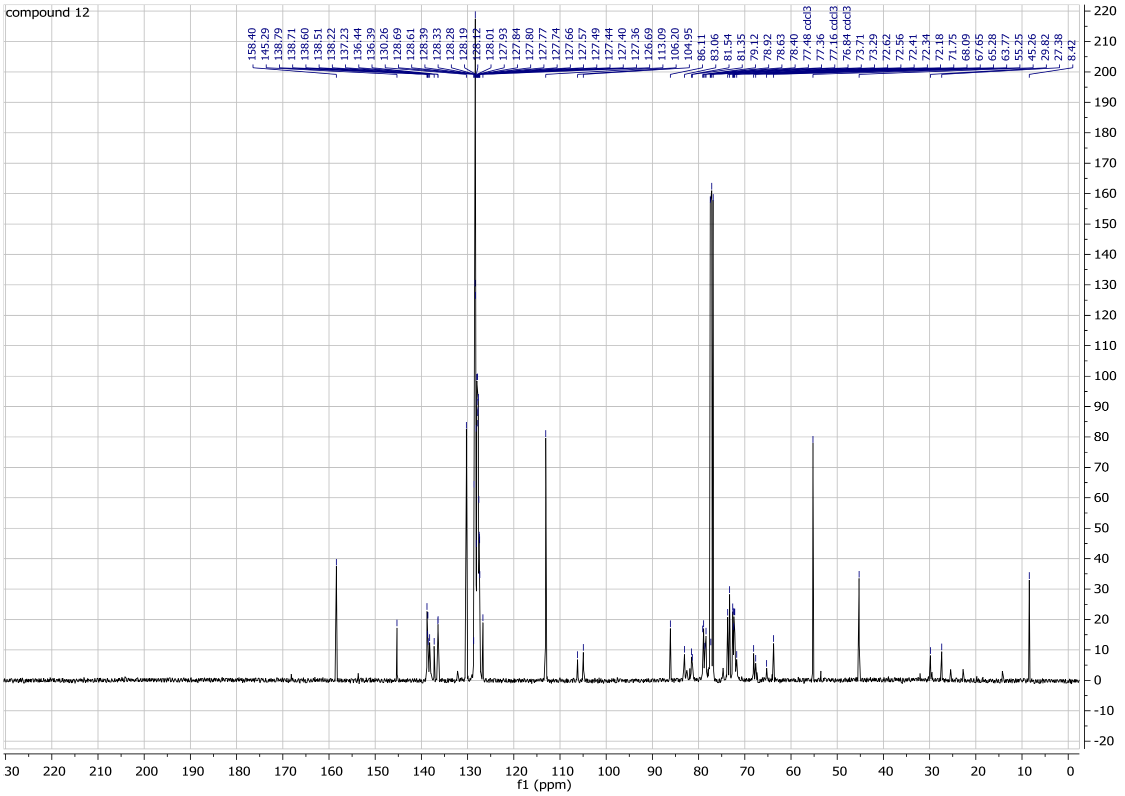

^{31}P NMR (243 MHz, $CDCl_3$)

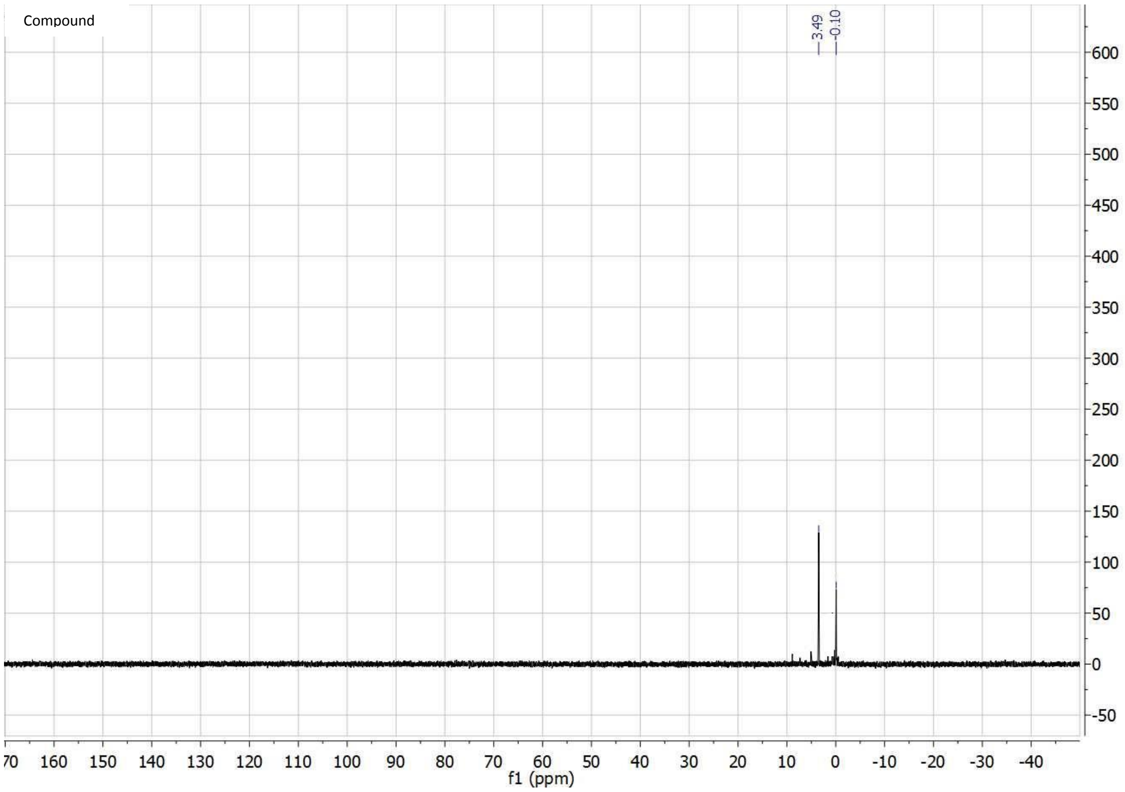

Compound 5

^{1}H NMR (600 MHz, $CDCl_3$)

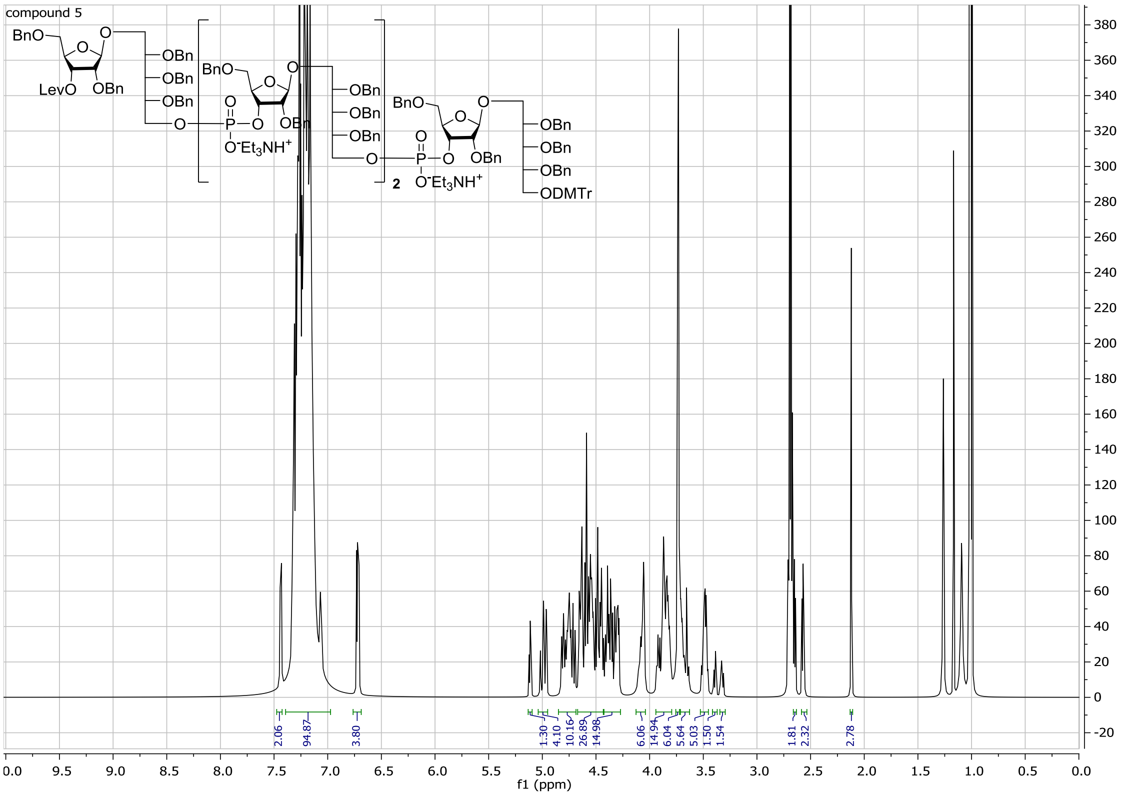

^{13}C NMR (151 MHz, $CDCl_3$)

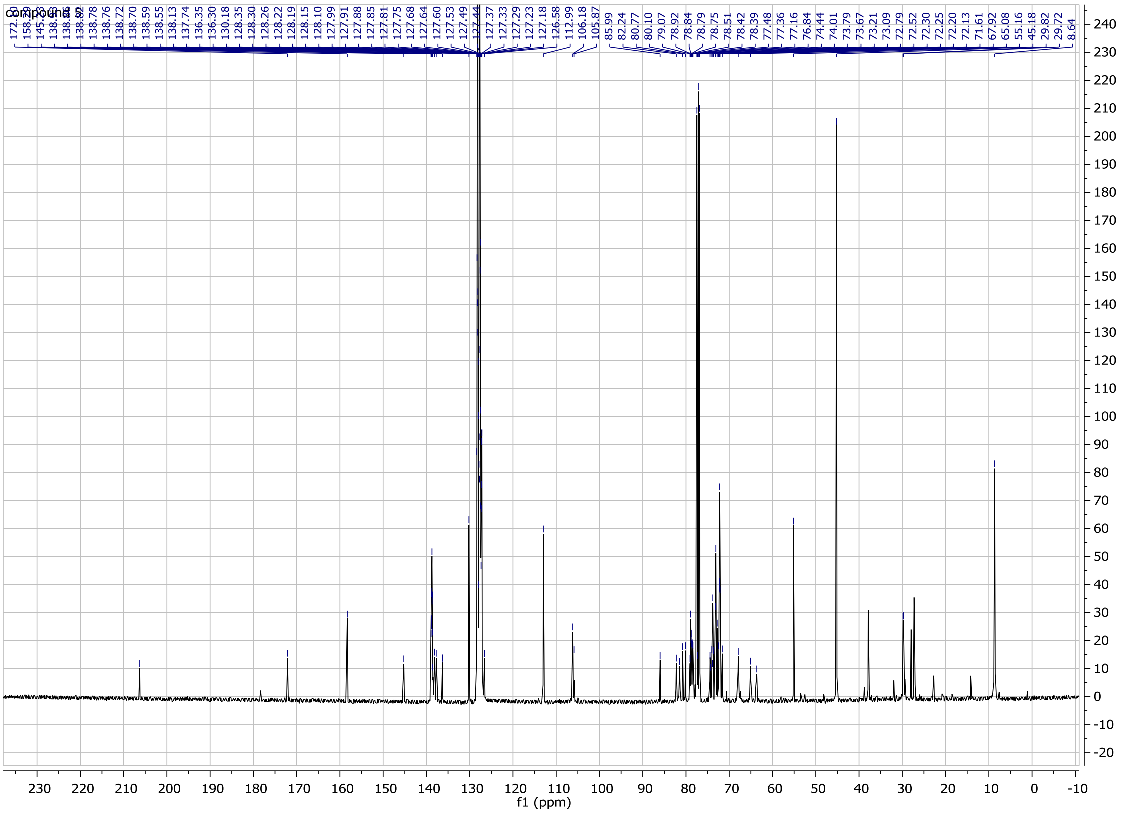

^{31}P NMR (243 MHz, $CDCl_3$)

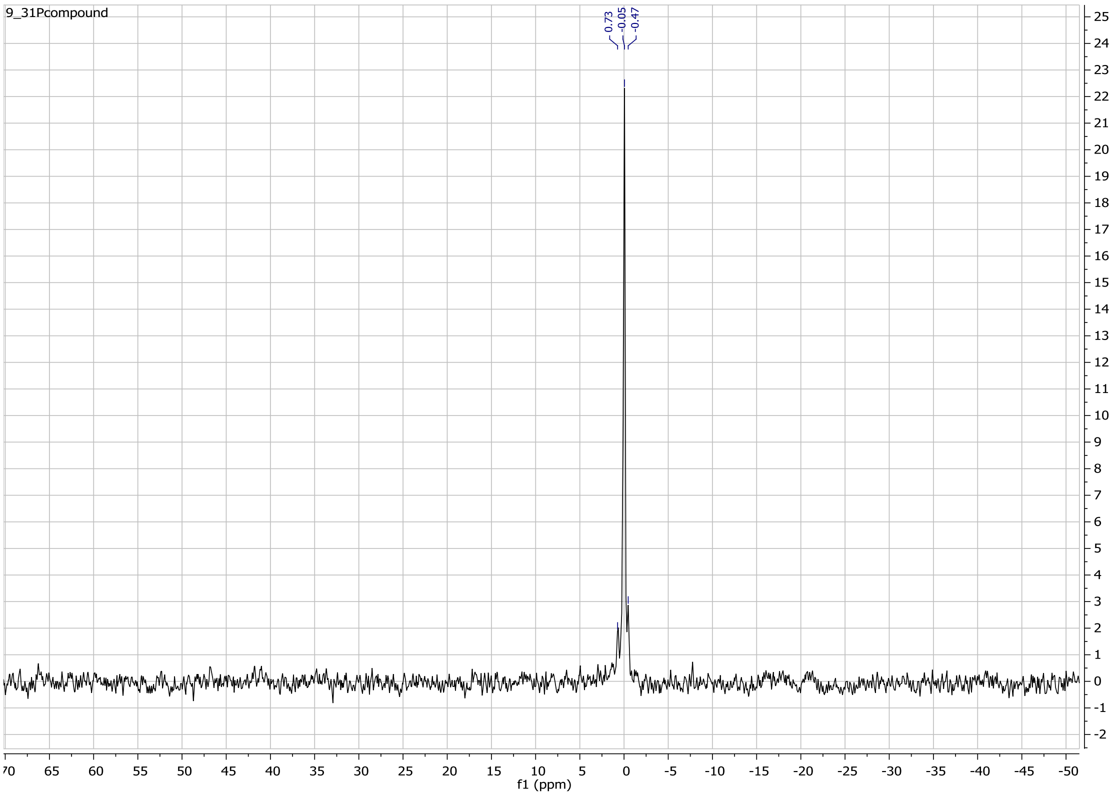

HSQC (600 MHz, $CDCl_3$)

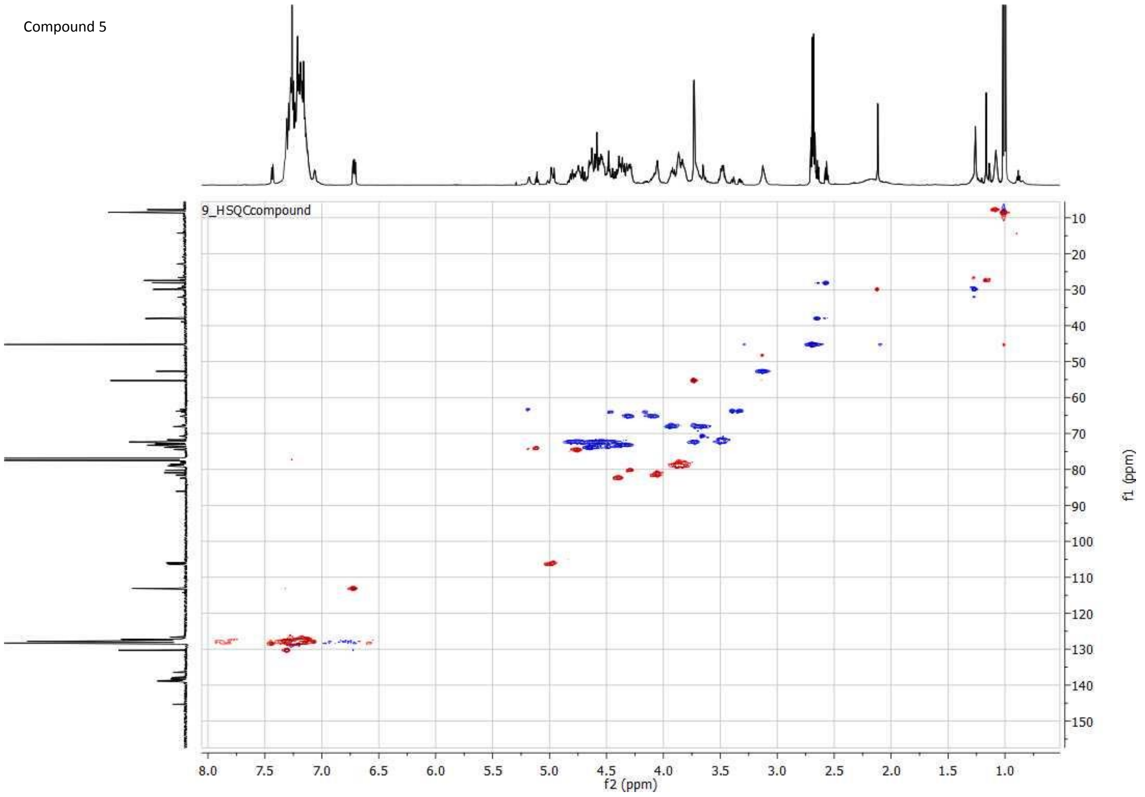

Compound 9

^{1}H NMR (400 MHz, $CDCl_3$)

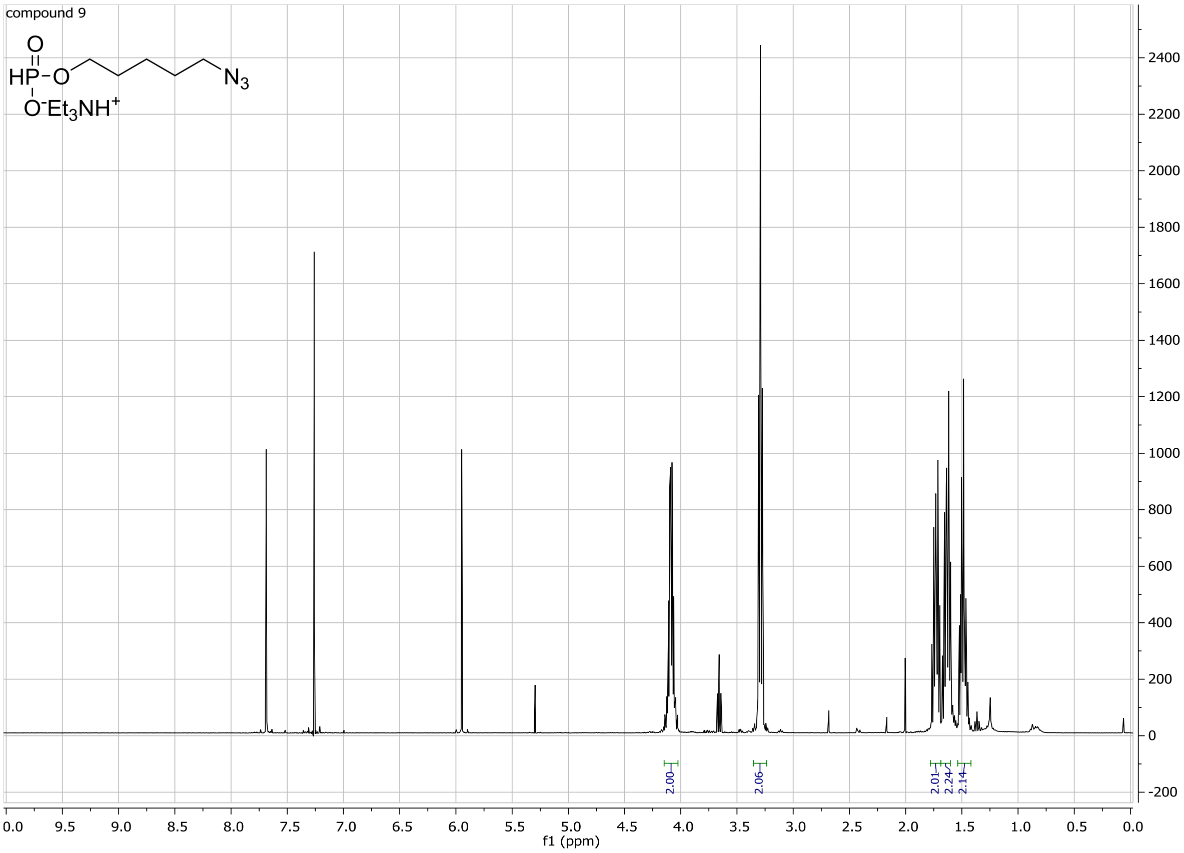

^{1}H-^{1}H COSY (400 MHz, $CDCl_3$)

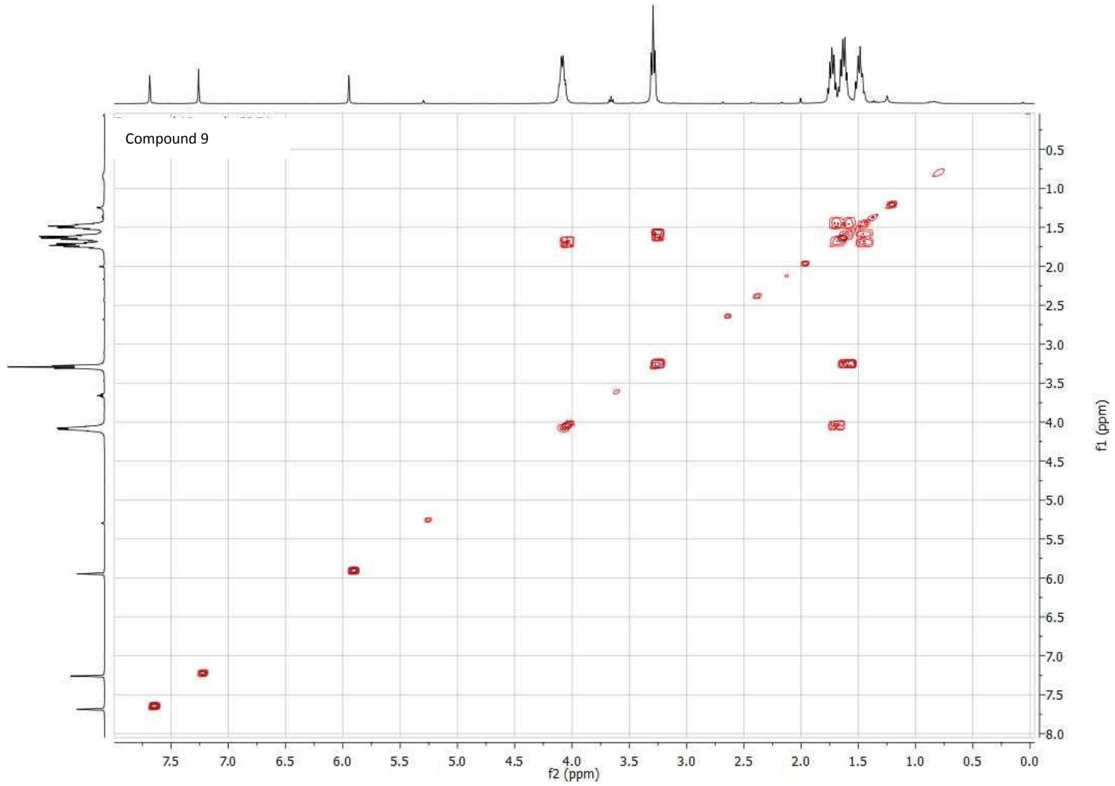

^{31}P NMR(162 MHz, $CDCl_3$)

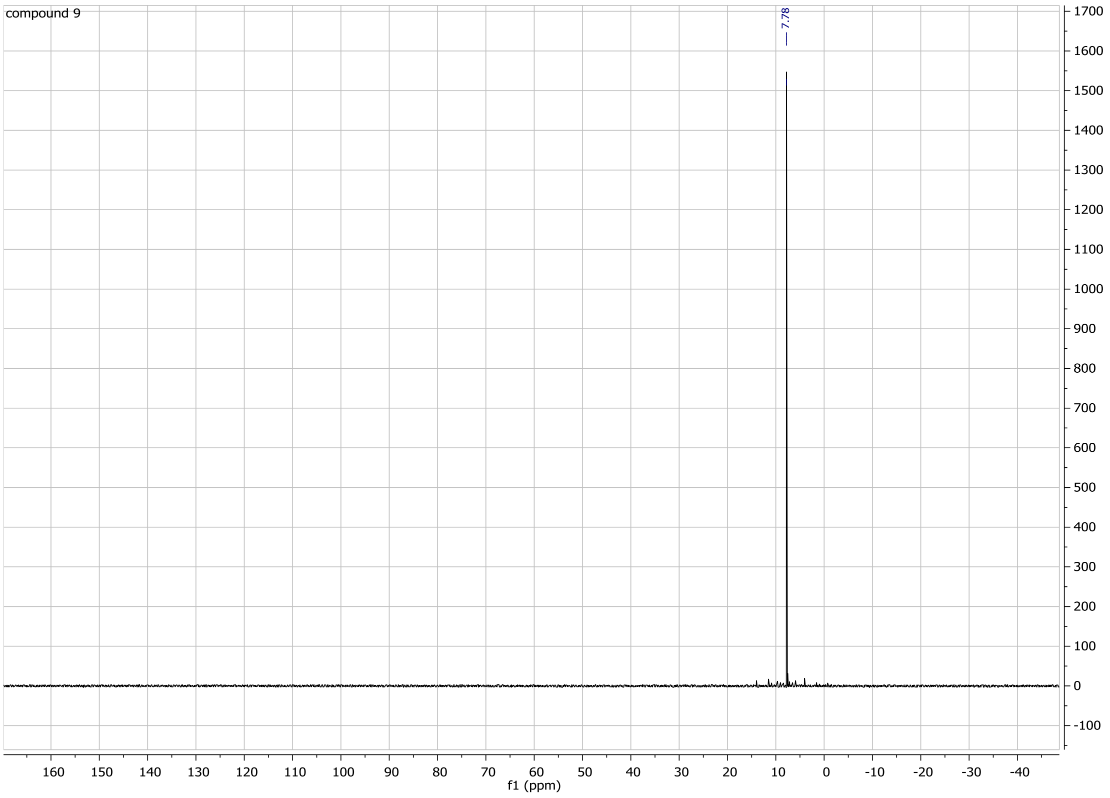

HSQC (400 MHz, $CDCl_3$)

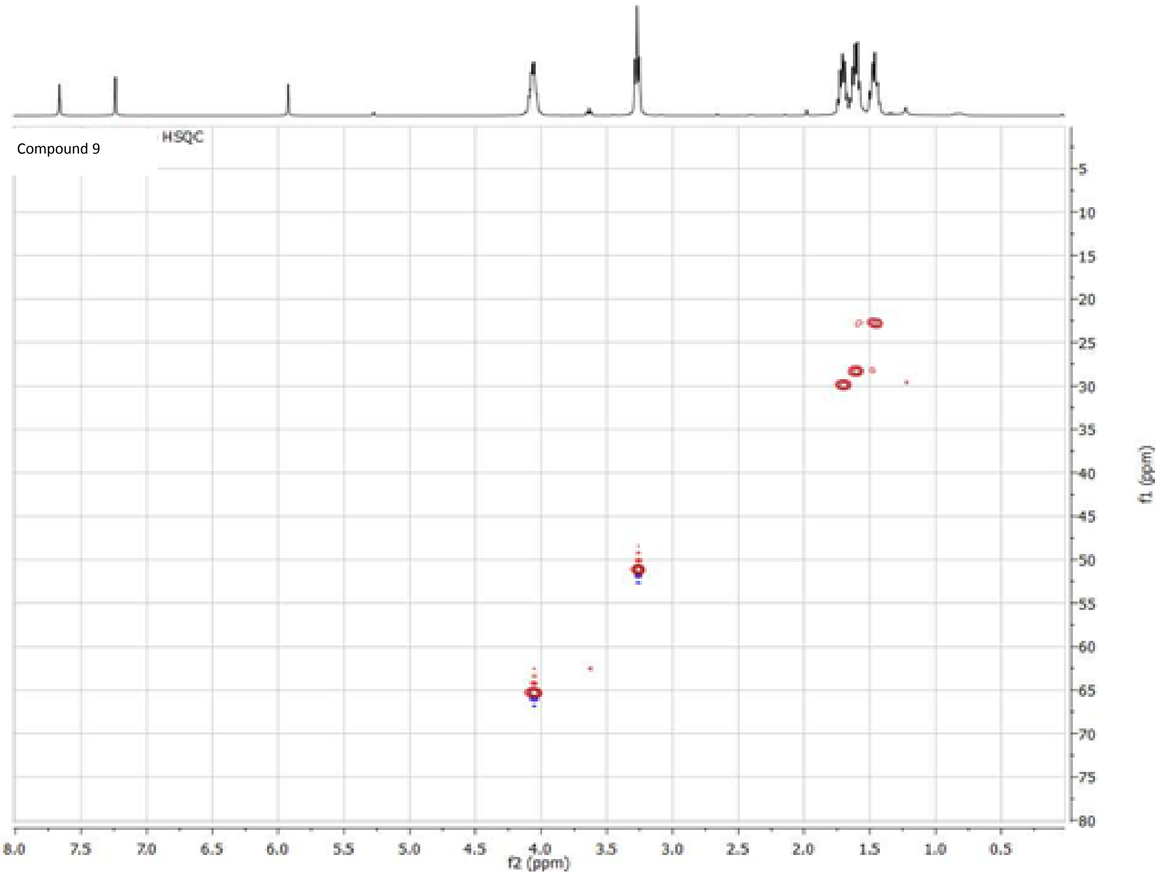

Compound 25

^{1}H NMR (600 MHz, $CDCl_3$)

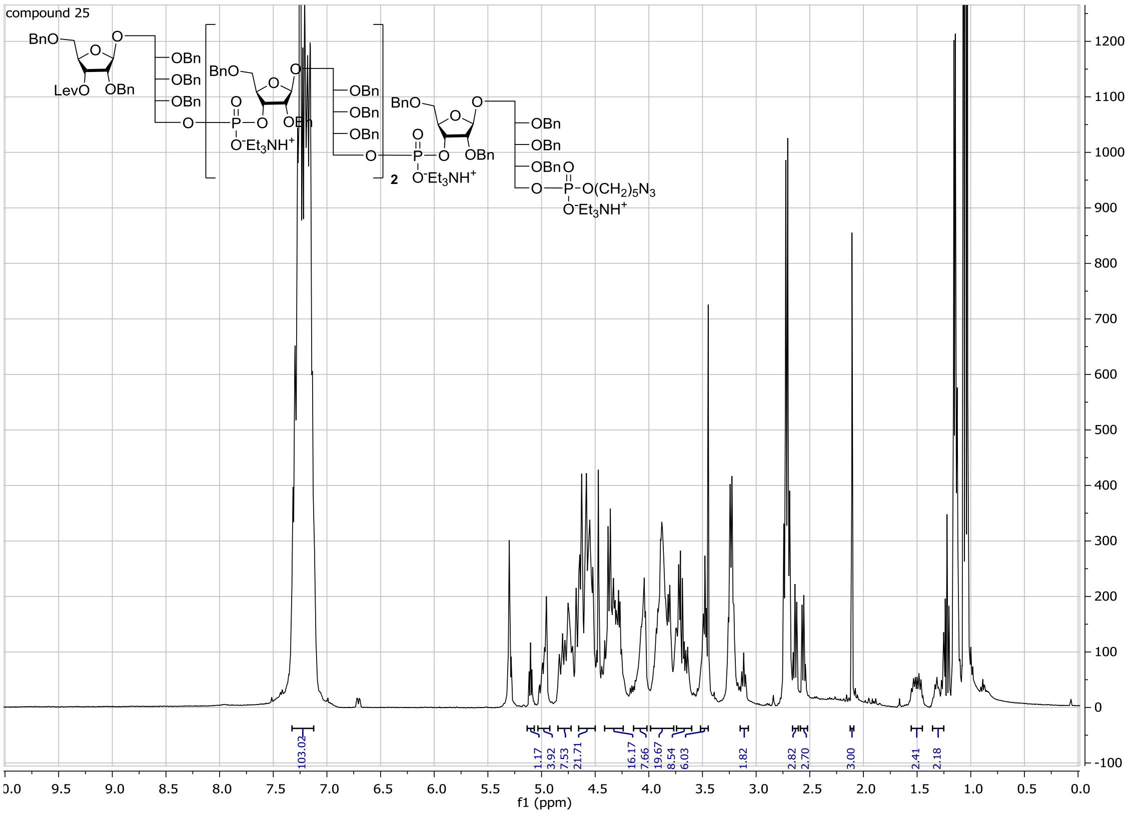

^{13}C NMR(151 MHz, $CDCl_3$)

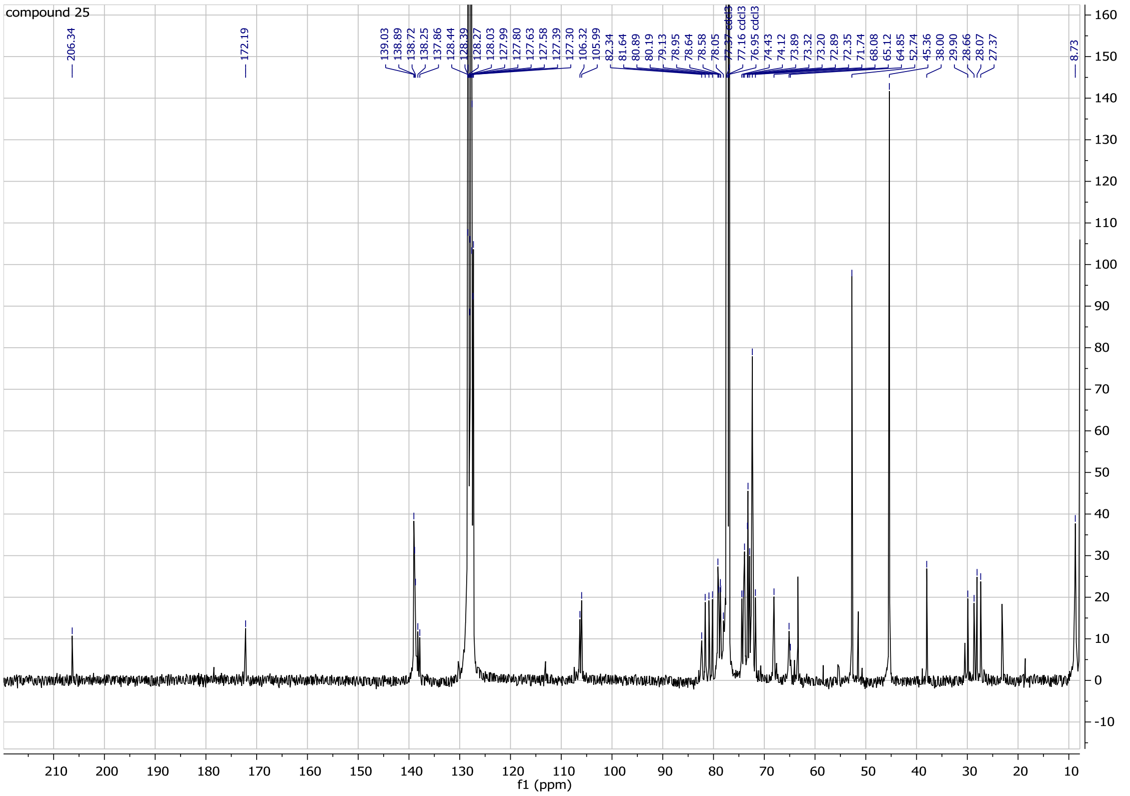

^{31}P NMR(243 MHz, $CDCl_3$)

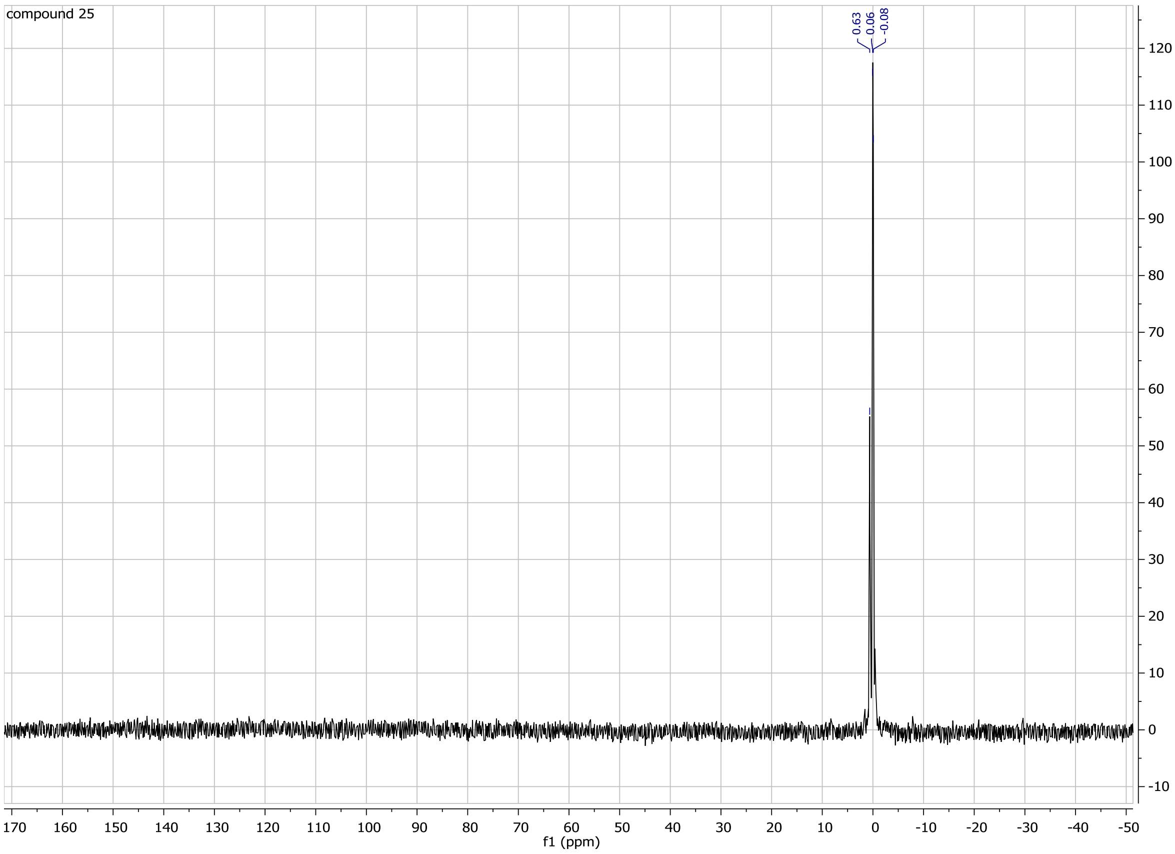

HSQC(600 MHz, $CDCl_3$)

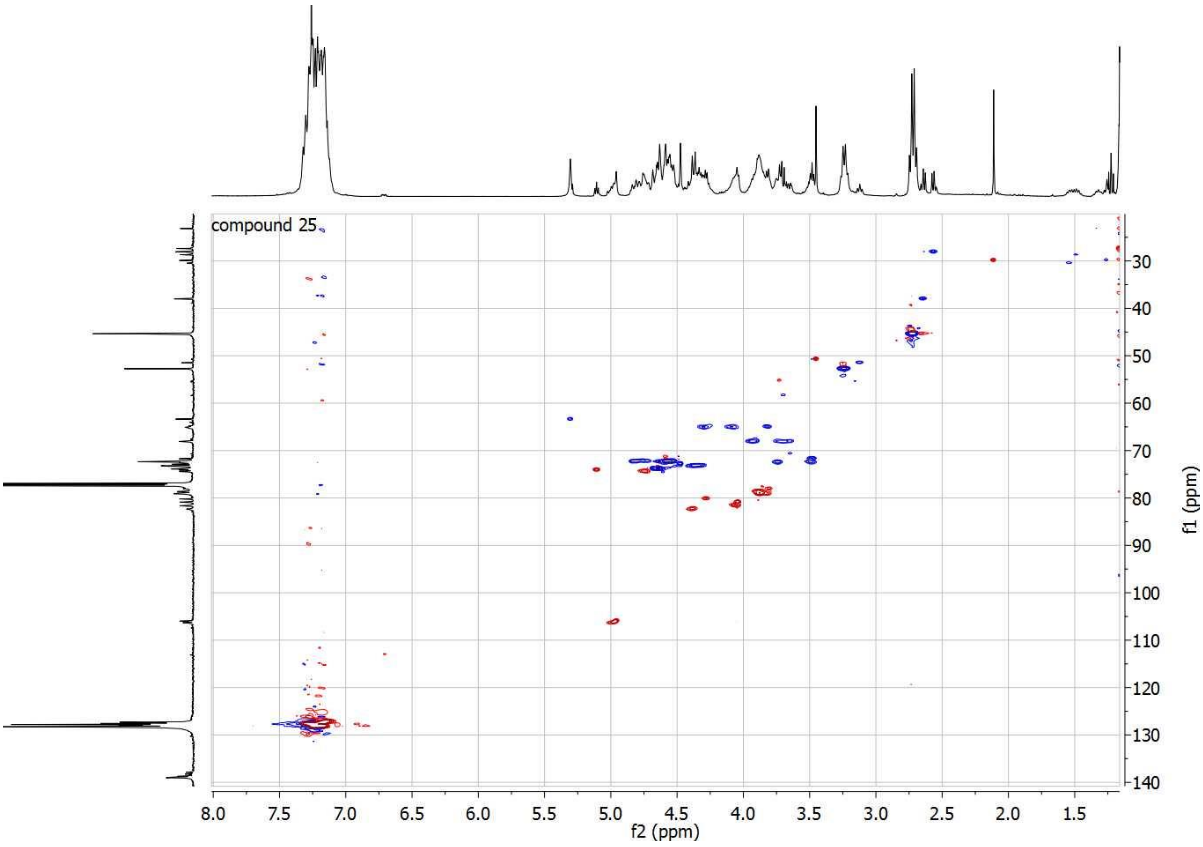

Compound 26

^{1}H NMR (600 MHz, $CDCl_3$)

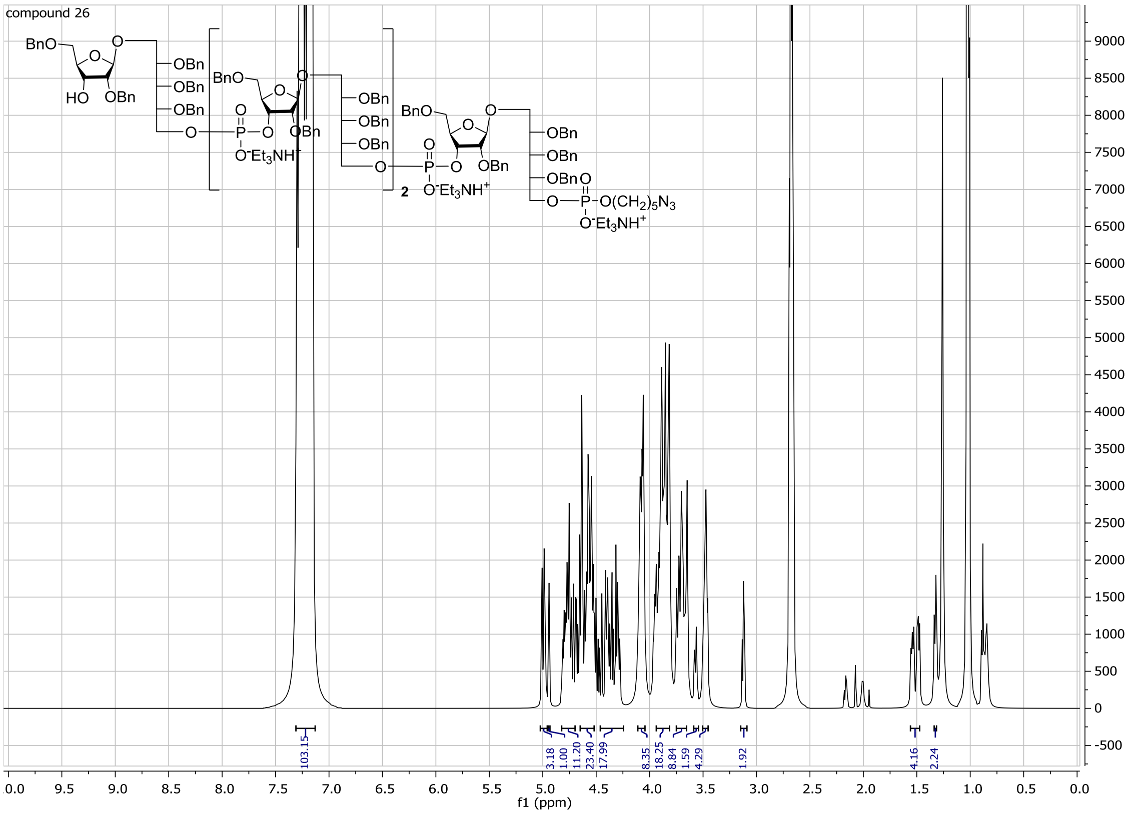

^{13}C NMR(151 MHz, $CDCl_3$)

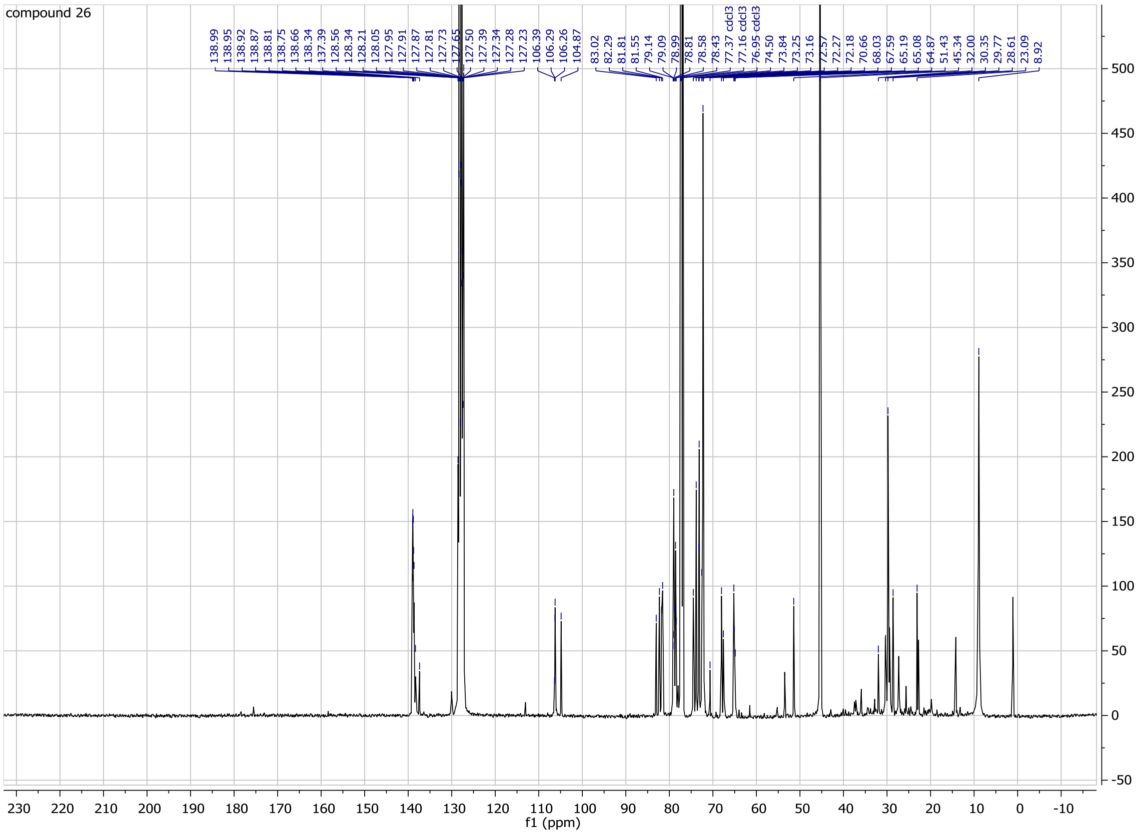

^{31}P NMR(243 MHz, $CDCl_3$)

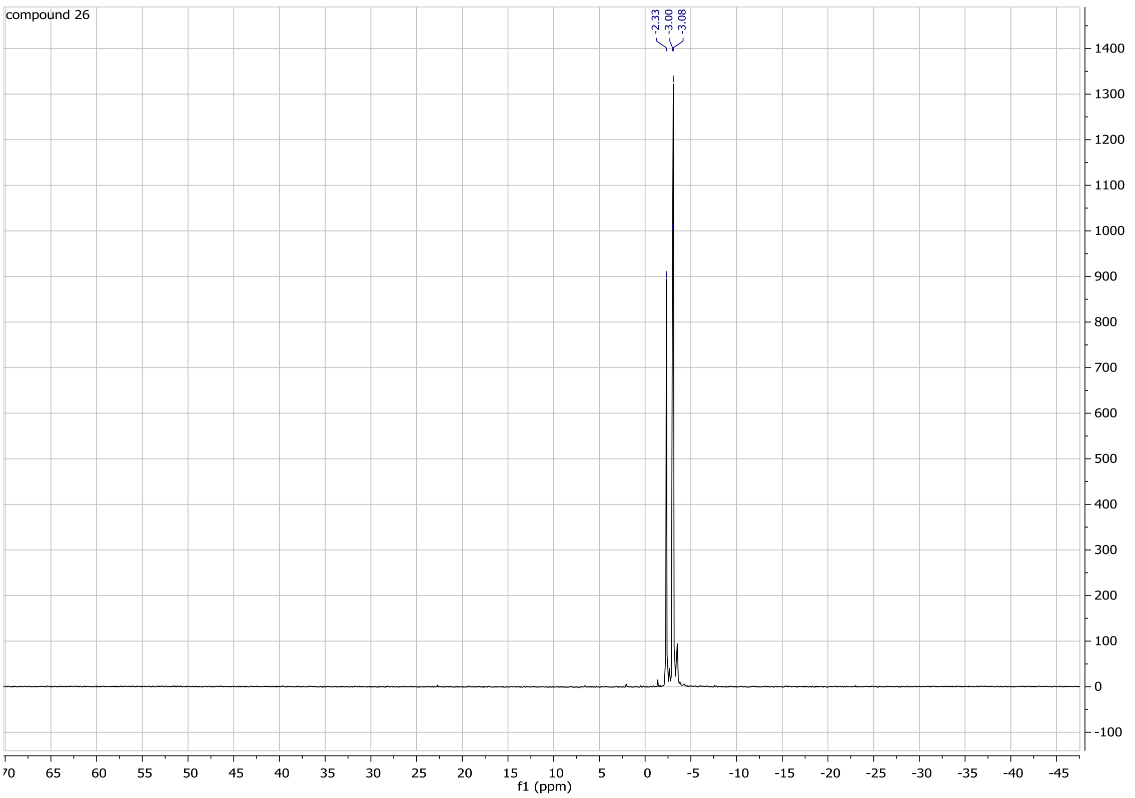

HSQC (600 MHz, $CDCl_3$)

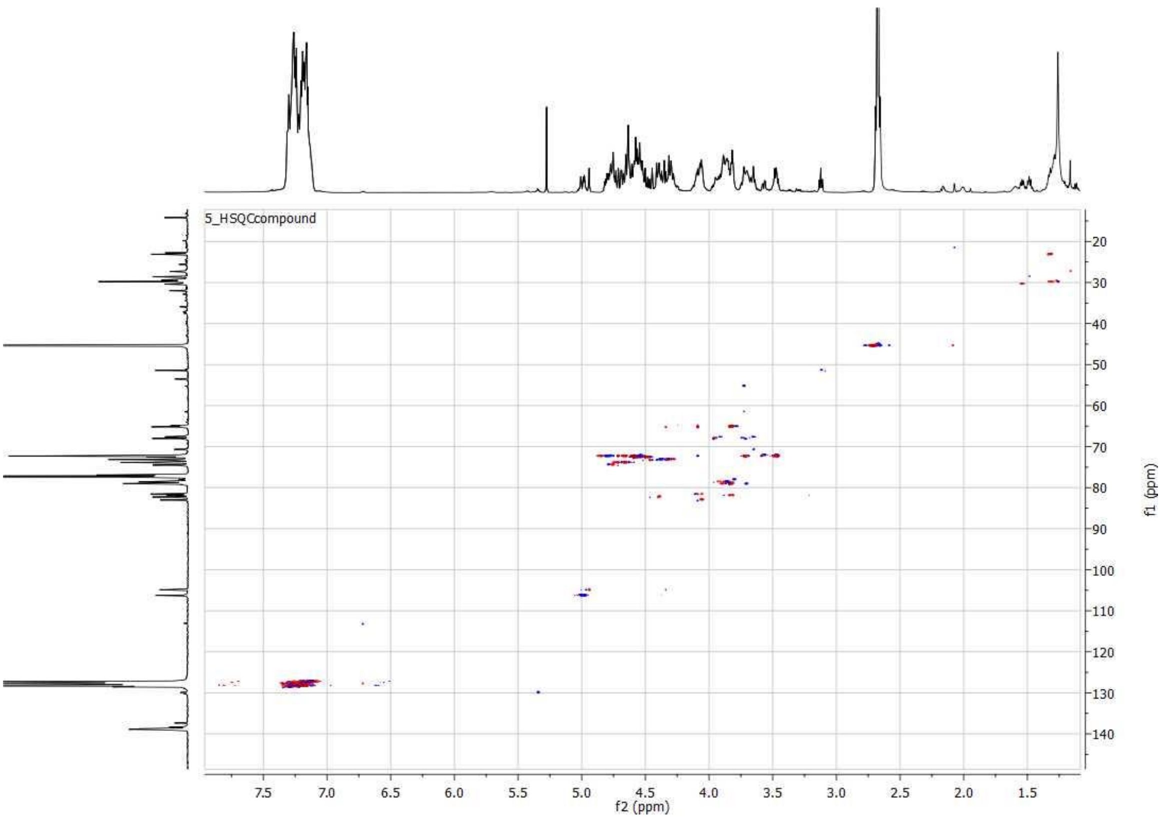

Compound 1

^{1}H NMR (600 MHz, D_2O)

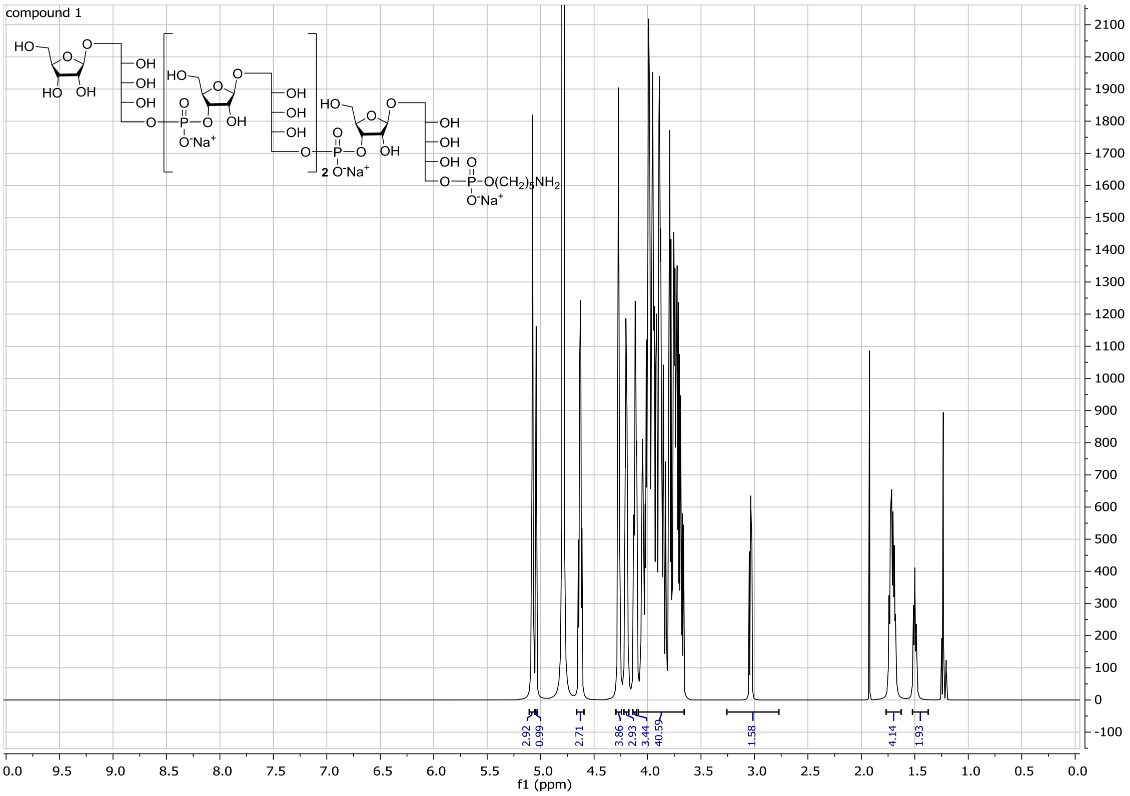

^{13}C NMR (151 MHz, D_2O)

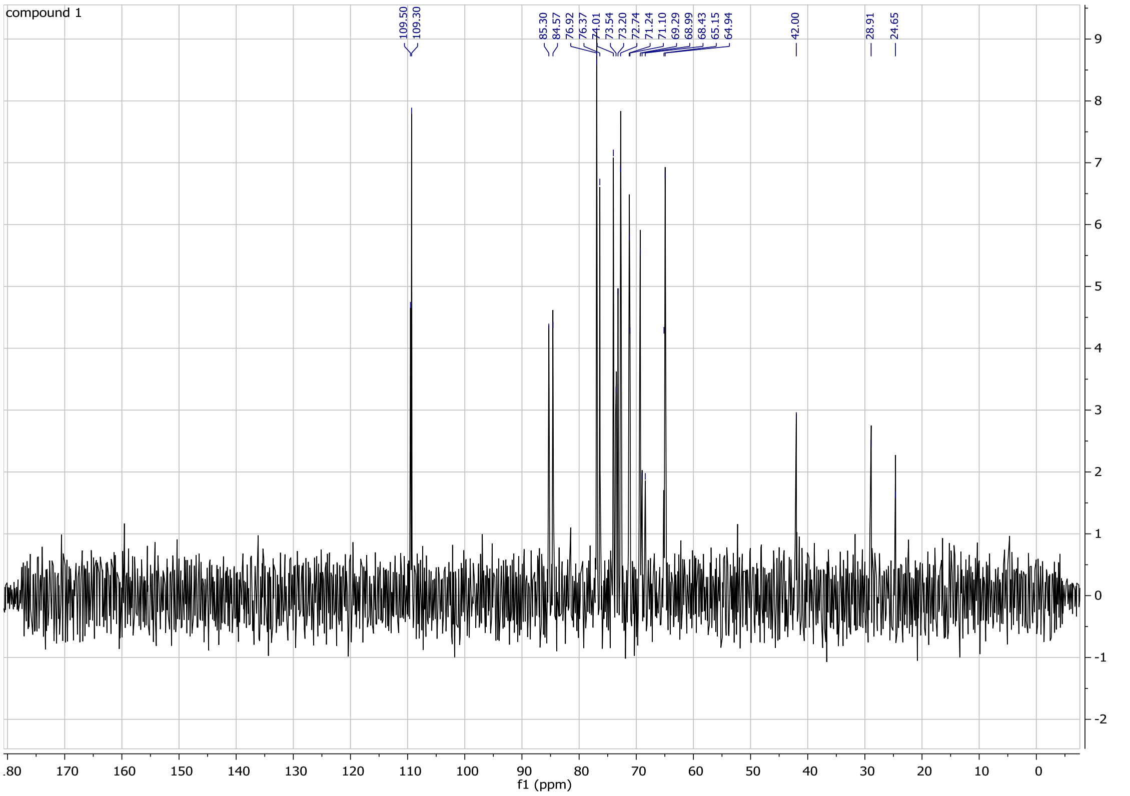

^{31}P NMR (243 MHz, D_2O)

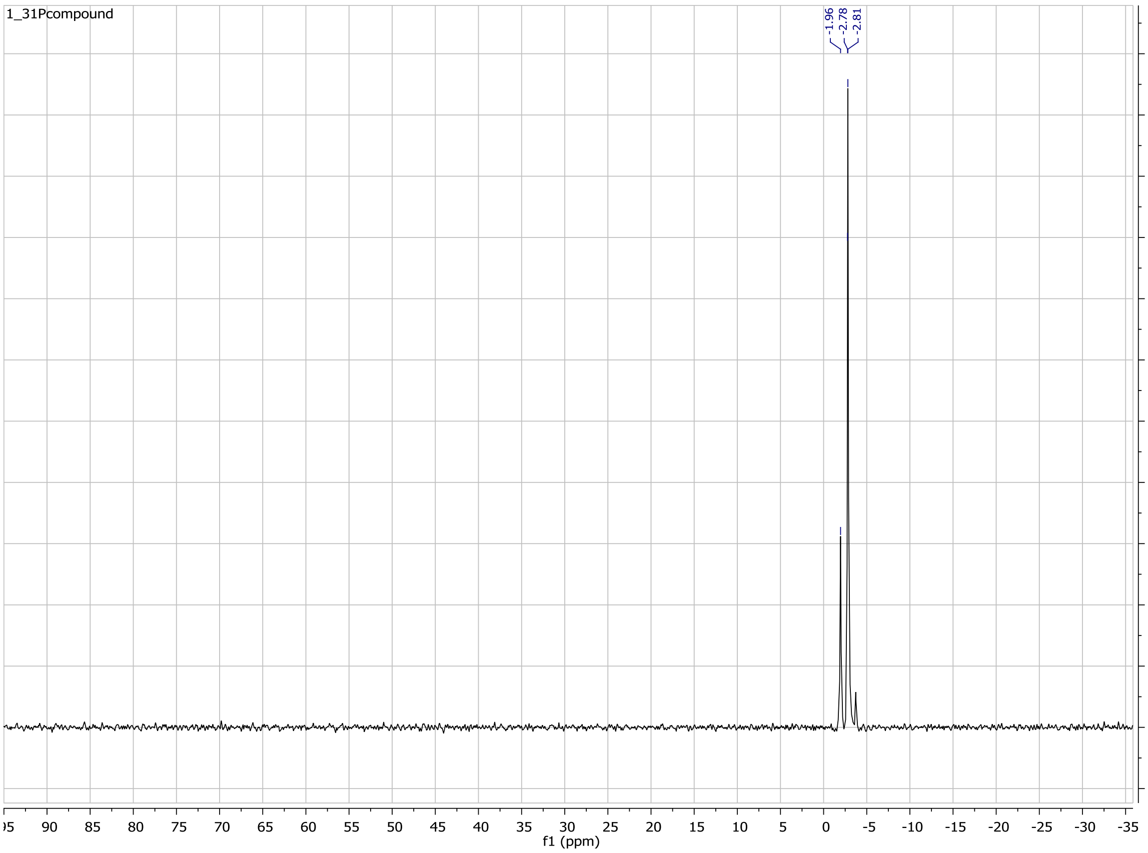

^1H-^1H COSY (600 MHz, D_2O)

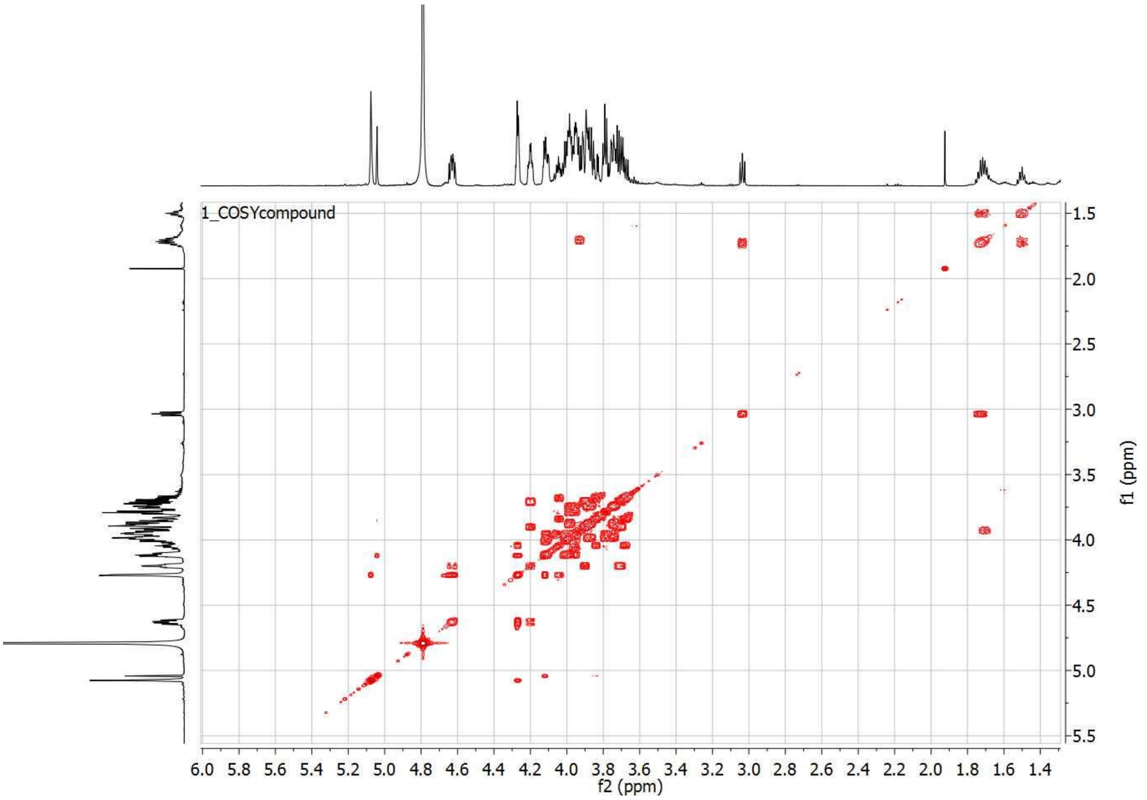

^1H-^1H TOCSY (600 MHz, D_2O)

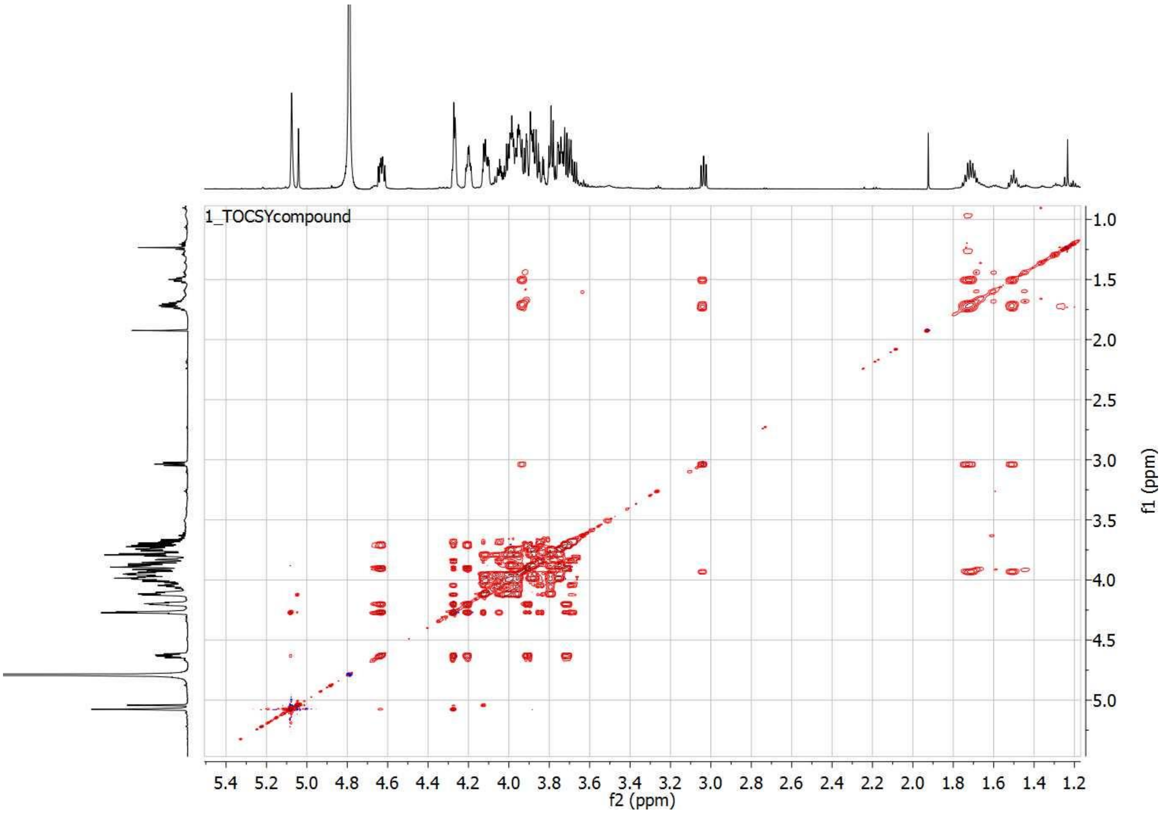

HSQC (600 MHz, D_2O)

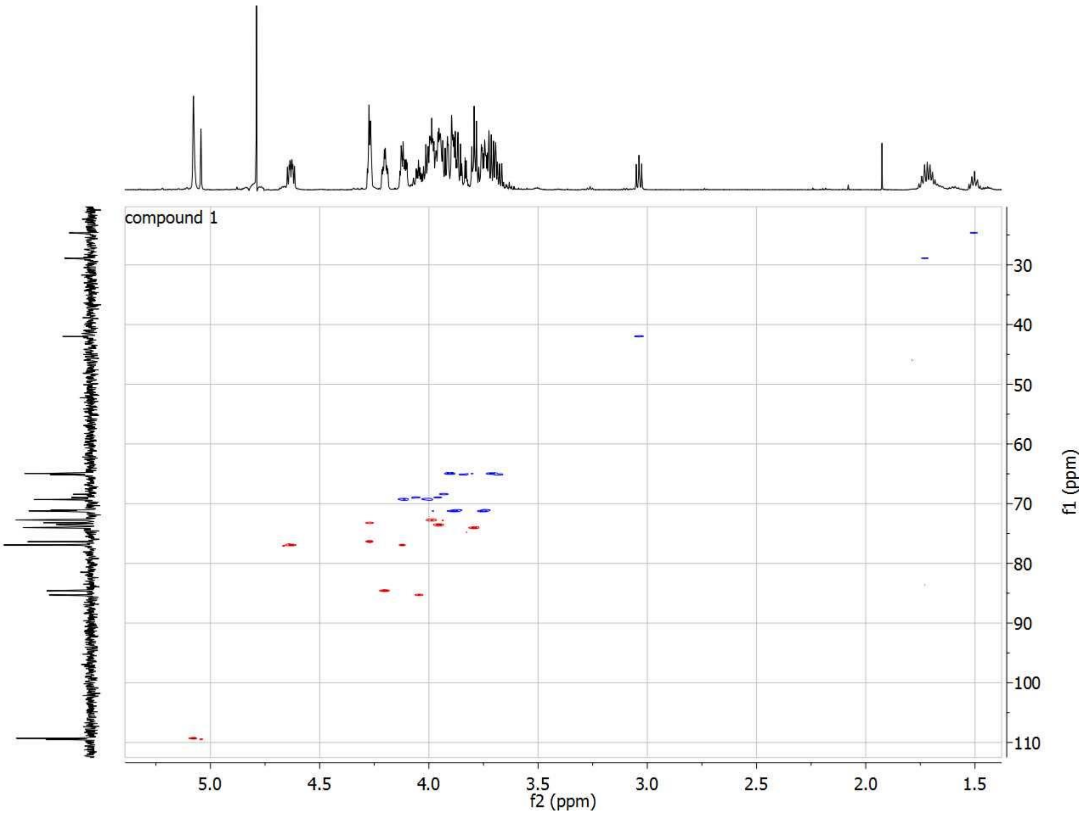

Compound 6

^{1}H NMR (600 MHz, CDCl$_3$)

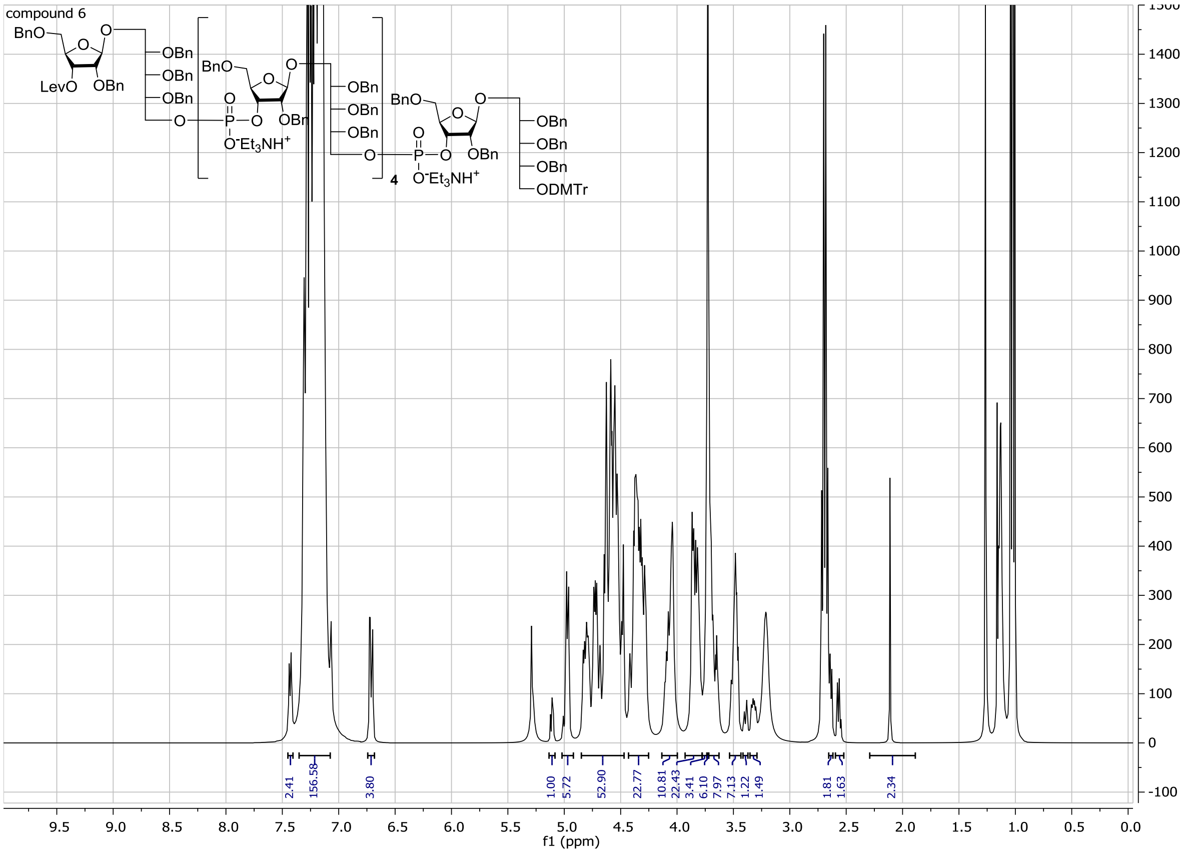

^{13}C NMR (151 MHz, CDCl$_3$)

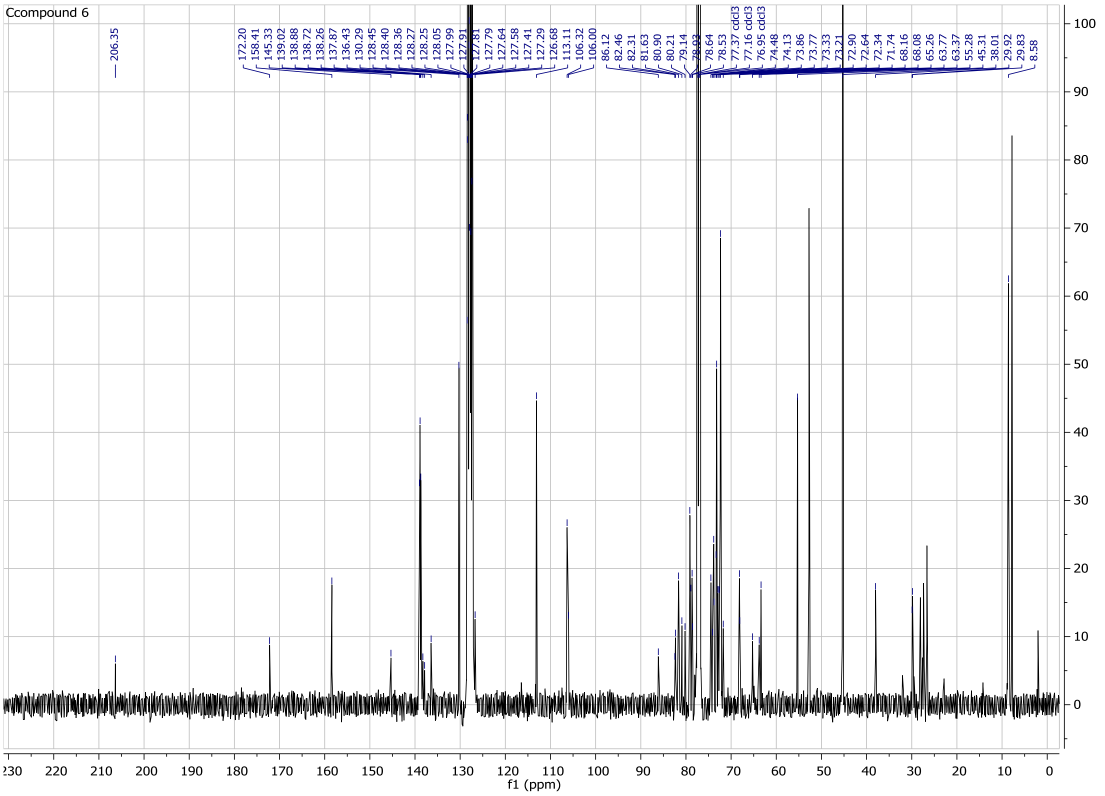

^{31}P NMR (243 MHz, $CDCl_3$)

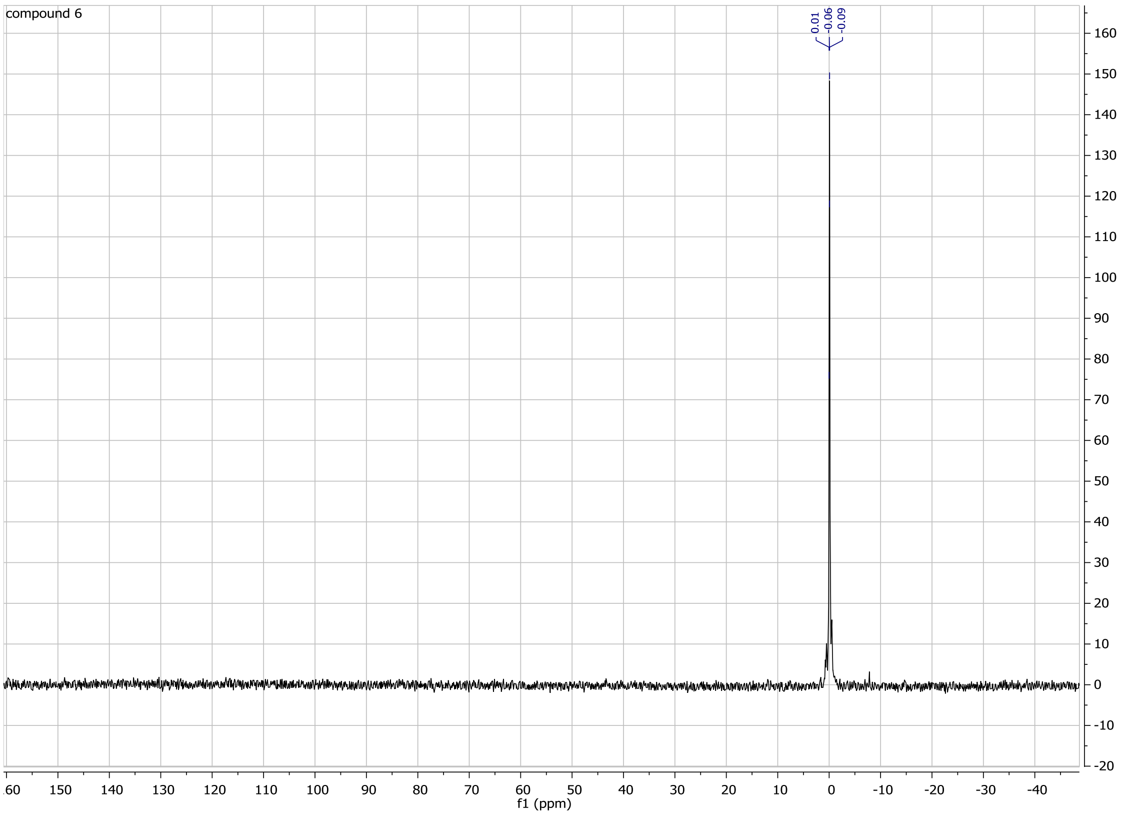

HSQC (600 MHz, $CDCl_3$)

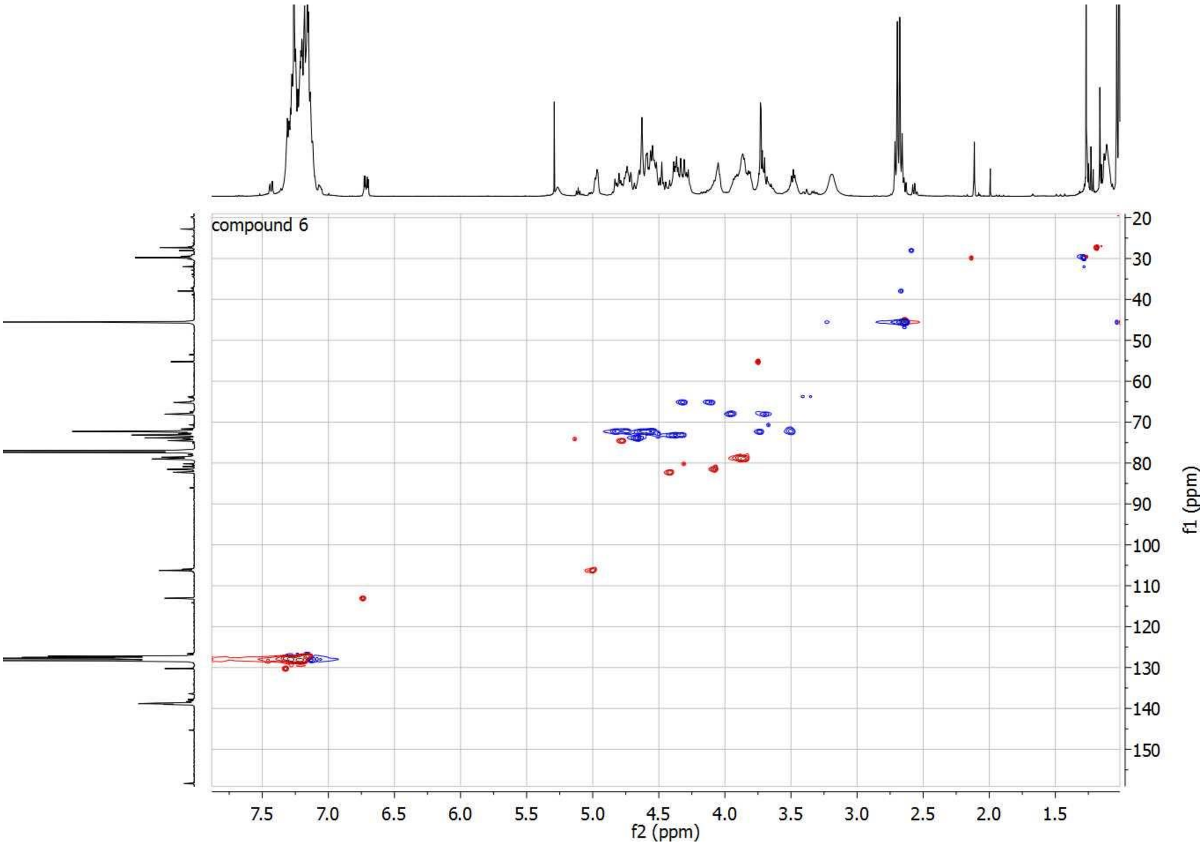

Compound 27

^{1}H NMR (600 MHz, $CDCl_3$)

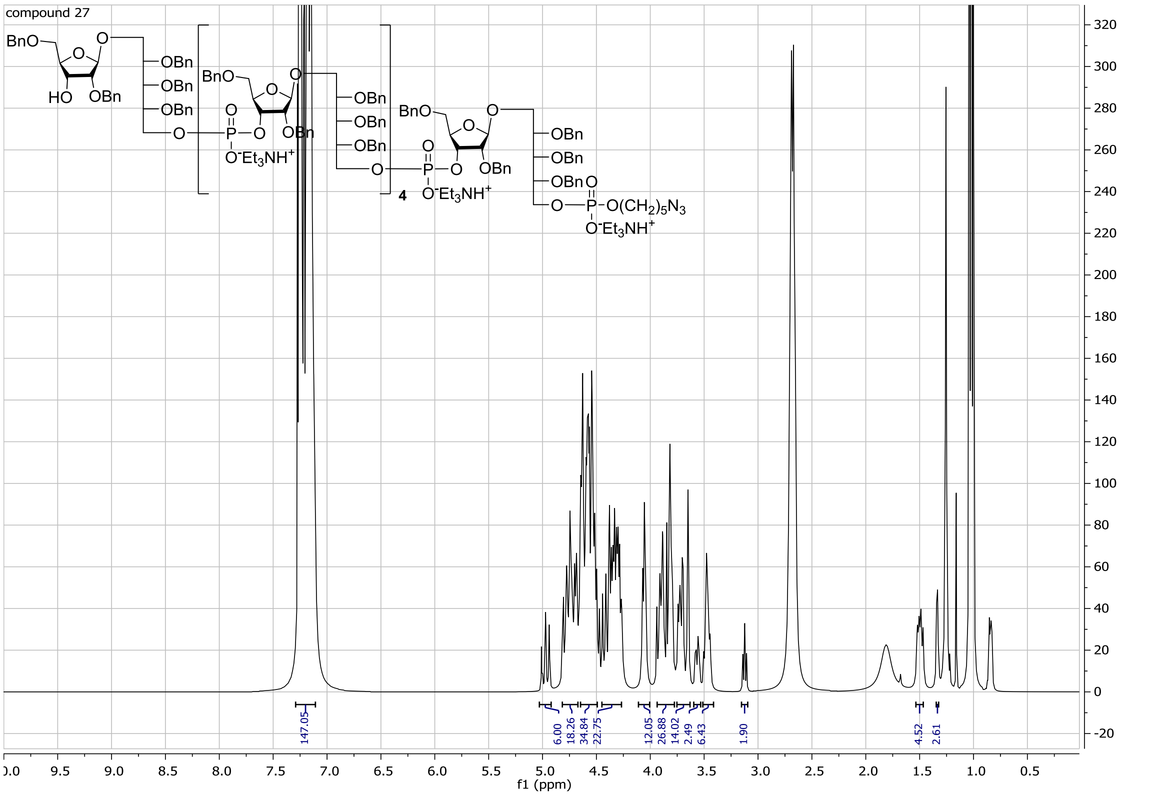

^{13}C NMR(151 MHz, $CDCl_3$)

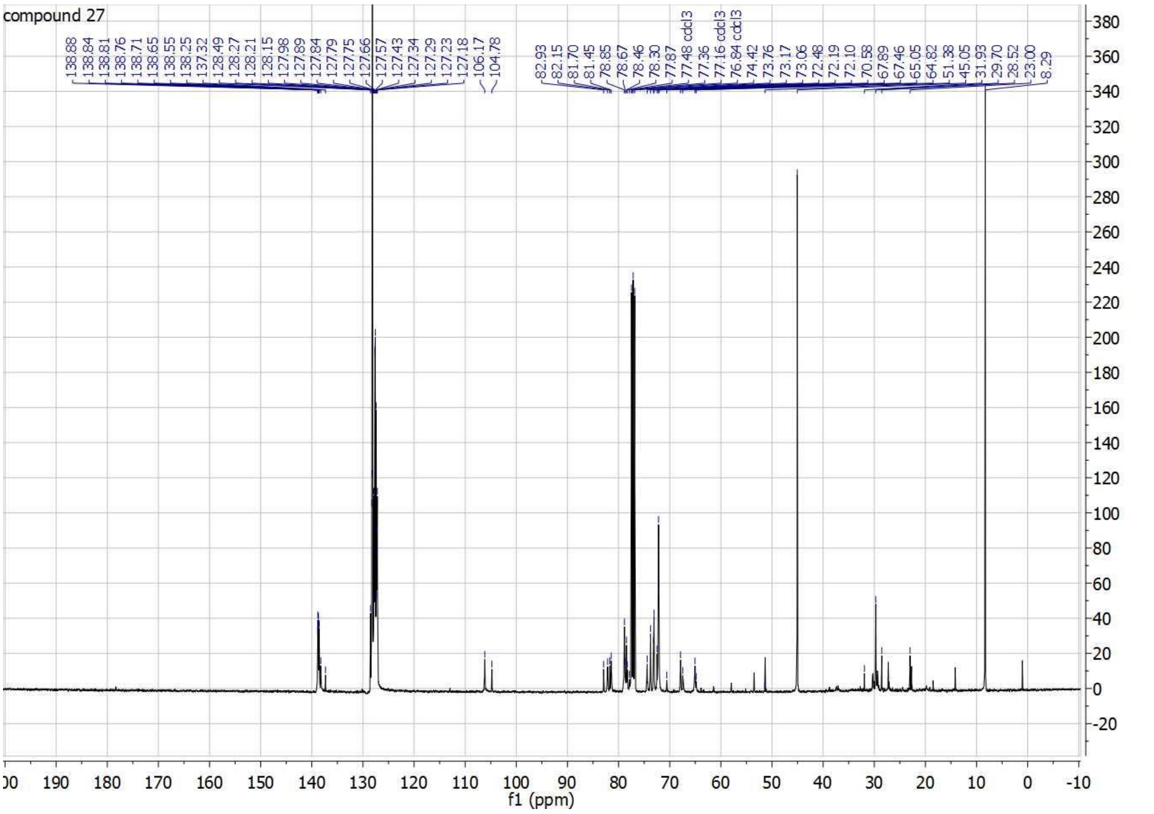

^{31}P NMR (243 MHz, $CDCl_3$)

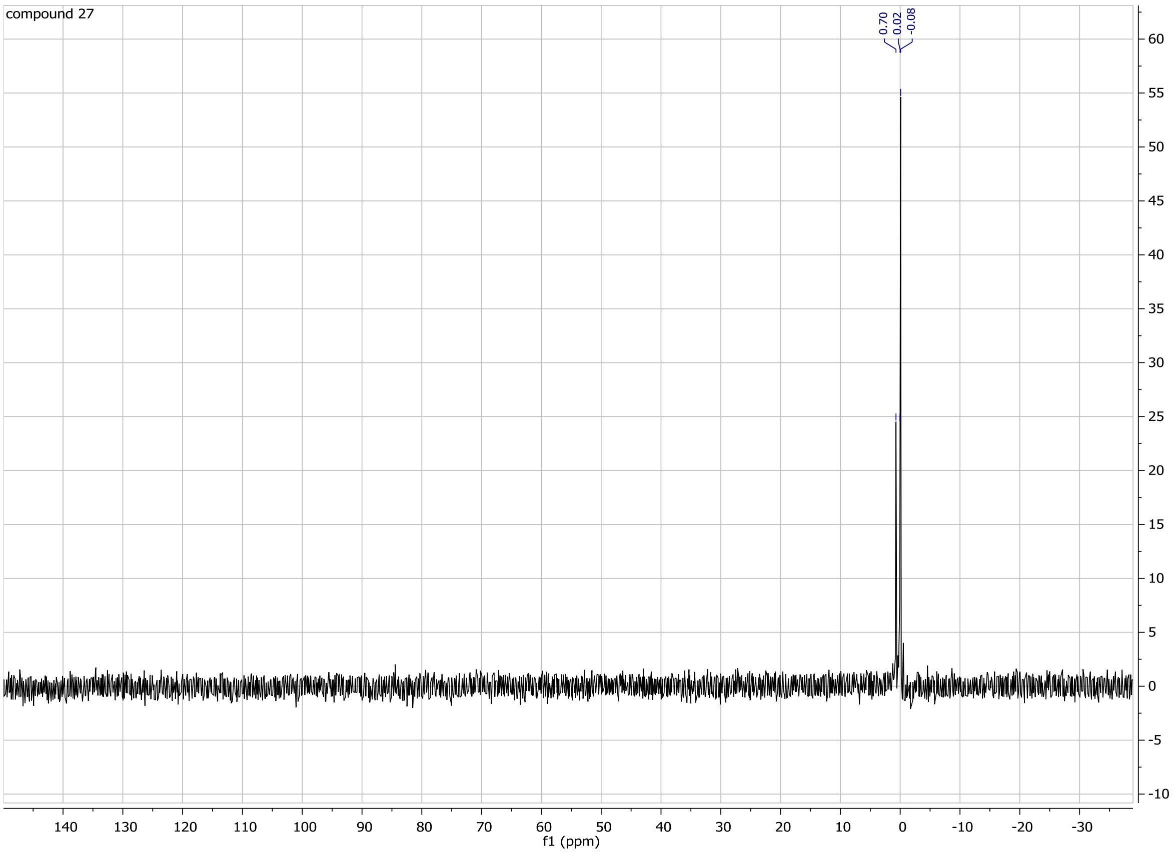

HSQC (600 MHz, $CDCl_3$)

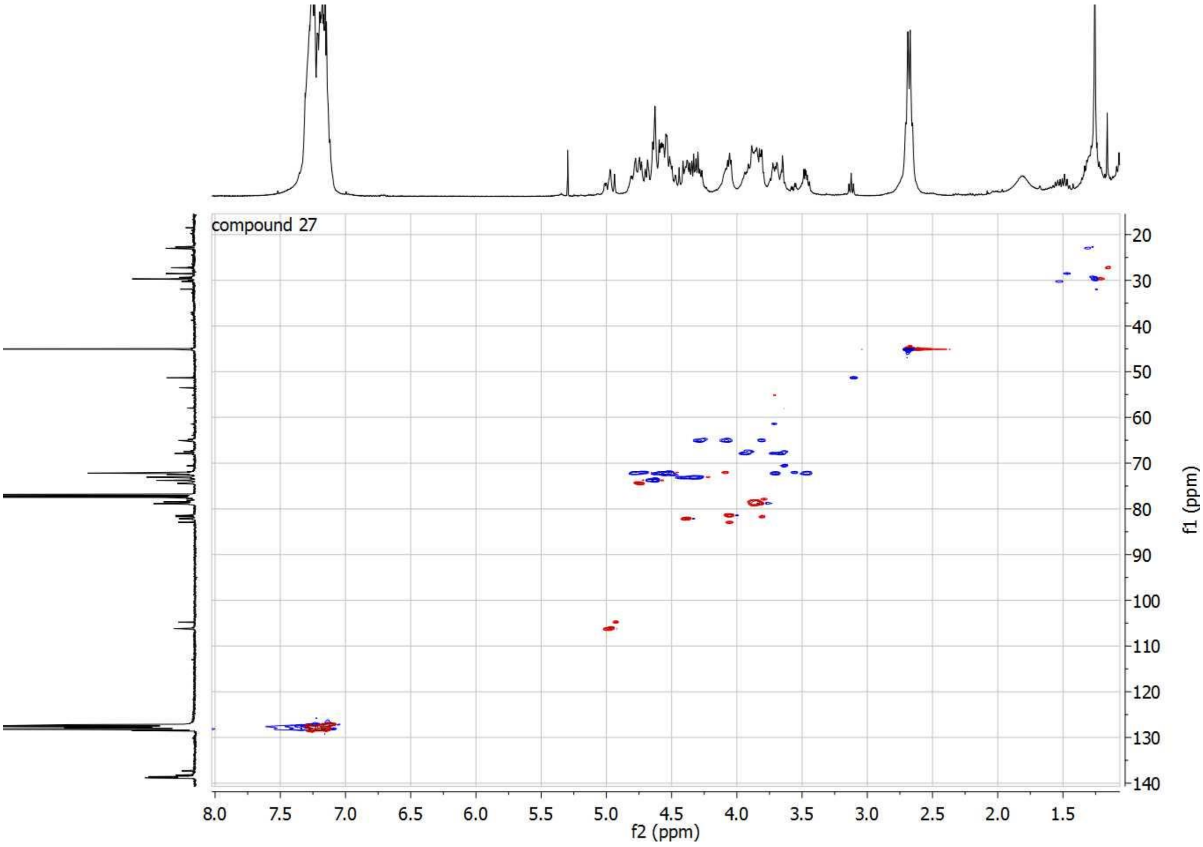

Compound 2

^{1}H NMR (600 MHz, D_2O)

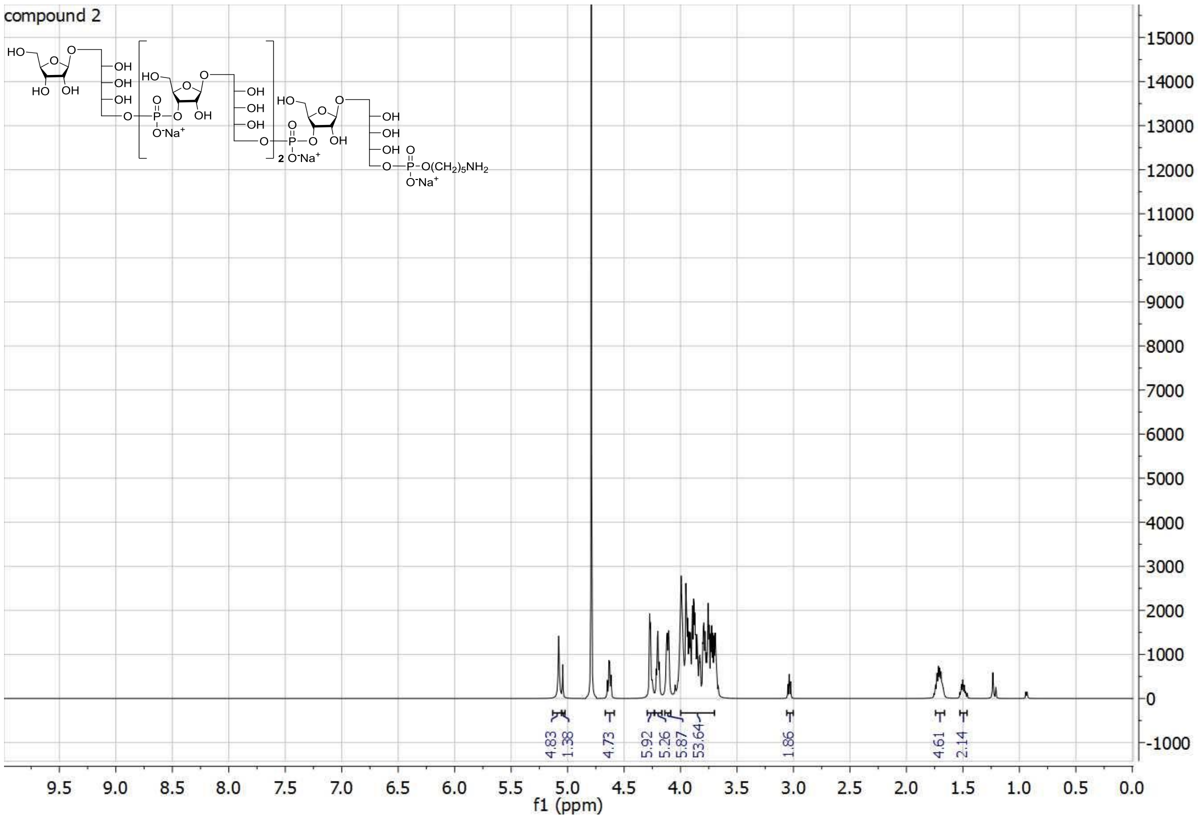

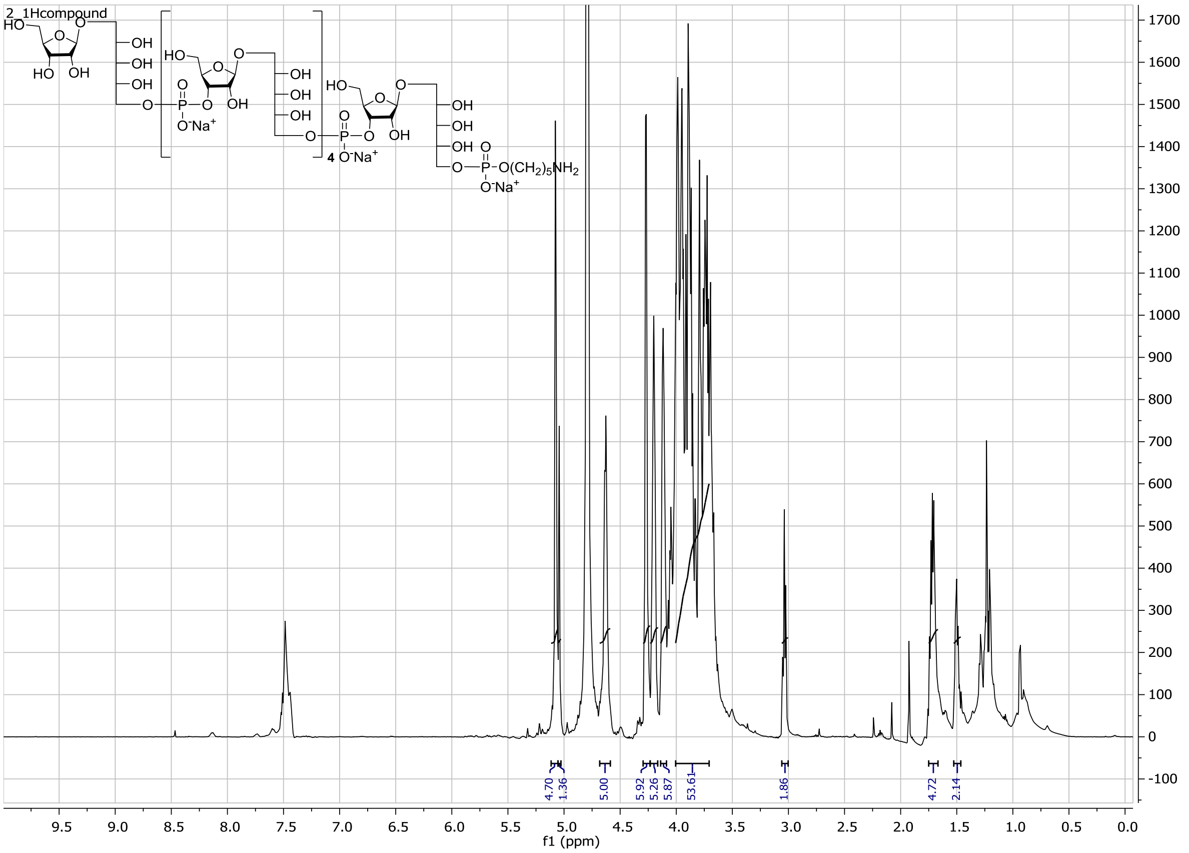

^{13}C NMR(151 MHz, D_2O)

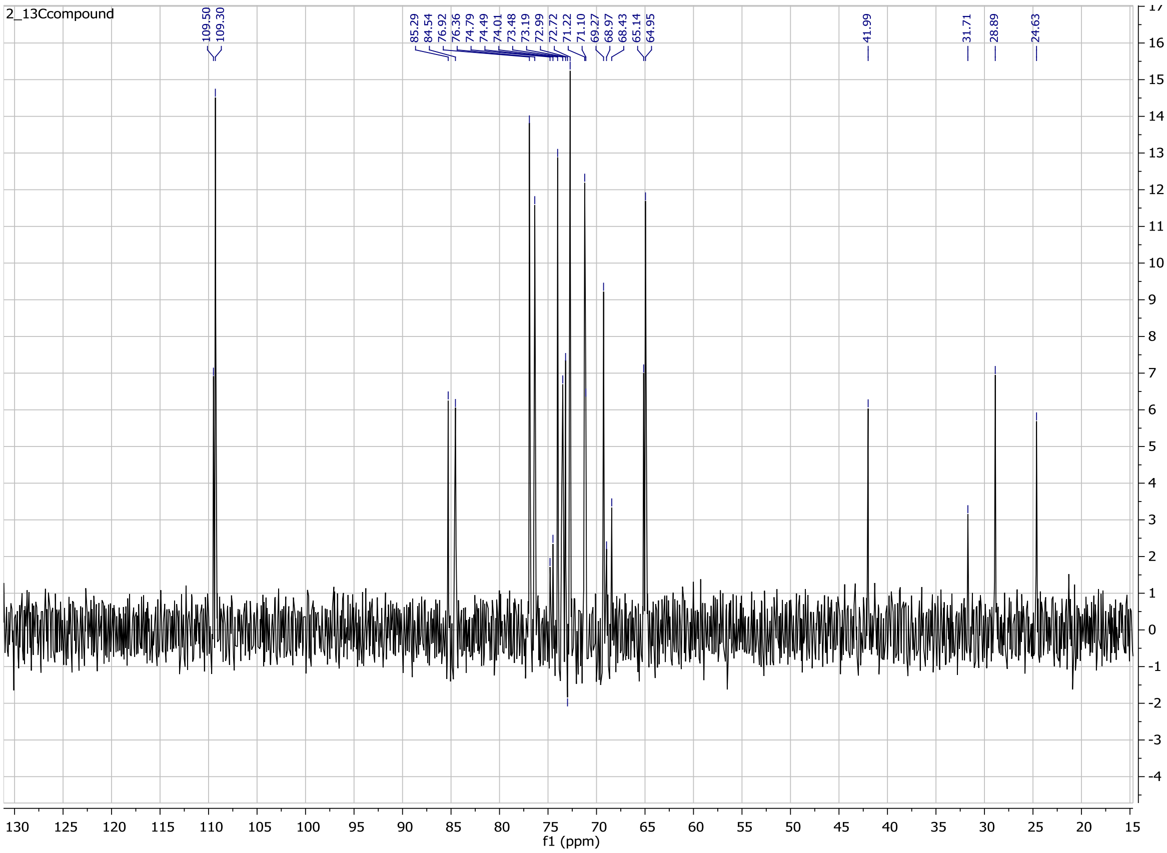

^{31}P NMR(243 MHz, D_2O)

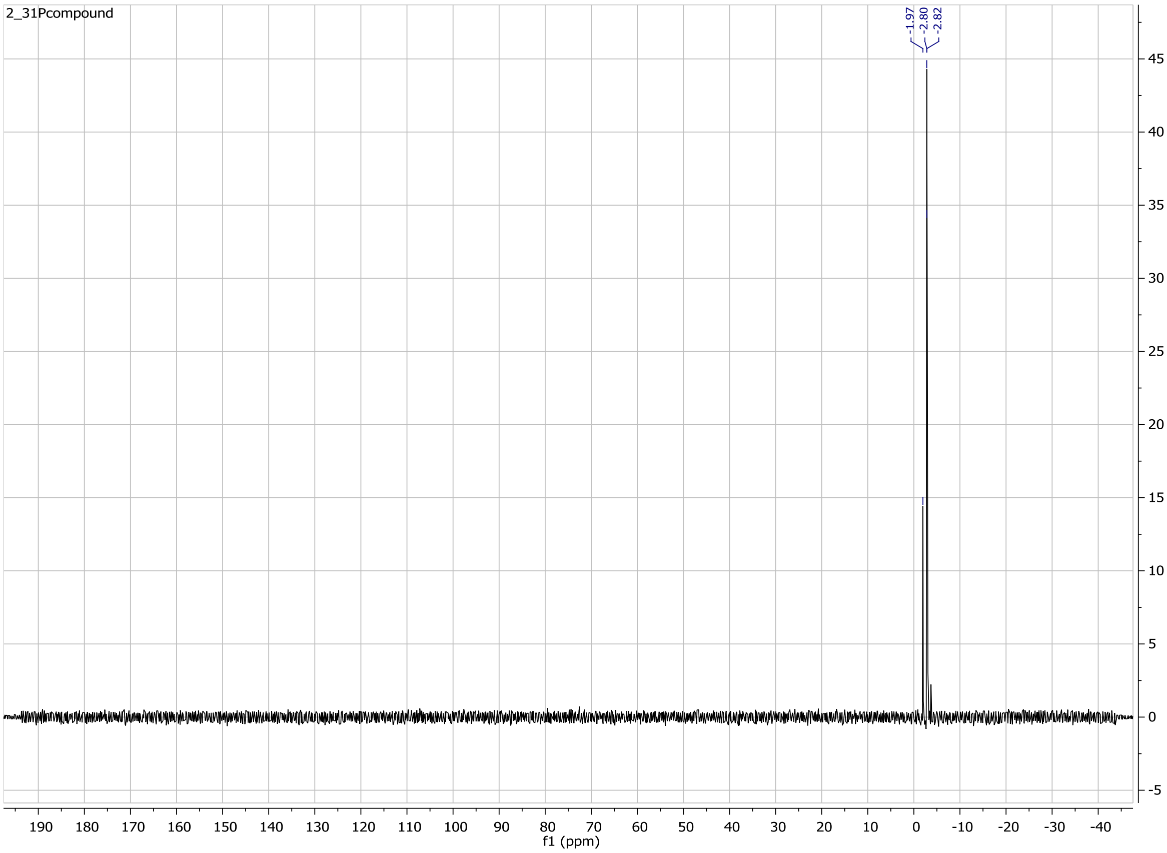

^{1}H-^{1}H COSY (600 MHz, D_2O)

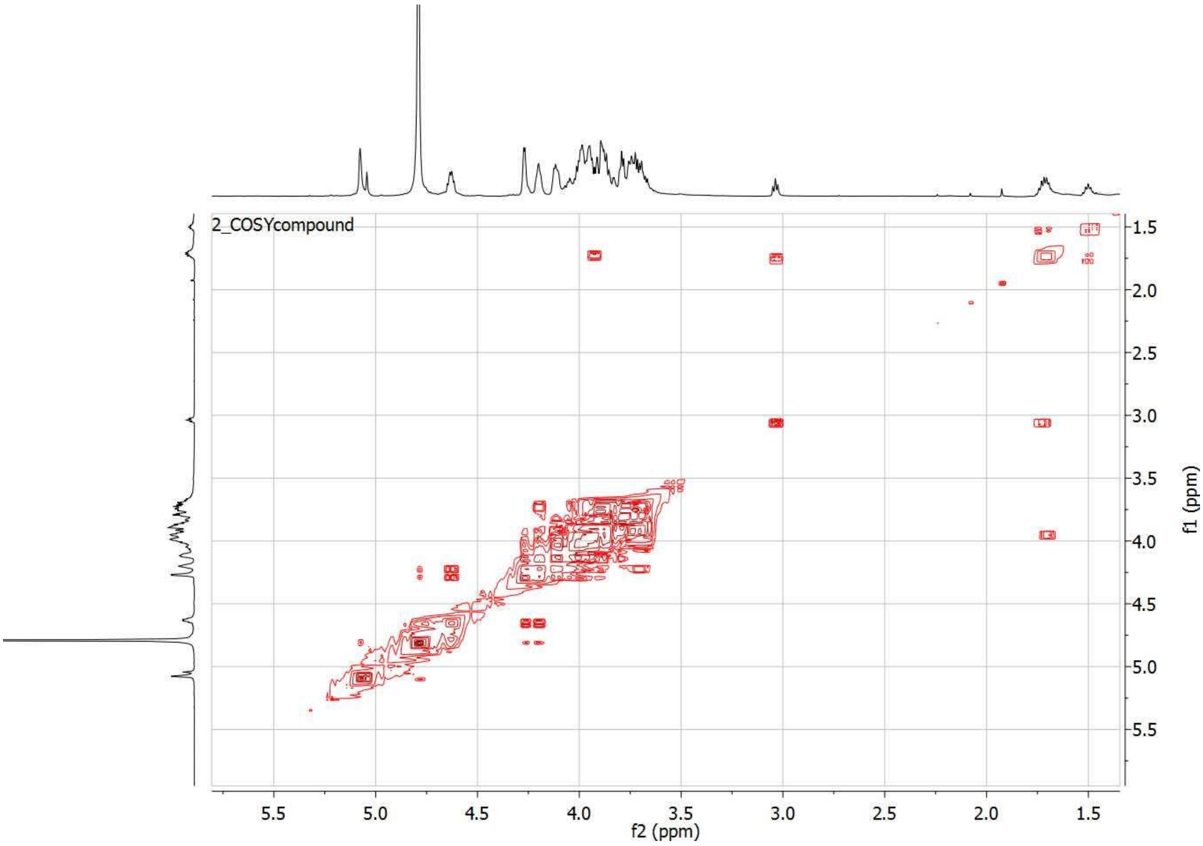

^{1}H-^{1}H TOCSY (600 MHz, D_2O)

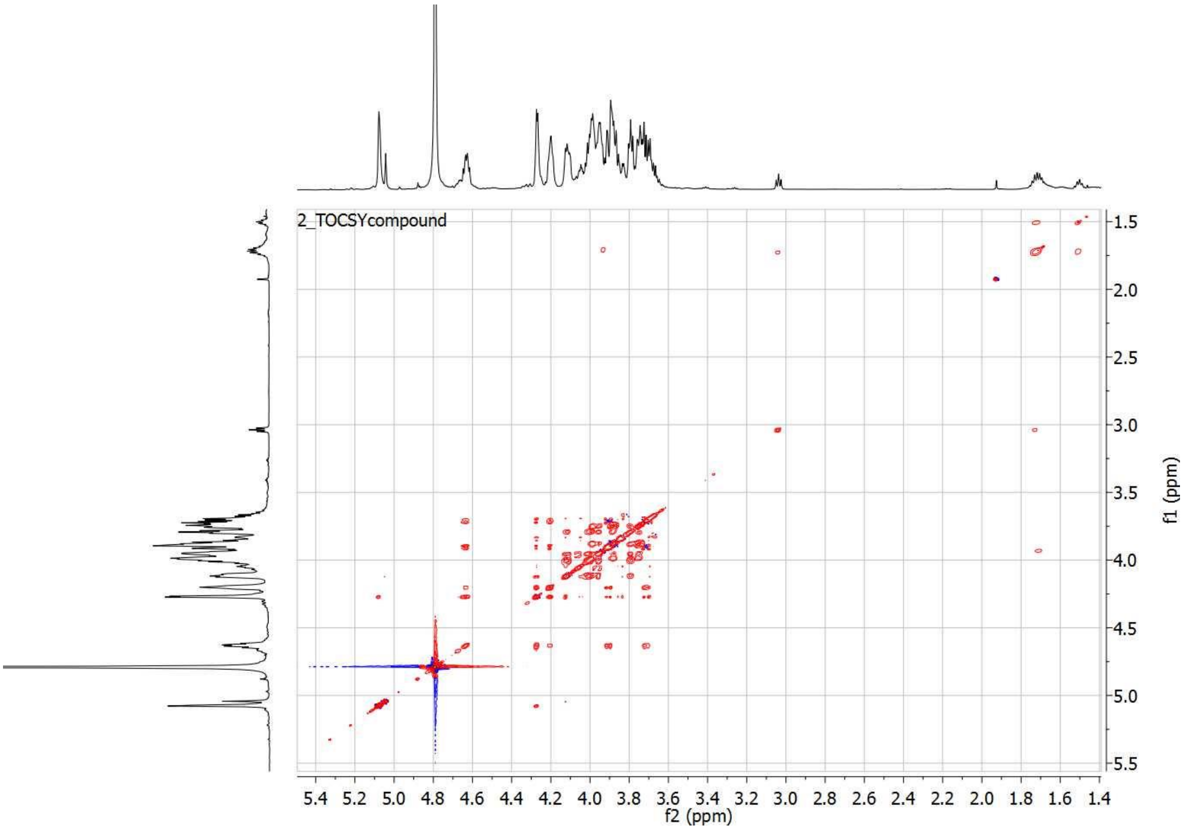

HSQC (600 MHz, D_2O)

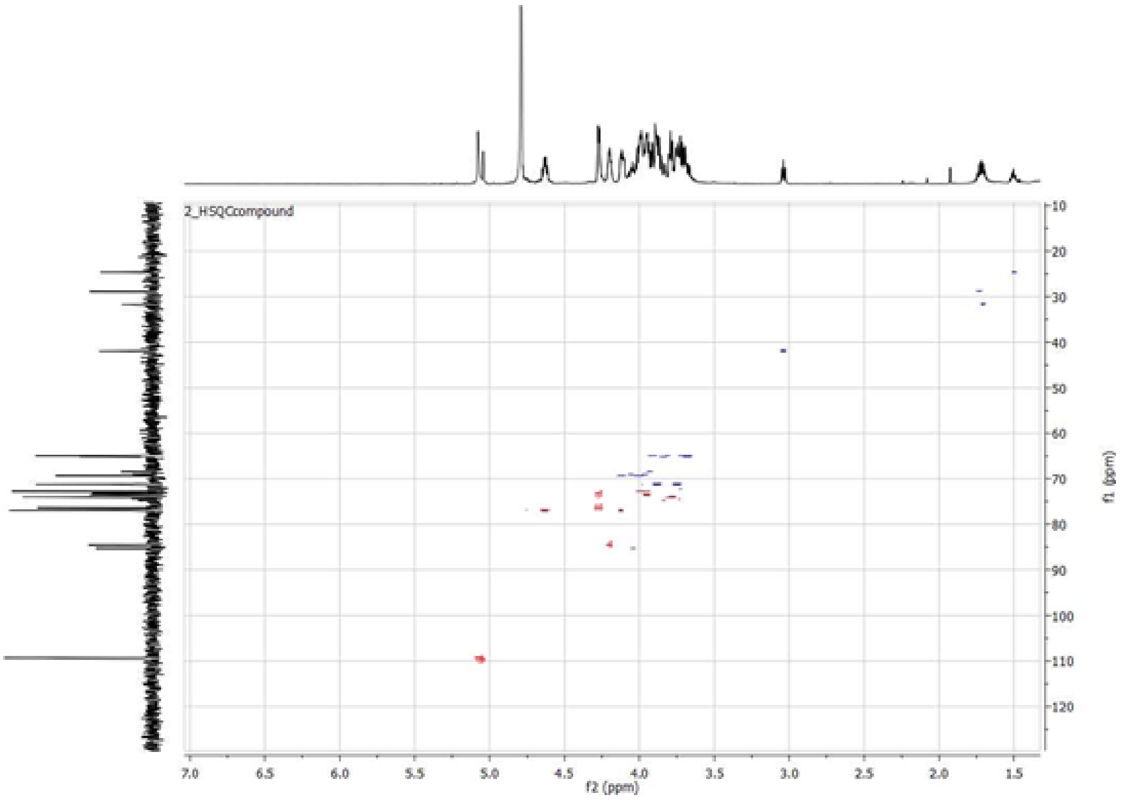

Compound 7

^{1}H NMR (600 MHz, $CDCl_3$)

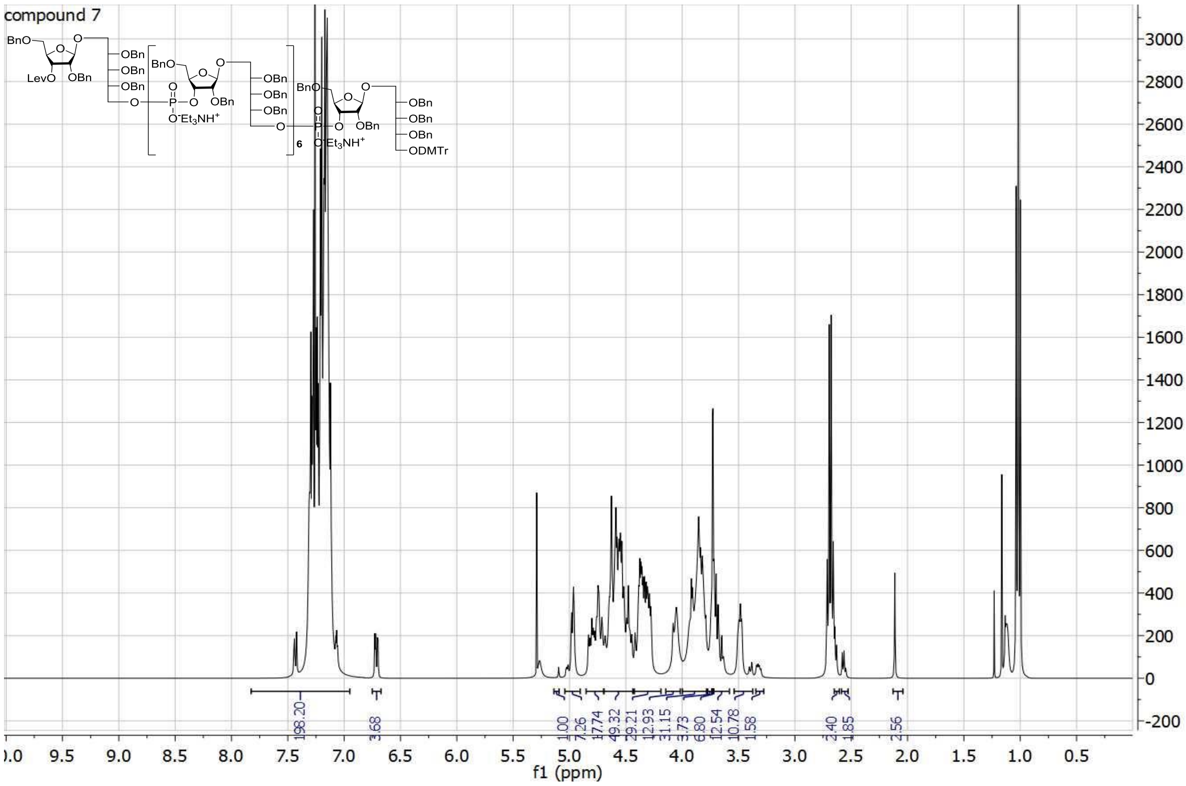

^{13}C NMR(151 MHz, $CDCl_3$)

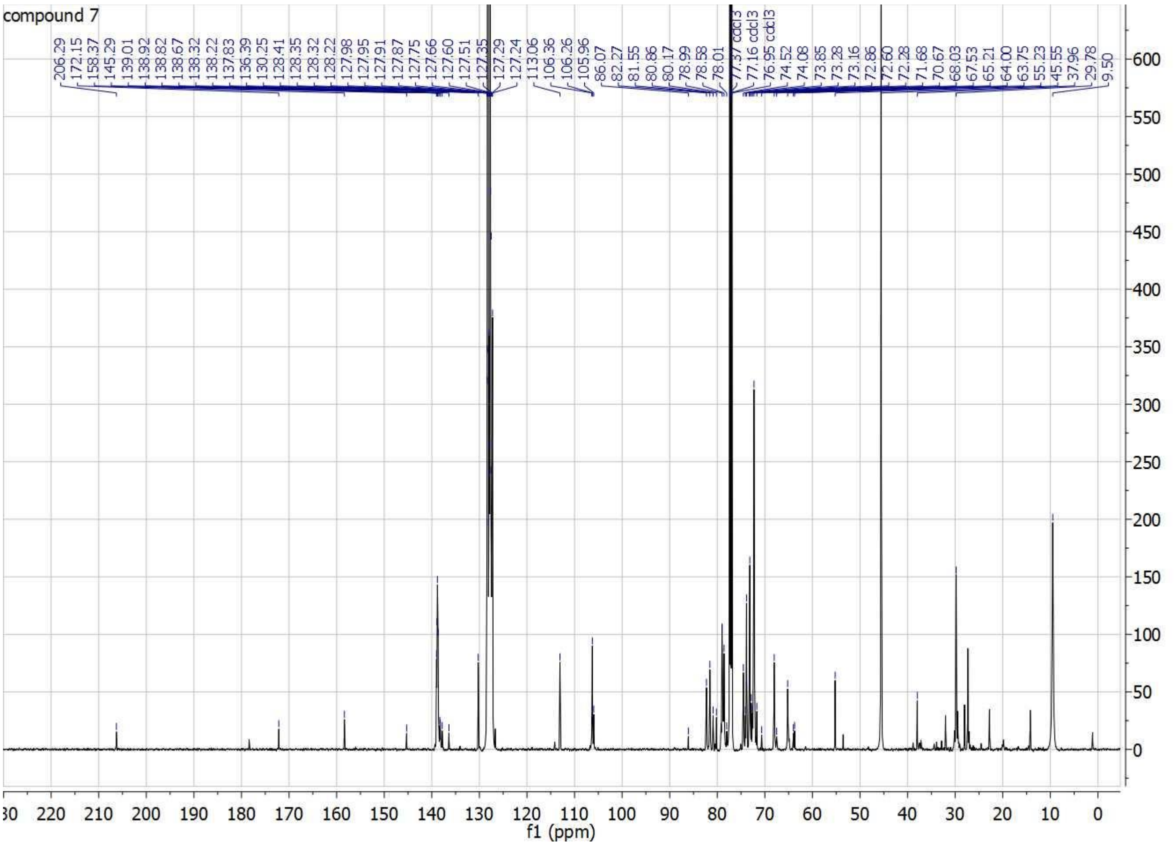

^{31}P NMR (243 MHz, $CDCl_3$)

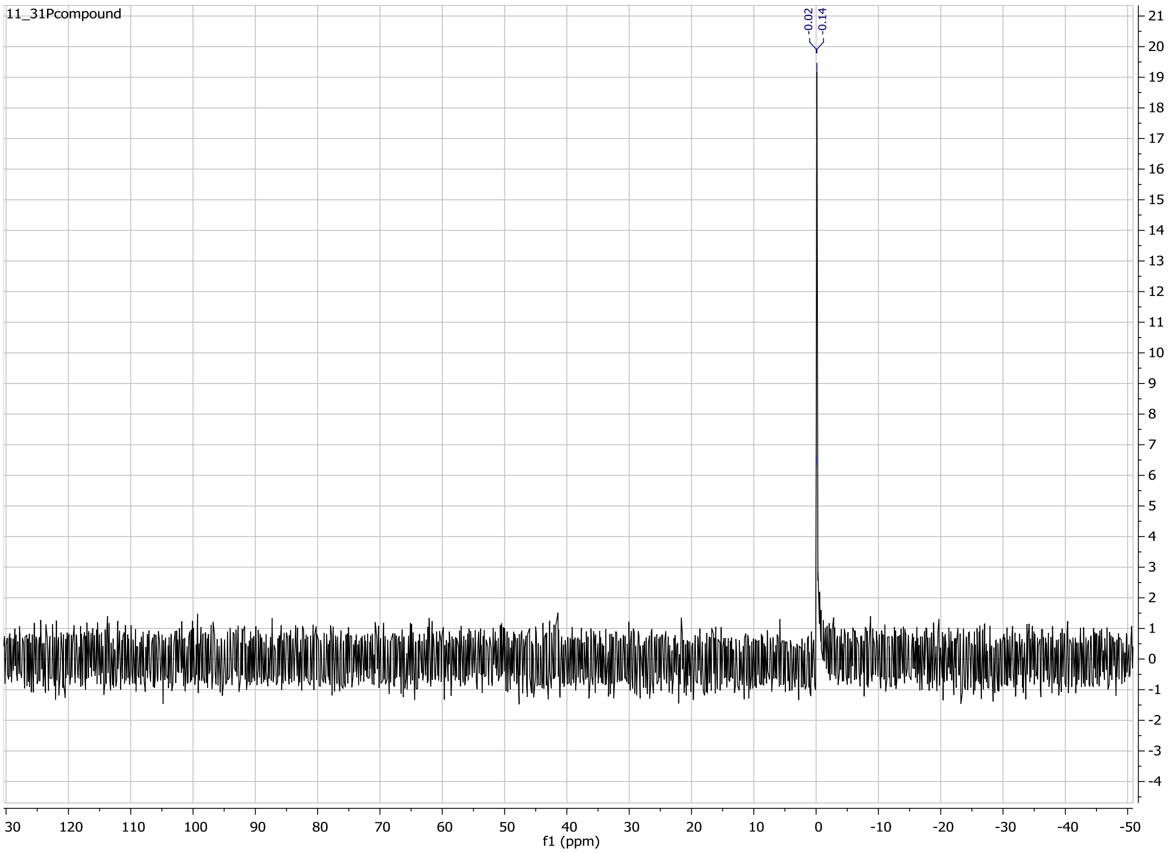

HSQC (600 MHz, $CDCl_3$)

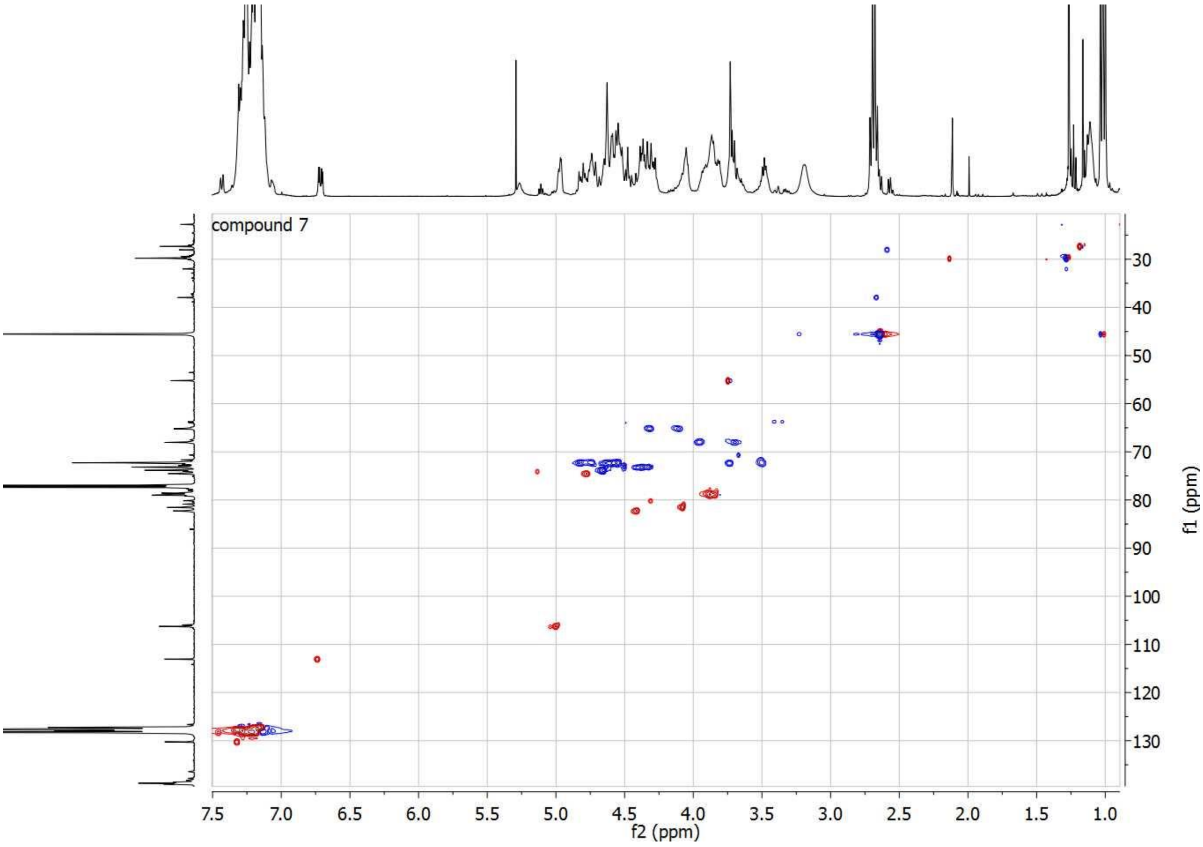

Compound 28

^{1}H NMR (600 MHz, CDCl$_3$)

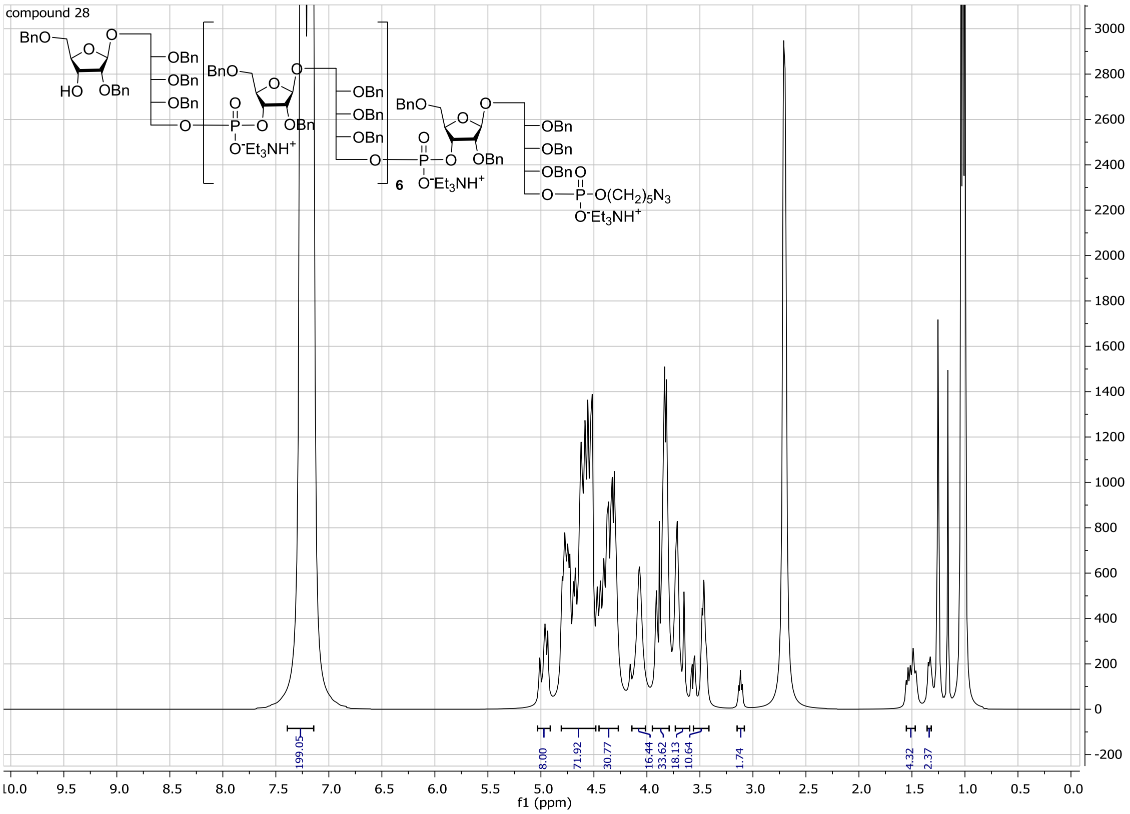

^{13}C NMR(151 MHz, CDCl$_3$)

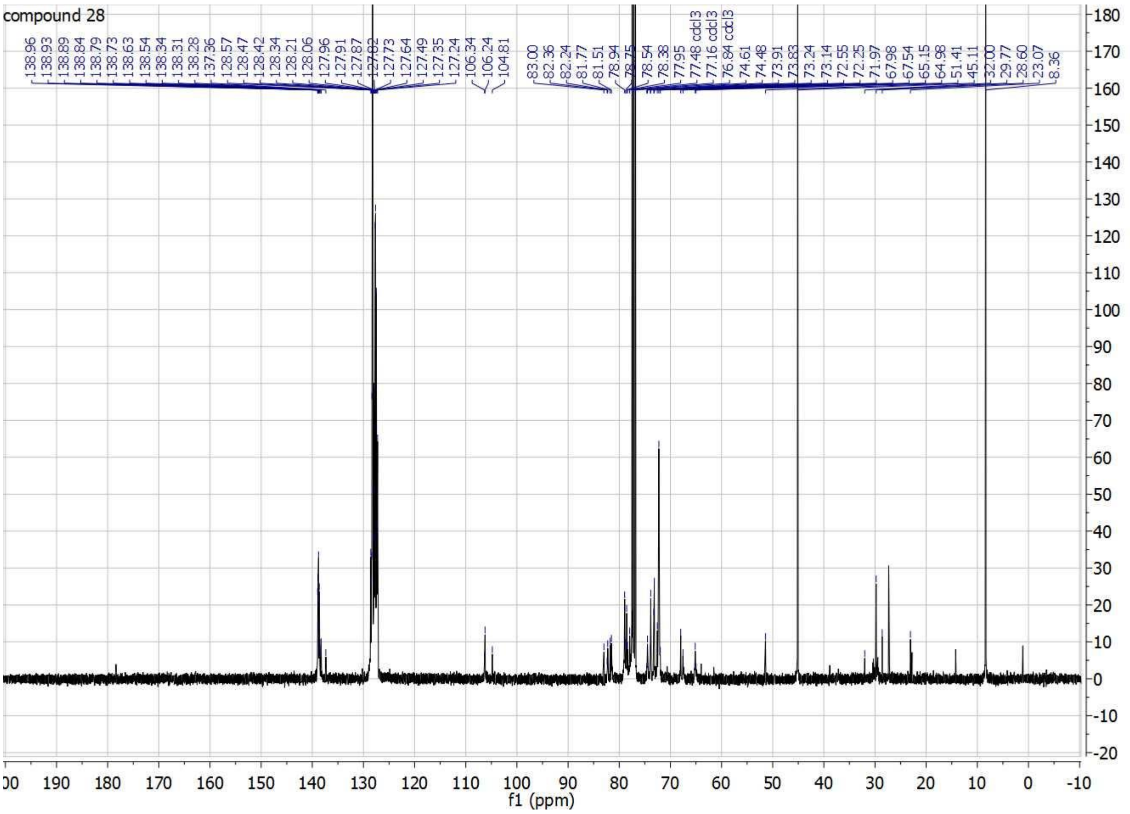

^{31}P NMR(243 MHz, $CDCl_3$)

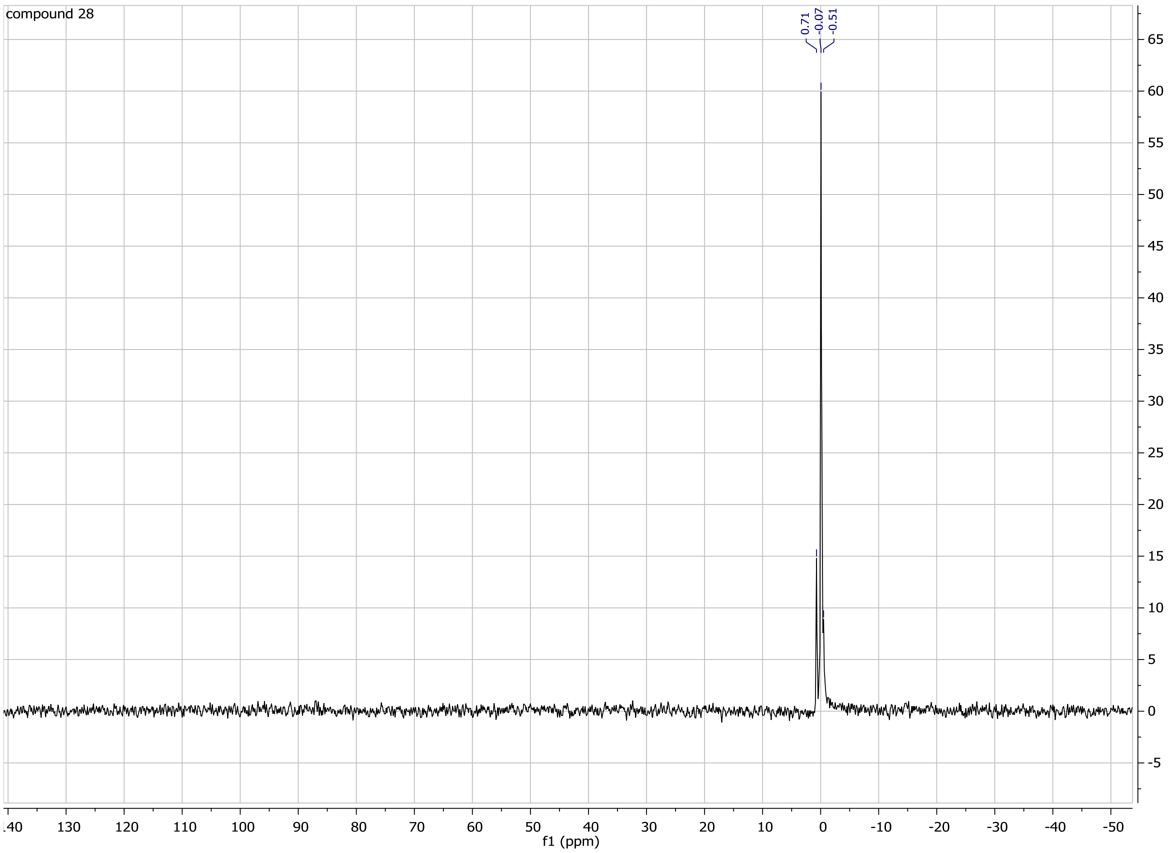

HSQC (600 MHz, $CDCl_3$)

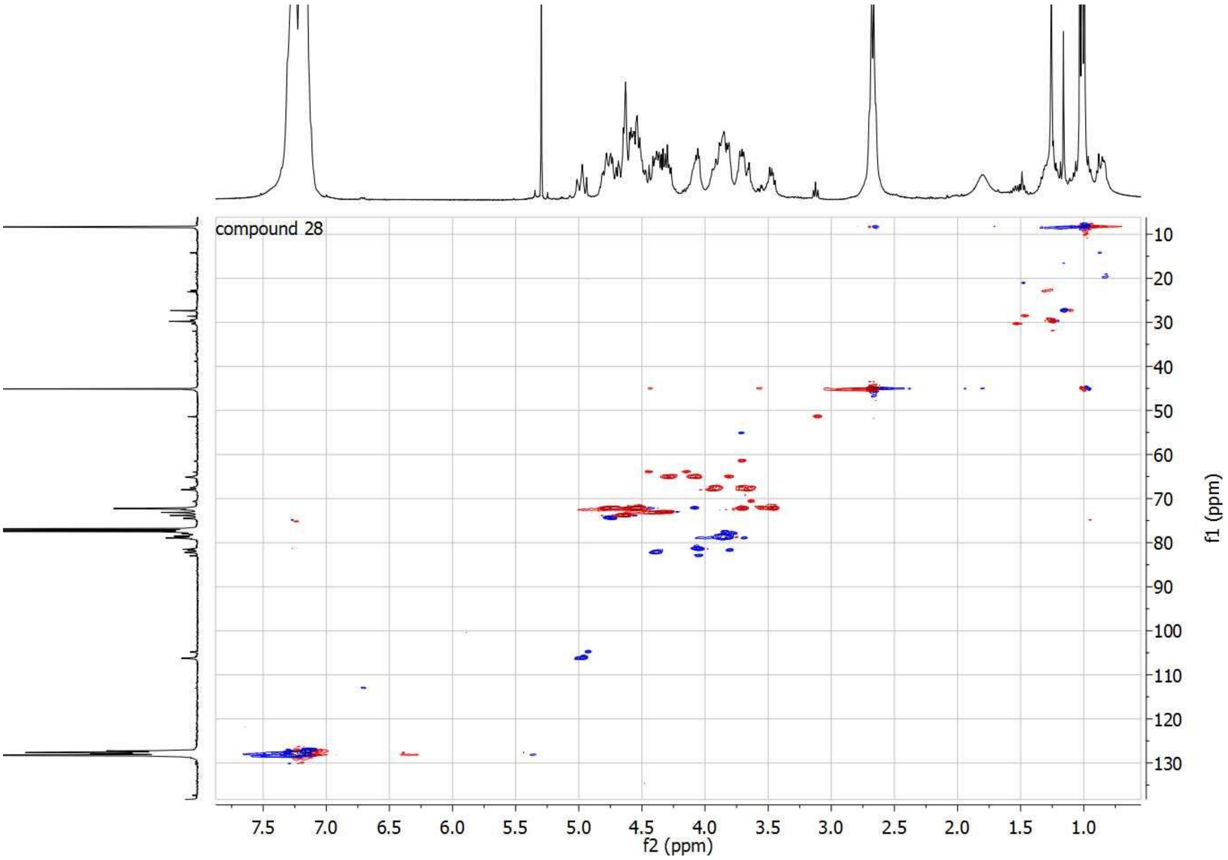

Compound 3

^{1}H NMR (600 MHz, D_2O)

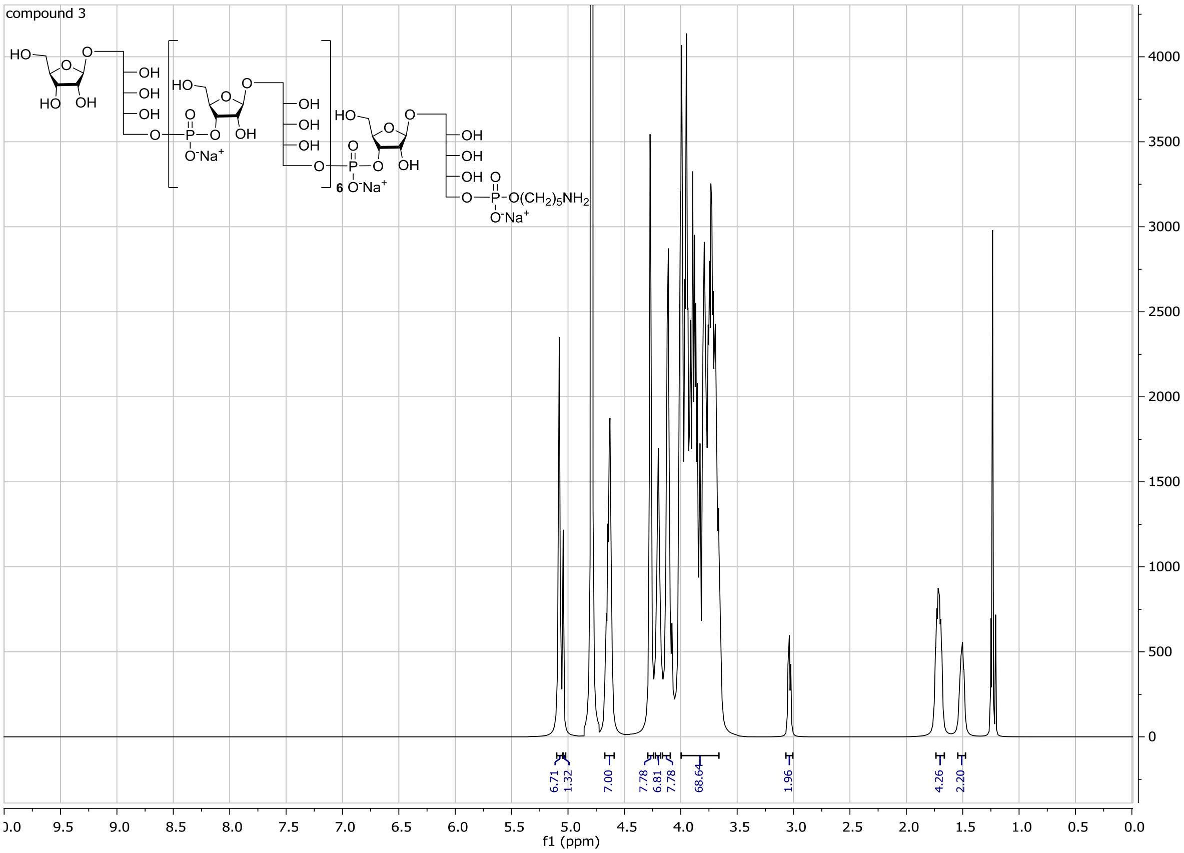

^{13}C NMR (151 MHz, D_2O)

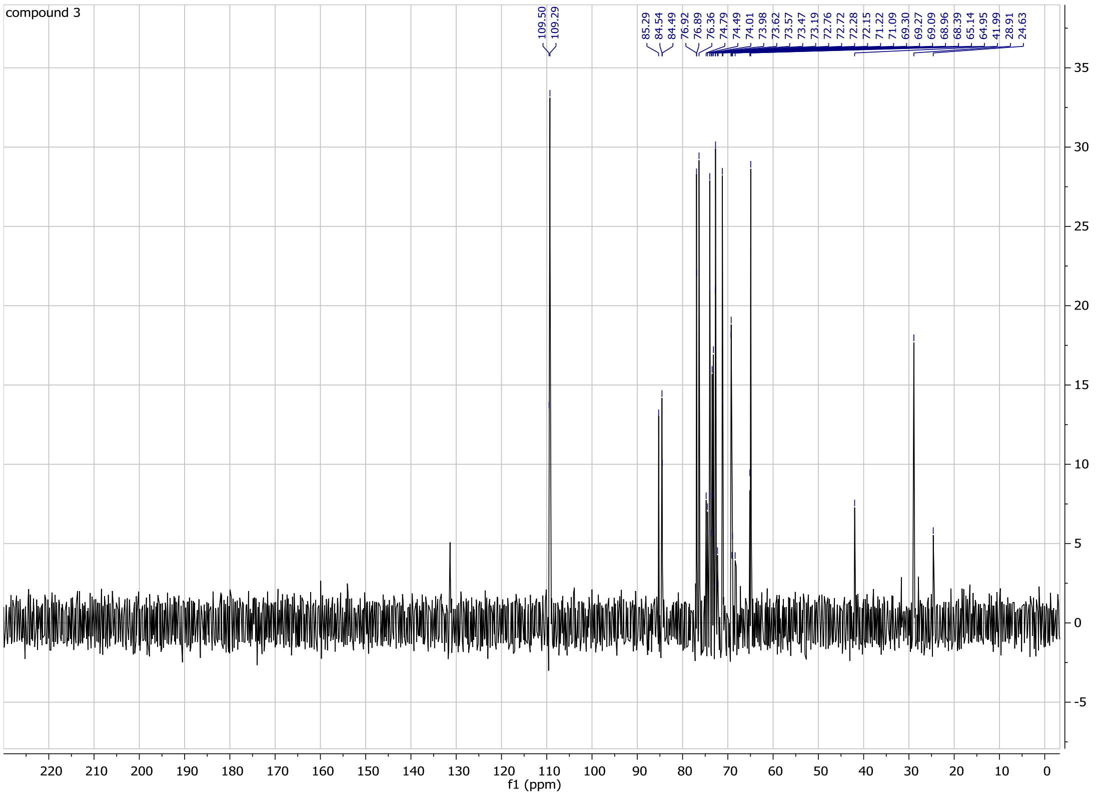

^{31}P NMR (243 MHz, D_2O)

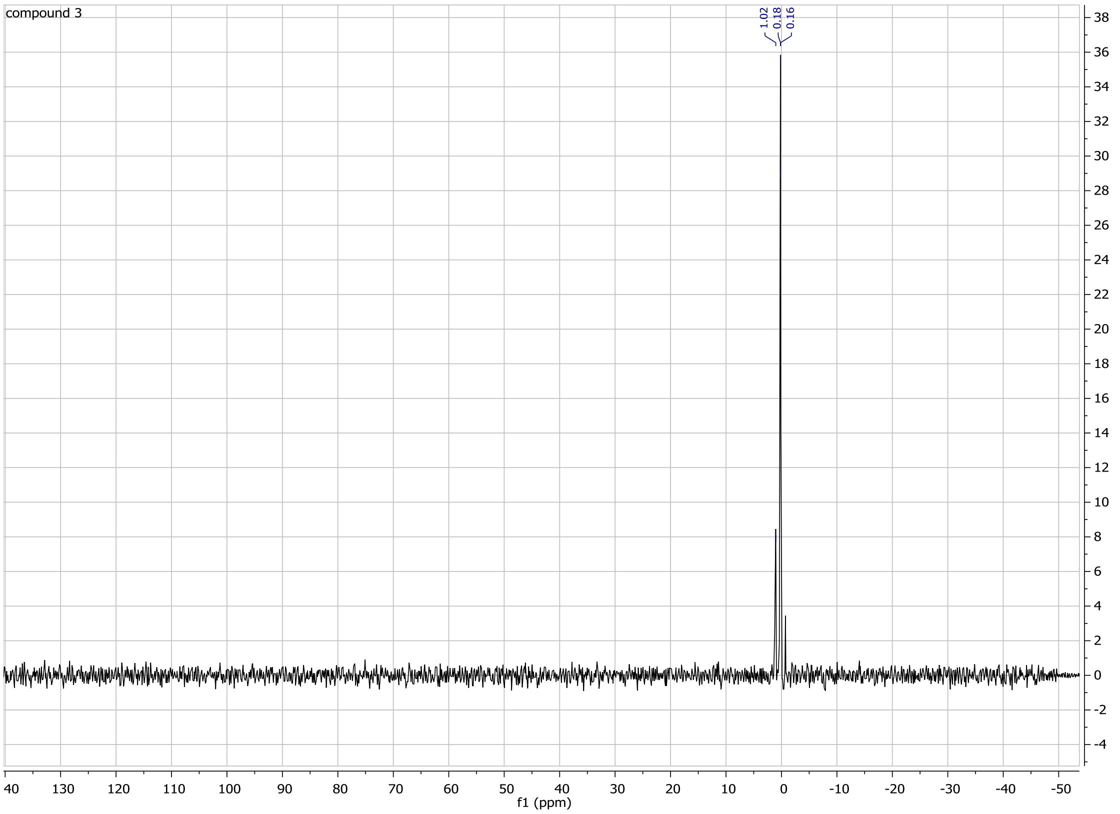

^{1}H-^{1}H COSY (600 MHz, D_2O)

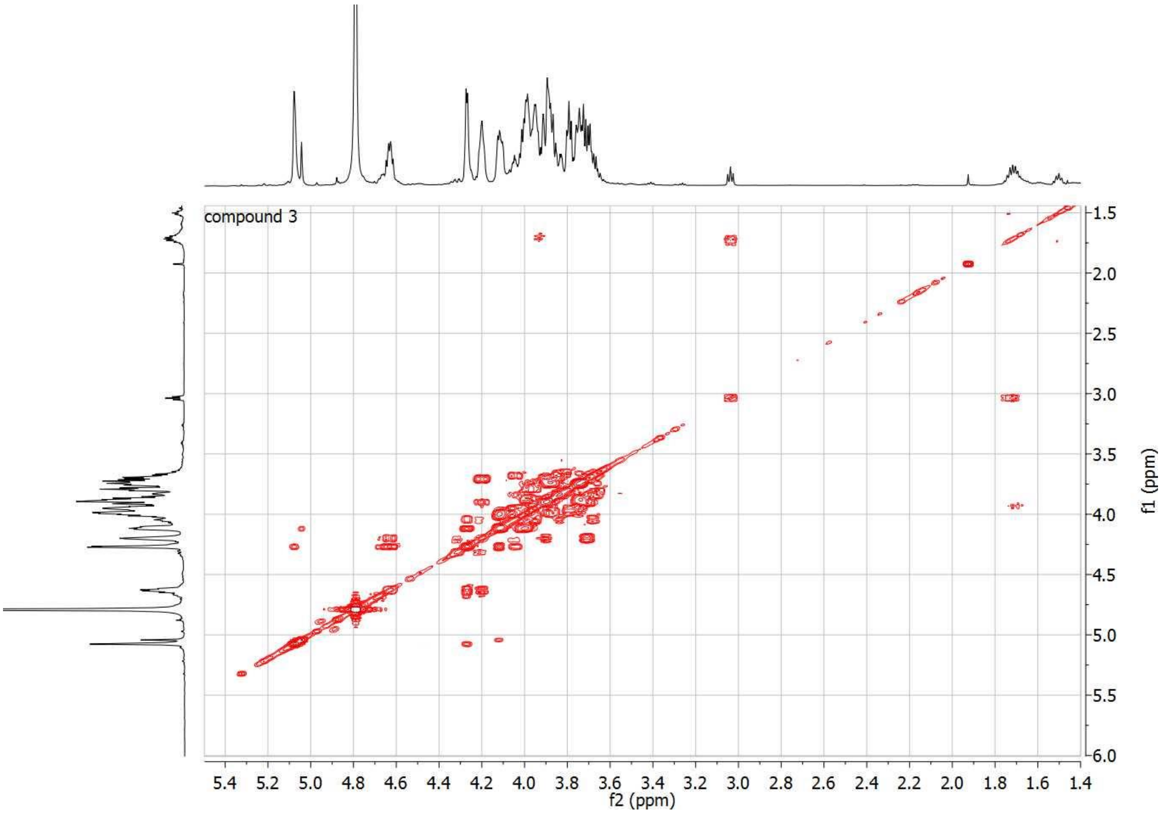

^{1}H-^{1}H TOCSY (600 MHz, D_2O)

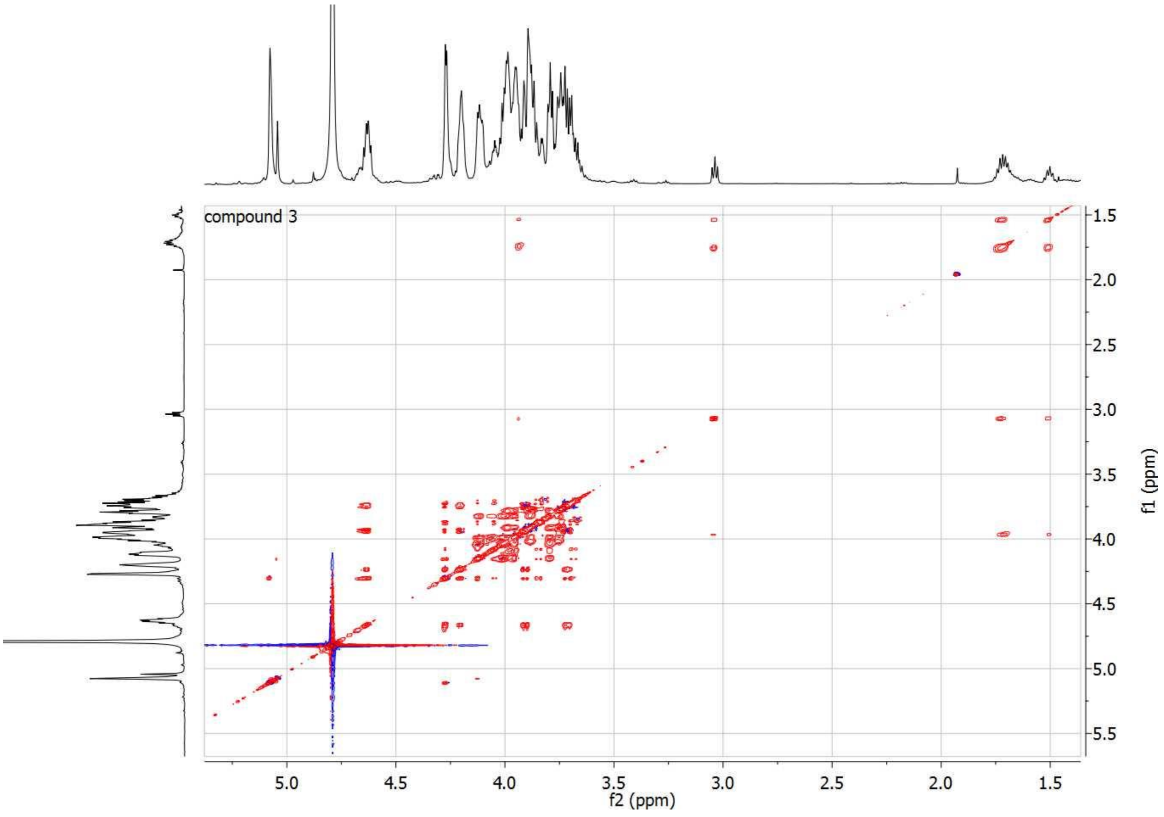

HSQC (600 MHz, D_2O)

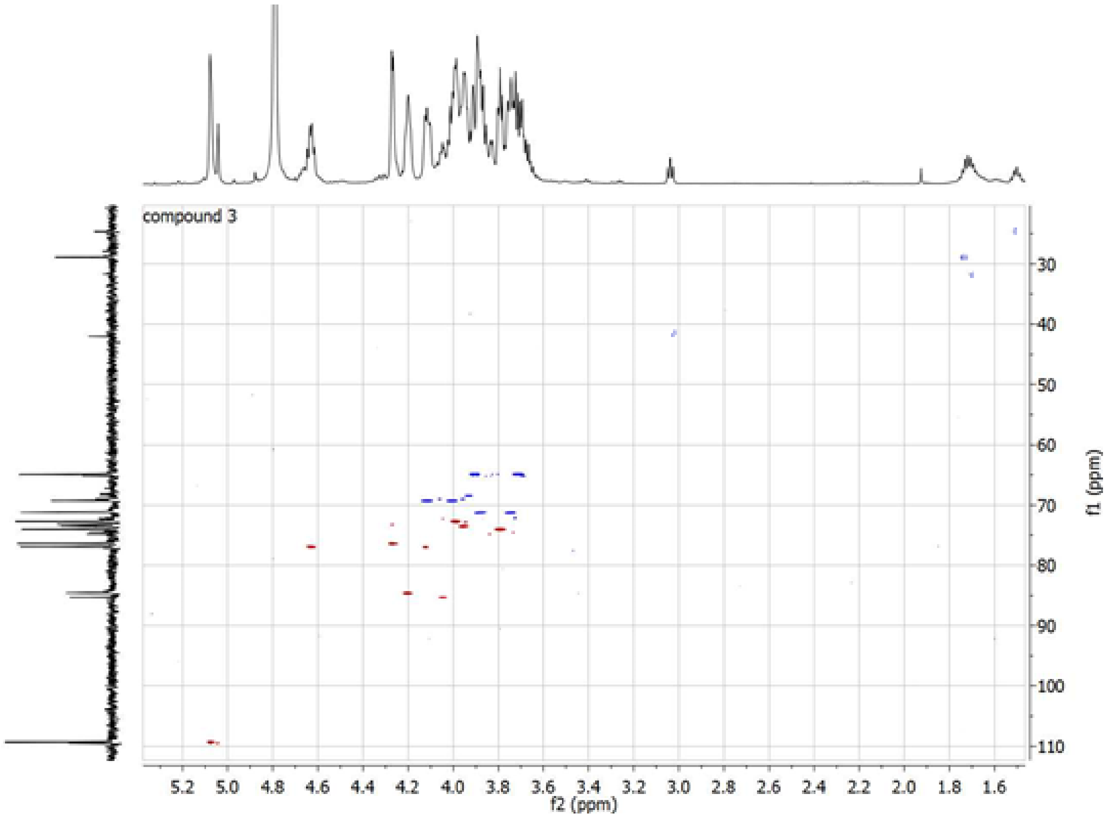

Compound 8

^{1}H NMR (600 MHz, $CDCl_3$)

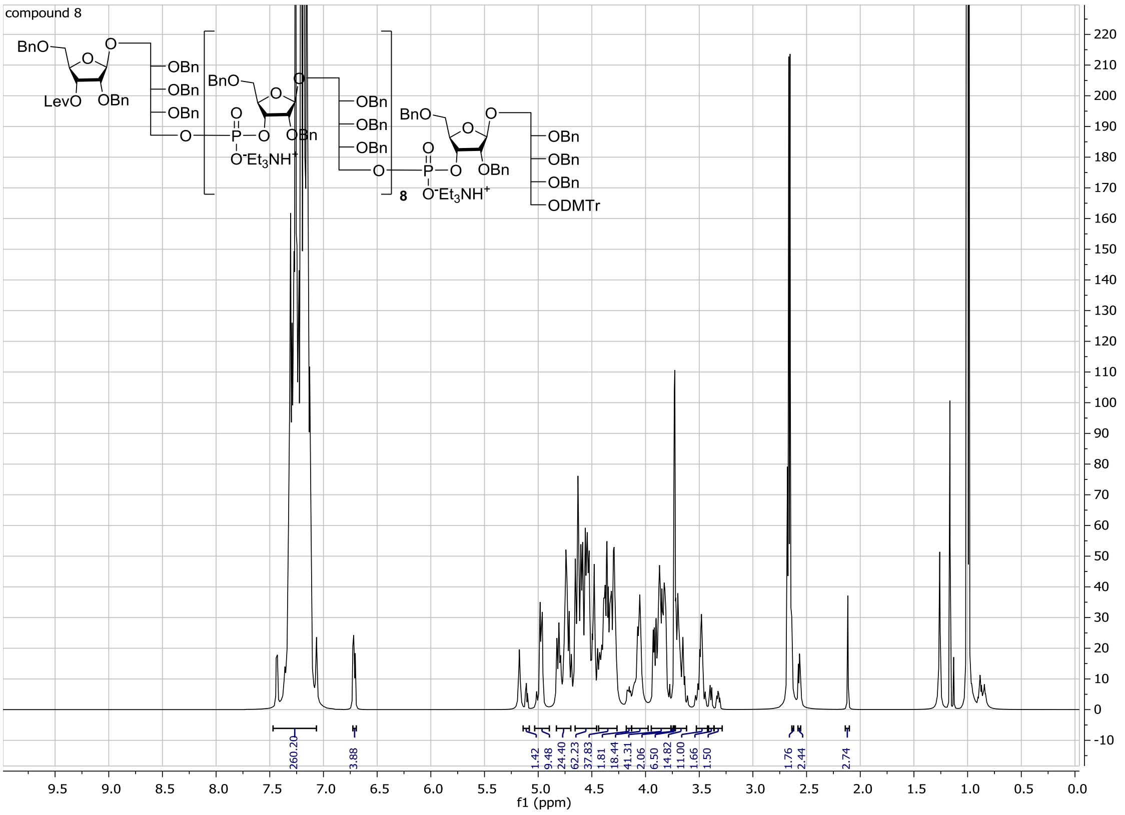

^{13}C NMR (151 MHz, $CDCl_3$)

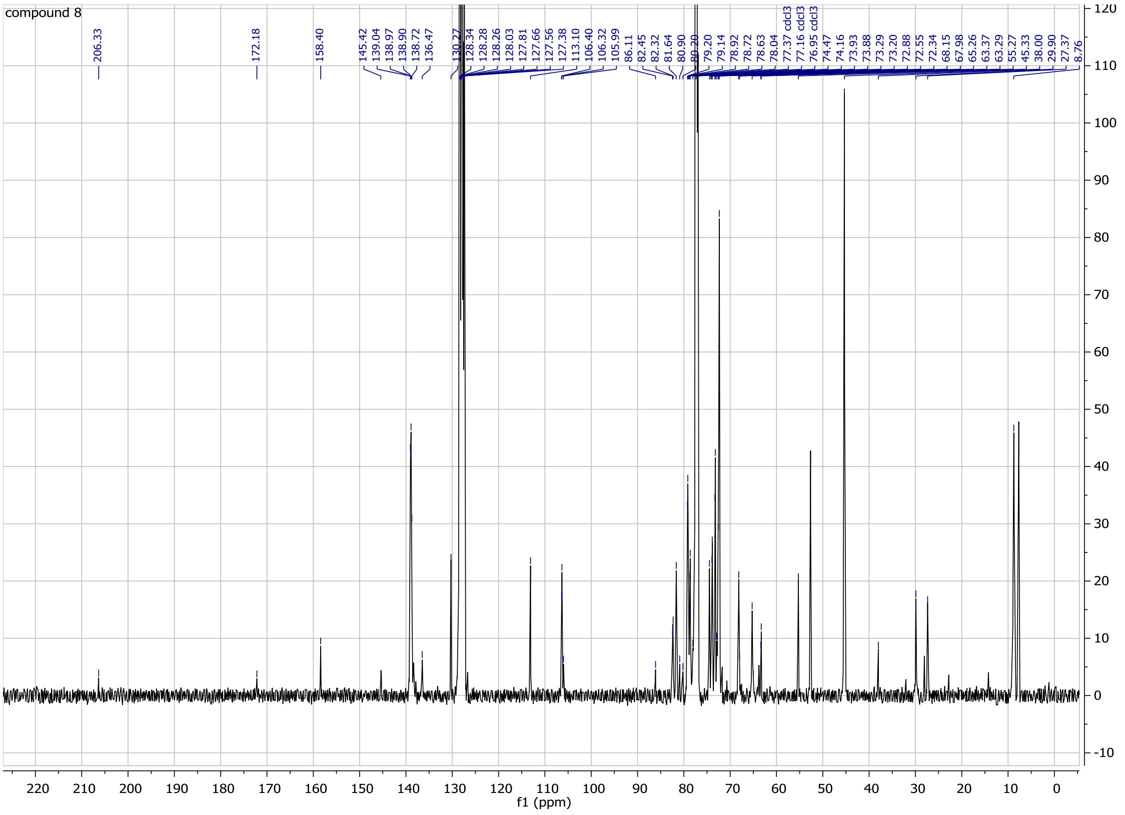

^{31}P NMR (243 MHz, $CDCl_3$)

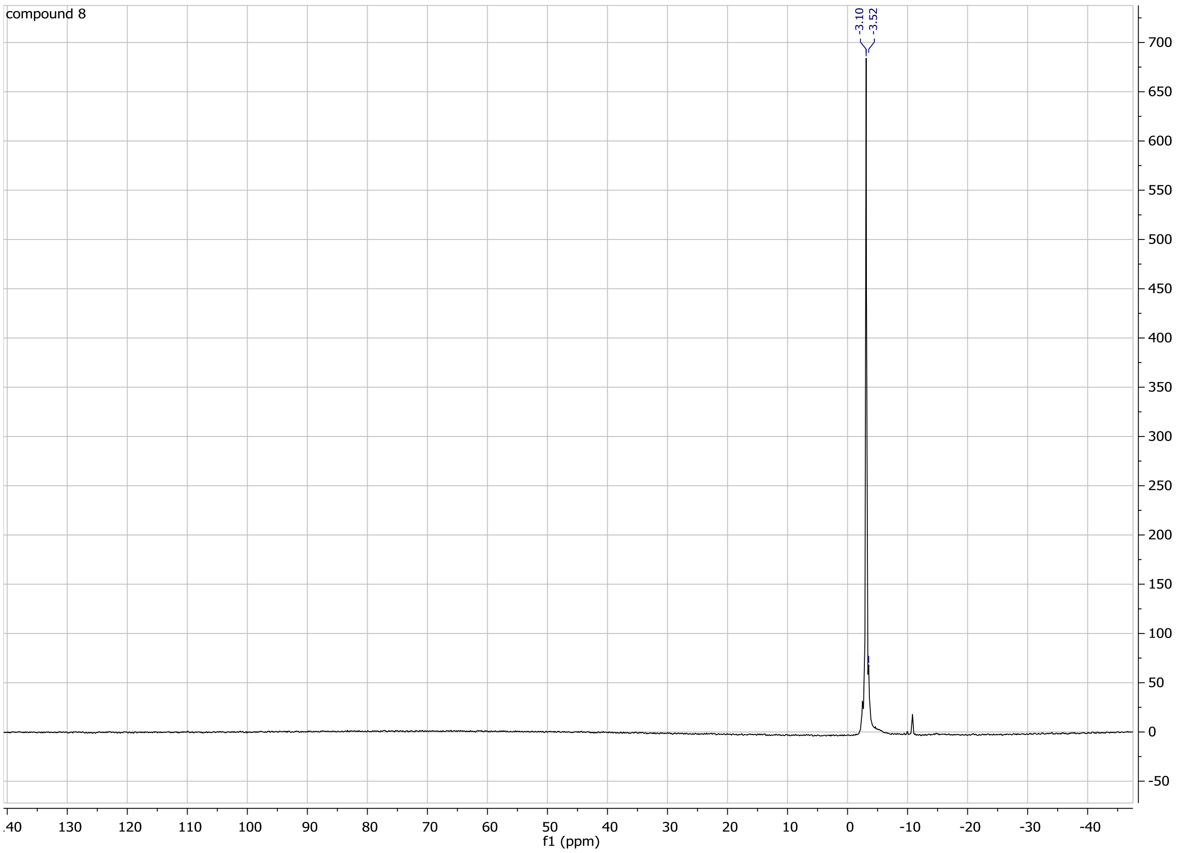

HSQC (600 MHz, $CDCl_3$)

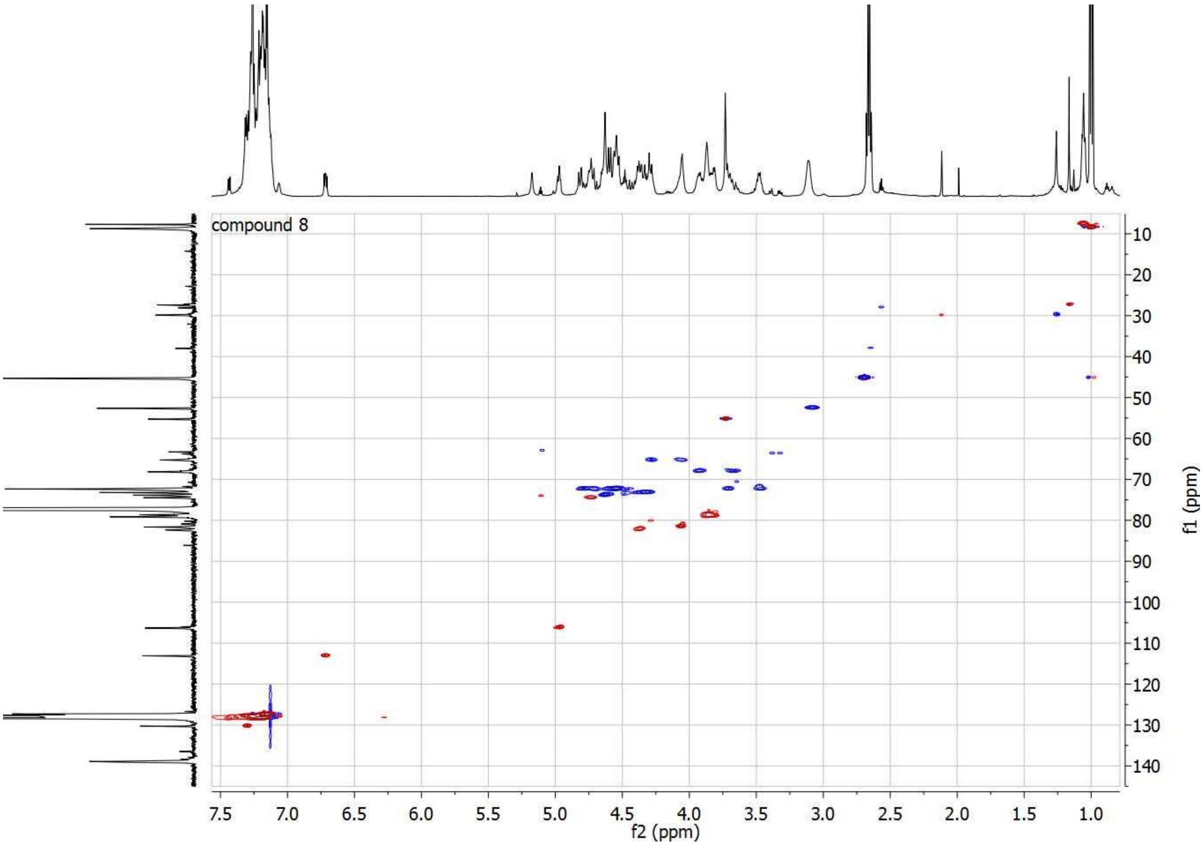

Compound 29

^{1}H NMR (600 MHz, $CDCl_3$)

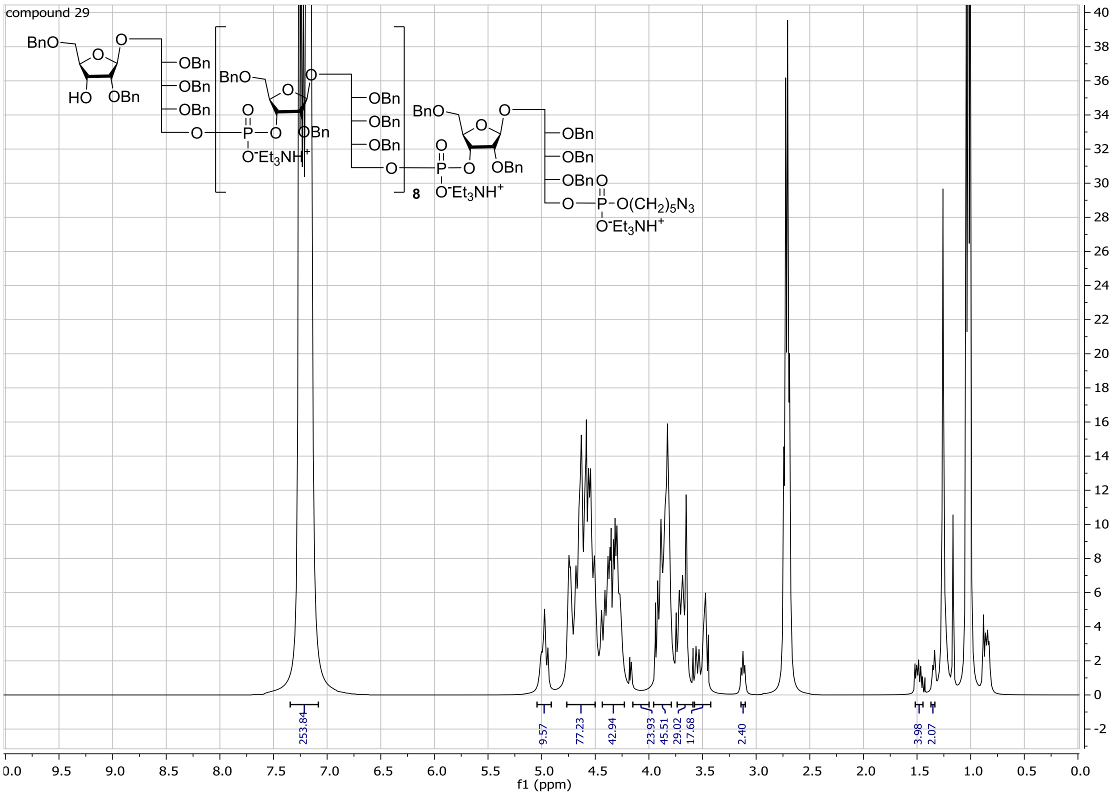

^{13}C NMR (151 MHz, $CDCl_3$)

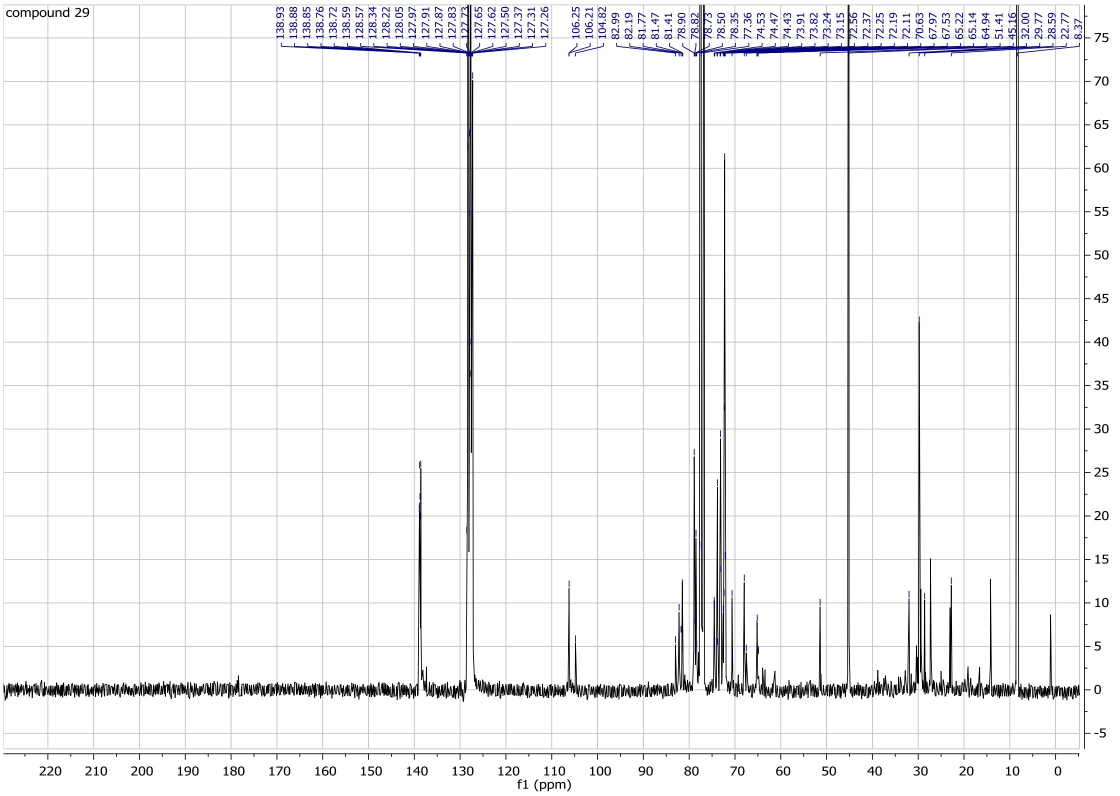

^{31}P NMR (243 MHz, $CDCl_3$)

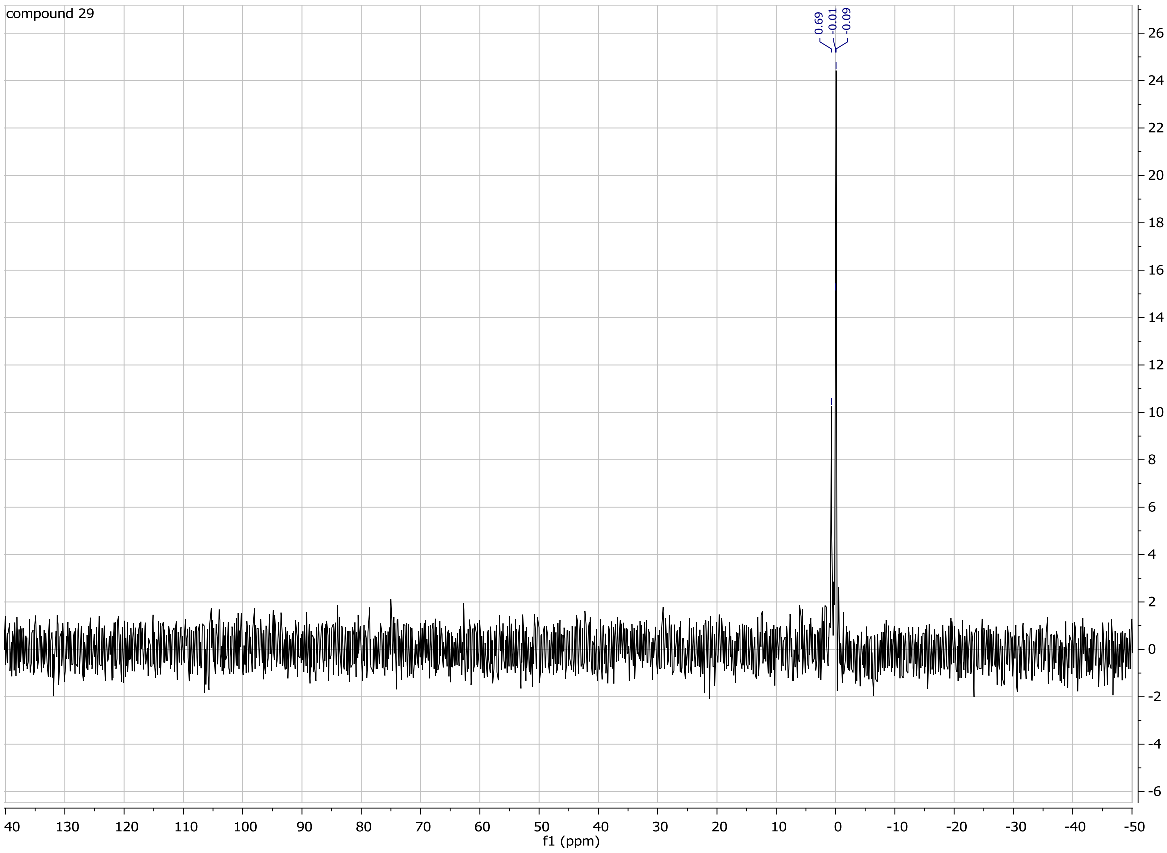

HSQC (600 MHz, $CDCl_3$)

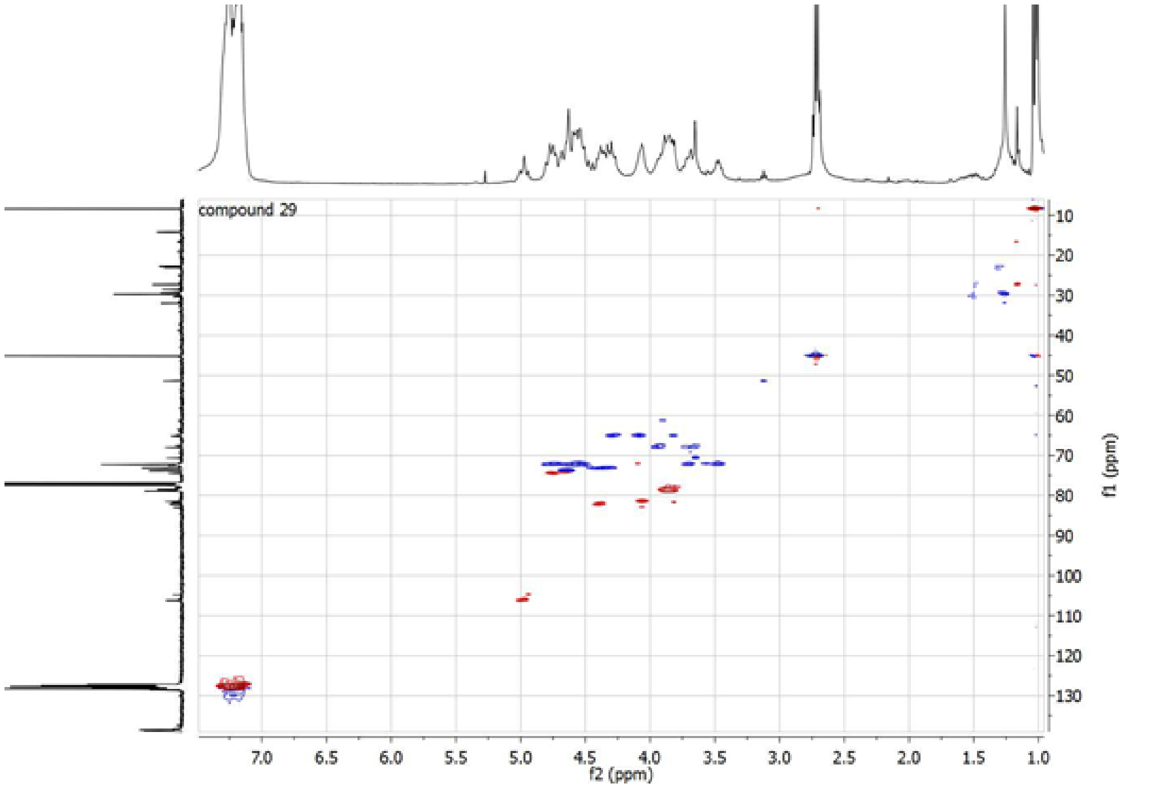

Compound 4

^{1}H NMR (600 MHz, D$_2$O)

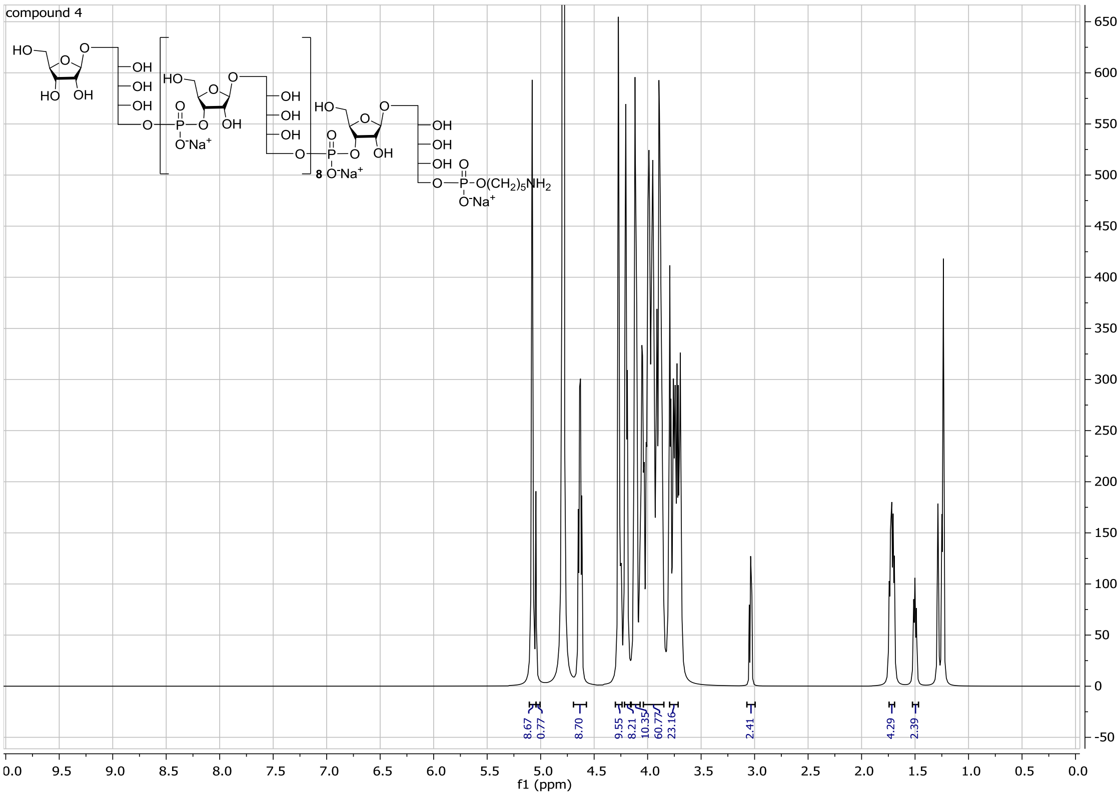

^{13}C NMR (151 MHz, D$_2$O)

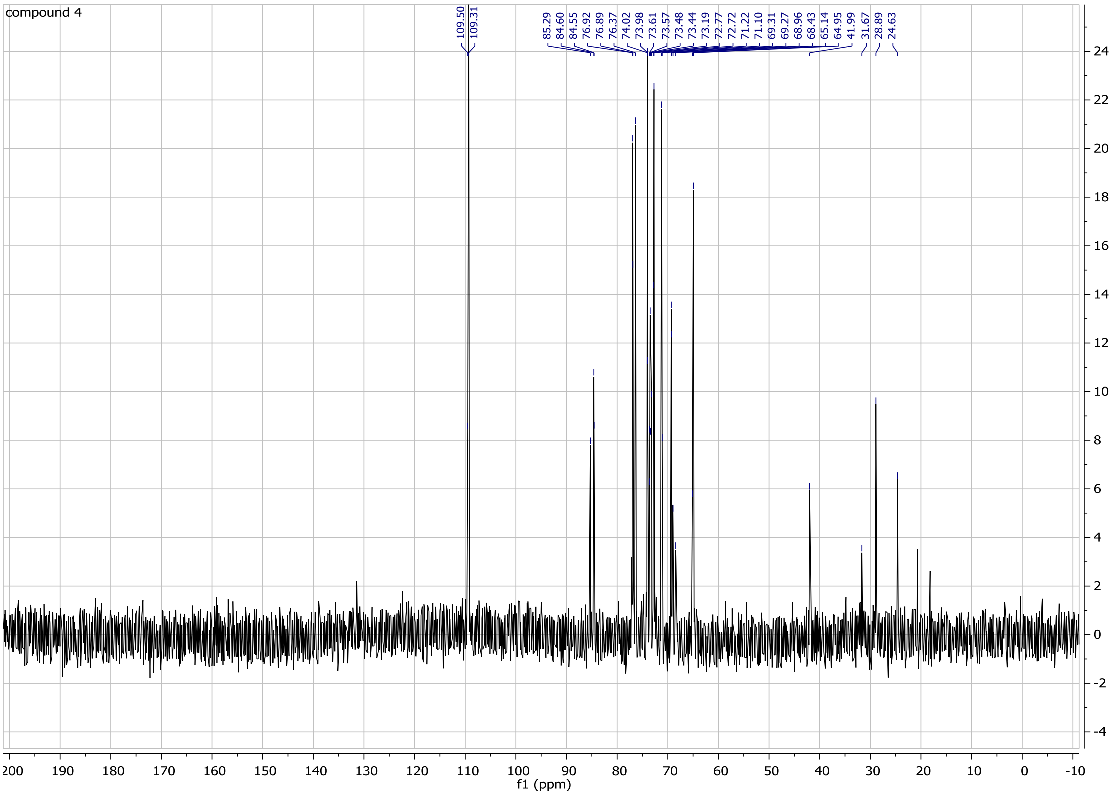

^{31}P NMR (243 MHz, D_2O)

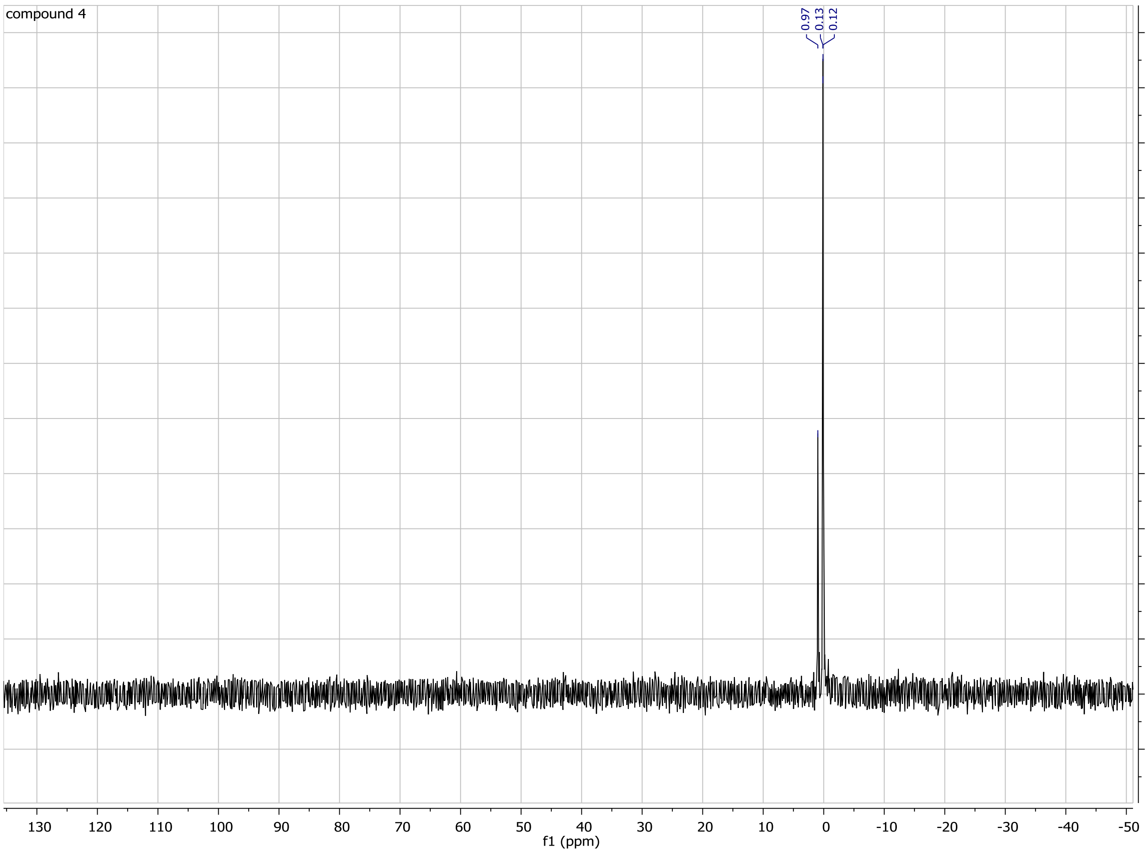

^{1}H-^{1}H COSY (600 MHz, D_2O)

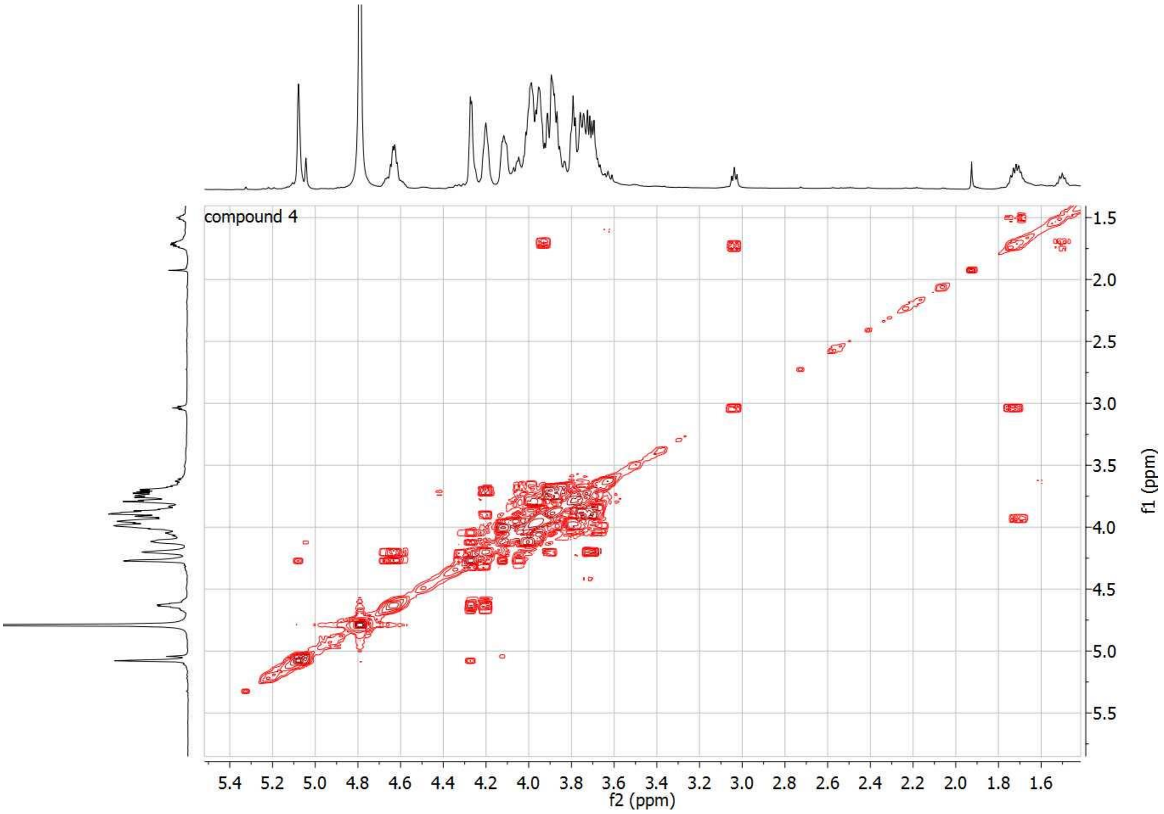

^1H-^1H TOCSY (600 MHz, D_2O)

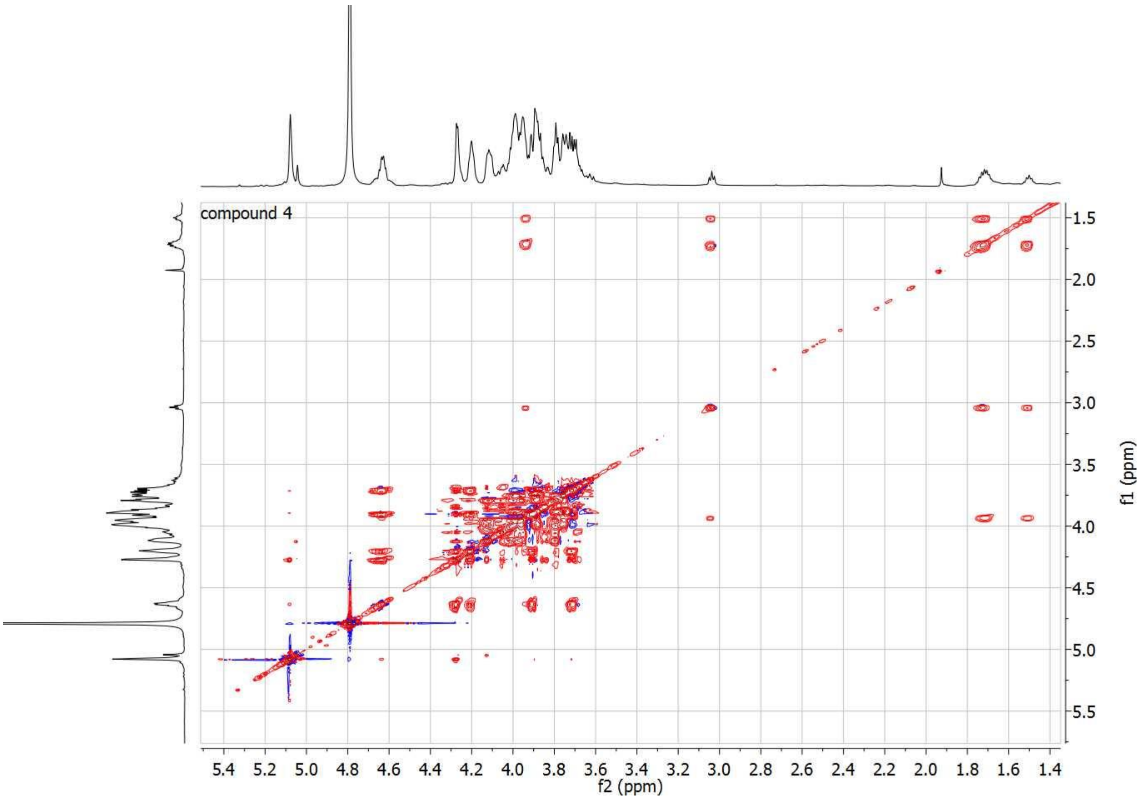

HSQC (600 MHz, D_2O)

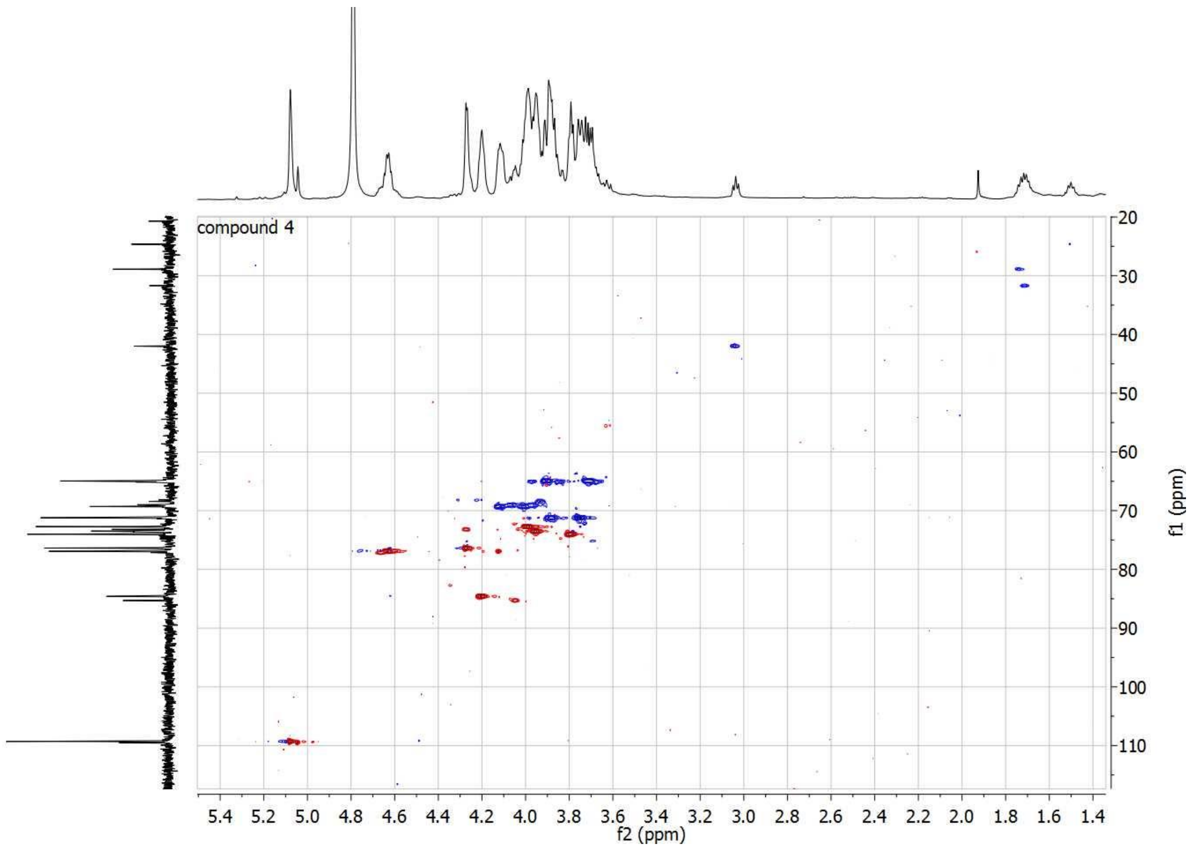

^{1}H-^{1}H TOCSY (600 MHz, D_2O)[6]

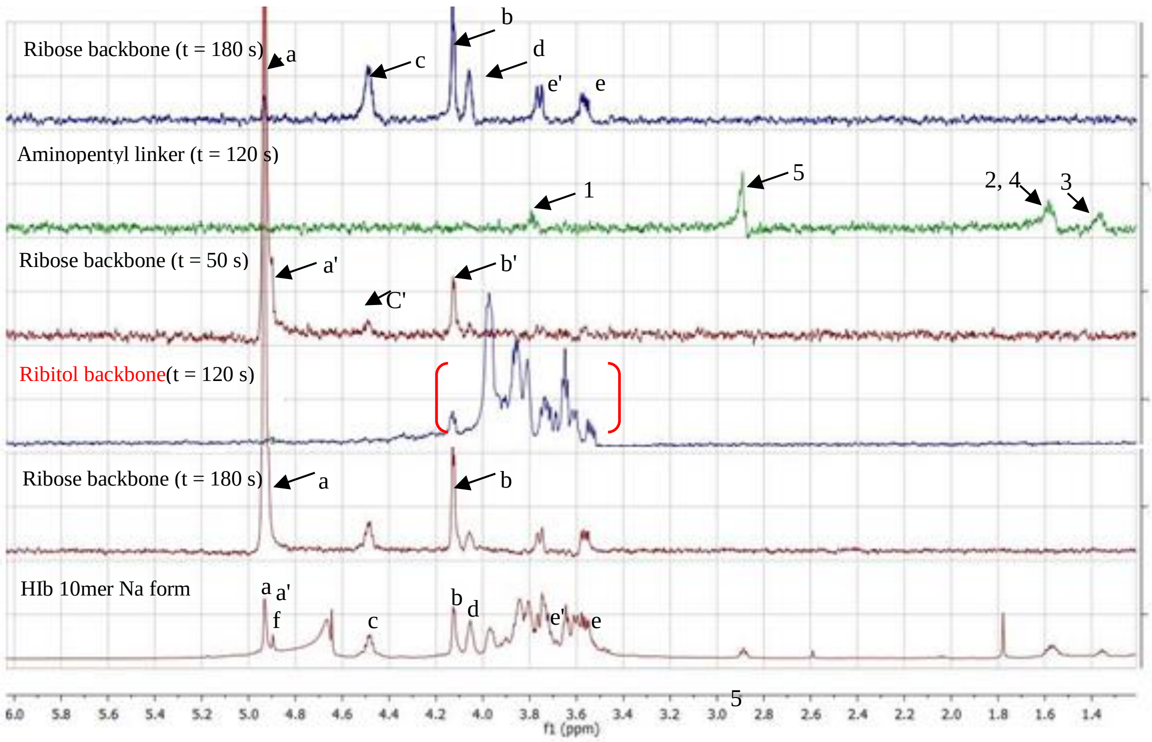

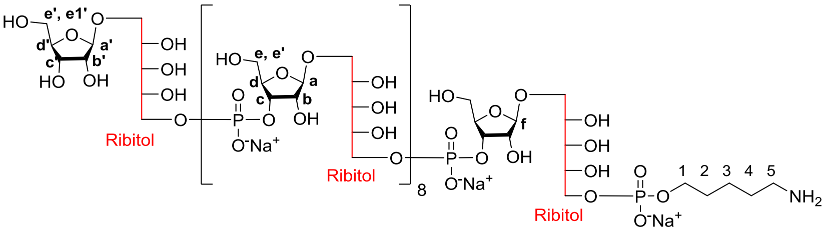

Compound S4

^{1}H NMR (600 MHz, D_2O)

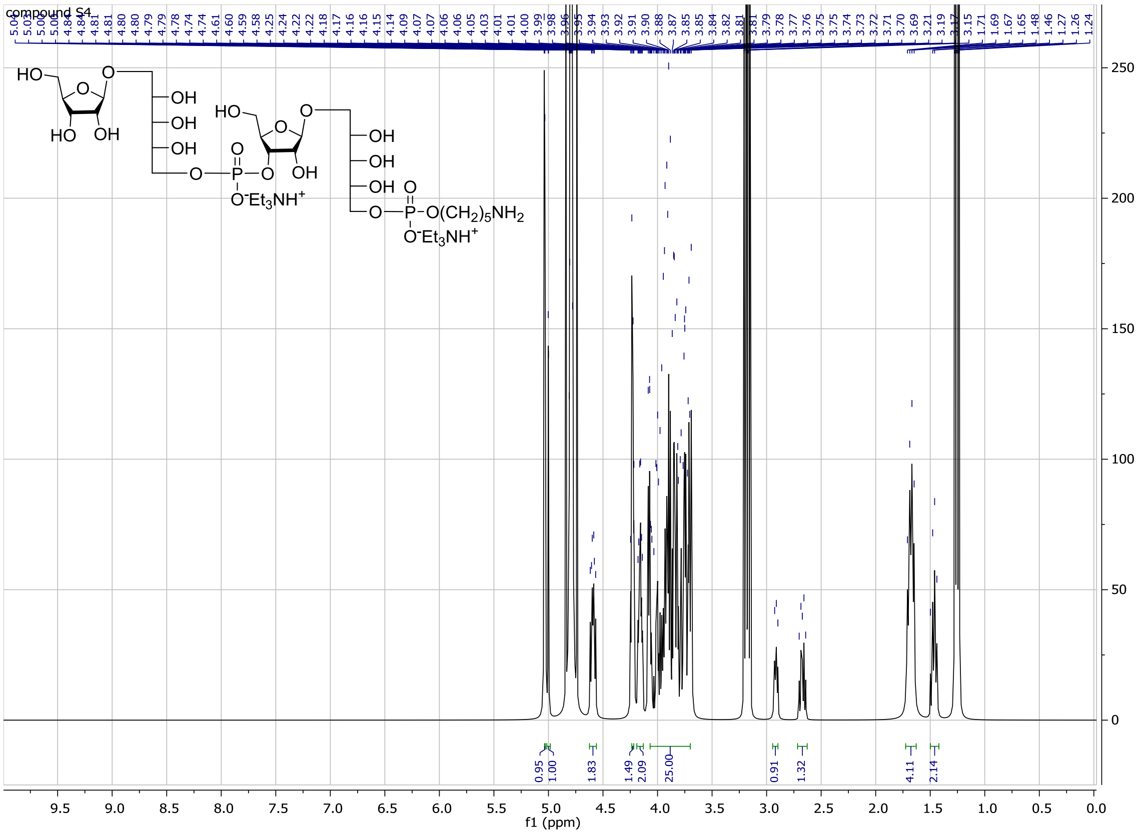

^{31}P NMR (243 MHz, D_2O)

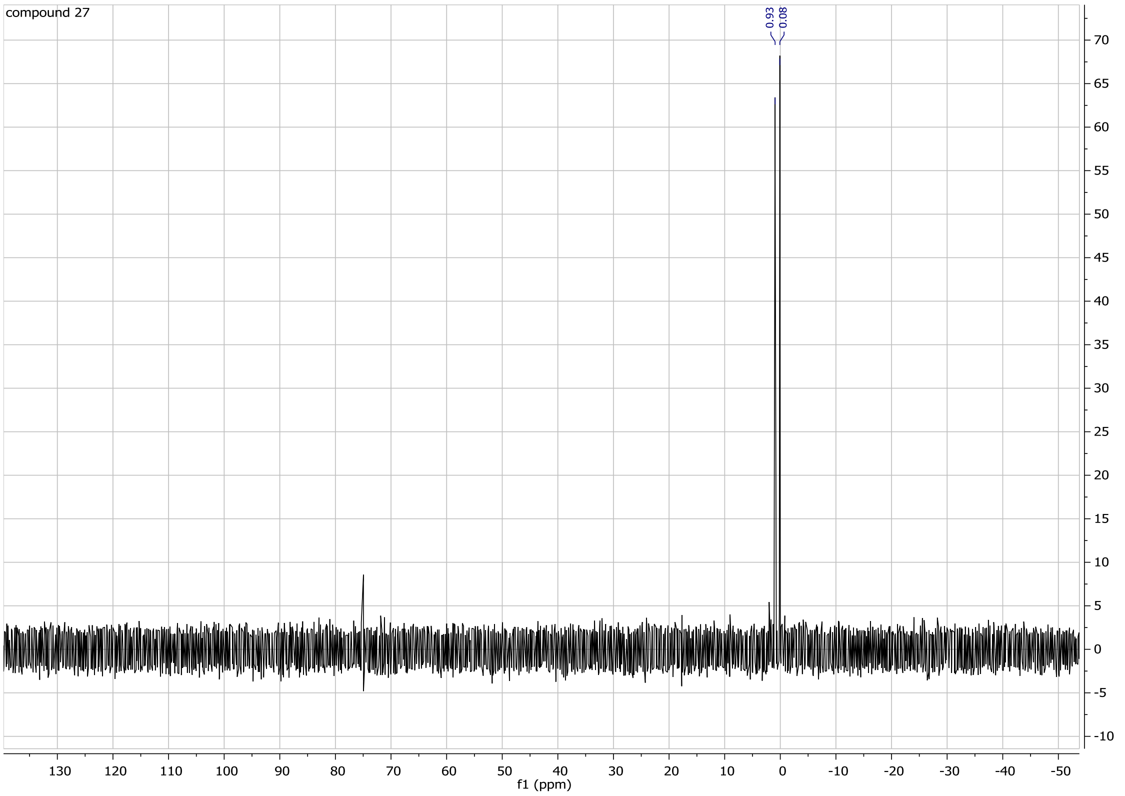

Compound 1-SH

^{1}H NMR (600 MHz, D_2O)

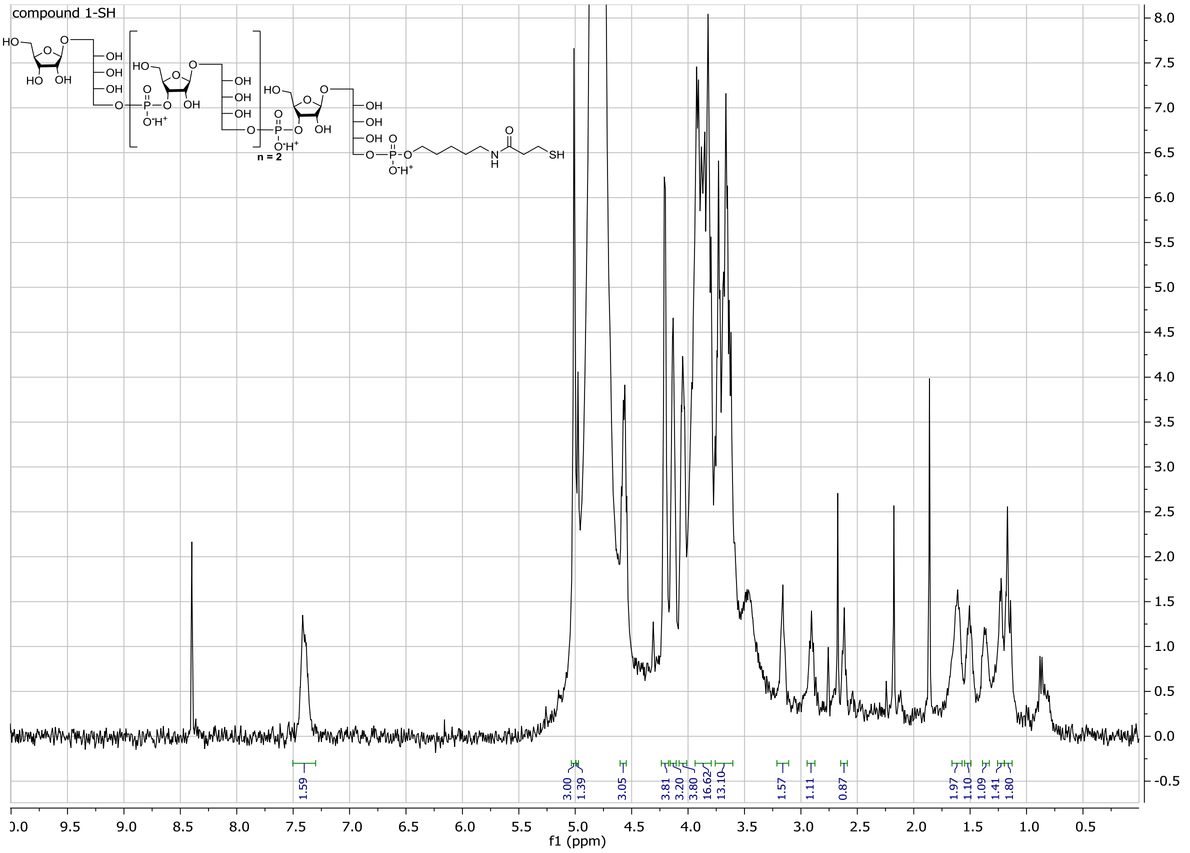

^{31}P NMR(243 MHz, D_2O)

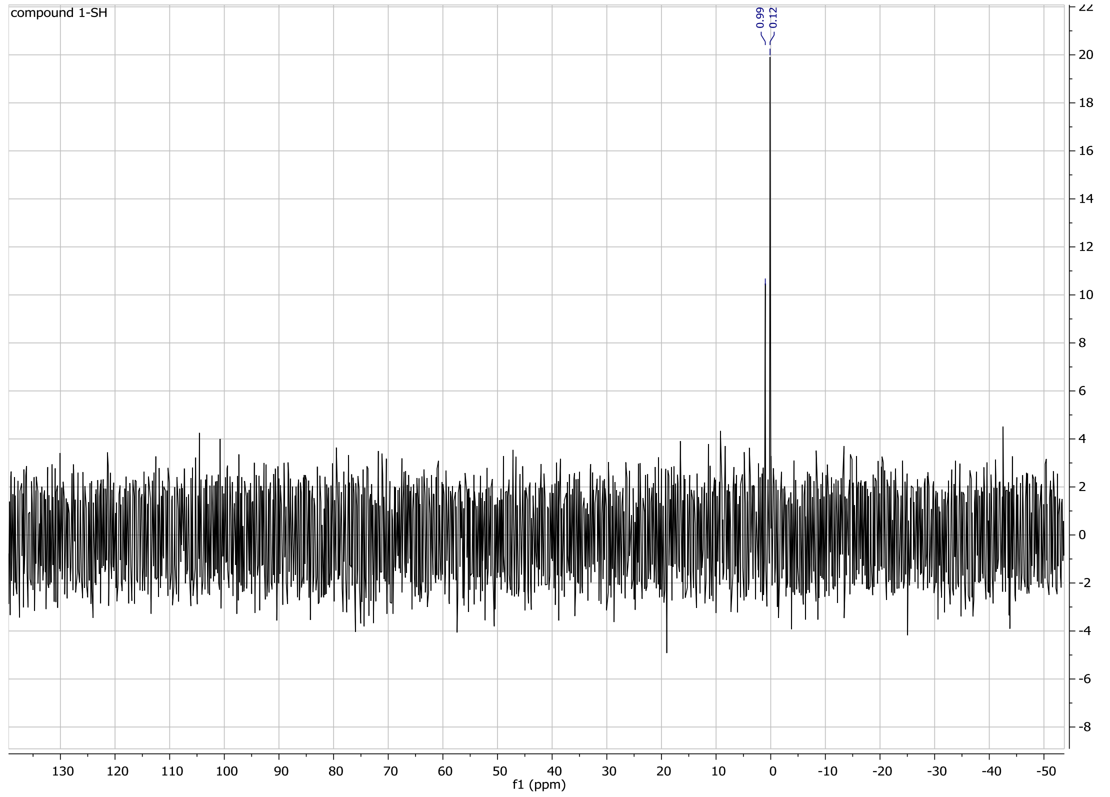

MALDI-TOF

Compound 1-SH

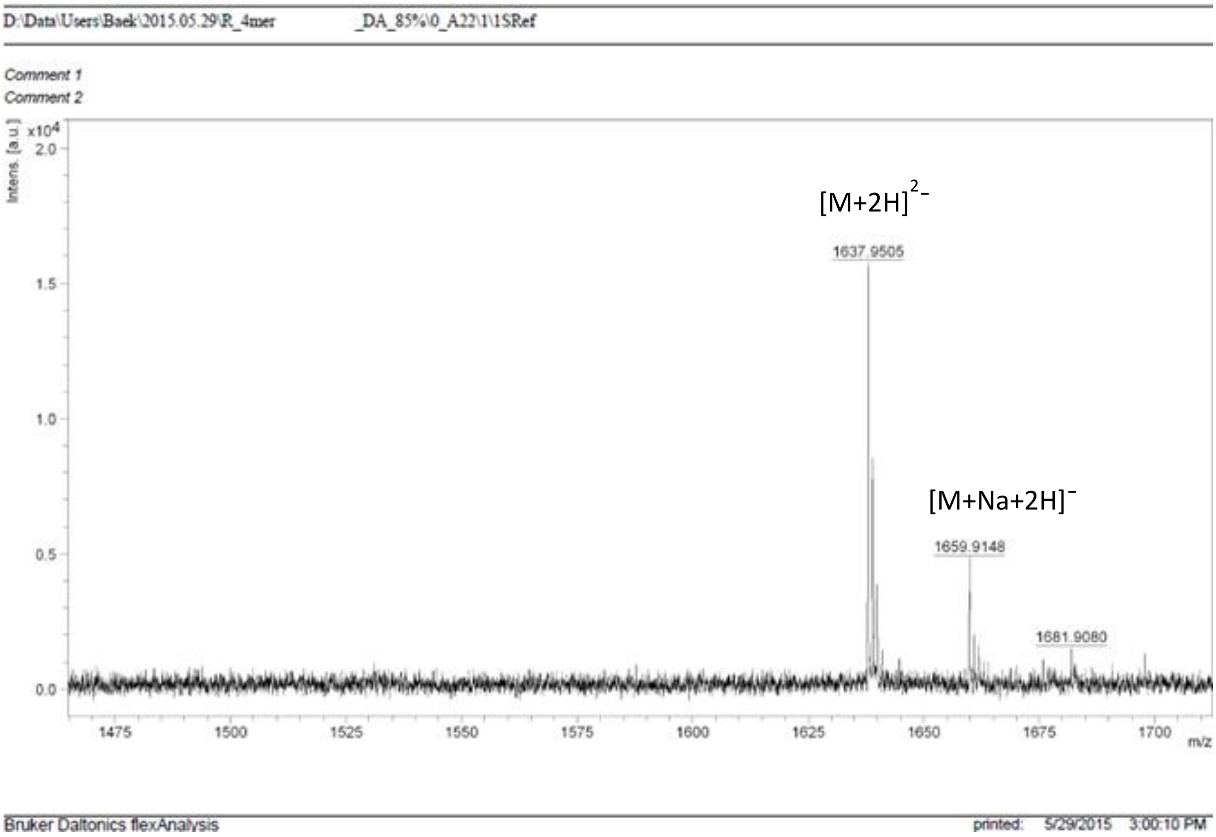

Supplemental biological experimental procedures

Glycan Array preparation

Synthetic glycans, carrier protein CRM197 (Pfenex), and Hib PRP (1st international standard, NIBSC, UK) were dissolved in phosphate buffered saline (PBS, pH 7.4) and printed to NHS activated glass slides (CodeLink slides, Surmodics, Eden Prairie, MN, USA) using an S3 microarray spotter (Scienion, Berlin, Germany) as described earlier.[7] Each slide was printed with 64 identical fields to allow multiple sample analyses (Figure S1a). Subsequent to printing, slides were incubated overnight at room temperature (RT) in a humidity saturated chamber followed by incubation for 2 h in 100 mM ethanolamine containing 50 mM sodium phosphate pH 7.4, for the purpose of quenching remaining reactive NHS esters. Slides were washed three times with water, dried by centrifugation (300Xg, CombiSlide system, Eppendorf, Germany), and stored dry at 4 °C until use.

Glycan Array reference serum analysis

Slides were blocked with 3% bovine serum albumin in PBS (3% BSA-PBS) for 30 min at room temperature, washed twice with PBS and dried by centrifugation. A FlexWell 64 grid (Grace Biolabs, Bend, OR, USA) was attached to allow incubation of single fields. Sera (Human reference serum, NIBSC, UK, Product-ID: 09/222 or rabbit typing serum for Hib, SSI Diagnostica, Denmark, Product-ID: 3462) were diluted in 3% BSA-PBS containing 0.1% Tween-20 (BSA-PBS-T) and, in inhibition experiments, different concentrations of isolated PRP (1st WHO International Standard,[8] NIBSC, Product-ID: 02/208). Dilutions were incubated for 15 min at 37 °C and centrifuged for 5 min at 3220Xg. Supernatants were applied to the arrays and incubated for 1 h at RT. Single wells were washed three times with PBS containing 0.1% Tween-20 (PBS-T) and subsequently incubated for 30 min with the secondary antibody diluted in BSA-PBS-T. The following antibodies were used: goat anti-human IgG-Fc AlexaFluor 488 (Dianova, Product-ID 109-545-098) and goat anti-rabbit IgG FITC (Abcam, Product-ID ab6717), both diluted 1:400. Wells were washed twice with PBS-T. The incubation grid was removed and the entire slide washed twice in a Petri dish with PBS, then rinsed with water and dried by centrifugation. The slide was scanned using a GenePix 4300A microarray scanner (Molecular Devices, Sunnyvale, CA, USA). Fluorescence intensities were evaluated with GenePix Pro 7.2 software from the spots printed with 200 µM of glycan (Molecular Devices), yielding values for the mean of the spot using the same circle diameter for all spots subtracted by the mean of the local background. Calculations to obtain mean values of replicate spots and standard deviation (SD) were performed and graphs plotted with SigmaPlot 13 (Systat Software Inc., San Jose, CA, USA).

Glycoconjugate preparation and analysis

Generating maleimide containing CRM197 and oligosaccharide-protein conjugates

Carrier protein CRM197 was dissolved in pH 7.4 PBS buffer (1 mg/mL), and N-succinimidyl 3-maleimidopropionate (SMP) (0.23 mg) was added to the solution. The solution was stirred gently at room temperature for 2 h. The mixture was then diluted with sterile water and centrifuged against 3 changes of sterile water over an Amicon Ultra-0.5, 10 kDa membrane. The solution was stored at 0 ºC. The obtained CRM-maleimide was characterized by MALDI-TOF (positive mode, matrix 2',4',-dihydroxyacetophenone) analysis to determine the maleimide incorporation number. In general,

maleimide was conjugated with CRM197 at an average stoichiometry of 10. Activated maleimide-CRM197 was dissolved in pH 7.4 PBS buffer (1 mg/mL), and compounds **1-SH**, **2-SH**, **3-SH**, and **4-SH** were added to the solution in a 50-fold excess. The mixtures were stirred at room temperature for 3 h, followed by dilution with sterile water and centrifugation against 3 changes of sterile water over an Amicon Ultra-0.5, 10kDa membrane. The solution was collected into a sterile tube. The obtained Hib conjugates were characterized by MALDI-TOF (negative mode, matrix sinapinic acid) analysis to determine the incorporation stoichiometry of the oligosaccharides. Protein concentration was determined using the Micro BCA Protein Assay Kit (Thermo Scientific Pierce) according to the manufacturer's recommendation.

SDS-Polyacrylamide gel electrophoresis and Western Blot

Conjugates and unmodified CRM197 were diluted in Laemmli sample buffer at concentrations of 5 µg or 0.25 µg protein in 12 µL volume and heated to 95 °C for 5 min. Samples were separated on 10% polyacrylamide gels with 15 wells (BioRad Mini Protean Tetra Cell electrophoresis system). ColorPlus Prestained Protein Ladder (New England Biolabs) was used as a protein size marker. Gels loaded with 5 µg protein per well were stained with Coomassie, gels loaded (in duplicate) with 0.25 µg protein were electroblotted onto 0.22 µm pore size nitrocellulose membranes (Amersham HyBond-ECL, GE Healthcare). Blots were blocked by rocking with blocking solution (4% nonfat milk powder (AppliChem) in PBS) for 30 min at room temperature followed by primary antibody incubation at 4 °C overnight. The following primary antibodies were used on the physically separated membranes: goat anti-diphtheria toxin (Pierce antibodies, Thermo Scientific, Product-ID: PA1-7212) diluted 1:1000 in blocking solution and rabbit typing serum for Hib (SSI Diagnostica, Denmark, Product-ID: 3462) diluted 1:2500 in blocking solution. Membranes were washed three times for 5 min with PBS-T, incubated for 1 h at room temperature with secondary antibody diluted 1:5000 in blocking solution (rabbit anti-goat IgG HRP, Sigma-Aldrich, Product-ID: A4174 or goat anti-rabbit IgG HRP, Sigma Aldrich, Product-ID: A6154), and washed again three times with PBS-T. Bound antibody was visualized with ECL Prime Western Blot reagent (GE Healthcare) used according to the manufacturer's instructions in a LAS-4000 Mini imaging system (FujiFilm). Images using white illumination were also taken for size marker visibility.

HPAEC-PAD

High-Performance Anion-Exchange Chromatography with Pulsed Amperometric Detection (HPAEC-PAD) analysis was based on a published protocol with isocratic separation of reaction products after alkaline hydrolysis of the phosphodiester bonds connecting the repeating disaccharide units.[9] For alkaline hydrolysis, samples (WHO PRP 1st international standard at multiple concentrations,[8] conjugates at multiple dilutions, controls) in water were mixed with equal amounts of 200 mM sodium hydroxide freshly prepared by dilution from 50% sodium hydroxide (J.T.Baker, Thermo Fisher Scientific) followed by overnight incubation at 25 °C. Samples were filtered through Amicon 10k ultrafiltration devices (Merck Millipore) that were washed three times with 100 mM sodium hydroxide before use to remove residual glycerol. The filtrate was then used for chromatographic analysis.

Analysis of the samples was performed on an ICS 5000+ HPAEC-PAD system (Thermo Scientific Dionex) using a PA-20 column (Thermo Scientific Dionex) at 30 °C. The autosampler was cooled to

8 °C during analysis. All eluents were prepared from high purity water (Milli-Q Direct 8 system, Merck Millipore) that was thoroughly degassed by stirring under vacuum and were maintained under helium during analysis for up to one week. The following eluents were employed: Eluent A: Water. Eluent B: 1 M sodium acetate containing 250 mM sodium hydroxide. Analytical runs were performed for 15 min under isocratic conditions of 85% A, 15% B at a flow rate of 400 µL/min. The electrochemical detector was equipped with a conventional gold electrode. Signals were obtained at 2 Hz with the recommended standard quadruple pulse sequence for carbohydrates. The peak between minute 6 and 7 corresponding to the concentration-dependent signal for the PRP standard was integrated using Chromeleon 7 (Thermo Scientific Dionex) (see Figure S2c). After each, six or fewer, analytical runs the following gradient procedure was employed to remove any tightly bound material, followed by a blank injection of 100 mM sodium hydroxide under analytical isocratic conditions: 0-10 min: 5-50% B at 0.4 -0.2µL/min; 10-25 min at 0.2µL/min: 50% B; 25-35 min: 50-10% B at 0.2 -0.4µL/min; 35-50 min: 10-15% B at 0.05 -0.4µL/min; 50-55 min: 15% B at 0.4µL/min.

A linear calibration was performed with SigmaPlot 13 using the different concentrations of hydrolyzed PRP standard to calculate the PRP concentrations in the diluted glycoconjugate samples (see Figure S2d for calibration example). Only conjugate dilutions that gave peak areas within the range of the PRP concentration series (0.33 µg/mL to 27 µg/mL) were used for concentration calculation. The obtained values for each dilution were multiplied with the respective dilution factor and a mean of different dilutions and replicates was calculated (at least two, up to six chromatographic runs per conjugate). The reported value is the calculated mean multiplied with a correction factor to account for the non-visibility of the repeating unit of the short constructs on HPAEC-PAD after alkaline hydrolysis, due to the lack of a phosphate group (Factors: 4/3 for 4mer, 6/5 for 6mer, 8/7 for 8mer, 10/9 for 10mer).[9]

Rabbit immunizations

The immunization procedure was based on published protocols.[10] Immunization studies were approved by the responsible governmental authorities (Landesamt für Landwirtschaft, Lebensmittelsicherheit und Fischerei Mecklenburg-Vorpommern) and performed by BioGenes GmbH, Berlin, Germany, according to all applicable international regulations (European Union (EU) Directive 2010/63/EU) concerning housing and handling. The glycoconjugates were diluted in PBS without adjuvant and injected subcutaneously at multiple injection sites in four Zika rabbits per group. A dose of 5 µg saccharide, based on calculations from BCA and MALDI-MS, was used per dose. A total of six groups were immunized: one each with a glycoconjugate of size-defined sPRP, one group served as a CRM197 negative control, and one, immunized with the commercial vaccine ActHIB, served as a positive control. The CRM197 negative control group received 45 µg protein per dose corresponding to the highest amount of carrier protein in the glycoconjugate groups. A primary immunization at day 0 was followed by booster doses at days 14 and 28. The reported antibody levels were derived from serum collected on day 35, one week after the second booster shot.

Post-immune serum analysis

Glycan array

Slides were blocked for 30 min with 1% BSA-PBS, washed twice with PBS, and dried by centrifugation. Multiwell grids were attached, and the sera of single rabbits diluted 1:10 to 1:640 in 1% BSA-PBS applied to the slides. After incubation for 1 h at room temperature, wells were washed three times with PBS-T, followed by application of the secondary antibody solution (anti-rabbit IgG FITC and anti-rabbit IgM AlexaFluor 647 (Abcam Product-ID ab150095) diluted 1:400 in 1% BSA-PBS). After 30 min of incubation, each slide was washed twice with PBS-T, the multiwell grid was removed, the slide washed with PBS, rinsed with water, and dried by centrifugation. Slides were scanned using a GenePix 4300A microarray scanner (Molecular Devices, Sunnyvale, CA, USA). Fluorescence intensities were evaluated as described for the reference serum analysis.

ELISA

Costar high binding ELISA plates (Corning) were coated overnight with 10 µg/mL Hib PRP in PBS, washed twice with PBS-T and blocked with 10% fetal bovine serum (FBS, PAN Biotech) in PBS for 3 h at 37 °C. Plates were incubated with serum diluted in 10% FBS-PBS for 1.5 h at room temperature and subsequently washed three times with PBS-T. Secondary goat anti-rabbit IgG-HRP diluted 1:5000 in 10% FBS-PBS was added and incubated for 1 h at room temperature. Plates were washed three times with PBS-T. TMB substrate (1-Step Ultra TMB-ELISA Substrate Solution, Thermo Scientific Pierce) was added and plates were incubated for 10 min in the dark. The reaction was stopped by the addition of 2% sulfuric acid. Optical density was measured at 450 nm with a Tecan infinite M200 microplate reader.

References

1 Z. Yuan Wang and G. Just, *Tetrahedron Lett.*, 1988, **29**, 1525.
2 C. Laval and G. Just, *Tetrahedron*, 1990, **46**, 151.
3 I. Chiu-Machado, J. C. Castro-Palomino, O. Madrazo-Alonso, C. Lopetegui-Palacios and V. Verez-Bencomo, *J. Carbohydr. Chem.*, 1995, **14**, 551.
4 V. Verez-Bencomo, V. Fernández-Santana, E. Hardy, M. E. Toledo, M. C. Rodríguez, L. Heynngnezz, A. Rodriguez, A. Baly, L. Herrera, M. Izquierdo, A. Villar, Y. Valdés, K. Cosme, M. L. Deler, M. Montane, E. Garcia, A. Ramos, A. Aguilar, E. Medina, G. Toraño, I. Sosa, I. Hernandez, R. Martínez, A. Muzachio, A. Carmenates, L. Costa, F. Cardoso, C. Campa, M. Diaz and R. Roy, *Science*, 2004, **305**, 522.
5 S. Nielsen, C. M. Pedersen, S. G. Hansen, M. D. Petersen, S. Sinning, O. Wiborg, H. H. Jensen and M. Bols, *Bioorg. Med. Chem.*, 2009, **17**, 4900.
6 P. Hoogerhout, C. W. Funke, J.-R. Mellema, G. N. Wagenaars, A. van Boeckel, C. A., D. Evenberg, J. T. Poolman, Lefeber, A. W. M., van der Marel, G. A. and Van Booma, J. H., *J. Carbohydr. Chem.*, 1988, **7**, 399.
7 (a) C. L. Pereira, A. Geissner, C. Anish and P. H. Seeberger, *Angew. Chem. Int. Ed.*, 2015, **54**, 10016; (b) A. Geissner, C. L. Pereira, M. Leddermann, C. Anish and P. H. Seeberger, *ACS Chem. Biol.*, 2016, **11**, 335;
8 F. Mawas, B. Bolgiano, P. Rigsby, D. Crane, D. Belgrave and M. J. Corbel, *Biologicals*, 2007, **35**, 235.
9 A. de Haan, R. M. F. van der Put and M. Beurret, *Biomed. Chromatogr.*, 2013, **27**, 1137.
10 V. Fernandez-Santana, F. Cardoso, A. Rodriguez, T. Carmenate, L. Pena, Y. Valdes, E. Hardy, F. Mawas, L. Heynngnezz, M. C. Rodriguez, I. Figueroa, J. Chang, M. E. Toledo, A. Musacchio, I. Hernandez, M. Izquierdo, K. Cosme, R. Roy and V. Verez-Bencomo, *Infect. Immun.*, 2004, **72**, 7115.
